# Comparison and Analysis on the Existing Single-Herbal Strategies against Viral Myocarditis

**DOI:** 10.1155/2021/9952620

**Published:** 2021-08-07

**Authors:** Yu Cao, Yang Liu, Tian Zhang, Jing Pan, Wei Lei, Boli Zhang

**Affiliations:** ^1^Institute of Traditional Chinese Medicine, Tianjin University of Traditional Chinese Medicine, No. 10 Poyanghu Road, Tianjin 301617, China; ^2^School of Chemical Engineering and Technology, Tianjin University, No. 135 Yaguan Road, Tianjin 300350, China; ^3^State Key Laboratory of Dao-di Herbs, National Resource Center for Chinese Materia Medica, China Academy of Chinese Medical Sciences, No. 16 Neinan Street, Beijing 100700, China; ^4^Department of Reproductive Medicine, Inner Mongolia Maternal and Child Health Care Hospital, No. 18 North Second Ring Express Road, Hohhot 010020, China

## Abstract

**Purpose:**

Herbal medicine is one of crucial symbols of Chinese national medicine. Investigation on molecular responses of different herbal strategies against viral myocarditis is immeasurably conducive to targeting drug development in the current international absence of miracle treatment.

**Methods:**

Literature retrieval platforms were applied in the collection of existing empirical evidences for viral myocarditis-related single-herbal strategies. SwissTargetPrediction, Metascape, and Discovery Studio coordinating with multidatabases investigated underlying target genes, interactive proteins, and docking molecules in turn.

**Results:**

Six single-herbal medicines consisting of Huangqi (*Hedysarum Multijugum Maxim*), Yuganzi (*Phyllanthi Fructus*), Kushen (*Sophorae Flavescentis Radix*), Jianghuang (*Curcumaelongae Rhizoma*), Chaihu (*Radix Bupleuri*), and Jixueteng (*Spatholobus Suberectus Dunn*) meet the requirement. There were 11 overlapped and 73 unique natural components detected in these herbs. SLC6A2, SLC6A4, NOS2, PPARA, PPARG, ACHE, CYP2C19, CYP51A1, and CHRM2 were equally targeted by six herbs and identified as viral myocarditis-associated symbols. MCODE algorithm exposed the hub role of SRC and EGFR in strategies without Jianghuang. Subsequently, we learned intermolecular interactions of herbal components and their targeting heart-tissue-specific CHRM2, FABP3, TNNC1, TNNI3, TNNT2, and SCN5A and cardiac-myocytes-specific IL6, MMP1, and PLAT coupled with viral myocarditis. Ten interactive characteristics such as *π*-alkyl and van der Waals were modeled in which ARG111, LYS253, ILE114, and VAL11 on cardiac troponin (TNNC1-TNNI3-TNNT2) and ARG208, ASN106, and ALA258 on MMP1 fulfilled potential communicating anchor with ellagic acid, 5*α*, 9*α*-dihydroxymatrine, and leachianone g via hydrogen bond and hydrophobic interaction, respectively.

**Conclusions:**

The comprehensive outcomes uncover differences and linkages between six herbs against viral myocarditis through component and target analysis, fostering development of drugs.

## 1. Introduction

Herbal medicine is the keystone to uphold the existence and development of Traditional Chinese Medicine (TCM); besides China, herbs are also widely applied to improve human health in Sumer and ancient Egypt for thousands of years [1–4]. Currently, not only in China but also in Japan, Korea, and several Southeast Asian countries, herbal medicine is gaining increasing acceptance from public health and medical field in Western countries because of the recognized therapeutic properties of herbs [5].

There are several clinical and experimental evidence of herbal medicinal efficacy on angiocardiopathy, diabetes, cancer, and other inflammatory or viral diseases. A cardiovascular investigation involving 781 patients indicated that the intake of standardised garlic extract (600 to 900 mg per day) is coupled with 0.41 mmol/L reduction in serum cholesterol level [6]. Additionally, garlic extracts have been confirmed to decrease blood pressure and anticlotting bioactivity [7, 8]. The metformin (biguanide drug) acquired from French lilac, *Galega officinalis*, is a prevalent first-line treatment for diabetes [3]. A prior report also manifests that cinnamon contributes to improving glucose tolerance in patients with type 2 diabetes mellitus [9]. Ginger can weaken the inflammatory process, and its constituents in part are dual inhibitors of the arachidonic-acid metabolism in the inflammation-related pathway [10]. Epidemiological research has proved that, with ingesting foods rich in polyphenols such as ginger, people have lower risk of inflammatory disease [11]. A rat study exhibits that the natural anti-inflammatory ingredients silymarin, curcumin, and quercetin, as effective as nonsteroidal antiphlogistic indomethacin, suppress aberrant crypt foci [12]. Implicated in human colon cancer, geraniol is an acyclic monoterpene alcohol derived from lemon grass (*Cymbopogon citratus*) and dampens polyamine biosynthesis and cell growth [13]. The study of both Chinese medicine and Indian Ayurvedic medicine involves in management of memory and concentration. Ginkgo surveys show that it allows for ameliorations of cognitive decline in dementia and memory function in healthy adults [14, 15]. *Artemisia capillaris* is a famous traditional Chinese herb, and its extract enynes are responsible for the effect of anti-hepatitis B virus significantly inhibiting viral DNA replication [16]. Through treatments of 40 and 80 *μ*g/mL doses of *Sambucus nigra* fruit extract, the titer and protein synthesis of H9N2 influenza virus are palpably decreased in the human epithelium cell which reflects the herb interferes with either entry of viruses or release of the virus particle [17].

Myocarditis is an inflammatory cardiomyopathy, symptoms of which include irregular heartbeat, pectoralgia, shortness of breath, and impaired ability to exercise [18]. Compared with toxins, bacterial infections, and autoimmune disorders, viral infection is the biggest cause of myocarditis [18, 19]. The plus-strand RNA virus Coxsackievirus B3 (CVB3) and Coxsackievirus B5, as the members of the Coxsackie B family of the single-stranded RNA viruses, are major pathogens for acute and chronic viral myocarditis [20]. There are other pathogenic viruses, such as adenovirus, polio virus, rubella virus, hepatitis C, Epstein–Barr virus, parvovirus B19, and severe acute respiratory syndrome coronavirus 2 [21]. Research on neonates who developed enterovirus myocarditis mediated by Coxsackie virus B exhibits that the mortality of neonates is 31% and 66% of the survivors develop serious cardiac injury with only 23% of the infants fully recovered [22]. Myocarditis also occurs in patients infected with coronaviruses. For instance, acute myocarditis is reported in the Middle East respiratory syndrome coronavirus outbreak [23]. Autopsy studies reveal that 35% of patients infected with the virus present viral RNA in the myocardium during the outbreak of severe acute respiratory syndrome [24]. In the 12 patients with COVID-19, 5 patients demonstrate viral presence in the myocardium [25]. Similarly, Kang et al. and Tavazzi et al. reported the case of COVID-19 with myocarditis [26, 27]. Influenza A virus led to the deaths of more than 6 hundred thousand people in the United States alone near the end of World War I, whose mortality was more common in the elderly, pregnant women, infants, and in people with chronic diseases such as diabetes mellitus [28, 29]. Myocarditis is one of the characteristics of influenza infection. There is a clear acute myocarditis diagnosed clinically in 10% of cases of influenza, with up to 40% having a conclusive diagnosis on autopsy [30]. Under severe infection, myocarditis is associated with mortality in influenza patients in the intensive care unit [31]. Conversely, the case of dengue hemorrhagic fever complicated by acute myocarditis is rare [32]. A review of 51 cases of myocarditis manifests that the mortality rate is 27% [33]. In addition, fulminant myocarditis cases are reported occasionally [34]. At 11-year follow-up, 93% of patients with fulminant myocarditis are alive compared with 45% of patients with acute nonfulminant myocarditis [35], with higher in-hospital mortality rate in the fulminant group [36]. To be emphasized, cytomegalovirus-associated carditis causes the mortality as high as 60% in the immunosuppressed patients [37]. Viral myocarditis (VMC) is a global health issue; regretfully at present, it still lacks an effective therapeutic strategy. Systemic corticosteroids offer underlying positive effects in people with myocarditis [38]. Medications such as diuretics, beta blockers, and angiotensin-converting enzyme inhibitors are usually used for VMC treatments, but in severe cases, the patients would receive an implantable cardiac defibrillator or heart transplant [18, 19]. It is noteworthy that the VMC is an inducement of death and up to twenty percent of all are due to myocarditis in cases of sudden death of young adults [39].

Although abundant achievements clarify herbs' effectiveness on viruses, the differences of single-herbal strategies have been seldom pursued, especially against VMC. Herein, relying on open-resource platforms and bioinformatics methods, we designed and executed an investigation to compare the chemical compositions, molecular targets, and their interactions of distinct single-herbal strategies potentially coupled with treatment of VMC and attempted to provide inspirations against VMC.

## 2. Materials and Methods

### 2.1. Herb Information Retrieval

To comprehend medical strategies of single herb that treat with a single herb and have been revealed for antiviral activity on VMC, information search was performed by PubMed (https://pubmed.ncbi.nlm.nih.gov) and Web of Science (http://www.webofscience.com), free retrieval engines about the biomedical literature [40, 41]. The keywords for the retrieve referred to the combination of the following terms: viral myocarditis and herb. The literature published in the last twenty years and studied on single herb was considered, while herbs that are actually proven to be effective in cases of viral myocarditis were screened and collected.

### 2.2. Screening of the Herbal Active Component

The Traditional Chinese Medicine Systems Pharmacology Database and Analysis Platform (TCMSP) (https://tcmspw.com/tcmsp.php) displays twelve essential properties such as herbal distribution, absorption, excretion, and metabolism and is invoked to completely view herbal medicines based on the framework of systems pharmacology [42]. The herbal Latin names annotated by the TCMSP were employed in the present work. PubChem (https://pubchem.ncbi.nlm.nih.gov) as a public repository presents mostly small and also larger molecule data such as chemical structures, safety, and toxicity and is ordinarily applied to chemical biology investigation and drug discovery [43]. Combined with the two digital resources, the active components were elected in the light of the benchmarks of parameters oral bioavailability ≥30%, drug-likeness ≥0.18, and consistent PubChem Cid or InChIKey, but without nonlive status [44, 45].

### 2.3. Target Prediction

SwissTargetPrediction (http://www.swisstargetprediction.ch) is an analysis platform of ligand-based target prediction on a bioactive small molecule and delivers services to more than one hundred countries worldwide [46]. Taking molecular shape and chemical structure as a basis, the platform merges distinct measures of chemical similarity and achieves exact target prediction [47]. The herbal compound-target network was visualized through Cytoscape v3.6.0.

### 2.4. Viral Myocarditis-Centric Symbol

GeneCards (https://www.genecards.org) as an integrative and searchable database supplies inclusive, authoritative compendium of annotative information about human genes. The knowledge database integrates gene-related data from nearly one hundred and fifty web sources, embodying genomic, transcriptomic, proteomic, genetic, clinical, and functional information [48]. Viral myocarditis was input as the content of keywords, and the disease symbols were assembled subsequently.

### 2.5. Enrichment Analysis

The web-based resource Metascape (https://metascape.org) provides a comprehensive annotation and analysis of gene list to experimental biologists [49]. The enrichment analyses of targets were employed to detect the Gene Ontology (GO) term, Kyoto Encyclopedia of Genes and Genomes (KEGG) pathway, protein-protein interaction (PPI) network, and tissue- and cell-specific location by Metascape. The *p* value less than 0.05 was defined as statistically significant.

### 2.6. Protein-Component Interaction

The Protein Data Bank (PDB) (http://www1.rcsb.org/) archives and shares experimentally determined 3D structures of nucleic acids, proteins, and complex assemblies derived from crystallography, nuclear magnetic resonance spectroscopy, and electron microscopy [50]. We used the open-accessible PDB to collect the molecular structure of protein targeted by the herbal component with the *Homo sapiens* setting checked. The receptor-ligand interaction between the target protein and active component was carried out by the software BIOVIA Discovery Studio v16.1.0 [51].

## 3. Results

### 3.1. Six Single-Herbal Strategies and Natural Ingredients

**Figure 1:**
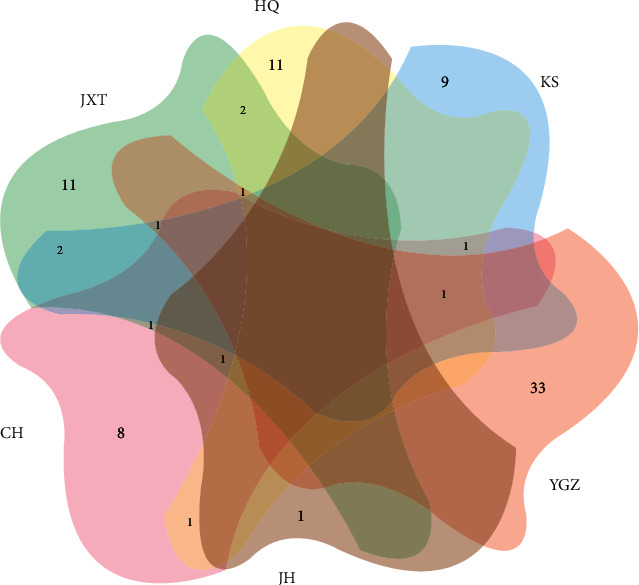
Common and unique herbal components in different strategies.

Based on the previous experimental evidence [52–57], we screened six single-herbal strategies including Huangqi (HQ, *Hedysarum Multijugum Maxim*), Yuganzi (YGZ, *Phyllanthi Fructus*), Kushen (KS, *Sophorae Flavescentis Radix*), Jianghuang (JH, *Curcumaelongae Rhizoma*), Chaihu (CH, *Radix Bupleuri*), and Jixueteng (JXT, *Spatholobus Suberectus Dunn*) as the qualified objects to analyse. In line with the preestablished criteria, we collected 17 (e.g., mairin and jaranol), 14 (e.g., ellagic acid and beta-sitosterol), 36 (e.g., inermine and sophocarpine), 2 (stigmasterol and CLR), 13 (e.g., linoleyl acetate and baicalin), and 19 (e.g., formononetin and calycosin) constituents in HQ, YGZ, KS, JH, CH, and JXT in turn (Table S1). Further statistical result illustrated that 11, 9, 33, 1, 8, and 11 unique components were independently identified in HQ, YGZ, KS, JH, CH, and JXT (Table S2). Contrary to that, quercetin (MOL000098) is common in the HQ, YGZ, CH, and KS, as well as kaempferol (MOL000422), formononetin (MOL000392), luteolin (MOL000006), and stigmasterol (MOL000449) overlapped in three different strategies and isorhamnetin (MOL000354), calycosin (MOL000417), (3S, 8S, 9S, 10 R, 13R, 14S, 17R)-10, 13-dimethyl-17-[(2R, 5S)-5-propan-2-yloctan-2-yl]-2, 3, 4, 7, 8, 9, 11, 12, 14, 15, 16, 17-dodecahydro-1H-cyclopenta[a]phenanthren-3-ol (MOL000033), (+)-catechin (MOL000492), beta-sitosterol (MOL000358), and petunidin (MOL000490) coincided in two different strategies (Figure 1; Table S2).

### 3.2. Locked Target Genes and VMC-Associated Symbols

Using SwissTargetPrediction, we predicted from the abovementioned components' corresponding targets that 408, 325, 505, 46, 326, and 468 targets had the opportunity to be captured individually in HQ, YGZ, KS, JH, CH, and JXT (Figure S1; Tables S3 and S4). A 100% probability was presented between mairin (MOL000211), isorhamnetin (MOL000354), formononetin (MOL000392), kaempferol (MOL000422), quercetin (MOL000098), ellagic acid (MOL001002), digallate (MOL000569), luteolin (MOL000006), (-)-epigallocatechin-3-gallate (MOL006821), hyperforin (MOL003347), psi-baptigenin (MOL000507), and their respective 4 (e.g., SAE1 and POLB), 6 (e.g., XDH and CA2), 1 (IL2), 17 (e.g., NOX4 and AKR1B1), 67 (e.g., AVPR2 and MAOA), 44 (e.g., GPR35 and ERBB2), 2 (POLA1 and POLB), 34 (e.g., CDK5R1 and FLT3), 15 (e.g., MAPT and DNMT1), 1 (NR1I2), and 1 (PPARA) targets (Table S5). There were 984 VMC-related symbols annotated by GeneCards (Table S6). We mapped the potential targets to these symbols and found out 74, 67, 100, 12, 64, and 96 identical elements in HQ, YGZ, KS, JH, CH, and JXT in order (Figure S1; Table S7). Nine VMC-related symbols, SLC6A2, SLC6A4, NOS2, PPARA, PPARG, ACHE, CYP2C19, CYP51A1, and CHRM2, were highlighted and shared as common targets in the six single-herbal strategies.

### 3.3. Intercomparison of GO Terms and KEGG Pathways between Two Classes of Targets

**Figure 2:**
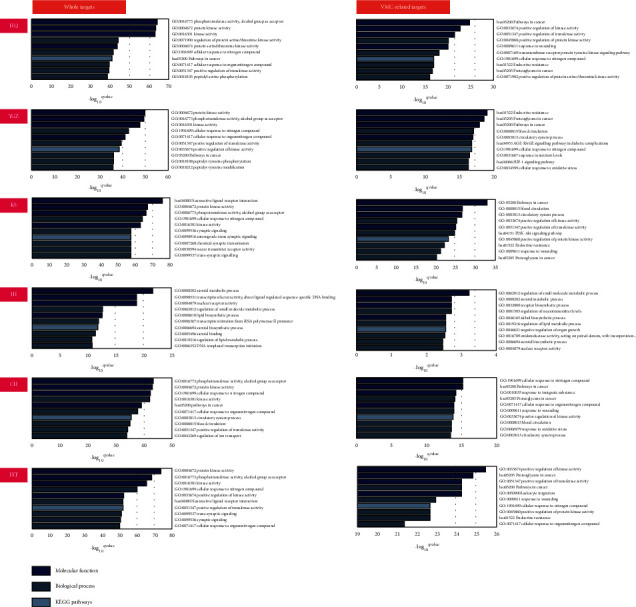
Top 10 elements enriched by whole targets or VMC-related targets in six herbal strategies.

The enrichment analysis was exerted to investigate herbal whole targets and VMC-related targets among them by GO and KEGG modules of Metascape. We detected significantly recruited (*p* < 0.05) 3514, 3290, 3979, 556, 3328, and 3811 terms and 393, 366, 418, 23, 370, and 416 pathways in all targets of HQ, YGZ, KS, JH, CH, and JXT in turn, as well as 1553, 1691, 1956, 117, 1540, and 2076 terms and 255, 253, 319, 0, 229, and 335 pathways in VMC-related targets of that. By analysing the top 10 (Figure 2; Table S8), we discovered that cellular response to the nitrogen compound (GO:1901699) was extensively recruited by targets of herbal strategies except all targets of JH and VMC-related targets of JH and KS, followed by positive regulation of transferase activity (GO:0051347) aimed by all targets of HQ, YGZ, CH, JXT, and VMC-related targets of HQ, KS, and JXT. The two overlapped GO terms containing response to wounding (GO:0009611) and positive regulation of protein kinase activity (GO:0045860) and two KEGG pathways involving proteoglycans in cancer (hsa05205) and endocrine resistance (hsa01522) only occurred in enriched VMC-related targets comparing with different herbal strategies, as well as phosphotransferase activity, an alcohol group as the acceptor (GO:0016773), protein kinase activity (GO:0004672), kinase activity (GO:0016301), trans-synaptic signaling (GO:0099537), synaptic signaling (GO:0099536), and neuroactive ligand-receptor interaction (hsa04080) in enriched all targets.

### 3.4. Interactive Correlation of Targets' Corresponding Proteins

MCODE algorithm was invoked to explore the PPI of herbal targets. The whole targets of HQ, YGZ, KS, JH, CH, and JXT were separately divided into 9, 12, 11, 2, 6, and 10 clusters, as well as VMC-related targets of that classified into 2, 1, 4, 0, 2, and 4 clusters, according to MCODE score (Figure S2; Table S9). SRC (DEGREE ≥ 21), EGFR (DEGREE ≥ 22), HSP90AA1 (DEGREE ≥ 25), AKT1 (DEGREE ≥ 11), MAPK1 (DEGREE ≥ 28), PRKCA (DEGREE ≥ 27), and PTK2 (DEGREE ≥ 16) with the core of PPI were targeted by more than two herbal strategies except JH, whereas APP (DEGREE ≥ 104) in HQ, YGZ, KS, CH, JXT, and HSP90AB1 (DEGREE ≥ 91) in HQ, YGZ, KS, and JXT merely were center members of whole targets (Table S9). JH displayed an individual sort of targeting, which might be attributable to fewer targets than other herbal strategies. CYP51A1, PPARG, NOS2, FDFT1, VDR, and CYP2C19 are among the few to be mapped as VMC-related targets with protein interaction.

### 3.5. Tissue- and Cell-specific Location of Herbal Targets

**Figure 3:**
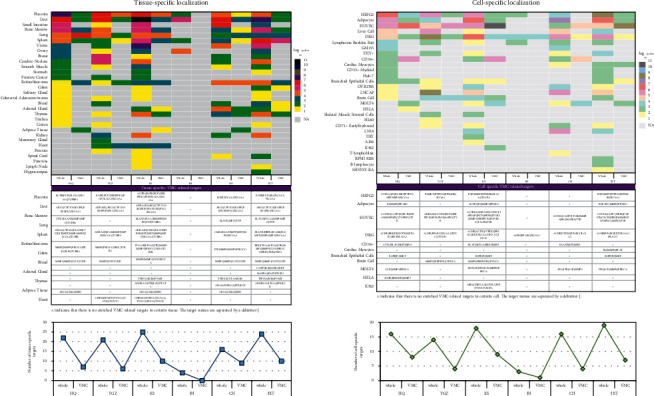
Tissue- and cell-specific localization of whole targets and VMC-related targets.

Through analysing the specific location of whole or VMC-related targets, in the 31 types of tissues and the 29 kinds of cells, we revealed that YGZ targeting whole AXL, CHRM2, FABP3, UTS2R, KCNA5, PDE3A, TNNC1, TNNI3, TNNT2, and TNKS and VMC-related CHRM2, FABP3, TNNC1, TNNI3, and TNNT2 were significantly (*p* < 0.01) located in heart, as significant (*p* < 0.001) as KS targeting whole AXL, CHRM2, CHRNA5, S1PR3, UTS2R, KCNA5, LNPEP, PDE3A, PLA2G5, SCN5A, TNNC1, TNNI3, TNNT2, TNKS, MAPKAPK2, and TNNI3K and VMC-related CHRM2, CHRNA5, SCN5A, TNNC1, TNNI3, and TNNT2 (Figure 3; Table S10). The cardiac-myocytes-specific location was significantly concentrated (*p* < 0.01) by HQ targeting whole AXL, MMP1, PLAT, RGS4, and PLK2, CH targeting whole AXL, F2R, and MMP1, and JXT targeting whole AXL, IL6, MMP1, PLAT, PLK2 and VMC-related IL6, MMP1, and PLAT (Figure 3).

### 3.6. Molecular Interaction Elicited by Herb Intervention

**Figure 4:**
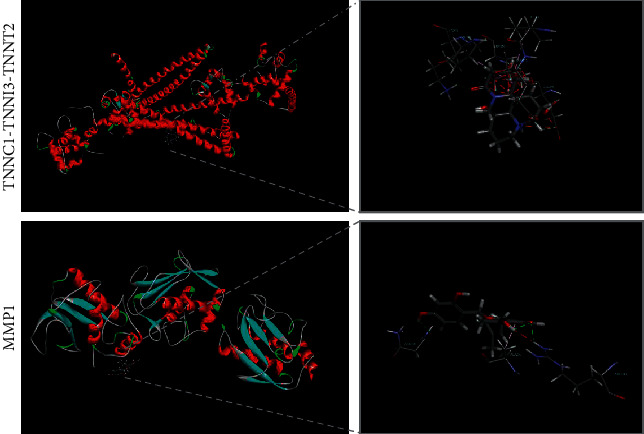
Amino acids on TNNC1-TNNI3-TNNT2 and MMP1 with the conventional hydrogen bond and hydrophobic interaction.

The targets including CHRM2, FABP3, TNNC1, TNNI3, TNNT2, CHRNA5, SCN5A, IL6, MMP1, and PLAT localized in the pathogenetic heart were selected to study the molecular interaction with herbal constituents by using digital PDB resource. Besides empty CHRNA5 information, the receptor-ligand interaction analyses of CHRM2 (PDB ID: 4mqs, 6oik), FABP3 (PDB ID: 3wxq, 5hz9), TNNC1-TNNI3-TNNT2 (PDB ID: 1j1e), SCN5A (PDB ID: 4dck, 6mud, 5dbr, 4jq0, 4ovn), IL6 (PDB ID: 5fuc, 4ni9), MMP1 (PDB ID: 2j0t, 3shi), PLAT (PDB ID: 1tpk, 5brr), and their binding components revealed reactive CHRM2 (PDB ID: 4mqs), FABP3 (PDB ID: 3wxq), TNNC1-TNNI3-TNNT2 (PDB ID: 1j1e), and MMP1 (PDB ID: 3shi) with respective 12, 3, 4, and 9 components (Table S11). The types of interactions consisted of alkyl, *π*-alkyl, carbon-hydrogen bond, *π*-anion, *π*-cation, amide-*π* stacked, van der Waals, attractive charge, conventional hydrogen bond, and *π*-lone pair, along with nonclassical hydrogen bonds occurred mainly on components communicating with CHRM2, FABP3, and TNNC1-TNNI3-TNNT2 (Figure 4; Figures S3 and S4).

## 4. Discussion

Previous research has reported that 10-mL HQ oral liquid daily significantly decreases sinus tachycardia, frequent premature ventricular contractions, and supraventricular tachycardia and improves myocardial enzymes and cardiac function indexes compared to placebo daily in 68 VMC children [57]. With intervention of the HQ oral liquid, the VMC children also show high levels of retinoic acid receptor-related orphan nuclear receptor gamma, forkhead transcription factor, interleukin-11, and transforming growth factor beta, as well as low levels of interleukin-17A, interleukin-21, creatine kinase-MB, cardiac troponin I, granzyme B, soluble fas ligand, and caspase-3 [57]. YGZ extract is linked to reduction of cardiac CVB3 titers, inhibition of CVB3-related apoptosis effects, and suppression of pathological damages of cardiac muscle in myocarditic mice [55]. Sophoridine is an alkaloid isolated from Chinese medicinal herb KS. The serum samples acquired from rats with oral sophoridine diminish the virus titers in infected myocardial cells, while sophoridine clearly decreases tumor necrosis factor mRNA expression and increases mRNA expression of interferon gamma and interleukin-10 [56]. Positive outcomes such as enhanced survival rate, improved weight loss, and heart histopathology are driven by JH's active component which alleviates the systemic and local myocardial expression of proinflammatory cytokines such as interleukin-6, interleukin-1*β*, and tumor necrosis factor in the CVB3-infected mice [53]. CH protects cells against virus infection and has a palpable inhibitory effect on CVB3m replication in the therapeutic cell group [54]. Aqueous extract of JXT markedly dampens the mRNA expression of CVB3 and severally reduces 15-day mortality to forty percent and forty-five percent and 30-day mortality to forty-five percent and fifty percent at doses of 50 mg/kg and 100 mg/kg in mice [52]. Hence, the six single-herbal strategies including HQ, YGZ, KS, JH, CH, and JXT were selected as responsible herbs against VMC to investigate.

Using the TCMSP, PubChem, and SwissTargetPrediction, we screened out 79 components and their 786 potential targets by duplication removing from six single-herbal strategies. The whole 786 targets ranged over 150 VMC-associated symbols. Our priority was to focus on analysing nine common VMC-associated targets including SLC6A2, NOS2, SLC6A4, PPARA, ACHE, CYP2C19, PPARG, CYP51A1, and CHRM2 in six herbal strategies. Sodium-dependent noradrenaline transporter targeted by 9 herbal components is encoded by *SLC6A2* and responsible for presynaptic noradrenaline reuptake. Between the vasculature, heart, and kidney, it plays an essential role in the distribution of sympathetic activity. Genetic *SLC6A2* dysfunction is capable of triggering the postural tachycardia syndrome while the impaired function of cardiac *SLC6A2* is familiar in a variety of organic heart disease such as ischemic heart disease, congestive heart failure, and stress-induced cardiomyopathy [58]. *NOS2* encodes inducible nitric oxide synthase. Myocardial infiltrating macrophages express high levels of inducible nitric oxide synthase in CVB3-infected male mice [59]. The higher circulatory and local concentrations of mRNA and protein of *NOS2* contribute to lower viral stocks [60]. Lack of *NOS2* results in a sudden rise in the mortality of mice with Coxsackievirus infection [61]. But notably in CVB3-infected mice, the intensifying of cardiac *NOS2* expression exaggerates myocardial damage [62]. Sodium-dependent serotonin transporter encoded by *SLC6A4* is active in heart valve development, and its deficiency is conjoined with apparent perivascular, interstitial, and valvular fibrosis [63]. Peroxisome-proliferator-activated receptors include alpha, beta, and gamma subtypes [64]. *PPARA* encodes peroxisome-proliferator-activated receptor alpha whose activation improves experimental autoimmune myocarditis through restraining Th17 cell differentiation under expression inhibition of retinoic acid receptor-related orphan nuclear receptor gamma and phosphorylated signal transducer and activator of transcription 3 *in vivo* [65]. *PPARG* encodes peroxisome-proliferator-activated receptor gamma. A small heterodimer partner expressed in the heart can attenuate the hypertrophic response, while changes in inflammation and metabolism are correlated with marked alterations in the mRNA levels of *PPARA* and *PPARG* in small heterodimer partner overexpressing cells [66]. There is evidence that treatment with the ligand (WY14643) of peroxisome-proliferator-activated receptor alpha facilitates the expression of anti-inflammatory cytokine interleukin-10 mRNA in rats [67]. The peroxisome-proliferator-activated receptor beta agonist (GW501516) and the peroxisome-proliferator-activated receptor gamma agonist (rosiglitazone) elicit the interleukin-10 release [68]. Besides upregulating M2 polarization-related factor interleukin-10, the use of peroxisome-proliferator-activated receptor gamma agonists also can downregulate macrophage M1 polarization-related factors such as interleukin-1 and interleukin-6 [69]. In terms of HQ and KS, it has been reported that the Huangqi glycoprotein and Fufang Kushen Injection Liquid contribute to increasing the level of interleukin-10 [70, 71]. The upregulated gene *CYP2C19* and frequent expression of the corresponding protein cytochrome P450 2C19 have been considered as a protective compensation reaction in chronic Keshan disease, an endemic cardiomyopathy [72]. *CYP51A1* encodes lanosterol 14-alpha demethylase. The *CYP51A1* deficiency in mice shows heart failure and lethality owing to heart hypoplasia, vasculogenesis, ventricle septum, and epicardial defects [73]. Acetylcholinesterase encoded by *ACHE* is involved in regulating levels of acetylcholine which is an anti-inflammatory molecule connected to inflammatory response [74]. *CHRM2* encodes muscarinic acetylcholine receptor M2 such that the missense mutation (C722 G) identified in the *CHRM2* triggers heart failure, arrhythmia, and sudden death in the patients with dilated cardiomyopathy [75]. In light of these characteristics, it is plausible that the six herbal strategies possess antiviral and anti-inflammatory effect, maintain the healthy development of the heart, and prevent heart failure by targeting and regulating SLC6A2, NOS2, SLC6A4, PPARA, PPARG, CYP2C19, CYP51A1, ACHE, and CHRM2.

What follows is machine learning of prospective targets that refers to functional enrichment, protein interaction, and specific location analyses comparing VMC-associated targets to whole targets in different herbal strategies. In terms of numbers of the abovementioned elements enriched by VMC-associated targets, more than 3000 GO terms and 300 KEGG pathways were recruited by the whole targets of herbal strategies without JH. Our findings demonstrated that cellular response to the nitrogen compound (GO:1901699) and positive regulation of transferase activity (GO:0051347) preferred to be significantly enriched by whole and VMC-associated targets. There is a report that nitric oxide disables the coxsackieviral protease 2A by active-cysteine S-nitrosylation *in vitro* and in living COS-7 cells and may be defensive in human heart failure [76]. Histone acetyl transferases are able to induce and antagonize hypertrophic growth [77]. Response to wounding (GO:0009611), blood circulation (GO:0008015), the circulatory system process (GO:0003013), and positive regulation of protein kinase activity (GO:0045860) were obviously recruited by VMC-associated targets. Macrophages as innate immune cells stimulate the immune response and wound healing, in which M2 macrophages cover anywhere from thirty to seventy percent of the infiltrate during acute viral myocarditis [78]. Moreover, the elevated M2 macrophage polarization is closely relevant to the inhibition of inflammation and conducive to alleviating VMC [79]. Adoptive transfer of M2 macrophages lowers cardiac inflammation [80], while accelerating M2 polarization of macrophages ameliorates cardiac damage following VMC in mice [81]. With viral infection, acute perimyocarditis leads to haemodynamic instability [82]. The P38 mitogen-activated protein kinase (MAPK) pathway plays an important role in CVB3-induced myocarditis. Experiments in a mouse model have verified that miRNA aiming the MAP2K3/P38 MAPK signaling appreciably decreases viral titers, attenuates the rate of cell apoptosis, and lengthens the living time against CVB3 infection [83]. The invaluable evidence has shown that HQ, KS, and CH are involved in repressing expression, phosphorylation, and activation of p38 MAPK in turn [84–86]. This part of results highlighted the fact that the herbal targets are intensively relevant to the development and response of VMC.

Besides single target, multiple targets are the tendency of new pharmaceutical development. We hope that, with the help of the PPI network, examines the role of single target or several targets on the balance of network and its perturbations. In the present work, we discovered that SRC and EGFR as PPI hubs have more than twenty partners possessing interactive potential both in whole and VMC-associated targets of HQ, YGZ, KS, CH, and JXT. *SRC* and *EGFR* separately encode proto-oncogene tyrosine-protein kinase Src and receptor protein-tyrosine kinase. Under coxsackieviral infection, the viral production in myocytes is reduced by SRC inhibition [87]. EGFR receptor activation contributes to the growth and survival of cardiomyocytes, while impotent EGFR signaling is linked in transition from compensatory hypertrophy to heart failure [88]. Compared to other strategies, JH's targets had certain individual features in the PPI network such that CYP51A1, CYP2C19, and PPARG were whole and VMC-associated targets, but NOS2, FDFT1, and VDR only occurred in VMC-associated targets. Moreover, their numbers of underlying interactive partners are rare (DEGREE < 10). In addition to *CYP51A1*, *CYP2C19*, *PPARG*, and *NOS2* noted earlier, *FDFT1* and *VDR* are responsible for encoding squalene synthase and vitamin D3 receptor, respectively. Ding *et al.* reported that changes in a network of coexpressed cholesterol metabolism genes encompassing sterol synthesis gene *FDFT1* are a characteristic mark of inflammatory stress [89]. VDR is supposed to participate in the inflammatory-immune process in VMC pathogenesis for the reason that the VDR expression is significantly increased after CVB3 injection in the mice myocardium [90]. Interference on these PPI hubs possibly will disturb the VMC system in the greatest degree.

The next detail is that specific targets were detected in 31 kinds of tissues and 29 types of cells. Taking significant enrichment as the screening standard, the categories of specific tissues focused by whole targets generally exceed that covered by VMC-associated targets in number. As shown in Figure 3, CHRM2, FABP3, TNNC1, TNNI3, and TNNT2 aimed by YGZ and CHRM2, CHRNA5, SCN5A, TNNC1, TNNI3, and TNNT2 directed by KS were localized in the heart, as well as JXT targeting IL6, MMP1, and PLAT localized in cardiac myocytes. Aside from the mentioned CHRM2, in heart tissue, fatty acid binding protein 3 (encoded by *FABP3*) deficiency alleviates myocardial apoptosis and cardiac remodeling, forming a protection from ischemic heart injuries [91]. *TNNC1* encodes slow skeletal and cardiac-type troponin C1, and its mutations play an essential role in the development of cardiomyopathy, in which the TNNC1-A8V mutant evokes diastolic disorder through raising the calcium-ion-binding affinity of the thin filament and altering calcium ion homeostasis and cellular remodeling [92]. Cardiac-type troponin I3 and sodium channel protein type 5 subunit alpha are severally encoded by *TNNI3* and *SCN5A*. Seven of 42 patients with acute myocarditis carry infrequent biallelic nonsynonymous or splice-site variations in cardiomyopathy-related *TNNI3* or *SCN5A* [93]. As a cardio-specific differentiation factor, cardiac-type troponin T2 encoded by *TNNT2* elevates the cardiomyogenic efficiency of cardiosphere-derived cells to form large cardiomyocytes populations [94]. *CHRNA5* encodes neuronal acetylcholine receptor subunit alpha-5. The secretion of proinflammatory cytokine interleukin-1*β* is significantly decreased by fifty percent in bone-marrow-derived macrophages by comparing *CHRNA5* knockout mice with wild-type controls [95]. In cardiac myocytes, CVB3 internalization triggers increased cell survival and the secretion of interleukin-6 (encoded by *IL6*) whose levels were reduced after receiving antiviral therapy [96, 97]. Astragaloside treatment downregulates interstitial collagenase (encoded by *MMP1*) expression and attenuates the myocardial fibrosis and reduces the mortality in mice with chronic myocarditis [98]. Polymorphisms in tissue-type plasminogen activator encoded by *PLAT* are implicated in strokes and myocardial infarctions and susceptible to bacterial osteomyelitis [99]. A prior report has validated that CVB3 infection results in the production of autoreactive T cells for multiantigens, implying that the autoreactive T cells localized in the liver probably circulate and promote viral myocarditis development [100]. This could suggest that the other VMC-associated targets nonlocalizing heart tissue, with the presence of herb intervention, equally participate in the regulation of the VMC process or myocardial lesion, except CHRM2, FABP3, TNNC1, TNNI3, TNNT2, CHRNA5, SCN5A, IL6, MMP1, and PLAT.

Intermolecular interactions dominate various important physical and chemical properties of herbal components. Correlated with 12, 3, 4, 9 components, and their respective target CHRM2 (PDB ID: 4mqs), FABP3 (PDB ID: 3wxq), TNNC1-TNNI3-TNNT2 (PDB ID: 1j1e), and MMP1 (PDB ID: 3shi), we found that, on human cardiac troponin (TNNC1-TNNI3-TNNT2), amino acid ARG111 showed a conventional hydrogen bond with ellagic acid (MOL001002 index 6), and LYS253, ILE114, and VAL118 individually acted as an interactive anchor of the conventional hydrogen bond and hydrophobic interaction with 5*α*, 9*α*-dihydroxymatrine (MOL006582 index 1), as well as ARG208, ASN106 coupled to conventional hydrogen bond, and ALA258 connected to hydrophobic interaction on MMP1 with leachianone g (MOL006626 index 2). A recent study proved that hydrophobic groups and hydrogen bond acceptors may work in the inhibitory potency of flavonoids existed in herbal products on breast cancer resistance protein [101]. The interactions of the high-affinity conventional hydrogen bond in *Trypanosoma brucei* pteridine reductase 1 or *Leishmania* major pteridine reductase 1 with chroman-4-one moiety expose their relevance on the compound activity and could be one of the causes of inhibitory effects of chroman-4-one moiety to the two reductases [102]. The binding affinity of FKBP22 of a psychrophilic bacterium, *Shewanella* sp. SIB1, to the native or reduced states of insulin is mainly facilitated by hydrophobic interaction [103]. Therefore, the ARG111, LYS253, ILE114, and VAL11 on cardiac troponin and the ARG208, ASN106, and ALA258 on MMP1 are possible to elucidate the binding potential of the herbal component and corresponding target against VMC.

## 5. Conclusions

In the present work, we collected six single-herbal strategies against VMC and screened out active components and their corresponding targets. Enrichment analysis underlined centric targets fixed in the PPI network and specific targets localized in heart, following annotation of VMC-related symbols. Besides that, a receptor-ligand interaction model clarified the underlying categories of intermolecular interactions and efficient amino acids based on herbal components and targets in the location of heart lesions. These findings may contribute to the development of new treatments and targeted drugs against VMC in the future.

## Data Availability

All data generated or analysed during this study are included in this article.

## Abbreviations

CH: Chaihu
CVB3: Coxsackievirus B3
GO: Gene Ontology
HQ: Huangqi
KEGG: Kyoto Encyclopedia of Genes and Genomes
JH: Jianghuang
JXT: Jixueteng
KS: Kushen
MAPK: Mitogen-activated protein kinase
PDB: Protein Data Bank
PPI: Protein-protein interaction
TCM: Traditional Chinese medicine
TCMSP: Traditional Chinese Medicine Systems Pharmacology Database and Analysis Platform
VMC: Viral myocarditis
YGZ: Yuganzi

## Supplementary Materials

Supplementary Materials Figure S1. Herbal components' corresponding whole and VMC-related targets. Figure S2. Interaction network of targets' corresponding proteins. Figure S3. Amino acids on TNNC1-TNNI3-TNNT2 and MMP1 with different interactions. Figure S4. Prospective intermolecular interactions binding herbal components to CHRM2, FABP3, TNNC1-TNNI3-TNNT2, and MMP1. Table S1. Herbs and their components. Table S2. The common and unique components of different herbal strategies. Table S3. Component ID and targets. Table S4. Herbal targets. Table S5. A 100% binding possibility between the component and target. Table S6. VMC-related symbols identified in GeneCards. Table S7. The common and unique VMC-related targets of different herbal strategies. Table S8. TOP 10 elements significantly enriched by whole target and VMC-related targets. Table S9. Target details in the PPI network. Table S10. Tissue- and cell-specific location of targets. Table S11. Potential match relation between the component and target.
