# Supplementary material for: Comparison and Analysis on the Existing Single-Herbal Strategies against Viral Myocarditis
Source: Genet Res (Camb). 2021 Aug 7;2021:9952620. doi: 10.1155/2021/9952620 (PMC8371739; doi:10.1155/2021/9952620)
Supplement: Supplementary Materials — Figure S1. Herbal components' corresponding whole and VMC-related targets. Figure S2. Interaction network of targets' corresponding proteins. Figure S3. Amino acids on TNNC1-TNNI3-TNNT2 and MMP1 with different interactions. Figure S4. Prospective intermolecular interactions binding herbal components to CHRM2, FABP3, TNNC1-TNNI3-TNNT2, and MMP1. Table S1. Herbs and their components. Table S2. The common and unique components of different herbal strategies. Table S3. Component ID and targets. Table S4. Herbal targets. Table S5. A 100% binding possibility between the component and target. Table S6. VMC-related symbols identified in GeneCards. Table S7. The common and unique VMC-related targets of different herbal strategies. Table S8. TOP 10 elements significantly enriched by whole target and VMC-related targets. Table S9. Target details in the PPI network. Table S10. Tissue- and cell-specific location of targets. Table S11. Potential match relation between the component and target. [file 9952620.f1.docx]

**Fig. S1.** Herbal components corresponding whole and VMC-related targets.

**Fig. S2.** Interaction network of targets corresponding proteins.

**Fig. S3.** Amino acids on TNNC1-TNNI3-TNNT2 and MMP1 with different interactions.

**Fig. S4.** Prospective intermolecular interactions binding herbal components to CHRM2, FABP3, TNNC1-TNNI3-TNNT2, MMP1.

| **Table S1. Herbs and their components** | | | | | | | | | | | | | | |
| --- | --- | --- | --- | --- | --- | --- | --- | --- | --- | --- | --- | --- | --- | --- |
| **Herb Name** | **Mol ID** | **Molecule Name** | **InChIKey** | **Pubchem Cid** | **MW** | **AlogP** | **Hdon** | **Hacc** | **OB (%)** | **Caco-2** | **BBB** | **DL** | **FASA-** | **HL** |
| Huangqi | MOL000211 | Mairin | QGJZLNKBHJESQX-FZFNOLFKSA-N | 64971 | 456.78 | 6.52 | 2 | 3 | 55.38 | 0.73 | 0.22 | 0.78 | 0.26 | 8.87 |
|  | MOL000239 | Jaranol | BJBUTJQYZDYRMJ-UHFFFAOYSA-N | 5318869 | 314.31 | 2.09 | 2 | 6 | 50.83 | 0.61 | -0.22 | 0.29 | 0.29 | 15.5 |
|  | MOL000033 | (3S,8S,9S,10R,13R,14S,17R)-10,13-dimethyl-17-[(2R,5S)-5-propan-2-yloctan-2-yl]-2,3,4,7,8,9,11,12,14,15,16,17-dodecahydro-1H-cyclopenta[a]phenanthren-3-ol | KLEXDBGYSOIREE-UIFQYPGESA-N | 15976101 | 428.82 | 8.54 | 1 | 1 | 36.23 | 1.45 | 1.09 | 0.78 | 0 | 5.22 |
|  | MOL000354 | isorhamnetin | IZQSVPBOUDKVDZ-UHFFFAOYSA-N | 5281654 | 316.28 | 1.76 | 4 | 7 | 49.6 | 0.31 | -0.54 | 0.31 | 0.32 | 14.34 |
|  | MOL000371 | 3,9-di-O-methylnissolin | RFFNFQZKHNKOPO-BBRMVZONSA-N | 15689655 | 314.36 | 2.89 | 0 | 5 | 53.74 | 1.18 | 0.63 | 0.48 | 0 | 9 |
|  | MOL000378 | 7-O-methylisomucronulatol | BLHQCBJSTMDZQA-LBPRGKRZSA-N | 15689652 | 316.38 | 3.38 | 1 | 5 | 74.69 | 1.08 | 0.84 | 0.3 | 0 | 2.98 |
|  | MOL000379 | 9,10-dimethoxypterocarpan-3-O-β-D-glucoside | PCIXSTFFMHVOMF-PBGSHFJYSA-N | 101679160 | 462.49 | 0.74 | 4 | 10 | 36.74 | -0.63 | -1.5 | 0.92 | 0 | 13.06 |
|  | MOL000380 | (6aR,11aR)-9,10-dimethoxy-6a,11a-dihydro-6H-benzofurano[3,2-c]chromen-3-ol | UOVGCLXUTLXAEC-WFASDCNBSA-N | 14077830 | 300.33 | 2.64 | 1 | 5 | 64.26 | 0.93 | 0.55 | 0.42 | 0 | 8.49 |
|  | MOL000387 | Bifendate | JMZOMFYRADAWOG-UHFFFAOYSA-N | 108213 | 418.38 | 2.56 | 0 | 10 | 31.1 | 0.15 | -0.06 | 0.67 | 0 | 17.96 |
|  | MOL000392 | formononetin | HKQYGTCOTHHOMP-UHFFFAOYSA-N | 5280378 | 268.28 | 2.58 | 1 | 4 | 69.67 | 0.78 | 0.02 | 0.21 | 0 | 17.04 |
|  | MOL000417 | Calycosin | ZZAJQOPSWWVMBI-UHFFFAOYSA-N | 5280448 | 284.28 | 2.32 | 2 | 5 | 47.75 | 0.52 | -0.43 | 0.24 | 0 | 17.1 |
|  | MOL000422 | kaempferol | IYRMWMYZSQPJKC-UHFFFAOYSA-N | 5280863 | 286.25 | 1.77 | 4 | 6 | 41.88 | 0.26 | -0.55 | 0.24 | 0 | 14.74 |
|  | MOL000433 | FA | OVBPIULPVIDEAO-LBPRGKRZSA-N | 6037 | 441.45 | 0.01 | 7 | 13 | 68.96 | -1.5 | -2.59 | 0.71 | 0 | 24.81 |
|  | MOL000438 | (3R)-3-(2-hydroxy-3,4-dimethoxyphenyl)chroman-7-ol | NQRBAPDEZYMKFL-NSHDSACASA-N | 10380176 | 302.35 | 3.13 | 2 | 5 | 67.67 | 0.96 | 0.34 | 0.26 | 0 | 2.9 |
|  | MOL000439 | isomucronulatol-7,2'-di-O-glucosiole | NHOPAJCVMDIGBN-MEPKZADGSA-N | 15689653 | 626.67 | -0.68 | 8 | 15 | 49.28 | -2.22 | -3.36 | 0.62 | 0 | 0.93 |
|  | MOL000442 | 1,7-Dihydroxy-3,9-dimethoxy pterocarpene | RVGZSUMTFIEORY-UHFFFAOYSA-N | 5316760 | 314.31 | 3.11 | 2 | 6 | 39.05 | 0.89 | -0.04 | 0.48 | 0 | 7.95 |
|  | MOL000098 | quercetin | REFJWTPEDVJJIY-UHFFFAOYSA-N | 5280343 | 302.25 | 1.5 | 5 | 7 | 46.43 | 0.05 | -0.77 | 0.28 | 0.38 | 14.4 |
| Yuganzi | MOL001002 | ellagic acid | AFSDNFLWKVMVRB-UHFFFAOYSA-N | 5281855 | 302.2 | 1.48 | 4 | 8 | 43.06 | -0.44 | -1.41 | 0.43 | 0.43 | -1.04 |
|  | MOL000358 | beta-sitosterol | KZJWDPNRJALLNS-VJSFXXLFSA-N | 222284 | 414.79 | 8.08 | 1 | 1 | 36.91 | 1.32 | 0.99 | 0.75 | 0.23 | 5.36 |
|  | MOL000422 | kaempferol | IYRMWMYZSQPJKC-UHFFFAOYSA-N | 5280863 | 286.25 | 1.77 | 4 | 6 | 41.88 | 0.26 | -0.55 | 0.24 | 0 | 14.74 |
|  | MOL000492 | (+)-catechin | PFTAWBLQPZVEMU-DZGCQCFKSA-N | 9064 | 290.29 | 1.92 | 5 | 6 | 54.83 | -0.03 | -0.73 | 0.24 | 0 | 0.61 |
|  | MOL000569 | digallate | COVFEVWNJUOYRL-UHFFFAOYSA-N | 341 | 322.24 | 1.53 | 6 | 9 | 61.85 | -0.76 | -1.52 | 0.26 | 0.43 | 5.29 |
|  | MOL000006 | luteolin | IQPNAANSBPBGFQ-UHFFFAOYSA-N | 5280445 | 286.25 | 2.07 | 4 | 6 | 36.16 | 0.19 | -0.84 | 0.25 | 0.39 | 15.94 |
|  | MOL006793 | mucic acid 1,4-lactone 2-0-gallate | PRTMDWTUELEYNR-UKKRHICBSA-N | 87515565 | 358.28 | -0.19 | 6 | 11 | 49.56 | -1.86 | -2.22 | 0.31 | 0.44 | 4.68 |
|  | MOL006796 | mucic acid 1,4-lactone 5-0-gallate | SVYWZVZMBHFNGC-KYXWUPHJSA-N | 87219915 | 344.25 | -0.67 | 6 | 11 | 52.26 | -1.73 | -2.29 | 0.27 | 0.42 | 7.01 |
|  | MOL006799 | (2S,3R,3aS,4R,4'S,5'R,6S,7aR)-3,4,4'-trihydroxy-3,5'-bis(hydroxymethyl)spiro[3a,4,5,6,7,7a-hexahydrobenzofuran-2,2'-tetrahydropyran]-6-carboxylic acid | YCATYHRROQAZDN-SFLVWTQSSA-N | 11057167 | 348.39 | -2.55 | 6 | 9 | 48.46 | -1.92 | -2.64 | 0.31 | 0.25 | 4.98 |
|  | MOL006812 | Phyllanthin | KFLQGJQSLUYUBF-WOJBJXKFSA-N | 358901 | 418.58 | 4.11 | 0 | 6 | 33.31 | 1.06 | 0.57 | 0.42 | 0.15 | 3.36 |
|  | MOL006821 | (-)-epigallocatechin-3-gallate | WMBWREPUVVBILR-WIYYLYMNSA-N | 65064 | 458.4 | 2.89 | 8 | 11 | 55.09 | -0.57 | -1.7 | 0.77 | 0.37 | 1.7 |
|  | MOL006824 | α-amyrin | FSLPMRQHCOLESF-ACUMZOKISA-N | 12358389 | 426.8 | 7.35 | 1 | 1 | 39.51 | 1.37 | 1.2 | 0.76 | 0.23 | 3.06 |
|  | MOL006826 | chebulic acid | COZMWVAACFYLBI-XJEVXTIOSA-N | 71308174 | 356.26 | -0.26 | 6 | 11 | 72 | -1.4 | -1.75 | 0.32 | 0.4 | 3.44 |
|  | MOL000098 | quercetin | REFJWTPEDVJJIY-UHFFFAOYSA-N | 5280343 | 302.25 | 1.5 | 5 | 7 | 46.43 | 0.05 | -0.77 | 0.28 | 0.38 | 14.4 |
| Kushen | MOL001040 | (2R)-5,7-dihydroxy-2-(4-hydroxyphenyl)chroman-4-one | FTVWIRXFELQLPI-CYBMUJFWSA-N | 667495 | 272.27 | 2.3 | 3 | 5 | 42.36 | 0.38 | -0.48 | 0.21 | 0.41 | 16.83 |
|  | MOL001484 | Inermine | HUKSJTUUSUGIDC-ZBEGNZNMSA-N | 91510 | 284.28 | 2.44 | 1 | 5 | 75.18 | 0.89 | 0.4 | 0.54 | 0.3 | 11.72 |
|  | MOL003542 | 8-Isopentenyl-kaempferol | NADCVNHITZNGJU-UHFFFAOYSA-N | 5318624 | 354.38 | 3.63 | 4 | 6 | 38.04 | 0.53 | -0.49 | 0.39 | 0 | 15.37 |
|  | MOL003627 | sophocarpine | AAGFPTSOPGCENQ-JLNYLFASSA-N | 115269 | 246.39 | 1.39 | 0 | 3 | 64.26 | 0.99 | 1 | 0.25 | 0.26 | 5.54 |
|  | MOL003648 | Inermin | HUKSJTUUSUGIDC-BDJLRTHQSA-N | 161298 | 284.28 | 2.44 | 1 | 5 | 65.83 | 0.91 | 0.36 | 0.54 | 0.3 | 11.73 |
|  | MOL003673 | Wighteone | KIMDVVKVNNSHGZ-UHFFFAOYSA-N | 5281814 | 338.38 | 3.92 | 3 | 5 | 42.8 | 0.64 | -0.16 | 0.36 | 0.31 | 17.04 |
|  | MOL003680 | sophoridine | ZSBXGIUJOOQZMP-BHPKHCPMSA-N | 165549 | 248.41 | 1.42 | 0 | 3 | 60.07 | 1.13 | 1.14 | 0.25 | 0.18 | 5.57 |
|  | MOL000392 | formononetin | HKQYGTCOTHHOMP-UHFFFAOYSA-N | 5280378 | 268.28 | 2.58 | 1 | 4 | 69.67 | 0.78 | 0.02 | 0.21 | 0 | 17.04 |
|  | MOL004580 | cis-Dihydroquercetin | CXQWRCVTCMQVQX-GJZGRUSLSA-N | 443758 | 304.27 | 1.49 | 5 | 7 | 66.44 | -0.34 | -1.11 | 0.27 | 0.4 | 14.51 |
|  | MOL004941 | (2R)-7-hydroxy-2-(4-hydroxyphenyl)chroman-4-one | FURUXTVZLHCCNA-CQSZACIVSA-N | 928837 | 256.27 | 2.57 | 2 | 4 | 71.12 | 0.41 | -0.25 | 0.18 | 0 | 18.09 |
|  | MOL005100 | 5,7-dihydroxy-2-(3-hydroxy-4-methoxyphenyl)chroman-4-one | AIONOLUJZLIMTK-CQSZACIVSA-N | 676152 | 302.3 | 2.28 | 3 | 6 | 47.74 | 0.28 | -0.3 | 0.27 | 0.31 | 16.51 |
|  | MOL005944 | matrine | ZSBXGIUJOOQZMP-JLNYLFASSA-N | 91466 | 248.41 | 1.42 | 0 | 3 | 63.77 | 1.39 | 1.52 | 0.25 | 0 | 6.69 |
|  | MOL000006 | luteolin | IQPNAANSBPBGFQ-UHFFFAOYSA-N | 5280445 | 286.25 | 2.07 | 4 | 6 | 36.16 | 0.19 | -0.84 | 0.25 | 0.39 | 15.94 |
|  | MOL006561 | (+)-14alpha-hydroxymatrine | WZKRTWRYJWGESZ-MRTXSQPYSA-N | 15385683 | 264.41 | 0.74 | 1 | 4 | 35.73 | 0.53 | 0.48 | 0.29 | 0.21 | 3.01 |
|  | MOL006562 | (+)-7,11-dehydromatrine,(leontalbinine) | CDDHEMJXKBELBO-NHYWBVRUSA-N | 127039206 | 246.39 | 1.42 | 0 | 3 | 62.08 | 1.06 | 1.12 | 0.25 | 0.16 | 6.46 |
|  | MOL006563 | (+)-9alpha-hydroxymatrine | JTWPUVIWKODBID-WHPHWUKISA-N | 15385684 | 264.41 | 0.45 | 1 | 4 | 32.04 | 0.61 | 0.15 | 0.29 | 0.19 | 4.55 |
|  | MOL006564 | (+)-allomatrine | ZSBXGIUJOOQZMP-KYEXWDHISA-N | 7000681 | 248.41 | 1.42 | 0 | 3 | 58.87 | 1.08 | 1.13 | 0.25 | 0.18 | 5.49 |
|  | MOL006565 | AIDS211310 | ZSBXGIUJOOQZMP-QVHKTLOISA-N | 5271984 | 248.41 | 1.42 | 0 | 3 | 68.68 | 1.15 | 1.38 | 0.25 | 0.17 | 6.15 |
|  | MOL006568 | isosophocarpine | AAGFPTSOPGCENQ-RGCMKSIDSA-N | 5746484 | 246.39 | 1.39 | 0 | 3 | 61.57 | 1.39 | 1.45 | 0.25 | 0.24 | 5.9 |
|  | MOL006569 | (-)-14beta-hydroxymatrine | WZKRTWRYJWGESZ-ZSLBOAEBSA-N | 10659287 | 264.41 | 0.74 | 1 | 4 | 37.26 | 0.77 | 0.77 | 0.29 | 0.21 | 3.09 |
|  | MOL006570 | (-)-9alpha-hydroxysophoramine | HAPHBHKQJIPUEP-WHPHWUKISA-N | 12133310 | 262.39 | 0.43 | 1 | 4 | 35.23 | 0.38 | -0.12 | 0.29 | 0.25 | 4.28 |
|  | MOL006571 | anagyrine | FQEQMASDZFXSJI-JHJVBQTASA-N | 5351589 | 244.37 | 1.15 | 0 | 3 | 62.01 | 1.16 | 1.13 | 0.24 | 0 | 4.74 |
|  | MOL006573 | 13,14-dehydrosophoridine | AAGFPTSOPGCENQ-COMQUAJESA-N | 7605281 | 246.39 | 1.39 | 0 | 3 | 65.34 | 1.06 | 1.11 | 0.25 | 0.24 | 6.24 |
|  | MOL006582 | 5α,9α-dihydroxymatrine | BYQQDLUCCAZYJO-PGKPSXLWSA-N | 14274649 | 280.41 | -0.3 | 2 | 5 | 40.93 | 0.04 | -0.43 | 0.32 | 0.2 | 4.59 |
|  | MOL006596 | Glyceollin | YIFYYPKWOQSCRI-AZUAARDMSA-N | 162807 | 338.38 | 2.85 | 2 | 5 | 97.27 | 0.53 | -0.19 | 0.76 | 0.35 | 6.24 |
|  | MOL003347 | hyperforin | KGSZHKRKHXOAMG-HQKKAZOISA-N | 441298 | 536.87 | 8.62 | 1 | 4 | 44.03 | 0.87 | 0.4 | 0.6 | 0 | 2.15 |
|  | MOL006604 | (2S)-7-hydroxy-2-(4-hydroxyphenyl)-5-methoxy-8-(3-methylbut-2-enyl)chroman-4-one | YKGCBLWILMDSAV-SFHVURJKSA-N | 9928523 | 354.43 | 4.41 | 2 | 5 | 48.09 | 0.8 | 0 | 0.39 | 0.3 | 15.54 |
|  | MOL006613 | kushenin | NYGZYUAVZPIKBZ-BDJLRTHQSA-N | 154496847 | 286.3 | 2.39 | 2 | 5 | 47.62 | 0.71 | 0.35 | 0.38 | 0.27 | 8.86 |
|  | MOL006623 | kushenol,t | PFTQIVMILQKDQN-PEBXRYMYSA-N | 44563159 | 442.55 | 4.46 | 5 | 7 | 51.28 | -0.05 | -0.95 | 0.64 | 0.35 | 17.47 |
|  | MOL006626 | leachianone,g | VBOYLFNGTSLAAZ-GOSISDBHSA-N | 101711280 | 356.4 | 3.89 | 4 | 6 | 60.97 | 0.33 | -0.36 | 0.4 | 0.37 | 15.53 |
|  | MOL006627 | Lehmanine | WUVYENIUARJBNM-JLNYLFASSA-N | 3041752 | 246.39 | 1.11 | 0 | 3 | 62.23 | 1.18 | 1.25 | 0.25 | 0.21 | 6 |
|  | MOL006628 | (+)-Lupanine | JYIJIIVLEOETIQ-ZOBORPQBSA-N | 442956 | 248.41 | 1.42 | 0 | 3 | 52.71 | 1.16 | 1.19 | 0.24 | 0.18 | 4.25 |
|  | MOL006630 | Norartocarpetin | ZSYPIPFQOQGYHH-UHFFFAOYSA-N | 5481970 | 286.25 | 2.07 | 4 | 6 | 54.93 | 0.14 | -0.74 | 0.24 | 0.42 | 17.23 |
|  | MOL000456 | Phaseolin | LWTDZKXXJRRKDG-KXBFYZLASA-N | 91572 | 322.38 | 3.46 | 1 | 4 | 78.2 | 1.09 | 0.39 | 0.73 | 0.33 | 7.56 |
|  | MOL006650 | (-)-Maackiain-3-O-glucosyl-6'-O-malonate | ZHXRWFOBROFZAC-LVYGOKBNSA-N | 23724669 | 532.49 | 0.7 | 4 | 13 | 48.69 | -1.45 | -2.14 | 0.52 | 0.34 | 20.42 |
|  | MOL000098 | quercetin | REFJWTPEDVJJIY-UHFFFAOYSA-N | 5280343 | 302.25 | 1.5 | 5 | 7 | 46.43 | 0.05 | -0.77 | 0.28 | 0.38 | 14.4 |
| Jianghuang | MOL000449 | Stigmasterol | HCXVJBMSMIARIN-PHZDYDNGSA-N | 5280794 | 412.77 | 7.64 | 1 | 1 | 43.83 | 1.44 | 1 | 0.76 | 0.22 | 5.57 |
|  | MOL000953 | CLR | HVYWMOMLDIMFJA-DPAQBDIFSA-N | 5997 | 386.73 | 7.38 | 1 | 1 | 37.87 | 1.43 | 1.13 | 0.68 | 0.2 | 4.52 |
| Chaihu | MOL001645 | Linoleyl acetate | KFXARGMQYWECBV-ZDVGBALWSA-N | 5319042 | 308.56 | 6.85 | 0 | 2 | 42.1 | 1.36 | 1.08 | 0.2 | 0.21 | 7.48 |
|  | MOL002776 | Baicalin | IKIIZLYTISPENI-ZFORQUDYSA-N | 64982 | 446.39 | 0.64 | 6 | 11 | 40.12 | -0.85 | -1.74 | 0.75 | 0.36 | 17.36 |
|  | MOL000449 | Stigmasterol | HCXVJBMSMIARIN-PHZDYDNGSA-N | 5280794 | 412.77 | 7.64 | 1 | 1 | 43.83 | 1.44 | 1 | 0.76 | 0.22 | 5.57 |
|  | MOL000354 | isorhamnetin | IZQSVPBOUDKVDZ-UHFFFAOYSA-N | 5281654 | 316.28 | 1.76 | 4 | 7 | 49.6 | 0.31 | -0.54 | 0.31 | 0.32 | 14.34 |
|  | MOL000422 | kaempferol | IYRMWMYZSQPJKC-UHFFFAOYSA-N | 5280863 | 286.25 | 1.77 | 4 | 6 | 41.88 | 0.26 | -0.55 | 0.24 | 0 | 14.74 |
|  | MOL004598 | 3,5,6,7-tetramethoxy-2-(3,4,5-trimethoxyphenyl)chromone | SHRSLVWLFNSTLK-UHFFFAOYSA-N | 389001 | 432.46 | 2.54 | 0 | 9 | 31.97 | 0.75 | 0.08 | 0.59 | 0.13 | 15.54 |
|  | MOL004609 | Areapillin | IZWKTABKAJGBFW-UHFFFAOYSA-N | 158311 | 360.34 | 2.29 | 3 | 8 | 48.96 | 0.6 | -0.29 | 0.41 | 0.16 | 16.52 |
|  | MOL013187 | Cubebin | DIYWRNLYKJKHAM-MDOVXXIYSA-N | 117443 | 356.4 | 3.19 | 1 | 6 | 57.13 | 0.47 | -0.41 | 0.64 | 0.31 | 12.4 |
|  | MOL004624 | Longikaurin A | PSVHVXLCVSKJGM-MHRDNBEJSA-N | 102117144 | 348.48 | 1.16 | 3 | 5 | 47.72 | 0.08 | 0.09 | 0.53 | 0.27 | 1.71 |
|  | MOL004644 | Sainfuran | BVSPXSLCUKWRNP-UHFFFAOYSA-N | 185034 | 286.3 | 3.38 | 2 | 5 | 79.91 | 0.9 | 0.23 | 0.23 | 0.22 | 8.58 |
|  | MOL004653 | (+)-Anomalin | PNTWXEIQXBRCPS-JLTIQLCOSA-N | 5319252 | 426.5 | 5.05 | 0 | 7 | 46.06 | 0.46 | 0 | 0.66 | 0.36 | 1.03 |
|  | MOL000490 | petunidin | BLBZAMLPGFAHFX-UHFFFAOYSA-N | 131834464 | 317.29 | 1.65 | 5 | 7 | 30.05 | 0.16 | -0.64 | 0.31 | 0 | 1.21 |
|  | MOL000098 | quercetin | REFJWTPEDVJJIY-UHFFFAOYSA-N | 5280343 | 302.25 | 1.5 | 5 | 7 | 46.43 | 0.05 | -0.77 | 0.28 | 0.38 | 14.4 |
| Jixueteng | MOL000033 | (3S,8S,9S,10R,13R,14S,17R)-10,13-dimethyl-17-[(2R,5S)-5-propan-2-yloctan-2-yl]-2,3,4,7,8,9,11,12,14,15,16,17-dodecahydro-1H-cyclopenta[a]phenanthren-3-ol | KLEXDBGYSOIREE-UIFQYPGESA-N | 15976101 | 428.82 | 8.54 | 1 | 1 | 36.23 | 1.45 | 1.09 | 0.78 | 0 | 5.22 |
|  | MOL000358 | beta-sitosterol | KZJWDPNRJALLNS-VJSFXXLFSA-N | 222284 | 414.79 | 8.08 | 1 | 1 | 36.91 | 1.32 | 0.99 | 0.75 | 0.23 | 5.36 |
|  | MOL000392 | formononetin | HKQYGTCOTHHOMP-UHFFFAOYSA-N | 5280378 | 268.28 | 2.58 | 1 | 4 | 69.67 | 0.78 | 0.02 | 0.21 | 0 | 17.04 |
|  | MOL000417 | Calycosin | ZZAJQOPSWWVMBI-UHFFFAOYSA-N | 5280448 | 284.28 | 2.32 | 2 | 5 | 47.75 | 0.52 | -0.43 | 0.24 | 0 | 17.1 |
|  | MOL000449 | Stigmasterol | HCXVJBMSMIARIN-PHZDYDNGSA-N | 5280794 | 412.77 | 7.64 | 1 | 1 | 43.83 | 1.44 | 1 | 0.76 | 0.22 | 5.57 |
|  | MOL000468 | 8-o-Methylreyusi | SELGEUSJRWRBQR-UHFFFAOYSA-N | 5319771 | 298.31 | 2.57 | 1 | 5 | 70.32 | 0.86 | 0.02 | 0.27 | 0 | 15.83 |
|  | MOL000469 | 3-Hydroxystigmast-5-en-7-one | ICFXJOAKQGDRCT-ZIHMWMKCSA-N | 160608 | 428.77 | 7.15 | 1 | 2 | 40.93 | 1.01 | 0.45 | 0.78 | 0 | 4.89 |
|  | MOL000471 | aloe-emodin | YDQWDHRMZQUTBA-UHFFFAOYSA-N | 10207 | 270.25 | 1.67 | 3 | 5 | 83.38 | -0.12 | -1.07 | 0.24 | 0 | 31.49 |
|  | MOL000483 | (Z)-3-(4-hydroxy-3-methoxy-phenyl)-N-[2-(4-hydroxyphenyl)ethyl]acrylamide | NPNNKDMSXVRADT-UITAMQMPSA-N | 6440659 | 313.38 | 2.86 | 3 | 5 | 118.35 | 0.51 | -0.27 | 0.26 | 0 | 4.26 |
|  | MOL000490 | petunidin | BLBZAMLPGFAHFX-UHFFFAOYSA-N | 131834464 | 317.29 | 1.65 | 5 | 7 | 30.05 | 0.16 | -0.64 | 0.31 | 0 | 1.21 |
|  | MOL000491 | Augelicin | PNTWXEIQXBRCPS-UWOGZXHISA-N | 25717254 | 426.5 | 5.05 | 0 | 7 | 37.5 | 0.66 | 0.3 | 0.66 | 0 | 2.93 |
|  | MOL000492 | (+)-catechin | PFTAWBLQPZVEMU-DZGCQCFKSA-N | 9064 | 290.29 | 1.92 | 5 | 6 | 54.83 | -0.03 | -0.73 | 0.24 | 0 | 0.61 |
|  | MOL000497 | licochalcone a | KAZSKMJFUPEHHW-DHZHZOJOSA-N | 5318998 | 338.43 | 4.62 | 2 | 4 | 40.79 | 0.82 | -0.21 | 0.29 | 0 | 16.2 |
|  | MOL000500 | Vestitol | XRVFNNUXNVWYTI-LLVKDONJSA-N | 177149 | 272.32 | 3.15 | 2 | 4 | 74.66 | 0.86 | 0.3 | 0.21 | 0 | 3 |
|  | MOL000502 | Cajinin | ALFNTRJPGFNJQV-UHFFFAOYSA-N | 5281706 | 300.28 | 2.05 | 3 | 6 | 68.8 | 0.48 | -0.36 | 0.27 | 0 | 14.23 |
|  | MOL000503 | Medicagol | URMVEUAWRUQHON-UHFFFAOYSA-N | 5319322 | 296.24 | 3.05 | 1 | 6 | 57.49 | 0.62 | -0.32 | 0.6 | 0 | 11.1 |
|  | MOL000506 | Lupinidine | SLRCCWJSBJZJBV-TUVASFSCSA-N | 7014 | 234.43 | 2.36 | 0 | 2 | 61.89 | 1.41 | 1.7 | 0.21 | 0 | 7.46 |
|  | MOL000507 | Psi-Baptigenin | KNJNBKINYHZUGC-UHFFFAOYSA-N | 5281805 | 282.26 | 2.37 | 1 | 5 | 70.12 | 0.57 | -0.27 | 0.31 | 0 | 21.95 |
|  | MOL000006 | luteolin | IQPNAANSBPBGFQ-UHFFFAOYSA-N | 5280445 | 286.25 | 2.07 | 4 | 6 | 36.16 | 0.19 | -0.84 | 0.25 | 0.39 | 15.94 |

| **Table S2. The common and unique components of different herbal strategies** | | |
| --- | --- | --- |
| **Herb strategy** | **Number of components** | **Comonent ID** |
| HQ YGZ CH KS | 1 | MOL000098 |
| HQ YGZ CH | 1 | MOL000422 |
| HQ KS JXT | 1 | MOL000392 |
| YGZ KS JXT | 1 | MOL000006 |
| JH CH JXT | 1 | MOL000449 |
| HQ CH | 1 | MOL000354 |
| HQ JXT | 2 | MOL000417 |
|  |  | MOL000033 |
| YGZ JXT | 2 | MOL000492 |
|  |  | MOL000358 |
| CH JXT | 1 | MOL000490 |
| HQ | 11 | MOL000371 |
|  |  | MOL000439 |
|  |  | MOL000378 |
|  |  | MOL000379 |
|  |  | MOL000387 |
|  |  | MOL000380 |
|  |  | MOL000442 |
|  |  | MOL000433 |
|  |  | MOL000239 |
|  |  | MOL000211 |
|  |  | MOL000438 |
| YGZ | 9 | MOL006793 |
|  |  | MOL006824 |
|  |  | MOL000569 |
|  |  | MOL006812 |
|  |  | MOL001002 |
|  |  | MOL006821 |
|  |  | MOL006799 |
|  |  | MOL006826 |
|  |  | MOL006796 |
| KS | 33 | MOL005944 |
|  |  | MOL006568 |
|  |  | MOL006650 |
|  |  | MOL006571 |
|  |  | MOL006626 |
|  |  | MOL006613 |
|  |  | MOL001484 |
|  |  | MOL001040 |
|  |  | MOL006582 |
|  |  | MOL006570 |
|  |  | MOL003347 |
|  |  | MOL004580 |
|  |  | MOL000456 |
|  |  | MOL003627 |
|  |  | MOL005100 |
|  |  | MOL006573 |
|  |  | MOL004941 |
|  |  | MOL006623 |
|  |  | MOL006596 |
|  |  | MOL006563 |
|  |  | MOL006627 |
|  |  | MOL006628 |
|  |  | MOL006604 |
|  |  | MOL006564 |
|  |  | MOL003680 |
|  |  | MOL003542 |
|  |  | MOL003673 |
|  |  | MOL006562 |
|  |  | MOL006630 |
|  |  | MOL003648 |
|  |  | MOL006565 |
|  |  | MOL006569 |
|  |  | MOL006561 |
| JH | 1 | MOL000953 |
| CH | 8 | MOL004609 |
|  |  | MOL004644 |
|  |  | MOL004598 |
|  |  | MOL004624 |
|  |  | MOL013187 |
|  |  | MOL004653 |
|  |  | MOL002776 |
|  |  | MOL001645 |
| JXT | 11 | MOL000500 |
|  |  | MOL000483 |
|  |  | MOL000503 |
|  |  | MOL000469 |
|  |  | MOL000468 |
|  |  | MOL000507 |
|  |  | MOL000471 |
|  |  | MOL000502 |
|  |  | MOL000506 |
|  |  | MOL000491 |
|  |  | MOL000497 |

| **Table S3. Component ID and targets** | |
| --- | --- |
| **Component ID** | **Target** |
| MOL000211 | NR3C1 |
|  | VDR |
|  | FABP1 |
|  | FAAH |
|  | POLB |
|  | CYP17A1 |
|  | PPARD |
|  | CES2 |
|  | AKR1B10 |
|  | UBA2 |
|  | FABP4 |
|  | CDC25B |
|  | PTGES |
|  | HSF1 |
|  | FFAR1 |
|  | NPC1L1 |
|  | GABRB2 |
|  | PTPRF |
|  | PTPN2 |
|  | GABBR1 |
|  | TOP2A |
|  | SCD |
|  | SHBG |
|  | PTPN1 |
|  | FABP5 |
|  | GABRA2 |
|  | ACP1 |
|  | PPARG |
|  | SLC6A3 |
|  | SERPINA6 |
|  | SIGMAR1 |
|  | PDE4D |
|  | CYP19A1 |
|  | HSD17B3 |
|  | TERT |
|  | ADORA3 |
|  | NR1H4 |
|  | FABP3 |
|  | UGT2B7 |
|  | FABP2 |
|  | GPBAR1 |
|  | SAE1 |
|  | RORC |
|  | PLA2G1B |
|  | CDC25C |
|  | TOP1 |
|  | AR |
|  | PTPN11 |
|  | GABRG2 |
|  | CYP51A1 |
|  | CDC25A |
|  | HSD11B1 |
| MOL000239 | MMP12 |
|  | CA2 |
|  | SYK |
|  | BACE1 |
|  | CA3 |
|  | MPG |
|  | KIT |
|  | MMP2 |
|  | ESR2 |
|  | AKR1B10 |
|  | CDK1 |
|  | AURKB |
|  | PTK2 |
|  | CSNK2A1 |
|  | AKT1 |
|  | NUAK1 |
|  | PIK3R1 |
|  | XDH |
|  | ACHE |
|  | PLG |
|  | CCNB3 |
|  | TYR |
|  | GSK3B |
|  | CA14 |
|  | CA4 |
|  | DAPK1 |
|  | HSD17B1 |
|  | ALOX15 |
|  | BCHE |
|  | DRD4 |
|  | CYP19A1 |
|  | CDK5R1 |
|  | MCL1 |
|  | TERT |
|  | HSD17B2 |
|  | KDR |
|  | F2 |
|  | SLC22A12 |
|  | CA9 |
|  | ABCG2 |
|  | CDK6 |
|  | CA12 |
|  | GLO1 |
|  | PTGS2 |
|  | NOS2 |
|  | TAS2R31 |
|  | ARG1 |
|  | CAMK2B |
|  | CA13 |
|  | NAE1 |
|  | TNKS2 |
|  | MMP13 |
|  | OPRD1 |
|  | PIK3CG |
|  | PLK1 |
|  | CCNB1 |
|  | CYP1B1 |
|  | APP |
|  | PTPRS |
|  | TOP2A |
|  | ADORA2A |
|  | EGFR |
|  | PTPN1 |
|  | APEX1 |
|  | IGF1R |
|  | ABCC1 |
|  | SRC |
|  | MMP3 |
|  | GPR35 |
|  | MET |
|  | NOX4 |
|  | CDK2 |
|  | CA1 |
|  | AKR1C1 |
|  | MYLK |
|  | ABCB1 |
|  | AXL |
|  | ESRRA |
|  | AHR |
|  | AVPR2 |
|  | CDK5 |
|  | ADORA3 |
|  | CA5A |
|  | TNKS |
|  | AMY1A |
|  | ODC1 |
|  | FLT3 |
|  | ALK |
|  | PIM1 |
|  | MAOA |
|  | CCNB2 |
|  | CA7 |
|  | ADORA1 |
|  | ALOX5 |
|  | CA6 |
|  | MMP9 |
|  | NEK6 |
|  | PARP1 |
|  | AR |
|  | AKR1B1 |
|  | ESR1 |
|  | KDM4E |
|  | OPRM1 |
| MOL000033 | PREP |
|  | POLB |
|  | SQLE |
|  | VDR |
|  | PPARD |
|  | HMGCR |
|  | CYP17A1 |
|  | CES2 |
|  | G6PD |
|  | SREBF2 |
|  | ESR2 |
|  | GLRA1 |
|  | PTPN6 |
|  | PTGES |
|  | SLC6A4 |
|  | NPC1L1 |
|  | PTPN2 |
|  | SLC6A2 |
|  | SHBG |
|  | PTPN1 |
|  | ACHE |
|  | CYP2C19 |
|  | PPARG |
|  | PTGER1 |
|  | NR1H2 |
|  | SERPINA6 |
|  | NR1H3 |
|  | BCHE |
|  | CYP19A1 |
|  | CHRM2 |
|  | UGT2B7 |
|  | RORA |
|  | RORC |
|  | TBXAS1 |
|  | NR1I3 |
|  | AR |
|  | CYP51A1 |
|  | FDFT1 |
|  | ESR1 |
|  | NOS2 |
|  | PTGER2 |
|  | HSD11B1 |
|  | DHCR7 |
| MOL000354 | TNKS2 |
|  | CA2 |
|  | SYK |
|  | BACE1 |
|  | MPG |
|  | CA3 |
|  | ESR2 |
|  | AKR1C3 |
|  | MMP2 |
|  | AKR1B10 |
|  | CDK1 |
|  | AURKB |
|  | CSNK2A1 |
|  | PTK2 |
|  | AKT1 |
|  | NUAK1 |
|  | PIK3R1 |
|  | XDH |
|  | PLG |
|  | ACHE |
|  | CCNB3 |
|  | TYR |
|  | GSK3B |
|  | CA14 |
|  | CA4 |
|  | HSD17B1 |
|  | DAPK1 |
|  | ALOX15 |
|  | CYP19A1 |
|  | DRD4 |
|  | MCL1 |
|  | CDK5R1 |
|  | PYGL |
|  | TERT |
|  | HSD17B2 |
|  | KDR |
|  | F2 |
|  | SLC22A12 |
|  | CA9 |
|  | ABCG2 |
|  | CDK6 |
|  | CA12 |
|  | AKR1C2 |
|  | PLA2G1B |
|  | AKR1A1 |
|  | MAPT |
|  | GLO1 |
|  | NEK2 |
|  | ARG1 |
|  | CA13 |
|  | CAMK2B |
|  | INSR |
|  | MMP13 |
|  | MMP12 |
|  | PIK3CG |
|  | PLK1 |
|  | CCNB1 |
|  | ALOX12 |
|  | APP |
|  | CYP1B1 |
|  | PTPRS |
|  | TOP2A |
|  | EGFR |
|  | ADORA2A |
|  | APEX1 |
|  | IGF1R |
|  | ABCC1 |
|  | SRC |
|  | PKN1 |
|  | CD38 |
|  | GPR35 |
|  | MMP3 |
|  | MET |
|  | CDK2 |
|  | NOX4 |
|  | CA1 |
|  | AKR1C1 |
|  | MYLK |
|  | ABCB1 |
|  | ESRRA |
|  | AHR |
|  | AXL |
|  | CDK5 |
|  | AVPR2 |
|  | AKR1C4 |
|  | CA5A |
|  | FLT3 |
|  | PIM1 |
|  | MAOA |
|  | ALK |
|  | MPO |
|  | CCNB2 |
|  | CA7 |
|  | ADORA1 |
|  | ALOX5 |
|  | CA6 |
|  | MMP9 |
|  | NEK6 |
|  | PARP1 |
|  | TTR |
|  | CXCR1 |
|  | AKR1B1 |
|  | KDM4E |
| MOL000371 | CDK8 |
|  | SYK |
|  | KIT |
|  | GABRA3 |
|  | CDK4 |
|  | ROCK1 |
|  | MTOR |
|  | GABRB3 |
|  | GABRA5 |
|  | BRAF |
|  | HSP90B1 |
|  | GABRA2 |
|  | BRPF1 |
|  | PIM3 |
|  | PDGFRA |
|  | MERTK |
|  | CCNA1 |
|  | CLK4 |
|  | ROCK2 |
|  | GRM1 |
|  | BRD4 |
|  | CYP19A1 |
|  | MCL1 |
|  | HCRTR2 |
|  | ELANE |
|  | IKBKE |
|  | FPR2 |
|  | CSNK1D |
|  | HSD17B2 |
|  | PLAU |
|  | SLC1A3 |
|  | KDR |
|  | SLC27A1 |
|  | NQO2 |
|  | ALOX5AP |
|  | NTRK1 |
|  | TBXAS1 |
|  | PDE3A |
|  | PDGFRB |
|  | PIK3CB |
|  | CLK2 |
|  | PLK3 |
|  | BDKRB2 |
|  | TUBB1 |
|  | EPHX2 |
|  | CYP11B1 |
|  | SCN9A |
|  | SIRT2 |
|  | PIK3CG |
|  | ALOX12 |
|  | KCNK9 |
|  | TUBB3 |
|  | GABRA1 |
|  | AURKA |
|  | PIK3CD |
|  | PDE5A |
|  | PDE10A |
|  | ADORA2A |
|  | MAP2K1 |
|  | RGS4 |
|  | PTPN1 |
|  | NPBWR1 |
|  | BRD3 |
|  | SRC |
|  | FGFR1 |
|  | HCRTR1 |
|  | CRHR1 |
|  | PDE2A |
|  | MET |
|  | CDK2 |
|  | ABCB1 |
|  | CNR1 |
|  | CCNE1 |
|  | CYP1A1 |
|  | DHFR |
|  | BRD2 |
|  | CNR2 |
|  | CCND1 |
|  | ABL1 |
|  | PIK3CA |
|  | FLT3 |
|  | CCNA2 |
|  | DYRK1B |
|  | PPP1CA |
|  | PDE3B |
|  | PRKCQ |
|  | TTK |
|  | LRRK2 |
|  | HTR3A |
|  | RPS6KB1 |
|  | PARP1 |
|  | PLK2 |
|  | CCNE2 |
|  | PGK1 |
|  | AR |
|  | GABRG2 |
|  | CCNC |
|  | GABRA6 |
|  | CLK1 |
|  | MAPKAPK2 |
|  | PKN2 |
|  | HSD11B1 |
| MOL000378 | ALDH3A1 |
|  | TRPM8 |
|  | SYK |
|  | TLR9 |
|  | QPCTL |
|  | PLK1 |
|  | CCNB1 |
|  | ALOX12 |
|  | CDK1 |
|  | CDK4 |
|  | AURKB |
|  | ALPL |
|  | CDC25B |
|  | PTK2 |
|  | MKNK1 |
|  | PDE10A |
|  | CLK3 |
|  | CDK7 |
|  | GSK3B |
|  | WEE1 |
|  | TGFBR1 |
|  | CA14 |
|  | CCNA1 |
|  | CHEK1 |
|  | F3 |
|  | MET |
|  | CDK2 |
|  | NOX4 |
|  | CLK4 |
|  | ALOX15 |
|  | SIGMAR1 |
|  | HSD17B3 |
|  | LDHB |
|  | DRD2 |
|  | CCNH |
|  | ADAMTS5 |
|  | CHRNA7 |
|  | ABL1 |
|  | KDR |
|  | ALOX15B |
|  | CCNA2 |
|  | CDK9 |
|  | DYRK1B |
|  | MMP1 |
|  | CDC25C |
|  | CLK2 |
|  | RET |
|  | NDUFA4 |
|  | MAOB |
|  | CLK1 |
|  | CDC25A |
|  | CCNT1 |
| MOL000379 | CDK1 |
|  | EIF4A1 |
|  | MGMT |
|  | HK2 |
|  | ADORA3 |
|  | ABL1 |
|  | SLC28A2 |
|  | SLC5A2 |
|  | NADK |
|  | PDE5A |
|  | SLC2A1 |
|  | ADORA2B |
|  | ADORA2A |
|  | PDGFRB |
|  | ADORA1 |
|  | HSPA5 |
|  | SLC29A1 |
|  | ADK |
|  | TYR |
|  | PDGFRA |
|  | PTGS1 |
|  | CA14 |
|  | HK1 |
|  | PTGS2 |
|  | CDK2 |
| MOL000380 | ABL1 |
|  | CA2 |
|  | SYK |
|  | MMP8 |
|  | ESR2 |
|  | MMP25 |
|  | MMP2 |
|  | KIT |
|  | HDAC4 |
|  | VCP |
|  | CDK1 |
|  | CDK4 |
|  | MTOR |
|  | CCND2 |
|  | DNM1 |
|  | PIK3R1 |
|  | CCNB3 |
|  | PLAA |
|  | GSK3B |
|  | CA14 |
|  | PTGS1 |
|  | MBD2 |
|  | CHEK1 |
|  | CA4 |
|  | ALOX15 |
|  | CYP19A1 |
|  | HSD17B3 |
|  | CDK5R1 |
|  | MMP16 |
|  | CASP7 |
|  | HSD17B2 |
|  | KDR |
|  | PIM2 |
|  | CA9 |
|  | CA12 |
|  | CCND3 |
|  | PIK3CB |
|  | MMP7 |
|  | LNPEP |
|  | RET |
|  | CTSS |
|  | ANPEP |
|  | TUBB1 |
|  | CA13 |
|  | GRM4 |
|  | LCK |
|  | PIK3CG |
|  | HDAC11 |
|  | RAF1 |
|  | ALOX12 |
|  | CCNB1 |
|  | DBF4 |
|  | TUBB3 |
|  | CDC7 |
|  | PIK3CD |
|  | AURKA |
|  | ADAM10 |
|  | ALPL |
|  | COMT |
|  | MIF |
|  | MKNK1 |
|  | MAP2K1 |
|  | ADORA2A |
|  | CLK3 |
|  | HDAC9 |
|  | PTPN1 |
|  | PDE4C |
|  | WEE1 |
|  | NCOR2 |
|  | SRC |
|  | FGFR1 |
|  | MET |
|  | ALPG |
|  | CDK2 |
|  | CA1 |
|  | RPS6KA2 |
|  | ABCB1 |
|  | ERN1 |
|  | CDK5 |
|  | CA5B |
|  | CASP3 |
|  | CCND1 |
|  | CA5A |
|  | NCOR1 |
|  | PIK3CA |
|  | ADORA2B |
|  | DYRK2 |
|  | CCNB2 |
|  | HTT |
|  | BMP1 |
|  | HDAC5 |
|  | NR4A1 |
|  | JAK3 |
|  | MMP9 |
|  | PARP1 |
|  | EPHB2 |
|  | AR |
|  | ADAM17 |
|  | HDAC2 |
|  | PDE7A |
|  | CLK1 |
|  | ESR1 |
|  | MAPKAPK2 |
|  | HDAC7 |
|  | HDAC3 |
|  | EZR |
| MOL000387 | JUN |
|  | AVPR1A |
|  | SYK |
|  | CTSG |
|  | AVPR2 |
|  | JAK3 |
|  | OXTR |
|  | GSK3B |
|  | CMA1 |
|  | PTK2 |
|  | F2 |
|  | EGFR |
|  | ABCG2 |
| MOL000392 | BACE1 |
|  | TNKS2 |
|  | CA2 |
|  | PON1 |
|  | PFKFB3 |
|  | CA3 |
|  | TLR9 |
|  | ESR2 |
|  | RAF1 |
|  | ALOX12 |
|  | HTR2C |
|  | PTPRS |
|  | MIF |
|  | ERCC5 |
|  | SLC6A2 |
|  | EGFR |
|  | ADORA2A |
|  | PTPN1 |
|  | FEN1 |
|  | XDH |
|  | ACHE |
|  | TYR |
|  | CA14 |
|  | PTGS1 |
|  | MGAM |
|  | CA4 |
|  | NOX4 |
|  | HSD17B1 |
|  | CA1 |
|  | ALOX15 |
|  | ABCB1 |
|  | CYP19A1 |
|  | HTR2A |
|  | ESRRA |
|  | PPARA |
|  | STS |
|  | DHODH |
|  | CA5B |
|  | CA5A |
|  | HSD17B2 |
|  | ESRRB |
|  | TNKS |
|  | MAOA |
|  | CA9 |
|  | ABCG2 |
|  | TBXAS1 |
|  | CA12 |
|  | CA7 |
|  | CBR1 |
|  | CA6 |
|  | ADORA1 |
|  | ALDH2 |
|  | IL2 |
|  | MAOB |
|  | ESR1 |
|  | CA13 |
| MOL000417 | HSP90AB1 |
|  | CA2 |
|  | PON1 |
|  | PFKFB3 |
|  | PLAT |
|  | TLR9 |
|  | ESR2 |
|  | ALOX12 |
|  | CDC7 |
|  | CYP1B1 |
|  | HTR2C |
|  | CDC25B |
|  | PTPRS |
|  | MIF |
|  | SLC6A2 |
|  | ADORA2A |
|  | EGFR |
|  | PTPN1 |
|  | ABCC1 |
|  | XDH |
|  | ACHE |
|  | TYR |
|  | PTGS1 |
|  | F10 |
|  | MGAM |
|  | CA4 |
|  | NOX4 |
|  | HSD17B1 |
|  | CA1 |
|  | ALOX15 |
|  | ABCB1 |
|  | CYP19A1 |
|  | HTR2A |
|  | ESRRA |
|  | PPARA |
|  | STS |
|  | CA5B |
|  | HSD17B2 |
|  | PLAU |
|  | ESRRB |
|  | MAOA |
|  | ABCG2 |
|  | CA12 |
|  | TBXAS1 |
|  | CBR1 |
|  | CA7 |
|  | ADORA1 |
|  | DUSP3 |
|  | ALDH2 |
|  | IL2 |
|  | MAOB |
|  | ESR1 |
| MOL000422 | BCHE |
|  | CA2 |
|  | PFKFB3 |
|  | BACE1 |
|  | SYK |
|  | CA3 |
|  | MPG |
|  | AKR1C3 |
|  | ESR2 |
|  | MMP2 |
|  | AKR1B10 |
|  | AURKB |
|  | CDK1 |
|  | PTK2 |
|  | CSNK2A1 |
|  | AKT1 |
|  | NUAK1 |
|  | PIK3R1 |
|  | XDH |
|  | ACHE |
|  | CCNB3 |
|  | GSK3B |
|  | TYR |
|  | GRK6 |
|  | CA14 |
|  | CA4 |
|  | DAPK1 |
|  | HSD17B1 |
|  | ALOX15 |
|  | DRD4 |
|  | CYP19A1 |
|  | PYGL |
|  | CDK5R1 |
|  | TERT |
|  | HSD17B2 |
|  | KDR |
|  | F2 |
|  | SLC22A12 |
|  | CA9 |
|  | ABCG2 |
|  | CDK6 |
|  | CA12 |
|  | AKR1C2 |
|  | PLA2G1B |
|  | AKR1A1 |
|  | GLO1 |
|  | NEK2 |
|  | PTGS2 |
|  | ARG1 |
|  | CA13 |
|  | CAMK2B |
|  | TNKS2 |
|  | MMP13 |
|  | MMP12 |
|  | CFTR |
|  | PLK1 |
|  | ALOX12 |
|  | CCNB1 |
|  | APP |
|  | CYP1B1 |
|  | PTPRS |
|  | ADORA2A |
|  | EGFR |
|  | ABCC1 |
|  | IGF1R |
|  | PKN1 |
|  | SRC |
|  | CD38 |
|  | MMP3 |
|  | GPR35 |
|  | MET |
|  | NOX4 |
|  | CDK2 |
|  | AKR1C1 |
|  | CA1 |
|  | ABCB1 |
|  | AXL |
|  | ESRRA |
|  | AHR |
|  | CDK5 |
|  | AVPR2 |
|  | AKR1C4 |
|  | CA5A |
|  | TNKS |
|  | AMY1A |
|  | FLT3 |
|  | ALK |
|  | MAOA |
|  | PIM1 |
|  | MPO |
|  | CA7 |
|  | CCNB2 |
|  | CA6 |
|  | ALOX5 |
|  | ADORA1 |
|  | NEK6 |
|  | MMP9 |
|  | PARP1 |
|  | TOP1 |
|  | TTR |
|  | CXCR1 |
|  | ESR1 |
|  | AKR1B1 |
| MOL000433 | CASP3 |
|  | HDAC1 |
|  | HDAC6 |
|  | SLC19A1 |
|  | DHFR |
|  | SLC46A1 |
|  | FOLR1 |
|  | GART |
|  | SF3B3 |
|  | ATIC |
|  | FPGS |
|  | PYGM |
|  | TYMS |
|  | MME |
|  | PSMB5 |
|  | CSNK2A2 |
|  | HDAC8 |
|  | FOLR2 |
|  | AKR1B1 |
| MOL000438 | PDE7A |
|  | SYK |
|  | MMP8 |
|  | PRF1 |
|  | MMP25 |
|  | HSP90AB1 |
|  | HDAC4 |
|  | VCP |
|  | CDK1 |
|  | CDK4 |
|  | AURKB |
|  | MTOR |
|  | ROCK1 |
|  | CCND2 |
|  | DNM1 |
|  | PIK3R1 |
|  | CCNB3 |
|  | PIM3 |
|  | TYR |
|  | GSK3B |
|  | PTGS1 |
|  | CHEK1 |
|  | MAPK1 |
|  | ALOX15 |
|  | SIGMAR1 |
|  | CYP19A1 |
|  | IMPDH2 |
|  | HSD17B3 |
|  | CDK5R1 |
|  | MMP16 |
|  | CASP7 |
|  | HSD17B2 |
|  | KDR |
|  | PRKCA |
|  | IMPDH1 |
|  | PIM2 |
|  | MMP1 |
|  | CCND3 |
|  | PIK3CB |
|  | EP300 |
|  | MMP7 |
|  | LNPEP |
|  | RET |
|  | TAAR1 |
|  | CTSS |
|  | HDAC10 |
|  | FLT1 |
|  | LCK |
|  | PIK3CG |
|  | HDAC11 |
|  | RAF1 |
|  | PLK1 |
|  | CCNB1 |
|  | ALOX12 |
|  | AURKA |
|  | PIK3CD |
|  | ADAM10 |
|  | PDK1 |
|  | DAO |
|  | MIF |
|  | MKNK1 |
|  | EGFR |
|  | ADORA2A |
|  | CLK3 |
|  | HDAC9 |
|  | WEE1 |
|  | NCOR2 |
|  | SRC |
|  | MET |
|  | CDK2 |
|  | RPS6KA2 |
|  | CCNE1 |
|  | BAD |
|  | CDK5 |
|  | CASP3 |
|  | HSP90AA1 |
|  | CCND1 |
|  | ABL1 |
|  | RPS6KA1 |
|  | PIK3CA |
|  | NCOR1 |
|  | CSNK1G1 |
|  | SGK1 |
|  | ALOX15B |
|  | FLT3 |
|  | ALK |
|  | PIM1 |
|  | ADORA2B |
|  | DYRK2 |
|  | CCNB2 |
|  | HTT |
|  | BMP1 |
|  | HDAC5 |
|  | NR4A1 |
|  | CCNE2 |
|  | EPHB2 |
|  | ADAM17 |
|  | FCER2 |
|  | MAOB |
|  | HDAC2 |
|  | CLK1 |
|  | MAPKAPK2 |
|  | HDAC7 |
|  | HDAC3 |
|  | EZR |
| MOL000439 | ADORA2B |
|  | EPHX2 |
|  | CHIA |
|  | ADORA1 |
|  | SLC5A1 |
|  | ADORA3 |
|  | TYR |
|  | PTGS1 |
|  | SLC5A2 |
|  | HRAS |
|  | ADORA2A |
| MOL000442 | PLA2G1B |
| MOL000098 | TERT |
|  | CA2 |
|  | SYK |
|  | BACE1 |
|  | MPG |
|  | CA3 |
|  | ESR2 |
|  | AKR1C3 |
|  | MMP2 |
|  | AKR1B10 |
|  | CDK1 |
|  | AURKB |
|  | CSNK2A1 |
|  | PTK2 |
|  | AKT1 |
|  | NUAK1 |
|  | PIK3R1 |
|  | XDH |
|  | ACHE |
|  | CCNB3 |
|  | TYR |
|  | GSK3B |
|  | CA14 |
|  | CA4 |
|  | HSD17B1 |
|  | DAPK1 |
|  | ALOX15 |
|  | CYP19A1 |
|  | DRD4 |
|  | CDK5R1 |
|  | PYGL |
|  | HSD17B2 |
|  | KDR |
|  | F2 |
|  | SLC22A12 |
|  | CA9 |
|  | ABCG2 |
|  | CDK6 |
|  | CA12 |
|  | AKR1C2 |
|  | PLA2G1B |
|  | AKR1A1 |
|  | MAPT |
|  | GLO1 |
|  | NEK2 |
|  | ARG1 |
|  | CA13 |
|  | CAMK2B |
|  | TNKS2 |
|  | INSR |
|  | MMP13 |
|  | MMP12 |
|  | PIK3CG |
|  | PLK1 |
|  | CCNB1 |
|  | ALOX12 |
|  | APP |
|  | CYP1B1 |
|  | PTPRS |
|  | TOP2A |
|  | EGFR |
|  | ADORA2A |
|  | APEX1 |
|  | IGF1R |
|  | ABCC1 |
|  | SRC |
|  | PKN1 |
|  | CD38 |
|  | GPR35 |
|  | MMP3 |
|  | MET |
|  | CDK2 |
|  | NOX4 |
|  | CA1 |
|  | AKR1C1 |
|  | MYLK |
|  | ABCB1 |
|  | ESRRA |
|  | AHR |
|  | AXL |
|  | CDK5 |
|  | AVPR2 |
|  | AKR1C4 |
|  | CA5A |
|  | TNKS |
|  | FLT3 |
|  | PIM1 |
|  | MAOA |
|  | ALK |
|  | MPO |
|  | CCNB2 |
|  | CA7 |
|  | ALOX5 |
|  | ADORA1 |
|  | CA6 |
|  | MMP9 |
|  | NEK6 |
|  | PARP1 |
|  | TOP1 |
|  | TTR |
|  | CXCR1 |
|  | AKR1B1 |
|  | KDM4E |
| MOL001002 | SNCA |
|  | SQLE |
|  | CA2 |
|  | INSR |
|  | FLT4 |
|  | BACE1 |
|  | CES2 |
|  | ESR2 |
|  | LYN |
|  | MAP3K8 |
|  | PLK1 |
|  | AURKB |
|  | CDK4 |
|  | AURKA |
|  | DAO |
|  | CSNK2A1 |
|  | PTK2 |
|  | BRAF |
|  | EGFR |
|  | PTPN1 |
|  | AKT1 |
|  | NUAK1 |
|  | XDH |
|  | IGF1R |
|  | FGR |
|  | GSK3B |
|  | GSR |
|  | CA14 |
|  | SRC |
|  | GPR35 |
|  | CCNA1 |
|  | CA4 |
|  | MET |
|  | CDK2 |
|  | CA1 |
|  | HSD17B3 |
|  | CDK5 |
|  | CDK5R1 |
|  | TNNI3 |
|  | TEK |
|  | CA5B |
|  | TNNT2 |
|  | CA5A |
|  | CCND1 |
|  | KDR |
|  | PLK4 |
|  | MAOA |
|  | CCNA2 |
|  | CA9 |
|  | EPHB4 |
|  | CA12 |
|  | CBR1 |
|  | TNNC1 |
|  | CA7 |
|  | ALOX5 |
|  | PDGFRB |
|  | CA6 |
|  | ESR1 |
|  | PTGS2 |
|  | AKR1B1 |
|  | ERBB2 |
|  | CA13 |
|  | HSPA1A |
| MOL000358 | POLB |
|  | SQLE |
|  | VDR |
|  | PPARD |
|  | CYP17A1 |
|  | HMGCR |
|  | CES2 |
|  | G6PD |
|  | SREBF2 |
|  | ESR2 |
|  | GLRA1 |
|  | PTPN6 |
|  | PTGES |
|  | SLC6A4 |
|  | NPC1L1 |
|  | PTPN2 |
|  | SLC6A2 |
|  | SHBG |
|  | PTPN1 |
|  | ACHE |
|  | CYP2C19 |
|  | PPARG |
|  | PTGER1 |
|  | NR1H2 |
|  | SERPINA6 |
|  | NR1H3 |
|  | BCHE |
|  | CYP19A1 |
|  | CHRM2 |
|  | UGT2B7 |
|  | RORA |
|  | RORC |
|  | TBXAS1 |
|  | NR1I3 |
|  | AR |
|  | CYP51A1 |
|  | ESR1 |
|  | NOS2 |
|  | PTGER2 |
|  | HSD11B1 |
|  | DHCR7 |
| MOL000569 | FUT7 |
|  | CA2 |
|  | POLB |
|  | POLA1 |
|  | CA3 |
|  | CA5A |
|  | MAOA |
|  | CA9 |
|  | CA12 |
|  | CA7 |
|  | CA6 |
|  | CA14 |
|  | MAOB |
|  | SERPINE1 |
|  | TUBB1 |
|  | CA1 |
| MOL000006 | AR |
|  | CA2 |
|  | PFKFB3 |
|  | BACE1 |
|  | SYK |
|  | CA3 |
|  | AKR1C3 |
|  | ESR2 |
|  | MMP2 |
|  | AKR1B10 |
|  | AURKB |
|  | CDK1 |
|  | PTK2 |
|  | CSNK2A1 |
|  | AKT1 |
|  | NUAK1 |
|  | PIK3R1 |
|  | XDH |
|  | PLG |
|  | ACHE |
|  | CCNB3 |
|  | TYR |
|  | GSK3B |
|  | CA14 |
|  | GRK6 |
|  | CA4 |
|  | DAPK1 |
|  | HSD17B1 |
|  | ALOX15 |
|  | DRD4 |
|  | CYP19A1 |
|  | PYGL |
|  | CDK5R1 |
|  | TERT |
|  | HSD17B2 |
|  | KDR |
|  | F2 |
|  | CA9 |
|  | ABCG2 |
|  | CDK6 |
|  | CA12 |
|  | AKR1C2 |
|  | PLA2G1B |
|  | AKR1A1 |
|  | GLO1 |
|  | NEK2 |
|  | PTGS2 |
|  | ARG1 |
|  | CA13 |
|  | CAMK2B |
|  | MMP13 |
|  | TNKS2 |
|  | MMP12 |
|  | CFTR |
|  | PLK1 |
|  | CCNB1 |
|  | ALOX12 |
|  | APP |
|  | CYP1B1 |
|  | PTPRS |
|  | ADORA2A |
|  | EGFR |
|  | ABCC1 |
|  | IGF1R |
|  | PKN1 |
|  | SRC |
|  | MMP3 |
|  | CD38 |
|  | GPR35 |
|  | MET |
|  | NOX4 |
|  | CDK2 |
|  | AKR1C1 |
|  | CA1 |
|  | ABCB1 |
|  | AXL |
|  | AHR |
|  | ESRRA |
|  | CDK5 |
|  | AVPR2 |
|  | AKR1C4 |
|  | CA5A |
|  | TNKS |
|  | AMY1A |
|  | FLT3 |
|  | ALK |
|  | PIM1 |
|  | MAOA |
|  | MPO |
|  | CCNB2 |
|  | CA7 |
|  | CA6 |
|  | ALOX5 |
|  | ADORA1 |
|  | NEK6 |
|  | MMP9 |
|  | PARP1 |
|  | TOP1 |
|  | TTR |
|  | CXCR1 |
|  | AKR1B1 |
|  | ESR1 |
|  | KDM4E |
| MOL006793 | ABCB1 |
|  | ACHE |
|  | BACE1 |
|  | SERPINE1 |
|  | ADORA1 |
|  | P2RY1 |
| MOL006796 | ABCB1 |
|  | SERPINE1 |
|  | ACHE |
|  | BACE1 |
|  | SQLE |
|  | F10 |
|  | PTPN1 |
|  | PTPN2 |
| MOL006799 | HSP90AA1 |
|  | GLRA2 |
|  | VDR |
|  | PSENEN |
|  | ENGASE |
|  | VEGFA |
|  | SELL |
|  | GLRA1 |
|  | CDK1 |
|  | HTR2C |
|  | ADRA2A |
|  | ADRA1A |
|  | CYP2D6 |
|  | YARS |
|  | OPRK1 |
|  | FGF1 |
|  | APH1A |
|  | ADRA2C |
|  | PSEN2 |
|  | STAT3 |
|  | NCSTN |
|  | ADRA1D |
|  | HTR2A |
|  | HTR6 |
|  | PPM1A |
|  | DRD2 |
|  | HPSE |
|  | DRD3 |
|  | HTR1B |
|  | PTAFR |
|  | RORC |
|  | OGA |
|  | ADORA1 |
|  | APH1B |
|  | HTR2B |
|  | DRD1 |
|  | ADRA2B |
|  | PSEN1 |
|  | OPRM1 |
| MOL006812 | PIK3CA |
|  | FKBP5 |
|  | CA2 |
|  | SYK |
|  | POLA1 |
|  | GPR119 |
|  | PTGER3 |
|  | MAP3K12 |
|  | LYN |
|  | GABRA3 |
|  | FNTB |
|  | AURKB |
|  | MTOR |
|  | GABRB3 |
|  | GABRA5 |
|  | AKT1 |
|  | SHBG |
|  | PIK3R1 |
|  | JAK2 |
|  | GABRA2 |
|  | GSK3B |
|  | PPARG |
|  | SMO |
|  | CCNA1 |
|  | PDPK1 |
|  | CHEK1 |
|  | TNFRSF1A |
|  | CA4 |
|  | CLK4 |
|  | GRM1 |
|  | ALOX15 |
|  | PDE4D |
|  | CYP19A1 |
|  | CYP24A1 |
|  | STS |
|  | KDR |
|  | LYPLA1 |
|  | PTAFR |
|  | PDE3A |
|  | CFD |
|  | PIK3CB |
|  | TNK2 |
|  | CLK2 |
|  | LYPLA2 |
|  | PDE4A |
|  | FLT1 |
|  | DYRK3 |
|  | HTR1A |
|  | GRM4 |
|  | PIK3CG |
|  | GCK |
|  | MDM2 |
|  | ALOX12 |
|  | GABRA1 |
|  | AURKA |
|  | PIK3CD |
|  | KCNA5 |
|  | PDE10A |
|  | MAPK8 |
|  | MAP2K1 |
|  | ADORA2A |
|  | CLK3 |
|  | FNTA |
|  | ROS1 |
|  | PTGFR |
|  | IGF1R |
|  | TYK2 |
|  | SRC |
|  | CD38 |
|  | CRHR1 |
|  | PDE2A |
|  | HCRTR1 |
|  | SELE |
|  | ICAM1 |
|  | CDK2 |
|  | ACKR3 |
|  | CA1 |
|  | UTS2R |
|  | CCNE1 |
|  | DYRK1A |
|  | CNR1 |
|  | AVPR1B |
|  | PDE4B |
|  | CNR2 |
|  | FLT3 |
|  | DYRK1B |
|  | CCNA2 |
|  | PRKDC |
|  | PDE3B |
|  | DYRK2 |
|  | PDE11A |
|  | RPS6KB1 |
|  | ALOX5 |
|  | JAK3 |
|  | OXTR |
|  | CCNE2 |
|  | NR3C1 |
|  | TTR |
|  | MAPK10 |
|  | JAK1 |
|  | AR |
|  | GABRG2 |
|  | CDK8 |
|  | CLK1 |
|  | CCNC |
|  | ERBB2 |
| MOL006821 | DNM1 |
|  | MMP13 |
|  | CA2 |
|  | SQLE |
|  | PGD |
|  | BACE1 |
|  | MMP12 |
|  | CA3 |
|  | MMP14 |
|  | VEGFA |
|  | MMP2 |
|  | KLK1 |
|  | GABRA1 |
|  | APP |
|  | GABRB2 |
|  | ABCC1 |
|  | KCNH2 |
|  | PTGS1 |
|  | FUT4 |
|  | CA4 |
|  | MET |
|  | CA1 |
|  | DYRK1A |
|  | ABCB1 |
|  | PGF |
|  | FUT7 |
|  | BCL2 |
|  | TERT |
|  | MAPK14 |
|  | CA5B |
|  | CA5A |
|  | HIF1A |
|  | ST3GAL3 |
|  | DNMT1 |
|  | CA9 |
|  | ABCG2 |
|  | CA12 |
|  | STAT1 |
|  | CA7 |
|  | KLK2 |
|  | CA6 |
|  | MMP9 |
|  | MAPT |
|  | GABRG2 |
|  | TAS2R31 |
|  | CA13 |
| MOL006824 | UGT2B7 |
|  | FAAH |
|  | POLB |
|  | FABP1 |
|  | SQLE |
|  | PPARD |
|  | HMGCR |
|  | CYP17A1 |
|  | CES2 |
|  | SREBF2 |
|  | ESR2 |
|  | HSD11B2 |
|  | AKR1B10 |
|  | FABP4 |
|  | PTPN6 |
|  | CDC25B |
|  | SLC6A4 |
|  | NPC1L1 |
|  | PTPRF |
|  | PTPN2 |
|  | SCD |
|  | SLC6A2 |
|  | SHBG |
|  | PTPN1 |
|  | FABP5 |
|  | ACP1 |
|  | ACHE |
|  | CYP2C19 |
|  | PPARG |
|  | PREP |
|  | CD81 |
|  | NR1H3 |
|  | BCHE |
|  | CNR1 |
|  | PDE4D |
|  | CYP19A1 |
|  | CHRM2 |
|  | PPARA |
|  | TERT |
|  | ADORA3 |
|  | FABP3 |
|  | MAPK3 |
|  | RORC |
|  | PLA2G1B |
|  | NR1I3 |
|  | PTPN11 |
|  | AR |
|  | CYP51A1 |
|  | ESR1 |
|  | CDC25A |
|  | HSD11B1 |
| MOL006826 | F10 |
|  | HSP90AB1 |
|  | IDO1 |
|  | FOLH1 |
| MOL001040 | HNF4A |
|  | CA2 |
|  | POLB |
|  | MMP13 |
|  | BACE1 |
|  | SIRT2 |
|  | MMP12 |
|  | VEGFA |
|  | PTGER3 |
|  | CES2 |
|  | MMP2 |
|  | AKR1C3 |
|  | ESR2 |
|  | KLK1 |
|  | ALOX12 |
|  | CYP1B1 |
|  | SLC5A2 |
|  | EDNRA |
|  | SHBG |
|  | PTGER4 |
|  | RXRA |
|  | ABCC1 |
|  | ACHE |
|  | PPARG |
|  | PTGS1 |
|  | PTGER1 |
|  | SRC |
|  | SERPINE1 |
|  | CA4 |
|  | HSD17B1 |
|  | CA1 |
|  | PLA2G10 |
|  | BCHE |
|  | PGF |
|  | CYP19A1 |
|  | ERN1 |
|  | TERT |
|  | PLA2G2A |
|  | GRM2 |
|  | ADORA3 |
|  | HSD17B2 |
|  | HSP90AA1 |
|  | NR1H4 |
|  | CTSK |
|  | CHRNA7 |
|  | ALK |
|  | MAOA |
|  | GRM5 |
|  | CES1 |
|  | ABCG2 |
|  | CA12 |
|  | CBR1 |
|  | CA7 |
|  | ADORA1 |
|  | KLK2 |
|  | PLA2G1B |
|  | TOP1 |
|  | MAOB |
|  | RPS6KA5 |
|  | ESR1 |
|  | PTGER2 |
|  | TAS2R31 |
|  | PLA2G5 |
| MOL001484 | NEK1 |
|  | SYK |
|  | MMP8 |
|  | TRPM8 |
|  | HDAC4 |
|  | CDK4 |
|  | MTOR |
|  | CCND2 |
|  | BRAF |
|  | GSK3B |
|  | CA14 |
|  | TBK1 |
|  | DGAT1 |
|  | CHEK1 |
|  | F3 |
|  | ALOX15 |
|  | CHEK2 |
|  | PGF |
|  | IMPDH2 |
|  | HSD17B3 |
|  | HSD17B2 |
|  | ESRRB |
|  | KDR |
|  | IMPDH1 |
|  | PIM2 |
|  | CCND3 |
|  | PIK3CB |
|  | LNPEP |
|  | RET |
|  | GSTP1 |
|  | TUBB1 |
|  | TNKS2 |
|  | LCK |
|  | VEGFA |
|  | PIK3CG |
|  | MAP3K8 |
|  | RAF1 |
|  | ALOX12 |
|  | PIK3CD |
|  | MIF |
|  | RPS6KA3 |
|  | PTPN1 |
|  | IKBKB |
|  | WEE1 |
|  | SRC |
|  | GRK2 |
|  | GSTM2 |
|  | ADCY5 |
|  | ESRRA |
|  | ERN1 |
|  | CCND1 |
|  | RPS6KA1 |
|  | TNKS |
|  | PIK3CA |
|  | DYRK1B |
|  | JAK3 |
|  | PARP1 |
|  | TTR |
|  | AR |
|  | ADAM17 |
|  | HDAC2 |
|  | CLK1 |
|  | ESR1 |
|  | MAPKAPK2 |
|  | EZR |
| MOL003542 | AMY1A |
|  | CA2 |
|  | PFKFB3 |
|  | SYK |
|  | BACE1 |
|  | CA3 |
|  | MPG |
|  | MMP2 |
|  | AKR1C3 |
|  | HSP90AB1 |
|  | ESR2 |
|  | AKR1B10 |
|  | FNTB |
|  | CDK1 |
|  | CSNK2A1 |
|  | EGLN1 |
|  | HSP90B1 |
|  | AKT1 |
|  | XDH |
|  | PIK3R1 |
|  | ACHE |
|  | PPARG |
|  | TYR |
|  | GSK3B |
|  | CA4 |
|  | DAPK1 |
|  | HSD17B1 |
|  | BCHE |
|  | ALOX15 |
|  | CYP19A1 |
|  | MCL1 |
|  | PYGL |
|  | HSD17B2 |
|  | F2 |
|  | SLC22A12 |
|  | ABCG2 |
|  | CA12 |
|  | AKR1C2 |
|  | PLA2G1B |
|  | EP300 |
|  | MAPT |
|  | NEK2 |
|  | PTGS2 |
|  | ARG1 |
|  | CA13 |
|  | NAE1 |
|  | TNKS2 |
|  | CFTR |
|  | PLK1 |
|  | ALOX12 |
|  | CYP1B1 |
|  | ALPL |
|  | PTPRS |
|  | PDE5A |
|  | TOP2A |
|  | PDE10A |
|  | EGFR |
|  | FNTA |
|  | PTPN1 |
|  | APEX1 |
|  | ABCC1 |
|  | GPR35 |
|  | MMP3 |
|  | NOX4 |
|  | AKR1C1 |
|  | MYLK |
|  | ABCB1 |
|  | ESRRA |
|  | AHR |
|  | DHFR |
|  | AVPR2 |
|  | GCGR |
|  | ADORA3 |
|  | AKR1C4 |
|  | HSP90AA1 |
|  | CA5A |
|  | TNKS |
|  | FLT3 |
|  | MAOA |
|  | ALK |
|  | MPO |
|  | ALOX5 |
|  | CA6 |
|  | MMP9 |
|  | NEK6 |
|  | TTR |
|  | CXCR1 |
|  | CCR4 |
|  | AKR1B1 |
|  | ESR1 |
|  | ERBB2 |
|  | KDM4E |
| MOL003627 | SLC6A3 |
|  | DRD4 |
|  | HTR1A |
|  | SRD5A1 |
|  | SRD5A2 |
|  | CYP2D6 |
|  | DRD3 |
|  | CHRM4 |
|  | SLC6A2 |
|  | HTR1B |
|  | CHRNA4 |
|  | PRMT3 |
|  | HTR3A |
|  | KISS1R |
|  | KCNH2 |
|  | CHRM1 |
|  | ACHE |
|  | PARP1 |
|  | CYP2C19 |
|  | CHRNB2 |
|  | AR |
|  | HTR1D |
|  | CHRM5 |
|  | CYP2C9 |
|  | ADRA2B |
|  | BCHE |
|  | CHRM3 |
| MOL003648 | ADAM17 |
|  | CA2 |
|  | MMP8 |
|  | ATP4A |
|  | HDAC4 |
|  | HCK |
|  | CDK1 |
|  | CDK4 |
|  | MTOR |
|  | ROCK1 |
|  | CCND2 |
|  | BRAF |
|  | ATP4B |
|  | TYMS |
|  | CCNB3 |
|  | GSK3B |
|  | PIM3 |
|  | PLAA |
|  | GUSB |
|  | CA14 |
|  | CHEK1 |
|  | DGAT1 |
|  | KDM1A |
|  | MAPK1 |
|  | ALOX15 |
|  | PGF |
|  | HSD17B3 |
|  | CDK5R1 |
|  | KDR |
|  | PIM2 |
|  | EPHB4 |
|  | CCND3 |
|  | PIK3CB |
|  | LNPEP |
|  | RET |
|  | ANPEP |
|  | TUBB1 |
|  | GRM4 |
|  | LCK |
|  | VEGFA |
|  | PIK3CG |
|  | MAP3K8 |
|  | RAF1 |
|  | ALOX12 |
|  | CCNB1 |
|  | TUBB3 |
|  | PIK3CD |
|  | ALPL |
|  | COMT |
|  | DAO |
|  | MKNK1 |
|  | ADORA2A |
|  | WEE1 |
|  | ALPG |
|  | CDK2 |
|  | GRK2 |
|  | ADRA1D |
|  | ABCB1 |
|  | ERN1 |
|  | CDK5 |
|  | CCND1 |
|  | ABL1 |
|  | RPS6KA1 |
|  | PIK3CA |
|  | CSNK1G1 |
|  | PI4KB |
|  | PRKDC |
|  | ADORA2B |
|  | CCNB2 |
|  | HDAC5 |
|  | JAK3 |
|  | PARP1 |
|  | TTR |
|  | CXCR1 |
|  | HDAC2 |
|  | MAPKAPK2 |
|  | HDAC7 |
| MOL003673 | EP300 |
|  | PFKFB3 |
|  | HSP90AB1 |
|  | ESR2 |
|  | ALOX12 |
|  | CDK4 |
|  | FNTB |
|  | HTR2C |
|  | PDE5A |
|  | MIF |
|  | PDE10A |
|  | EGLN1 |
|  | SLC6A2 |
|  | ADORA2A |
|  | EGFR |
|  | FNTA |
|  | HSP90B1 |
|  | PTPN1 |
|  | AKT1 |
|  | SIRT1 |
|  | XDH |
|  | ACHE |
|  | TYR |
|  | F10 |
|  | MGAM |
|  | CA4 |
|  | NOX4 |
|  | HSD17B1 |
|  | ROCK2 |
|  | ALOX15 |
|  | ABCB1 |
|  | HTR2A |
|  | ESRRA |
|  | CYP19A1 |
|  | CCND1 |
|  | HSP90AA1 |
|  | HSD17B2 |
|  | RELA |
|  | ESRRB |
|  | F2 |
|  | MAOA |
|  | ABCG2 |
|  | TBXAS1 |
|  | CA12 |
|  | CA7 |
|  | ADORA1 |
|  | ALDH2 |
|  | CCR4 |
|  | IL2 |
|  | ESR1 |
|  | NOS2 |
| MOL003680 | CHRNA3 |
|  | FAAH |
|  | DPP8 |
|  | MMP8 |
|  | EPHX2 |
|  | HDAC11 |
|  | SRD5A1 |
|  | EPHX1 |
|  | SRD5A2 |
|  | PAOX |
|  | NAAA |
|  | CHRM4 |
|  | DPP7 |
|  | CHRNB4 |
|  | MME |
|  | CHRNA4 |
|  | ADH1C |
|  | DNM1 |
|  | METAP1 |
|  | DPP9 |
|  | CHRM1 |
|  | HDAC8 |
|  | DPP4 |
|  | CHRNB2 |
|  | NCOR2 |
|  | SLC6A3 |
|  | ITGB3 |
|  | CHRM5 |
|  | SLC47A1 |
|  | CHRM3 |
|  | BCHE |
|  | RNPEP |
|  | HDAC6 |
|  | SIGMAR1 |
|  | DRD2 |
|  | CHRM2 |
|  | HTR3B |
|  | CTSK |
|  | CHRNA7 |
|  | GRM5 |
|  | HTR1B |
|  | PRMT3 |
|  | HTR3A |
|  | HSD17B7 |
|  | PARP1 |
|  | DNPEP |
|  | SLC22A2 |
|  | DRD1 |
|  | ITGA2B |
|  | CTSB |
|  | HTR1D |
|  | HDAC2 |
|  | ADH1A |
|  | HDAC10 |
|  | HDAC3 |
|  | CTSL |
|  | HSD11B1 |
| MOL004941 | MMP2 |
|  | CA2 |
|  | BACE1 |
|  | CA3 |
|  | PTGER3 |
|  | AKR1C3 |
|  | ESR2 |
|  | CDK4 |
|  | CDK1 |
|  | AURKB |
|  | PNMT |
|  | ROCK1 |
|  | SHBG |
|  | RXRA |
|  | ACHE |
|  | CCNB3 |
|  | GSK3B |
|  | PPARG |
|  | PTGS1 |
|  | PDPK1 |
|  | CHEK1 |
|  | SERPINE1 |
|  | CA4 |
|  | HSD17B1 |
|  | HNF4A |
|  | PLA2G10 |
|  | DCTPP1 |
|  | BCHE |
|  | PGF |
|  | CYP19A1 |
|  | HSD17B3 |
|  | CDK5R1 |
|  | CISD1 |
|  | PRKACA |
|  | CES1 |
|  | GRM5 |
|  | CA9 |
|  | ABCG2 |
|  | CA12 |
|  | PLA2G1B |
|  | LNPEP |
|  | TAS2R31 |
|  | CA13 |
|  | PLA2G5 |
|  | MMP13 |
|  | POLB |
|  | GRM4 |
|  | MMP12 |
|  | VEGFA |
|  | CES2 |
|  | PIK3CG |
|  | KLK1 |
|  | CCNB1 |
|  | AURKA |
|  | APP |
|  | CYP1B1 |
|  | ALPL |
|  | SLC5A2 |
|  | DAO |
|  | RPS6KA3 |
|  | MME |
|  | EDNRA |
|  | IGF1R |
|  | ABCC1 |
|  | EEF2K |
|  | WEE1 |
|  | SRC |
|  | PTGER1 |
|  | MMP3 |
|  | MET |
|  | CDK2 |
|  | ALPG |
|  | GRK2 |
|  | CA1 |
|  | CCNE1 |
|  | ERN1 |
|  | GCGR |
|  | CDK5 |
|  | PLA2G2A |
|  | GRM2 |
|  | ADORA3 |
|  | CA5B |
|  | CA5A |
|  | ABL1 |
|  | MAOA |
|  | ALK |
|  | CCNB2 |
|  | CA7 |
|  | CBR1 |
|  | RPS6KB1 |
|  | ADORA1 |
|  | CA6 |
|  | KLK2 |
|  | CCNE2 |
|  | MAOB |
|  | ESR1 |
|  | RPS6KA5 |
|  | QDPR |
| MOL005100 | PIK3CA |
|  | PGD |
|  | CA2 |
|  | MMP8 |
|  | SYK |
|  | BACE1 |
|  | CA3 |
|  | ECE1 |
|  | KIT |
|  | MMP2 |
|  | AKR1C3 |
|  | ESR2 |
|  | FFAR1 |
|  | MTOR |
|  | ROCK1 |
|  | SHBG |
|  | RXRA |
|  | ACHE |
|  | PPARG |
|  | PIM3 |
|  | GSK3B |
|  | GUSB |
|  | PTGS1 |
|  | CHEK1 |
|  | FUT4 |
|  | SERPINE1 |
|  | CA4 |
|  | HSD17B1 |
|  | PLA2G10 |
|  | MAPK1 |
|  | BCHE |
|  | CYP19A1 |
|  | FUT7 |
|  | BCL2 |
|  | TERT |
|  | HSD17B2 |
|  | KDR |
|  | PIM2 |
|  | CES1 |
|  | GRM5 |
|  | DNMT1 |
|  | CA9 |
|  | ABCG2 |
|  | CA12 |
|  | STAT1 |
|  | PDGFRB |
|  | PLA2G1B |
|  | RET |
|  | TAS2R31 |
|  | CA13 |
|  | PLA2G5 |
|  | MMP13 |
|  | POLB |
|  | MMP14 |
|  | MMP12 |
|  | CES2 |
|  | PIK3CG |
|  | KLK1 |
|  | ALOX12 |
|  | CYP1B1 |
|  | APP |
|  | ALPL |
|  | EDNRA |
|  | IKBKB |
|  | ABCC1 |
|  | IGF1R |
|  | SRC |
|  | MMP3 |
|  | FGFR1 |
|  | MET |
|  | ALPG |
|  | CA1 |
|  | MYLK |
|  | DYRK1A |
|  | ABCB1 |
|  | ADCY5 |
|  | ERN1 |
|  | PLA2G2A |
|  | ADORA3 |
|  | GRM2 |
|  | TDP1 |
|  | CA5B |
|  | CA5A |
|  | CHRNA7 |
|  | RPS6KA1 |
|  | ST3GAL3 |
|  | CSNK1G1 |
|  | ODC1 |
|  | PIM1 |
|  | CBR1 |
|  | CA7 |
|  | KLK2 |
|  | ADORA1 |
|  | CA6 |
|  | MMP9 |
|  | HSD17B14 |
|  | TOP1 |
|  | MAOB |
|  | ESR1 |
|  | MAPKAPK2 |
| MOL005944 | PRMT3 |
|  | FAAH |
|  | DPP8 |
|  | MMP8 |
|  | EPHX2 |
|  | HDAC11 |
|  | SRD5A1 |
|  | EPHX1 |
|  | SRD5A2 |
|  | PAOX |
|  | NAAA |
|  | CHRM4 |
|  | DPP7 |
|  | CHRNB4 |
|  | MME |
|  | CHRNA4 |
|  | ADH1C |
|  | DNM1 |
|  | METAP1 |
|  | DPP9 |
|  | CHRM1 |
|  | HDAC8 |
|  | DPP4 |
|  | CHRNB2 |
|  | NCOR2 |
|  | SLC6A3 |
|  | ITGB3 |
|  | CHRM5 |
|  | CHRM3 |
|  | BCHE |
|  | RNPEP |
|  | HDAC6 |
|  | SIGMAR1 |
|  | CHRM2 |
|  | CHRNA7 |
|  | CTSK |
|  | GRM5 |
|  | HTR1B |
|  | HTR3A |
|  | HSD17B7 |
|  | PARP1 |
|  | DNPEP |
|  | DRD1 |
|  | HTR1D |
|  | ITGA2B |
|  | CHRNA3 |
|  | CTSB |
|  | HDAC2 |
|  | ADH1A |
|  | HDAC10 |
|  | HDAC3 |
|  | CTSL |
|  | HSD11B1 |
| MOL006561 | PLA2G5 |
|  | CHRNA5 |
|  | HTR1A |
|  | PTGER3 |
|  | HTR1F |
|  | HTR2C |
|  | EPHX1 |
|  | SRD5A2 |
|  | ADRA1A |
|  | SLC6A4 |
|  | GRM6 |
|  | SLC6A2 |
|  | CHRNB4 |
|  | CHRNA4 |
|  | MME |
|  | DNM1 |
|  | PTGER4 |
|  | KCNH2 |
|  | GRM8 |
|  | DPP4 |
|  | CHRNB2 |
|  | GBA |
|  | ITGB3 |
|  | CHRNB3 |
|  | ADRB3 |
|  | KDM1A |
|  | REN |
|  | PLA2G10 |
|  | ROCK2 |
|  | BCHE |
|  | SLC6A9 |
|  | ADRA1D |
|  | RNPEP |
|  | ABCB1 |
|  | FKBP1A |
|  | DRD4 |
|  | HSD17B3 |
|  | CHRNA2 |
|  | GRM2 |
|  | FUCA1 |
|  | CHRNA6 |
|  | HRH4 |
|  | CHRNA7 |
|  | PRKCA |
|  | DRD3 |
|  | GBA2 |
|  | PLA2G2C |
|  | HTR3A |
|  | ADRB1 |
|  | PARP1 |
|  | GRM3 |
|  | AR |
|  | DNPEP |
|  | CYP51A1 |
|  | DRD1 |
|  | ADRB2 |
|  | CHRNA3 |
|  | ITGA2B |
|  | HTR1D |
|  | PTGER2 |
|  | HSD11B1 |
| MOL006562 | HTR2C |
|  | OPRD1 |
|  | HTR1A |
|  | SRD5A2 |
|  | SLC6A4 |
|  | CYP2D6 |
|  | CHRM4 |
|  | SLC6A2 |
|  | CHRNB4 |
|  | CHRNA4 |
|  | OPRK1 |
|  | KCNH2 |
|  | KISS1R |
|  | CHRM1 |
|  | DPP4 |
|  | SLC6A3 |
|  | CHRM5 |
|  | DRD5 |
|  | SLC47A1 |
|  | CHRM3 |
|  | HTR2A |
|  | SIGMAR1 |
|  | DRD4 |
|  | CHRM2 |
|  | DRD2 |
|  | HTR3B |
|  | DRD3 |
|  | HTR1B |
|  | PRMT3 |
|  | HTR3A |
|  | PARP1 |
|  | HRH3 |
|  | HTR2B |
|  | DRD1 |
|  | SLC22A2 |
|  | CHRNA3 |
|  | OPRM1 |
| MOL006563 | H1F0 |
|  | BACE1 |
|  | PTGER3 |
|  | HTR1F |
|  | CYP2D6 |
|  | UGCG |
|  | ROCK1 |
|  | SLC6A2 |
|  | CHRNA4 |
|  | DNM1 |
|  | GRM8 |
|  | CHRNB2 |
|  | ADRA2C |
|  | GBA |
|  | HRH1 |
|  | ADRB3 |
|  | SLC18A2 |
|  | HTR5A |
|  | PLA2G10 |
|  | ROCK2 |
|  | BCHE |
|  | SLC6A9 |
|  | RNPEP |
|  | HTR6 |
|  | FKBP1A |
|  | DRD4 |
|  | HSD17B3 |
|  | FUCA1 |
|  | HRH2 |
|  | KCNK2 |
|  | PRKCA |
|  | DRD3 |
|  | HTR1B |
|  | PLA2G2C |
|  | ADRB1 |
|  | GRM3 |
|  | CYP51A1 |
|  | ADRB2 |
|  | ADRA2B |
|  | PLA2G5 |
|  | CHRNA5 |
|  | SCN5A |
|  | HTR1A |
|  | ADRA2A |
|  | HTR2C |
|  | KCNA5 |
|  | SRD5A2 |
|  | SLC6A4 |
|  | ADRA1A |
|  | GRM6 |
|  | PDE10A |
|  | CHRNB4 |
|  | MME |
|  | PTGER4 |
|  | KCNH2 |
|  | DPP4 |
|  | ITGB3 |
|  | DRD5 |
|  | CHRNB3 |
|  | REN |
|  | ADRA1D |
|  | UTS2R |
|  | ABCB1 |
|  | CHRNA2 |
|  | GRM2 |
|  | TACR1 |
|  | CHRNA6 |
|  | CHRNA7 |
|  | HRH4 |
|  | GBA2 |
|  | HTR3A |
|  | PARP1 |
|  | ADRA1B |
|  | HTR2B |
|  | DNPEP |
|  | AR |
|  | JAK1 |
|  | DRD1 |
|  | ITGA2B |
|  | CHRNA3 |
|  | HTR1D |
|  | CTSC |
|  | PTGER2 |
|  | HSD11B1 |
| MOL006564 | HTR1F |
|  | FAAH |
|  | DPP8 |
|  | MMP8 |
|  | EPHX2 |
|  | HDAC11 |
|  | SRD5A1 |
|  | EPHX1 |
|  | SRD5A2 |
|  | PAOX |
|  | NAAA |
|  | CHRM4 |
|  | DPP7 |
|  | CHRNB4 |
|  | MME |
|  | CHRNA4 |
|  | ADH1C |
|  | DNM1 |
|  | METAP1 |
|  | DPP9 |
|  | CHRM1 |
|  | HDAC8 |
|  | DPP4 |
|  | CHRNB2 |
|  | NCOR2 |
|  | SLC6A3 |
|  | ITGB3 |
|  | CHRM5 |
|  | SLC47A1 |
|  | CHRM3 |
|  | BCHE |
|  | RNPEP |
|  | HDAC6 |
|  | SIGMAR1 |
|  | DRD2 |
|  | CHRM2 |
|  | HTR3B |
|  | CTSK |
|  | CHRNA7 |
|  | GRM5 |
|  | HTR1B |
|  | PRMT3 |
|  | HTR3A |
|  | HSD17B7 |
|  | PARP1 |
|  | HTR2B |
|  | DNPEP |
|  | SLC22A2 |
|  | DRD1 |
|  | ITGA2B |
|  | CHRNA3 |
|  | CTSB |
|  | HTR1D |
|  | HDAC2 |
|  | ADH1A |
|  | HDAC10 |
|  | HDAC3 |
|  | CTSL |
|  | HSD11B1 |
| MOL006565 | CHRM3 |
|  | HDAC1 |
|  | FAAH |
|  | DPP8 |
|  | MMP8 |
|  | EPHX2 |
|  | HDAC11 |
|  | SRD5A1 |
|  | EPHX1 |
|  | SCARB1 |
|  | SRD5A2 |
|  | PAOX |
|  | NAAA |
|  | CHRM4 |
|  | DPP7 |
|  | CHRNB4 |
|  | MME |
|  | CHRNA4 |
|  | ADH1C |
|  | DNM1 |
|  | METAP1 |
|  | DPP9 |
|  | CHRM1 |
|  | HDAC8 |
|  | DPP4 |
|  | CHRNB2 |
|  | NCOR2 |
|  | SLC6A3 |
|  | ITGB3 |
|  | CHRM5 |
|  | REN |
|  | BCHE |
|  | RNPEP |
|  | HDAC6 |
|  | SIGMAR1 |
|  | CHRM2 |
|  | CTSK |
|  | CHRNA7 |
|  | GRM5 |
|  | HTR1B |
|  | HTR3A |
|  | HSD17B7 |
|  | PARP1 |
|  | DNPEP |
|  | HTR1D |
|  | ITGA2B |
|  | CHRNA3 |
|  | CTSB |
|  | HDAC2 |
|  | ADH1A |
|  | HDAC10 |
|  | HDAC3 |
|  | CTSL |
|  | HSD11B1 |
| MOL006568 | HTR7 |
|  | DRD4 |
|  | OPRD1 |
|  | HTR1A |
|  | SRD5A1 |
|  | SRD5A2 |
|  | SLC6A4 |
|  | CYP2D6 |
|  | DRD3 |
|  | CHRM4 |
|  | SLC6A2 |
|  | HTR1B |
|  | OPRL1 |
|  | CHRNA4 |
|  | PRMT3 |
|  | HTR3A |
|  | KISS1R |
|  | KCNH2 |
|  | CHRM1 |
|  | ACHE |
|  | PARP1 |
|  | CYP2C19 |
|  | CHRNB2 |
|  | AR |
|  | HTR1D |
|  | CHRM5 |
|  | CYP2C9 |
|  | ADRA2B |
|  | BCHE |
|  | CHRM3 |
| MOL006569 | PLA2G5 |
|  | CHRNA5 |
|  | PTGER3 |
|  | EPHX1 |
|  | SRD5A2 |
|  | SLC6A4 |
|  | GRM6 |
|  | UGCG |
|  | SLC6A2 |
|  | CHRNB4 |
|  | CHRNA4 |
|  | MME |
|  | DNM1 |
|  | PTGER4 |
|  | KCNH2 |
|  | GRM8 |
|  | DPP4 |
|  | CHRNB2 |
|  | GBA |
|  | ITGB3 |
|  | DRD5 |
|  | PLA2G10 |
|  | REN |
|  | SLC6A9 |
|  | RNPEP |
|  | FKBP1A |
|  | DRD4 |
|  | HSD17B3 |
|  | CHRNA2 |
|  | FUCA1 |
|  | GRM2 |
|  | CHRNA7 |
|  | LAP3 |
|  | PRKCA |
|  | DRD3 |
|  | GBA2 |
|  | PLA2G2C |
|  | HTR3A |
|  | GRM3 |
|  | JAK1 |
|  | DNPEP |
|  | AR |
|  | DRD1 |
|  | HTR1D |
|  | ITGA2B |
|  | CHRNA3 |
|  | PTGER2 |
|  | HSD11B1 |
| MOL006570 | CTSC |
|  | DPP8 |
|  | CHRNA5 |
|  | SCN5A |
|  | HTR1A |
|  | HTR1F |
|  | HTR2C |
|  | ADRA2A |
|  | KCNA5 |
|  | CYP2D6 |
|  | ADRA1A |
|  | SLC6A4 |
|  | ROCK1 |
|  | PDE10A |
|  | SLC6A2 |
|  | CHRNB4 |
|  | SHBG |
|  | CHRNA4 |
|  | OPRK1 |
|  | KISS1R |
|  | KCNH2 |
|  | DPP9 |
|  | CYP2C19 |
|  | CHRNB2 |
|  | HTR7 |
|  | HRH1 |
|  | CYP2C9 |
|  | CHRNB3 |
|  | ADRB3 |
|  | HTR5A |
|  | ROCK2 |
|  | BCHE |
|  | UTS2R |
|  | SLC6A9 |
|  | ABCB1 |
|  | DRD4 |
|  | HTR2A |
|  | TNNI3 |
|  | CHRNA2 |
|  | DRD2 |
|  | TNNT2 |
|  | CHRNA6 |
|  | HRH4 |
|  | DRD3 |
|  | HTR1B |
|  | OPRL1 |
|  | FAP |
|  | TNNC1 |
|  | HTR3A |
|  | ADRB1 |
|  | ADRA1B |
|  | PARP1 |
|  | JAK1 |
|  | AR |
|  | CYP51A1 |
|  | DRD1 |
|  | CHRNA3 |
|  | HTR1D |
|  | ADRB2 |
|  | ADRA2B |
| MOL006571 | HTR3A |
|  | DRD2 |
|  | CHRNA1 |
|  | CHRNA7 |
|  | SRD5A2 |
|  | CYP2D6 |
|  | DRD3 |
|  | CHRNB4 |
|  | CHRNA4 |
|  | PRMT3 |
|  | KISS1R |
|  | PARP1 |
|  | CHRNB2 |
|  | HTR2B |
|  | CHRNG |
|  | HTR7 |
|  | SLC6A3 |
|  | CHRNA3 |
|  | ADRA2B |
|  | CHRNB1 |
|  | CHRND |
| MOL006573 | DRD1 |
|  | DRD4 |
|  | OPRD1 |
|  | HTR1A |
|  | SRD5A1 |
|  | SRD5A2 |
|  | CYP2D6 |
|  | SLC6A4 |
|  | DRD3 |
|  | CHRM4 |
|  | SLC6A2 |
|  | HTR1B |
|  | OPRL1 |
|  | CHRNA4 |
|  | PRMT3 |
|  | HTR3A |
|  | KISS1R |
|  | KCNH2 |
|  | CHRM1 |
|  | ACHE |
|  | PARP1 |
|  | CYP2C19 |
|  | CHRNB2 |
|  | HTR7 |
|  | AR |
|  | HTR1D |
|  | CHRM5 |
|  | CYP2C9 |
|  | ADRA2B |
|  | BCHE |
|  | CHRM3 |
| MOL006582 | SLC18A2 |
|  | PTGER3 |
|  | HTR1F |
|  | GRIN1 |
|  | UGCG |
|  | SLC6A2 |
|  | CHRNA4 |
|  | DNM1 |
|  | GRIN2B |
|  | GRM8 |
|  | CHRNB2 |
|  | GBA |
|  | ADRB3 |
|  | PLA2G10 |
|  | RNPEP |
|  | DRD4 |
|  | HSD17B3 |
|  | FUCA1 |
|  | TNNT2 |
|  | PRKCA |
|  | DRD3 |
|  | PLA2G2C |
|  | TNNC1 |
|  | TRPV3 |
|  | ADRB1 |
|  | GRM3 |
|  | ADRB2 |
|  | PLA2G5 |
|  | HTR1A |
|  | EPHX1 |
|  | HTR2C |
|  | SRD5A2 |
|  | KCNA5 |
|  | SLC6A4 |
|  | GRM6 |
|  | PDE5A |
|  | PDE10A |
|  | CHRNB4 |
|  | MME |
|  | PTGER4 |
|  | KCNH2 |
|  | DPP4 |
|  | ITGB3 |
|  | REN |
|  | ABCB1 |
|  | CNR1 |
|  | MTAP |
|  | TNNI3 |
|  | PLA2G2A |
|  | CNR2 |
|  | PNP |
|  | GRM2 |
|  | CHRNA7 |
|  | NR1H4 |
|  | GBA2 |
|  | OPRL1 |
|  | PDE11A |
|  | HTR3A |
|  | KCNJ1 |
|  | DUSP3 |
|  | DNPEP |
|  | AR |
|  | HTR1D |
|  | CHRNA3 |
|  | ITGA2B |
|  | PTGER2 |
| MOL006596 | NR3C2 |
|  | SCN9A |
|  | SYK |
|  | HPGD |
|  | PIK3CG |
|  | ESR2 |
|  | CDK1 |
|  | AGTR1 |
|  | ALPL |
|  | MTOR |
|  | PTPN1 |
|  | P2RX3 |
|  | WEE1 |
|  | PLAA |
|  | MAP3K7 |
|  | CHEK1 |
|  | CDK2 |
|  | ROCK2 |
|  | BRD4 |
|  | FKBP1A |
|  | PDE4D |
|  | PYGL |
|  | ITK |
|  | ALOX5AP |
|  | TAB1 |
|  | TOP1 |
|  | AR |
|  | ADAM17 |
|  | FCER2 |
|  | RET |
|  | HDAC2 |
|  | ESR1 |
|  | FLT1 |
| MOL003347 | NR1I2 |
|  | PTGES |
|  | ITGAL |
|  | PTGS2 |
|  | CYP19A1 |
|  | ALOX5 |
|  | NOS2 |
|  | CCKBR |
|  | NR3C2 |
|  | HSD11B2 |
| MOL006604 | SERPINE1 |
|  | MMP8 |
|  | BACE1 |
|  | ESR2 |
|  | AKR1B10 |
|  | NAAA |
|  | MTOR |
|  | BRAF |
|  | SHBG |
|  | P2RX3 |
|  | RXRA |
|  | ACHE |
|  | PPARG |
|  | PTGS1 |
|  | CHEK1 |
|  | CA4 |
|  | HSD17B1 |
|  | PLA2G10 |
|  | BCHE |
|  | CYP19A1 |
|  | SPHK1 |
|  | MMP16 |
|  | HSD17B2 |
|  | ESRRB |
|  | CES1 |
|  | MMP1 |
|  | ABCG2 |
|  | CA12 |
|  | PLA2G1B |
|  | CDK3 |
|  | TAS2R31 |
|  | TNF |
|  | PLA2G5 |
|  | POLB |
|  | MMP13 |
|  | MMP12 |
|  | CTSD |
|  | LCK |
|  | CES2 |
|  | KLK1 |
|  | MDM2 |
|  | MMP15 |
|  | CYP1B1 |
|  | SLC5A2 |
|  | PDK1 |
|  | SPHK2 |
|  | PTPN1 |
|  | ABCC1 |
|  | WEE1 |
|  | CDK2 |
|  | CCNE1 |
|  | ESRRA |
|  | ADORA3 |
|  | HSP90AA1 |
|  | CTSK |
|  | ODC1 |
|  | ADORA2B |
|  | CBR1 |
|  | CA7 |
|  | ADORA1 |
|  | KLK2 |
|  | ALOX5 |
|  | AR |
|  | ADAM17 |
|  | MAOB |
|  | PDE7A |
|  | ESR1 |
|  | AKR1B1 |
|  | CTSL |
|  | MMP26 |
| MOL006613 | ESRRB |
|  | FLT4 |
|  | CA2 |
|  | TRPM8 |
|  | MMP8 |
|  | MMP2 |
|  | ESR2 |
|  | HDAC4 |
|  | CDK1 |
|  | CDK4 |
|  | PARP2 |
|  | BRAF |
|  | DNM1 |
|  | PIM3 |
|  | GSK3B |
|  | PTGS1 |
|  | PDGFRA |
|  | GUSB |
|  | CHEK1 |
|  | F3 |
|  | KDM1A |
|  | ALOX15 |
|  | CYP19A1 |
|  | HSD17B3 |
|  | IMPDH2 |
|  | CISD1 |
|  | MMP16 |
|  | HSD17B2 |
|  | KDR |
|  | IMPDH1 |
|  | PIM2 |
|  | CA9 |
|  | PDGFRB |
|  | CDK3 |
|  | LNPEP |
|  | TNKS2 |
|  | MMP14 |
|  | GRM4 |
|  | MAP3K8 |
|  | ALOX12 |
|  | CCNB1 |
|  | PIK3CD |
|  | AURKA |
|  | PDE5A |
|  | ADORA2A |
|  | PTPN1 |
|  | HDAC9 |
|  | YWHAG |
|  | WEE1 |
|  | SRC |
|  | MET |
|  | CDK2 |
|  | GRK2 |
|  | CA1 |
|  | CCNE1 |
|  | DYRK1A |
|  | ESRRA |
|  | ERN1 |
|  | BCL2L1 |
|  | CHRNA7 |
|  | TNKS |
|  | SGK1 |
|  | ALOX15B |
|  | DYRK1B |
|  | HDAC5 |
|  | MMP9 |
|  | PARP1 |
|  | TOP1 |
|  | TTR |
|  | AR |
|  | ADAM17 |
|  | HDAC2 |
|  | RPS6KA5 |
|  | AKR1B1 |
|  | ESR1 |
|  | CLK1 |
|  | HDAC7 |
|  | HDAC3 |
| MOL006623 | HSD17B3 |
|  | MMP8 |
|  | PCNA |
|  | SYK |
|  | BACE1 |
|  | MAP3K12 |
|  | ESR2 |
|  | AKR1B10 |
|  | VCP |
|  | CDK1 |
|  | ADAMTS4 |
|  | WNT3A |
|  | ACVRL1 |
|  | MTOR |
|  | BRAF |
|  | SHBG |
|  | RXRA |
|  | PIK3R1 |
|  | CHRM1 |
|  | ACHE |
|  | CCNB3 |
|  | PIM3 |
|  | PPARG |
|  | MERTK |
|  | PTGS1 |
|  | SORD |
|  | PDPK1 |
|  | CHEK1 |
|  | SERPINE1 |
|  | CA4 |
|  | HSD17B1 |
|  | PLA2G10 |
|  | GRM1 |
|  | BCHE |
|  | FKBP1A |
|  | CYP19A1 |
|  | MCL1 |
|  | ITK |
|  | TXK |
|  | MAP3K14 |
|  | F2 |
|  | PIM2 |
|  | CES1 |
|  | XIAP |
|  | MMP1 |
|  | ABCG2 |
|  | CA12 |
|  | ADAMTS1 |
|  | TYMP |
|  | PIK3CB |
|  | PLA2G1B |
|  | MMP7 |
|  | LNPEP |
|  | CTSB |
|  | TAS2R31 |
|  | HDAC10 |
|  | PLA2G5 |
|  | POLB |
|  | MMP13 |
|  | TNKS2 |
|  | MMP12 |
|  | CES2 |
|  | STAT6 |
|  | PIK3CG |
|  | CCNB1 |
|  | PIK3CD |
|  | AURKA |
|  | CYP1B1 |
|  | SLC5A2 |
|  | PDK1 |
|  | PDE10A |
|  | PTPN1 |
|  | ROS1 |
|  | IGF1R |
|  | ABCC1 |
|  | WEE1 |
|  | ADCY1 |
|  | NCOR2 |
|  | MMP3 |
|  | ERN1 |
|  | PDE4B |
|  | ADORA3 |
|  | BIRC2 |
|  | ADAMTS5 |
|  | HSP90AA1 |
|  | MAPK3 |
|  | PIK3CA |
|  | PRKDC |
|  | PIM1 |
|  | CCNB2 |
|  | CA7 |
|  | CBR1 |
|  | ADORA1 |
|  | KLK2 |
|  | HDAC5 |
|  | PAK4 |
|  | MAPK10 |
|  | ADAM17 |
|  | FCER2 |
|  | MAOB |
|  | ESR1 |
|  | AKR1B1 |
|  | HDAC3 |
| MOL006626 | HTR7 |
|  | MMP8 |
|  | SYK |
|  | BACE1 |
|  | ECE1 |
|  | AKR1C3 |
|  | ESR2 |
|  | AKR1B10 |
|  | CDK1 |
|  | BRAF |
|  | SHBG |
|  | OPRK1 |
|  | DNM1 |
|  | RXRA |
|  | ACHE |
|  | PIM3 |
|  | GSK3B |
|  | PPARG |
|  | PTGS1 |
|  | CCNA1 |
|  | CHEK1 |
|  | F3 |
|  | SERPINE1 |
|  | CA4 |
|  | HSD17B1 |
|  | PLA2G10 |
|  | BCHE |
|  | DRD4 |
|  | CYP19A1 |
|  | SPHK1 |
|  | HSD17B2 |
|  | PIM2 |
|  | DRD3 |
|  | CES1 |
|  | NTRK1 |
|  | MMP1 |
|  | EPHB4 |
|  | ABCG2 |
|  | CA12 |
|  | PLA2G1B |
|  | CDK3 |
|  | TAS2R31 |
|  | PLA2G5 |
|  | MMP13 |
|  | POLB |
|  | INSR |
|  | OPRD1 |
|  | MMP12 |
|  | CES2 |
|  | MDM2 |
|  | RAF1 |
|  | KLK1 |
|  | CCNB1 |
|  | AURKA |
|  | CYP1B1 |
|  | SLC5A2 |
|  | PDK1 |
|  | SPHK2 |
|  | PTPN1 |
|  | ABCC1 |
|  | IGF1R |
|  | WEE1 |
|  | NCOR2 |
|  | MMP3 |
|  | DRD5 |
|  | NOX4 |
|  | CDK2 |
|  | CCNE1 |
|  | ERN1 |
|  | PLA2G2A |
|  | ADORA3 |
|  | CHRNA7 |
|  | ODC1 |
|  | SGK1 |
|  | PIM1 |
|  | CCNA2 |
|  | ADORA2B |
|  | CBR1 |
|  | CA7 |
|  | RPS6KB1 |
|  | ADORA1 |
|  | KLK2 |
|  | ALOX5 |
|  | ADAM17 |
|  | DRD1 |
|  | MAOB |
|  | PDE7A |
|  | HDAC2 |
|  | AKR1B1 |
|  | ESR1 |
|  | HDAC3 |
|  | CTSL |
|  | OPRM1 |
| MOL006627 | DRD1 |
|  | SIGMAR1 |
|  | HTR3B |
|  | SLC6A4 |
|  | CYP2D6 |
|  | CHRM4 |
|  | SLC6A2 |
|  | HTR1B |
|  | OPRL1 |
|  | CHRNA4 |
|  | PRMT3 |
|  | HTR3A |
|  | KISS1R |
|  | PARP1 |
|  | ACHE |
|  | SLC22A2 |
|  | HTR1D |
|  | CHRNA3 |
|  | ADRA2B |
|  | CHRM5 |
|  | SLC47A1 |
| MOL006628 | CHRNA4 |
|  | HDAC1 |
|  | DPP8 |
|  | FAAH |
|  | MMP8 |
|  | EPHX2 |
|  | HDAC11 |
|  | SRD5A1 |
|  | EPHX1 |
|  | SRD5A2 |
|  | PAOX |
|  | NAAA |
|  | DPP7 |
|  | CHRNB4 |
|  | MME |
|  | ADH1C |
|  | DNM1 |
|  | METAP1 |
|  | DPP9 |
|  | HDAC8 |
|  | DPP4 |
|  | CHRNB2 |
|  | NCOR2 |
|  | ITGB3 |
|  | REN |
|  | BCHE |
|  | RNPEP |
|  | HDAC6 |
|  | CHRNA7 |
|  | CTSK |
|  | GRM5 |
|  | HSD17B7 |
|  | DNPEP |
|  | AR |
|  | ITGA2B |
|  | CHRNA3 |
|  | CTSB |
|  | HDAC2 |
|  | ADH1A |
|  | HDAC10 |
|  | CTSL |
|  | HDAC3 |
|  | HSD11B1 |
| MOL006630 | PFKFB3 |
|  | CA2 |
|  | BACE1 |
|  | SYK |
|  | CA3 |
|  | MPG |
|  | AKR1C3 |
|  | MMP2 |
|  | ESR2 |
|  | AKR1B10 |
|  | AURKB |
|  | CDK1 |
|  | PTK2 |
|  | CSNK2A1 |
|  | AKT1 |
|  | NUAK1 |
|  | PIK3R1 |
|  | XDH |
|  | ACHE |
|  | CCNB3 |
|  | TYR |
|  | GSK3B |
|  | CA14 |
|  | GRK6 |
|  | CA4 |
|  | DAPK1 |
|  | HSD17B1 |
|  | ALOX15 |
|  | DRD4 |
|  | CYP19A1 |
|  | PYGL |
|  | CDK5R1 |
|  | TERT |
|  | HSD17B2 |
|  | KDR |
|  | F2 |
|  | SLC22A12 |
|  | CA9 |
|  | ABCG2 |
|  | CA12 |
|  | CDK6 |
|  | AKR1C2 |
|  | PLA2G1B |
|  | AKR1A1 |
|  | GLO1 |
|  | NEK2 |
|  | PTGS2 |
|  | ARG1 |
|  | CA13 |
|  | CAMK2B |
|  | NAE1 |
|  | MMP13 |
|  | TNKS2 |
|  | MMP12 |
|  | CFTR |
|  | PLK1 |
|  | CCNB1 |
|  | ALOX12 |
|  | CYP1B1 |
|  | APP |
|  | PTPRS |
|  | EGFR |
|  | ADORA2A |
|  | ABCC1 |
|  | IGF1R |
|  | PKN1 |
|  | SRC |
|  | MMP3 |
|  | GPR35 |
|  | CD38 |
|  | MET |
|  | NOX4 |
|  | CDK2 |
|  | AKR1C1 |
|  | CA1 |
|  | ABCB1 |
|  | AXL |
|  | ESRRA |
|  | AHR |
|  | CDK5 |
|  | AVPR2 |
|  | AKR1C4 |
|  | CA5A |
|  | TNKS |
|  | AMY1A |
|  | FLT3 |
|  | ALK |
|  | PIM1 |
|  | MAOA |
|  | MPO |
|  | CA7 |
|  | CCNB2 |
|  | CA6 |
|  | ALOX5 |
|  | ADORA1 |
|  | NEK6 |
|  | MMP9 |
|  | PARP1 |
|  | TTR |
|  | TOP1 |
|  | CXCR1 |
|  | AKR1B1 |
|  | ESR1 |
| MOL000456 | HSP90AA1 |
|  | FLT4 |
|  | MMP8 |
|  | PCNA |
|  | PFKFB3 |
|  | ESR2 |
|  | HCK |
|  | GABRA3 |
|  | VCP |
|  | AURKB |
|  | CDK1 |
|  | CDK4 |
|  | GABRB3 |
|  | MTOR |
|  | GABRA5 |
|  | CCND2 |
|  | BRAF |
|  | PTK6 |
|  | GSK3B |
|  | PIM3 |
|  | PDGFRA |
|  | MERTK |
|  | CHEK1 |
|  | ROCK2 |
|  | MAPK1 |
|  | DRD4 |
|  | HSD17B3 |
|  | CDK5R1 |
|  | SNCA |
|  | MCL1 |
|  | ELANE |
|  | HSD17B2 |
|  | PIM2 |
|  | DRD3 |
|  | EPHB4 |
|  | PTAFR |
|  | CCND3 |
|  | PDE3A |
|  | NOX1 |
|  | PDGFRB |
|  | PIK3CB |
|  | EP300 |
|  | RET |
|  | LNPEP |
|  | CTSS |
|  | HDAC1 |
|  | GRM4 |
|  | LCK |
|  | SREBF2 |
|  | PIK3CG |
|  | PLK1 |
|  | DBF4 |
|  | CDC7 |
|  | GABRA1 |
|  | PIK3CD |
|  | AURKA |
|  | COMT |
|  | MIF |
|  | HPGDS |
|  | PDE5A |
|  | PDE10A |
|  | RPS6KA3 |
|  | ADORA2A |
|  | EGFR |
|  | HDAC9 |
|  | PTPN1 |
|  | DPP4 |
|  | WEE1 |
|  | CMA1 |
|  | CRHR1 |
|  | CDK2 |
|  | NOX4 |
|  | BAD |
|  | CCNE1 |
|  | HDAC6 |
|  | TRPV1 |
|  | ERN1 |
|  | PGR |
|  | GCGR |
|  | LTA4H |
|  | PDE4B |
|  | CDK5 |
|  | DRD2 |
|  | S1PR1 |
|  | CCND1 |
|  | ABL1 |
|  | S1PR3 |
|  | HRH4 |
|  | GPBAR1 |
|  | FLT3 |
|  | PRKDC |
|  | ADORA2B |
|  | PIM1 |
|  | PDE3B |
|  | TSPO |
|  | NR3C1 |
|  | CCNE2 |
|  | ALDH2 |
|  | AR |
|  | GABRG2 |
|  | TNNI3K |
|  | HDAC2 |
|  | PDE7A |
|  | ESR1 |
|  | GABRA6 |
|  | ERBB2 |
| MOL006650 | EIF4A1 |
|  | CYP19A1 |
|  | PTGS1 |
| MOL000449 | POLB |
|  | VDR |
|  | SQLE |
|  | PPARD |
|  | CYP17A1 |
|  | HMGCR |
|  | G6PD |
|  | SREBF2 |
|  | ESR2 |
|  | PTPN6 |
|  | PTGES |
|  | SLC6A4 |
|  | NPC1L1 |
|  | PTPN2 |
|  | SLC6A2 |
|  | SHBG |
|  | PTPN1 |
|  | ACHE |
|  | CYP2C19 |
|  | PPARG |
|  | NR1H2 |
|  | PTGER1 |
|  | SERPINA6 |
|  | NR1H3 |
|  | BCHE |
|  | CYP19A1 |
|  | CHRM2 |
|  | PPARA |
|  | UGT2B7 |
|  | RORA |
|  | RORC |
|  | TBXAS1 |
|  | NR1I3 |
|  | AR |
|  | CYP51A1 |
|  | FDFT1 |
|  | ESR1 |
|  | NOS2 |
|  | PTGER2 |
|  | HSD11B1 |
|  | DHCR7 |
| MOL000953 | PREP |
|  | POLB |
|  | SQLE |
|  | VDR |
|  | PPARD |
|  | CYP17A1 |
|  | HMGCR |
|  | CES2 |
|  | G6PD |
|  | SREBF2 |
|  | ESR2 |
|  | GLRA1 |
|  | PTPN6 |
|  | SHH |
|  | PTGES |
|  | SLC6A4 |
|  | NPC1L1 |
|  | PTPN2 |
|  | SLC6A2 |
|  | SHBG |
|  | PTPN1 |
|  | ACHE |
|  | CYP2C19 |
|  | PPARG |
|  | PTGER1 |
|  | NR1H2 |
|  | SERPINA6 |
|  | NR1H3 |
|  | BCHE |
|  | SIGMAR1 |
|  | CYP19A1 |
|  | CHRM2 |
|  | PPARA |
|  | UGT2B7 |
|  | RORA |
|  | RORC |
|  | TBXAS1 |
|  | NR1I3 |
|  | AR |
|  | CYP51A1 |
|  | FDFT1 |
|  | ESR1 |
|  | NOS2 |
|  | PTGER2 |
|  | HSD11B1 |
|  | DHCR7 |
| MOL001645 | HSD17B2 |
|  | PSENEN |
|  | FAAH |
|  | OPRD1 |
|  | PPARD |
|  | HMGCR |
|  | CYP17A1 |
|  | GPR119 |
|  | HSD11B2 |
|  | FABP4 |
|  | CDC7 |
|  | CDK4 |
|  | APP |
|  | EPHX1 |
|  | SOAT1 |
|  | GRIN1 |
|  | PTGES |
|  | ROCK1 |
|  | PRCP |
|  | RBP4 |
|  | ABHD6 |
|  | SHBG |
|  | PTPN1 |
|  | GRIN2B |
|  | APH1A |
|  | ACACB |
|  | SMO |
|  | SRC |
|  | PSEN2 |
|  | DGAT1 |
|  | CALCRL |
|  | AKR1C1 |
|  | F2R |
|  | ROCK2 |
|  | NCSTN |
|  | CNR1 |
|  | ACACA |
|  | CYP19A1 |
|  | SLC5A1 |
|  | SNCA |
|  | CNR2 |
|  | HRH2 |
|  | SSTR4 |
|  | FABP3 |
|  | MGLL |
|  | PIK3CA |
|  | ALOX5AP |
|  | PTAFR |
|  | PORCN |
|  | CDK6 |
|  | P2RY1 |
|  | CFD |
|  | APH1B |
|  | MAPT |
|  | NAMPT |
|  | PSEN1 |
|  | TAS2R31 |
|  | HSD11B1 |
| MOL002776 | CA12 |
|  | CA2 |
|  | ADRA2A |
|  | NQO2 |
|  | RPS6KA3 |
|  | EGFR |
|  | ADORA1 |
|  | XDH |
|  | ACHE |
|  | ALDH2 |
|  | IL2 |
|  | ADRA2C |
|  | FDFT1 |
|  | PTGS2 |
|  | AKR1B1 |
|  | NMUR2 |
|  | NOX4 |
|  | TNF |
| MOL004598 | TERT |
|  | CA2 |
|  | BACE1 |
|  | SYK |
|  | MPG |
|  | CA3 |
|  | AKR1C3 |
|  | ESR2 |
|  | MMP2 |
|  | KIT |
|  | CDK1 |
|  | AURKB |
|  | CSNK2A1 |
|  | PTK2 |
|  | AKT1 |
|  | NUAK1 |
|  | PIK3R1 |
|  | XDH |
|  | ACHE |
|  | PLG |
|  | CCNB3 |
|  | TYR |
|  | GSK3B |
|  | CA14 |
|  | CA4 |
|  | DAPK1 |
|  | ALOX15 |
|  | CYP19A1 |
|  | DRD4 |
|  | PYGL |
|  | MCL1 |
|  | CDK5R1 |
|  | HSD17B2 |
|  | KDR |
|  | F2 |
|  | CA9 |
|  | ABCG2 |
|  | CDK6 |
|  | CA12 |
|  | AKR1C2 |
|  | PLA2G1B |
|  | AKR1A1 |
|  | MAPT |
|  | NEK2 |
|  | PTGS2 |
|  | NOS2 |
|  | ARG1 |
|  | CA13 |
|  | CAMK2B |
|  | INSR |
|  | MMP13 |
|  | OPRD1 |
|  | PIK3CG |
|  | PLK1 |
|  | ALOX12 |
|  | CCNB1 |
|  | CYP1B1 |
|  | APP |
|  | PTPRS |
|  | PDE5A |
|  | TOP2A |
|  | RPS6KA3 |
|  | ADORA2A |
|  | EGFR |
|  | APEX1 |
|  | ABCC1 |
|  | IGF1R |
|  | SRC |
|  | PKN1 |
|  | GPR35 |
|  | MMP3 |
|  | MET |
|  | CDK2 |
|  | NOX4 |
|  | CA1 |
|  | AKR1C1 |
|  | MYLK |
|  | ABCB1 |
|  | ESRRA |
|  | AHR |
|  | AXL |
|  | AVPR2 |
|  | CDK5 |
|  | ADORA3 |
|  | AKR1C4 |
|  | CA5A |
|  | FLT3 |
|  | MAOA |
|  | PIM1 |
|  | ALK |
|  | MPO |
|  | CCNB2 |
|  | CA7 |
|  | ALOX5 |
|  | ADORA1 |
|  | CA6 |
|  | ST6GAL1 |
|  | MMP9 |
|  | NEK6 |
|  | PARP1 |
|  | AKR1B1 |
|  | KDM4E |
|  | OPRM1 |
| MOL004609 | TERT |
|  | CA2 |
|  | BACE1 |
|  | MPG |
|  | CA3 |
|  | KIT |
|  | ESR2 |
|  | MMP2 |
|  | AKR1C3 |
|  | AURKB |
|  | CDK1 |
|  | PTK2 |
|  | AKT1 |
|  | NUAK1 |
|  | XDH |
|  | PIK3R1 |
|  | PLG |
|  | GSK3B |
|  | TYR |
|  | CA14 |
|  | GRK6 |
|  | CA4 |
|  | DAPK1 |
|  | ALOX15 |
|  | DRD4 |
|  | MCL1 |
|  | PYGL |
|  | HSD17B2 |
|  | KDR |
|  | SLC22A12 |
|  | CA9 |
|  | ABCG2 |
|  | CDK6 |
|  | CA12 |
|  | AKR1C2 |
|  | PLA2G1B |
|  | AKR1A1 |
|  | MAPT |
|  | GLO1 |
|  | NEK2 |
|  | PTGS2 |
|  | ARG1 |
|  | CA13 |
|  | CAMK2B |
|  | NAE1 |
|  | MMP13 |
|  | TNKS2 |
|  | INSR |
|  | OPRD1 |
|  | PIK3CG |
|  | CFTR |
|  | ALOX12 |
|  | APP |
|  | CYP1B1 |
|  | PTPRS |
|  | TOP2A |
|  | ADORA2A |
|  | EGFR |
|  | APEX1 |
|  | ABCC1 |
|  | IGF1R |
|  | PKN1 |
|  | SRC |
|  | MMP3 |
|  | GPR35 |
|  | CD38 |
|  | MET |
|  | CDK2 |
|  | NOX4 |
|  | CA1 |
|  | AKR1C1 |
|  | MYLK |
|  | ABCB1 |
|  | ESRRA |
|  | AHR |
|  | AVPR2 |
|  | PLA2G2A |
|  | ADORA3 |
|  | AKR1C4 |
|  | CA5A |
|  | TNKS |
|  | AMY1A |
|  | ODC1 |
|  | FLT3 |
|  | MAOA |
|  | ALK |
|  | PIM1 |
|  | MPO |
|  | CA7 |
|  | CA6 |
|  | ADORA1 |
|  | ALOX5 |
|  | ST6GAL1 |
|  | MMP9 |
|  | NEK6 |
|  | TTR |
|  | AR |
|  | AKR1B1 |
|  | KDM4E |
|  | OPRM1 |
| MOL013187 | CASP1 |
|  | SYK |
|  | CYP17A1 |
|  | HCK |
|  | CDK4 |
|  | FNTB |
|  | CDK1 |
|  | ROCK1 |
|  | MTOR |
|  | SLC6A2 |
|  | SHBG |
|  | JAK2 |
|  | CHEK1 |
|  | ROCK2 |
|  | ALOX15 |
|  | CHEK2 |
|  | BRD4 |
|  | FBP1 |
|  | TDP2 |
|  | PYGL |
|  | MCL1 |
|  | ITK |
|  | TXK |
|  | CA9 |
|  | NTRK1 |
|  | LYPLA1 |
|  | EPHB4 |
|  | ADA |
|  | PIK3CB |
|  | MMP7 |
|  | LYPLA2 |
|  | MMP13 |
|  | HTR1A |
|  | LCK |
|  | MMP14 |
|  | PIK3CG |
|  | PLK1 |
|  | ALOX12 |
|  | KMO |
|  | PIK3CD |
|  | MAPK9 |
|  | ADAM10 |
|  | SLC6A4 |
|  | MAP2K1 |
|  | EGFR |
|  | FNTA |
|  | WEE1 |
|  | SLC6A3 |
|  | FGFR1 |
|  | PDE2A |
|  | CDK2 |
|  | ERN1 |
|  | CASP6 |
|  | PLA2G2A |
|  | PNP |
|  | CASP3 |
|  | CCND1 |
|  | CAPN1 |
|  | ABL1 |
|  | PIK3CA |
|  | PI4KB |
|  | CDK9 |
|  | PRKDC |
|  | JAK3 |
|  | TOP1 |
|  | JAK1 |
|  | ADAM17 |
|  | FCER2 |
|  | HDAC2 |
| MOL004644 | MPG |
|  | TNKS2 |
|  | PDE4D |
|  | PDE4B |
|  | ESR2 |
|  | TNKS |
|  | PTGES |
|  | PTPRS |
|  | ODC1 |
|  | SHBG |
|  | SIRT1 |
|  | XDH |
|  | ABCC1 |
|  | PLG |
|  | GLO1 |
|  | GPR35 |
|  | CD38 |
|  | ESR1 |
|  | DAPK1 |
|  | NR1H3 |
|  | NAE1 |
| MOL004653 | NAMPT |
|  | FAAH |
|  | PFKFB3 |
|  | F2RL1 |
|  | ECE1 |
|  | NPY5R |
|  | CDK4 |
|  | CDK1 |
|  | PTK2 |
|  | ROCK1 |
|  | KCNJ3 |
|  | CHRM4 |
|  | CCND2 |
|  | RBP4 |
|  | BRAF |
|  | TGM2 |
|  | P2RX3 |
|  | TGM1 |
|  | JAK2 |
|  | APH1A |
|  | CHRM1 |
|  | CPT1A |
|  | CCNB3 |
|  | NOS1 |
|  | GSK3B |
|  | KCNJ6 |
|  | TACR3 |
|  | MGAT2 |
|  | PREP |
|  | CCNA1 |
|  | CPT1B |
|  | PABPC1 |
|  | CYP19A1 |
|  | HTR2A |
|  | DRD4 |
|  | HTR6 |
|  | ELANE |
|  | HCRTR2 |
|  | KCNJ5 |
|  | PPIA |
|  | CASP7 |
|  | KDR |
|  | GRM5 |
|  | MMP1 |
|  | BDKRB1 |
|  | CCND3 |
|  | CFD |
|  | CTSS |
|  | NOS2 |
|  | PSEN1 |
|  | PSENEN |
|  | CYP11B1 |
|  | SCN9A |
|  | GRM4 |
|  | LCK |
|  | HTR1A |
|  | ELOVL6 |
|  | GCK |
|  | DBF4 |
|  | KCNK9 |
|  | CCNB1 |
|  | CDC7 |
|  | P2RX7 |
|  | CPT2 |
|  | MAPK9 |
|  | PDE10A |
|  | MAPK8 |
|  | EGFR |
|  | ADORA2A |
|  | MME |
|  | KCNK3 |
|  | HTR7 |
|  | PSEN2 |
|  | ITGB3 |
|  | CMA1 |
|  | SRC |
|  | MMP3 |
|  | F10 |
|  | HCRTR1 |
|  | CDK2 |
|  | CHRM3 |
|  | NCSTN |
|  | CCNE1 |
|  | CNR1 |
|  | TRPV1 |
|  | PDE4B |
|  | CSF1R |
|  | CHRM2 |
|  | DRD2 |
|  | CNR2 |
|  | ADORA3 |
|  | CASP3 |
|  | CCND1 |
|  | CTSK |
|  | CTSV |
|  | F13A1 |
|  | ABL1 |
|  | PIM1 |
|  | CCNA2 |
|  | PRKDC |
|  | TSPO |
|  | FAP |
|  | CCNB2 |
|  | ADORA1 |
|  | JAK3 |
|  | MMP9 |
|  | CCNE2 |
|  | CYP11B2 |
|  | APH1B |
|  | MAPK10 |
|  | DAPK3 |
|  | ITGAV |
|  | PDE7A |
|  | CTSL |
| MOL000468 | CA9 |
|  | CA2 |
|  | PON1 |
|  | PFKFB3 |
|  | GRM4 |
|  | ESR2 |
|  | TLR9 |
|  | RAF1 |
|  | ALOX12 |
|  | HTR2C |
|  | PTPRS |
|  | MIF |
|  | EGLN1 |
|  | PDE10A |
|  | SLC6A2 |
|  | EGFR |
|  | ADORA2A |
|  | XDH |
|  | TYR |
|  | MGAM |
|  | CA4 |
|  | NOX4 |
|  | HSD17B1 |
|  | CA1 |
|  | ALOX15 |
|  | BRD4 |
|  | ABCB1 |
|  | CYP19A1 |
|  | HTR2A |
|  | ESRRA |
|  | ERN1 |
|  | DHODH |
|  | HSD17B2 |
|  | ESRRB |
|  | MAOA |
|  | ABCG2 |
|  | CA12 |
|  | TBXAS1 |
|  | CA7 |
|  | CBR1 |
|  | ADORA1 |
|  | ALDH2 |
|  | IL2 |
|  | ESR1 |
|  | EZR |
| MOL000469 | LSS |
|  | FAAH |
|  | BACE1 |
|  | HMGCR |
|  | CYP17A1 |
|  | PTGER3 |
|  | ESR2 |
|  | HSD11B2 |
|  | AKR1B10 |
|  | GABRA3 |
|  | FABP4 |
|  | FNTB |
|  | PTGES |
|  | GABRB3 |
|  | GABRA5 |
|  | SHBG |
|  | FABP5 |
|  | NR3C2 |
|  | PRKCH |
|  | GABRA2 |
|  | PPARG |
|  | PREP |
|  | MPEG1 |
|  | NR1H3 |
|  | BCHE |
|  | PDE4D |
|  | CYP19A1 |
|  | SIGMAR1 |
|  | HSD17B3 |
|  | NR1I2 |
|  | TERT |
|  | HSD17B2 |
|  | FABP3 |
|  | IDO1 |
|  | CES1 |
|  | XIAP |
|  | RORC |
|  | PTPN11 |
|  | CYP51A1 |
|  | FDFT1 |
|  | IL6 |
|  | SLC22A6 |
|  | PTGS2 |
|  | ITGAL |
|  | NOS2 |
|  | GLUL |
|  | PDE4A |
|  | TNF |
|  | FABP1 |
|  | POLB |
|  | G6PD |
|  | CES2 |
|  | SREBF2 |
|  | MDM2 |
|  | GABRA1 |
|  | KAT2B |
|  | PTPN6 |
|  | SRD5A2 |
|  | SLC6A4 |
|  | NPC1L1 |
|  | PTPRF |
|  | PTPN2 |
|  | TOP2A |
|  | MAPK8 |
|  | PTPN1 |
|  | FNTA |
|  | MAST3 |
|  | ACP1 |
|  | PTGFR |
|  | PDE4C |
|  | SLC6A3 |
|  | PTGER1 |
|  | SERPINA6 |
|  | CNR1 |
|  | PDE4B |
|  | PGR |
|  | DRD2 |
|  | PPARA |
|  | S1PR1 |
|  | ADORA3 |
|  | CXCR3 |
|  | PTGIR |
|  | S1PR3 |
|  | MAPK3 |
|  | RORA |
|  | ALOX5 |
|  | NR3C1 |
|  | NR1I3 |
|  | TOP1 |
|  | AR |
|  | GABRG2 |
|  | ESR1 |
|  | ATP12A |
|  | CDC25A |
|  | PTGER2 |
|  | HSD11B1 |
| MOL000483 | SLC5A1 |
|  | MMP8 |
|  | SYK |
|  | EPHA5 |
|  | MMP2 |
|  | LYN |
|  | VCP |
|  | YES1 |
|  | CDK4 |
|  | CDK1 |
|  | ROCK1 |
|  | MTOR |
|  | BRAF |
|  | TYMS |
|  | SHBG |
|  | DNM1 |
|  | ACHE |
|  | TYR |
|  | PTK6 |
|  | CHEK1 |
|  | HSD17B1 |
|  | UPP1 |
|  | BCHE |
|  | ROCK2 |
|  | CHEK2 |
|  | MTNR1A |
|  | EPHA2 |
|  | EPHB3 |
|  | BCL2 |
|  | MCL1 |
|  | CDK5R1 |
|  | BLK |
|  | EPHA4 |
|  | MMP16 |
|  | TXK |
|  | HSD17B2 |
|  | ESRRB |
|  | PRKACA |
|  | SF3B3 |
|  | DRD3 |
|  | MMP1 |
|  | NEK1 |
|  | CFD |
|  | CSK |
|  | EPHA6 |
|  | MMP7 |
|  | EPHA3 |
|  | ANPEP |
|  | PTGS2 |
|  | COQ8B |
|  | TNF |
|  | HDAC1 |
|  | MMP13 |
|  | INSR |
|  | MMP14 |
|  | MMP12 |
|  | HDAC11 |
|  | THRB |
|  | MMP15 |
|  | PDK1 |
|  | MIF |
|  | ADORA2A |
|  | EGFR |
|  | PTPN1 |
|  | ABCC1 |
|  | FGR |
|  | EPHA1 |
|  | WEE1 |
|  | NCOR2 |
|  | MMP3 |
|  | PRKCZ |
|  | CALCRL |
|  | MTNR1B |
|  | ESRRA |
|  | ERN1 |
|  | CDK5 |
|  | BCL2L1 |
|  | DRD2 |
|  | CNR2 |
|  | ADORA3 |
|  | CAPN1 |
|  | ABL1 |
|  | NCOR1 |
|  | BMX |
|  | ALOX5 |
|  | BMP1 |
|  | RPS6KB1 |
|  | HTR3A |
|  | ADORA1 |
|  | MMP9 |
|  | DUSP3 |
|  | TYRO3 |
|  | EPHB2 |
|  | ALDH2 |
|  | ADAM17 |
|  | NPY1R |
|  | MAOB |
|  | THRA |
|  | MMP26 |
|  | HDAC3 |
|  | CTSL |
|  | BTK |
| MOL000491 | VCP |
|  | FAAH |
|  | MMP8 |
|  | UBA2 |
|  | NPY5R |
|  | CDK1 |
|  | TLR4 |
|  | FNTB |
|  | CDK4 |
|  | CSNK2A1 |
|  | PTK2 |
|  | ROCK1 |
|  | CCND2 |
|  | JAK2 |
|  | APH1A |
|  | NMBR |
|  | CCNB3 |
|  | CPT1A |
|  | NOS1 |
|  | GSK3B |
|  | PDE9A |
|  | PDGFRA |
|  | SCN2A |
|  | PREP |
|  | CCNA1 |
|  | MAPK1 |
|  | SLC6A9 |
|  | HTR6 |
|  | BPTF |
|  | PLA2G7 |
|  | CDK5R1 |
|  | PYGL |
|  | ELANE |
|  | HCRTR2 |
|  | KCNQ2 |
|  | PPIA |
|  | KDR |
|  | PRKCA |
|  | KCNQ3 |
|  | PIM2 |
|  | GRM5 |
|  | SCN10A |
|  | MMP1 |
|  | BDKRB1 |
|  | CCND3 |
|  | PDGFRB |
|  | TRPA1 |
|  | CFD |
|  | MALT1 |
|  | IDH1 |
|  | NOS3 |
|  | PTGS2 |
|  | PSEN1 |
|  | NOS2 |
|  | FLT1 |
|  | PSENEN |
|  | SCN5A |
|  | SCN9A |
|  | MMP12 |
|  | HTR1A |
|  | PLK1 |
|  | CCNB1 |
|  | KCNK9 |
|  | KAT2B |
|  | P2RX7 |
|  | PIK3CD |
|  | CPT2 |
|  | MAPK9 |
|  | CACNA1G |
|  | PDE10A |
|  | MAPK8 |
|  | ADORA2A |
|  | FNTA |
|  | IKBKB |
|  | KCNK3 |
|  | TYK2 |
|  | HTR7 |
|  | PTGER1 |
|  | PSEN2 |
|  | MMP3 |
|  | F10 |
|  | HCRTR1 |
|  | PDE2A |
|  | CDK2 |
|  | NCSTN |
|  | CNR1 |
|  | CCNE1 |
|  | CDK5 |
|  | PDE4B |
|  | DRD2 |
|  | CNR2 |
|  | ADORA3 |
|  | CCND1 |
|  | CTSK |
|  | ABL1 |
|  | CCNA2 |
|  | PIM1 |
|  | SAE1 |
|  | TSPO |
|  | CCNB2 |
|  | ALOX5 |
|  | ADORA1 |
|  | MMP9 |
|  | JAK3 |
|  | FPR1 |
|  | PLK2 |
|  | OXTR |
|  | CCNE2 |
|  | APH1B |
|  | ITGB7 |
|  | JAK1 |
|  | MAPK10 |
|  | HTR2B |
|  | PDE7A |
|  | HDAC2 |
|  | NAMPT |
|  | PGGT1B |
|  | ITGA4 |
| MOL000497 | PTPN6 |
|  | MMP8 |
|  | TRPM8 |
|  | BACE1 |
|  | HDAC4 |
|  | NPY5R |
|  | VCP |
|  | CDC25B |
|  | NAAA |
|  | ROCK1 |
|  | BRAF |
|  | KCNA3 |
|  | CHRNA4 |
|  | P2RX3 |
|  | PRKCB |
|  | JAK2 |
|  | APH1A |
|  | ACHE |
|  | PIM3 |
|  | CHRNB2 |
|  | TYR |
|  | PTGS1 |
|  | TACR3 |
|  | PDPK1 |
|  | F3 |
|  | MAPK1 |
|  | ROCK2 |
|  | BCHE |
|  | CYP19A1 |
|  | SLC5A1 |
|  | HCRTR2 |
|  | ITK |
|  | MAPK14 |
|  | ESRRB |
|  | IMPDH1 |
|  | PIM2 |
|  | MMP1 |
|  | ABCG2 |
|  | PDGFRB |
|  | PLA2G1B |
|  | ITGB2 |
|  | RET |
|  | PTGS2 |
|  | PSEN1 |
|  | ITGAL |
|  | NOS2 |
|  | HDAC10 |
|  | MMP13 |
|  | PSENEN |
|  | HDAC1 |
|  | CYP11B1 |
|  | HTR1A |
|  | CTSD |
|  | HDAC11 |
|  | MDM2 |
|  | RAF1 |
|  | THRB |
|  | APP |
|  | ADAM10 |
|  | PDK1 |
|  | SLC5A2 |
|  | MIF |
|  | HPGDS |
|  | PDE10A |
|  | EGFR |
|  | PTPN1 |
|  | HDAC9 |
|  | SLC29A1 |
|  | HDAC8 |
|  | NCOR2 |
|  | PSEN2 |
|  | MDM4 |
|  | ICAM1 |
|  | NCSTN |
|  | ABCB1 |
|  | HDAC6 |
|  | ERN1 |
|  | PDE4B |
|  | PLA2G2A |
|  | S1PR1 |
|  | CAPN1 |
|  | S1PR3 |
|  | CHRNA7 |
|  | NR1H4 |
|  | ABL1 |
|  | ODC1 |
|  | FLT3 |
|  | PIM1 |
|  | ADORA2B |
|  | GNRHR |
|  | MAOA |
|  | ALOX5 |
|  | BMP1 |
|  | RPS6KB1 |
|  | HDAC5 |
|  | MMP9 |
|  | ADRA1B |
|  | APH1B |
|  | CYP11B2 |
|  | MAPK10 |
|  | ADAM17 |
|  | MAOB |
|  | AKR1B1 |
|  | THRA |
|  | FASN |
|  | CDC25A |
|  | MAPKAPK2 |
|  | HDAC7 |
|  | HDAC3 |
| MOL000500 | PIK3CA |
|  | TRPM8 |
|  | PFKFB3 |
|  | PRF1 |
|  | ESR2 |
|  | HSP90AB1 |
|  | AKR1B10 |
|  | VCP |
|  | AURKB |
|  | CDK4 |
|  | CDK1 |
|  | ROCK1 |
|  | BRAF |
|  | CCNB3 |
|  | PIM3 |
|  | TYR |
|  | GSK3B |
|  | GRK6 |
|  | CA14 |
|  | PTGS1 |
|  | CHEK1 |
|  | F3 |
|  | BCHE |
|  | ALOX15 |
|  | SIGMAR1 |
|  | HTR2A |
|  | CYP19A1 |
|  | KIF11 |
|  | CYP24A1 |
|  | HSD17B3 |
|  | MCL1 |
|  | SNCA |
|  | CDK5R1 |
|  | EIF2AK2 |
|  | CASP7 |
|  | HSD17B2 |
|  | ESRRB |
|  | PRKCA |
|  | PRKACA |
|  | PIM2 |
|  | GRIA1 |
|  | NOX1 |
|  | RET |
|  | CDK3 |
|  | LNPEP |
|  | TAAR1 |
|  | GSTP1 |
|  | CTSS |
|  | PTGS2 |
|  | HDAC10 |
|  | INSR |
|  | TNKS2 |
|  | LCK |
|  | PIK3CG |
|  | HDAC11 |
|  | RAF1 |
|  | PLK1 |
|  | ALOX12 |
|  | CCNB1 |
|  | PIK3CD |
|  | AURKA |
|  | ALPL |
|  | PDK1 |
|  | COMT |
|  | MIF |
|  | LIMK1 |
|  | ADORA2A |
|  | MAP2K1 |
|  | HDAC9 |
|  | PTPN1 |
|  | IGF1R |
|  | WEE1 |
|  | NOX4 |
|  | CDK2 |
|  | GSTM2 |
|  | ADCY5 |
|  | CCNE1 |
|  | ESRRA |
|  | CDK5 |
|  | DRD2 |
|  | CASP3 |
|  | HSP90AA1 |
|  | ABL1 |
|  | CHRNA7 |
|  | TNKS |
|  | ALOX15B |
|  | ADORA2B |
|  | PIM1 |
|  | DYRK1B |
|  | CCNB2 |
|  | HTT |
|  | RPS6KB1 |
|  | DUSP3 |
|  | HSD17B14 |
|  | ADAM17 |
|  | MAOB |
|  | PDE7A |
|  | HDAC2 |
|  | CLK1 |
|  | ESR1 |
|  | MAPKAPK2 |
|  | HDAC3 |
|  | EZR |
| MOL000502 | ALOX12 |
|  | CA2 |
|  | PON1 |
|  | PFKFB3 |
|  | ESR2 |
|  | HTR2C |
|  | ALPL |
|  | PTPRS |
|  | MTOR |
|  | MIF |
|  | SLC6A2 |
|  | ADORA2A |
|  | EGFR |
|  | PTPN1 |
|  | XDH |
|  | ACHE |
|  | PLAA |
|  | TYR |
|  | GUSB |
|  | PTGS1 |
|  | MGAM |
|  | CA4 |
|  | NOX4 |
|  | HSD17B1 |
|  | KDM1A |
|  | CA1 |
|  | ALOX15 |
|  | ABCB1 |
|  | ESRRA |
|  | CYP19A1 |
|  | HTR2A |
|  | PPARA |
|  | HSD17B2 |
|  | CHRNA7 |
|  | ESRRB |
|  | PIK3CA |
|  | PIM2 |
|  | MAOA |
|  | ABCG2 |
|  | TBXAS1 |
|  | CA12 |
|  | CA7 |
|  | CBR1 |
|  | ADORA1 |
|  | ALDH2 |
|  | IL2 |
|  | MAOB |
|  | ESR1 |
|  | EZR |
| MOL000503 | ALOX5 |
|  | CBR1 |
|  | ESR1 |
|  | ESR2 |
|  | CA12 |
|  | CA7 |
|  | CA2 |
| MOL000506 | HRH1 |
|  | ADRA2A |
|  | HTR2C |
|  | PNMT |
|  | SLC6A4 |
|  | CYP2D6 |
|  | PAOX |
|  | CHRM4 |
|  | SLC6A2 |
|  | CHRNB4 |
|  | EGFR |
|  | CHRNA4 |
|  | OPRK1 |
|  | DNM1 |
|  | KCNH2 |
|  | ACHE |
|  | CHRM1 |
|  | CHRNB2 |
|  | ADRA2C |
|  | SLC6A3 |
|  | CHRM5 |
|  | FYN |
|  | SLC18A2 |
|  | BCHE |
|  | CHRM3 |
|  | SIGMAR1 |
|  | DRD2 |
|  | CHRM2 |
|  | PLA2G2A |
|  | CHRNA7 |
|  | DRD3 |
|  | ADRA1B |
|  | PLA2G1B |
|  | HRH3 |
|  | MAOB |
|  | CHRNA3 |
|  | TAAR1 |
|  | ADRA2B |
|  | SLC5A7 |
| MOL000507 | ALOX12 |
|  | CA2 |
|  | LCK |
|  | ESR2 |
|  | TLR9 |
|  | HTR2C |
|  | PTPRS |
|  | MIF |
|  | SLC6A2 |
|  | ADORA2A |
|  | EGFR |
|  | XDH |
|  | TYR |
|  | PTGS1 |
|  | MGAM |
|  | CA4 |
|  | HSD17B1 |
|  | CA1 |
|  | ABCB1 |
|  | CYP19A1 |
|  | HTR2A |
|  | ESRRA |
|  | PPARA |
|  | HSD17B2 |
|  | ESRRB |
|  | MAOA |
|  | ABCG2 |
|  | CA12 |
|  | TBXAS1 |
|  | CBR1 |
|  | CA7 |
|  | ADORA1 |
|  | ALDH2 |
|  | IL2 |
|  | MAOB |
|  | ESR1 |
|  | TNF |

| **Table S4. Herbal targets** | |
| --- | --- |
| **Herb** | **Target** |
| Huangqi | ABCB1 |
|  | ABCC1 |
|  | ABCG2 |
|  | ABL1 |
|  | ACHE |
|  | ACP1 |
|  | ADAM10 |
|  | ADAM17 |
|  | ADAMTS5 |
|  | ADK |
|  | ADORA1 |
|  | ADORA2A |
|  | ADORA2B |
|  | ADORA3 |
|  | AHR |
|  | AKR1A1 |
|  | AKR1B1 |
|  | AKR1B10 |
|  | AKR1C1 |
|  | AKR1C2 |
|  | AKR1C3 |
|  | AKR1C4 |
|  | AKT1 |
|  | ALDH2 |
|  | ALDH3A1 |
|  | ALK |
|  | ALOX12 |
|  | ALOX15 |
|  | ALOX15B |
|  | ALOX5 |
|  | ALOX5AP |
|  | ALPG |
|  | ALPL |
|  | AMY1A |
|  | ANPEP |
|  | APEX1 |
|  | APP |
|  | AR |
|  | ARG1 |
|  | ATIC |
|  | AURKA |
|  | AURKB |
|  | AVPR1A |
|  | AVPR2 |
|  | AXL |
|  | BACE1 |
|  | BAD |
|  | BCHE |
|  | BDKRB2 |
|  | BMP1 |
|  | BRAF |
|  | BRD2 |
|  | BRD3 |
|  | BRD4 |
|  | BRPF1 |
|  | CA1 |
|  | CA12 |
|  | CA13 |
|  | CA14 |
|  | CA2 |
|  | CA3 |
|  | CA4 |
|  | CA5A |
|  | CA5B |
|  | CA6 |
|  | CA7 |
|  | CA9 |
|  | CAMK2B |
|  | CASP3 |
|  | CASP7 |
|  | CBR1 |
|  | CCNA1 |
|  | CCNA2 |
|  | CCNB1 |
|  | CCNB2 |
|  | CCNB3 |
|  | CCNC |
|  | CCND1 |
|  | CCND2 |
|  | CCND3 |
|  | CCNE1 |
|  | CCNE2 |
|  | CCNH |
|  | CCNT1 |
|  | CD38 |
|  | CDC25A |
|  | CDC25B |
|  | CDC25C |
|  | CDC7 |
|  | CDK1 |
|  | CDK2 |
|  | CDK4 |
|  | CDK5 |
|  | CDK5R1 |
|  | CDK6 |
|  | CDK7 |
|  | CDK8 |
|  | CDK9 |
|  | CES2 |
|  | CFTR |
|  | CHEK1 |
|  | CHIA |
|  | CHRM2 |
|  | CHRNA7 |
|  | CLK1 |
|  | CLK2 |
|  | CLK3 |
|  | CLK4 |
|  | CMA1 |
|  | CNR1 |
|  | CNR2 |
|  | COMT |
|  | CRHR1 |
|  | CSNK1D |
|  | CSNK1G1 |
|  | CSNK2A1 |
|  | CSNK2A2 |
|  | CTSG |
|  | CTSS |
|  | CXCR1 |
|  | CYP11B1 |
|  | CYP17A1 |
|  | CYP19A1 |
|  | CYP1A1 |
|  | CYP1B1 |
|  | CYP2C19 |
|  | CYP51A1 |
|  | DAO |
|  | DAPK1 |
|  | DBF4 |
|  | DHCR7 |
|  | DHFR |
|  | DHODH |
|  | DNM1 |
|  | DRD2 |
|  | DRD4 |
|  | DUSP3 |
|  | DYRK1B |
|  | DYRK2 |
|  | EGFR |
|  | EIF4A1 |
|  | ELANE |
|  | EP300 |
|  | EPHB2 |
|  | EPHX2 |
|  | ERCC5 |
|  | ERN1 |
|  | ESR1 |
|  | ESR2 |
|  | ESRRA |
|  | ESRRB |
|  | EZR |
|  | F10 |
|  | F2 |
|  | F3 |
|  | FAAH |
|  | FABP1 |
|  | FABP2 |
|  | FABP3 |
|  | FABP4 |
|  | FABP5 |
|  | FCER2 |
|  | FDFT1 |
|  | FEN1 |
|  | FFAR1 |
|  | FGFR1 |
|  | FLT1 |
|  | FLT3 |
|  | FOLR1 |
|  | FOLR2 |
|  | FPGS |
|  | FPR2 |
|  | G6PD |
|  | GABBR1 |
|  | GABRA1 |
|  | GABRA2 |
|  | GABRA3 |
|  | GABRA5 |
|  | GABRA6 |
|  | GABRB2 |
|  | GABRB3 |
|  | GABRG2 |
|  | GART |
|  | GLO1 |
|  | GLRA1 |
|  | GPBAR1 |
|  | GPR35 |
|  | GRK6 |
|  | GRM1 |
|  | GRM4 |
|  | GSK3B |
|  | HCRTR1 |
|  | HCRTR2 |
|  | HDAC1 |
|  | HDAC10 |
|  | HDAC11 |
|  | HDAC2 |
|  | HDAC3 |
|  | HDAC4 |
|  | HDAC5 |
|  | HDAC6 |
|  | HDAC7 |
|  | HDAC8 |
|  | HDAC9 |
|  | HK1 |
|  | HK2 |
|  | HMGCR |
|  | HRAS |
|  | HSD11B1 |
|  | HSD17B1 |
|  | HSD17B2 |
|  | HSD17B3 |
|  | HSF1 |
|  | HSP90AA1 |
|  | HSP90AB1 |
|  | HSP90B1 |
|  | HSPA5 |
|  | HTR2A |
|  | HTR2C |
|  | HTR3A |
|  | HTT |
|  | IGF1R |
|  | IKBKE |
|  | IL2 |
|  | IMPDH1 |
|  | IMPDH2 |
|  | INSR |
|  | JAK3 |
|  | JUN |
|  | KCNK9 |
|  | KDM4E |
|  | KDR |
|  | KIT |
|  | LCK |
|  | LDHB |
|  | LNPEP |
|  | LRRK2 |
|  | MAOA |
|  | MAOB |
|  | MAP2K1 |
|  | MAPK1 |
|  | MAPKAPK2 |
|  | MAPT |
|  | MBD2 |
|  | MCL1 |
|  | MERTK |
|  | MET |
|  | MGAM |
|  | MGMT |
|  | MIF |
|  | MKNK1 |
|  | MME |
|  | MMP1 |
|  | MMP12 |
|  | MMP13 |
|  | MMP16 |
|  | MMP2 |
|  | MMP25 |
|  | MMP3 |
|  | MMP7 |
|  | MMP8 |
|  | MMP9 |
|  | MPG |
|  | MPO |
|  | MTOR |
|  | MYLK |
|  | NADK |
|  | NAE1 |
|  | NCOR1 |
|  | NCOR2 |
|  | NDUFA4 |
|  | NEK2 |
|  | NEK6 |
|  | NOS2 |
|  | NOX4 |
|  | NPBWR1 |
|  | NPC1L1 |
|  | NQO2 |
|  | NR1H2 |
|  | NR1H3 |
|  | NR1H4 |
|  | NR1I3 |
|  | NR3C1 |
|  | NR4A1 |
|  | NTRK1 |
|  | NUAK1 |
|  | ODC1 |
|  | OPRD1 |
|  | OPRM1 |
|  | OXTR |
|  | PARP1 |
|  | PDE10A |
|  | PDE2A |
|  | PDE3A |
|  | PDE3B |
|  | PDE4C |
|  | PDE4D |
|  | PDE5A |
|  | PDE7A |
|  | PDGFRA |
|  | PDGFRB |
|  | PDK1 |
|  | PFKFB3 |
|  | PGK1 |
|  | PIK3CA |
|  | PIK3CB |
|  | PIK3CD |
|  | PIK3CG |
|  | PIK3R1 |
|  | PIM1 |
|  | PIM2 |
|  | PIM3 |
|  | PKN1 |
|  | PKN2 |
|  | PLA2G1B |
|  | PLAA |
|  | PLAT |
|  | PLAU |
|  | PLG |
|  | PLK1 |
|  | PLK2 |
|  | PLK3 |
|  | POLB |
|  | PON1 |
|  | PPARA |
|  | PPARD |
|  | PPARG |
|  | PPP1CA |
|  | PREP |
|  | PRF1 |
|  | PRKCA |
|  | PRKCQ |
|  | PSMB5 |
|  | PTGER1 |
|  | PTGER2 |
|  | PTGES |
|  | PTGS1 |
|  | PTGS2 |
|  | PTK2 |
|  | PTPN1 |
|  | PTPN11 |
|  | PTPN2 |
|  | PTPN6 |
|  | PTPRF |
|  | PTPRS |
|  | PYGL |
|  | PYGM |
|  | QPCTL |
|  | RAF1 |
|  | RET |
|  | RGS4 |
|  | ROCK1 |
|  | ROCK2 |
|  | RORA |
|  | RORC |
|  | RPS6KA1 |
|  | RPS6KA2 |
|  | RPS6KB1 |
|  | SAE1 |
|  | SCD |
|  | SCN9A |
|  | SERPINA6 |
|  | SF3B3 |
|  | SGK1 |
|  | SHBG |
|  | SIGMAR1 |
|  | SIRT2 |
|  | SLC19A1 |
|  | SLC1A3 |
|  | SLC22A12 |
|  | SLC27A1 |
|  | SLC28A2 |
|  | SLC29A1 |
|  | SLC2A1 |
|  | SLC46A1 |
|  | SLC5A1 |
|  | SLC5A2 |
|  | SLC6A2 |
|  | SLC6A3 |
|  | SLC6A4 |
|  | SQLE |
|  | SRC |
|  | SREBF2 |
|  | STS |
|  | SYK |
|  | TAAR1 |
|  | TAS2R31 |
|  | TBXAS1 |
|  | TERT |
|  | TGFBR1 |
|  | TLR9 |
|  | TNKS |
|  | TNKS2 |
|  | TOP1 |
|  | TOP2A |
|  | TRPM8 |
|  | TTK |
|  | TTR |
|  | TUBB1 |
|  | TUBB3 |
|  | TYMS |
|  | TYR |
|  | UBA2 |
|  | UGT2B7 |
|  | VCP |
|  | VDR |
|  | WEE1 |
|  | XDH |
| Yuganzi | TERT |
|  | FAAH |
|  | VDR |
|  | POLA1 |
|  | CA3 |
|  | PPARD |
|  | CYP17A1 |
|  | HMGCR |
|  | GPR119 |
|  | PTGER3 |
|  | MMP2 |
|  | AURKB |
|  | MTOR |
|  | GABRB3 |
|  | SLC6A2 |
|  | NUAK1 |
|  | XDH |
|  | PLG |
|  | TYR |
|  | ADRA2C |
|  | GSR |
|  | CCNA1 |
|  | SERPINE1 |
|  | HSD17B1 |
|  | CLK4 |
|  | CYP19A1 |
|  | STS |
|  | MAPK14 |
|  | HSD17B2 |
|  | FABP3 |
|  | KDR |
|  | IDO1 |
|  | PTAFR |
|  | ABCG2 |
|  | CDK6 |
|  | PDGFRB |
|  | P2RY1 |
|  | GLO1 |
|  | NOS2 |
|  | TAS2R31 |
|  | CAMK2B |
|  | CA13 |
|  | FLT1 |
|  | DYRK3 |
|  | MMP13 |
|  | TNKS2 |
|  | INSR |
|  | HTR1A |
|  | MAP3K8 |
|  | CCNB1 |
|  | ALOX12 |
|  | SLC6A4 |
|  | YARS |
|  | PTPRS |
|  | FNTA |
|  | PTPN1 |
|  | ACP1 |
|  | FGR |
|  | MMP3 |
|  | HCRTR1 |
|  | PDE2A |
|  | CRHR1 |
|  | SERPINA6 |
|  | MET |
|  | AKR1C1 |
|  | DYRK1A |
|  | AXL |
|  | CDK5 |
|  | PPARA |
|  | DRD2 |
|  | CNR2 |
|  | AKR1C4 |
|  | CA5B |
|  | ST3GAL3 |
|  | AMY1A |
|  | RORA |
|  | MPO |
|  | OGA |
|  | KLK2 |
|  | ALOX5 |
|  | JAK3 |
|  | APH1B |
|  | TTR |
|  | JAK1 |
|  | HTR2B |
|  | CXCR1 |
|  | GABRG2 |
|  | MAOB |
|  | PTGER2 |
|  | BACE1 |
|  | HSD11B2 |
|  | AKR1B10 |
|  | LYN |
|  | GLRA1 |
|  | FABP4 |
|  | FNTB |
|  | CDC25B |
|  | CYP2D6 |
|  | GABRB2 |
|  | GABRA5 |
|  | SCD |
|  | AKT1 |
|  | JAK2 |
|  | PIK3R1 |
|  | CYP2C19 |
|  | SMO |
|  | GRK6 |
|  | CA14 |
|  | NR1H2 |
|  | PDPK1 |
|  | TNFRSF1A |
|  | CA4 |
|  | STAT3 |
|  | NR1H3 |
|  | PGF |
|  | HSD17B3 |
|  | PYGL |
|  | TEK |
|  | TNNT2 |
|  | UGT2B7 |
|  | F2 |
|  | SLC22A12 |
|  | DNMT1 |
|  | LYPLA1 |
|  | HTR1B |
|  | CA9 |
|  | RORC |
|  | TBXAS1 |
|  | PLA2G1B |
|  | TNK2 |
|  | PTPN11 |
|  | ADRA2B |
|  | PTGS2 |
|  | ARG1 |
|  | FABP1 |
|  | PSENEN |
|  | SQLE |
|  | MMP14 |
|  | GRM4 |
|  | CES2 |
|  | PIK3CG |
|  | SELL |
|  | KLK1 |
|  | MDM2 |
|  | PLK1 |
|  | HTR2C |
|  | NPC1L1 |
|  | PTPRF |
|  | MAP2K1 |
|  | EGFR |
|  | CLK3 |
|  | ABCC1 |
|  | TYK2 |
|  | CD38 |
|  | NOX4 |
|  | MYLK |
|  | UTS2R |
|  | NCSTN |
|  | CCNE1 |
|  | AVPR1B |
|  | FOLH1 |
|  | CA5A |
|  | CCND1 |
|  | TNKS |
|  | PIK3CA |
|  | HPSE |
|  | FLT3 |
|  | PRKDC |
|  | ALK |
|  | MAOA |
|  | CCNB2 |
|  | ADORA1 |
|  | CDK8 |
|  | ESR1 |
|  | ERBB2 |
|  | HSPA1A |
|  | HSD11B1 |
|  | GLRA2 |
|  | FKBP5 |
|  | SYK |
|  | PFKFB3 |
|  | AKR1C3 |
|  | CDK4 |
|  | CDK1 |
|  | PTK2 |
|  | SHBG |
|  | FABP5 |
|  | DNM1 |
|  | GABRA2 |
|  | APH1A |
|  | PTGS1 |
|  | PREP |
|  | CHEK1 |
|  | GRM1 |
|  | BCHE |
|  | HTR2A |
|  | HTR6 |
|  | PDE4D |
|  | FUT7 |
|  | PPM1A |
|  | BCL2 |
|  | SNCA |
|  | DRD3 |
|  | PDE3A |
|  | PIK3CB |
|  | AKR1A1 |
|  | CLK2 |
|  | LYPLA2 |
|  | PSEN1 |
|  | DHCR7 |
|  | SREBF2 |
|  | G6PD |
|  | VEGFA |
|  | ENGASE |
|  | GCK |
|  | ADRA2A |
|  | PIK3CD |
|  | DAO |
|  | TOP2A |
|  | MAPK8 |
|  | ADORA2A |
|  | APEX1 |
|  | KCNH2 |
|  | IGF1R |
|  | PSEN2 |
|  | GPR35 |
|  | F10 |
|  | ICAM1 |
|  | CA1 |
|  | ADRA1D |
|  | ESRRA |
|  | AVPR2 |
|  | TNNI3 |
|  | ADORA3 |
|  | PLK4 |
|  | MAPK3 |
|  | CCNA2 |
|  | CA6 |
|  | OXTR |
|  | CCNE2 |
|  | MAPK10 |
|  | AR |
|  | AKR1B1 |
|  | CCNC |
|  | PGD |
|  | CA2 |
|  | FLT4 |
|  | MPG |
|  | MAP3K12 |
|  | HSP90AB1 |
|  | ESR2 |
|  | GABRA3 |
|  | PTGES |
|  | CSNK2A1 |
|  | BRAF |
|  | OPRK1 |
|  | FGF1 |
|  | ACHE |
|  | CCNB3 |
|  | PPARG |
|  | GSK3B |
|  | FUT4 |
|  | DAPK1 |
|  | CD81 |
|  | ALOX15 |
|  | DRD4 |
|  | CYP24A1 |
|  | CDK5R1 |
|  | HIF1A |
|  | EPHB4 |
|  | CA12 |
|  | STAT1 |
|  | TNNC1 |
|  | AKR1C2 |
|  | CFD |
|  | MAPT |
|  | CYP51A1 |
|  | NEK2 |
|  | TUBB1 |
|  | PDE4A |
|  | POLB |
|  | MMP12 |
|  | CFTR |
|  | GABRA1 |
|  | CYP1B1 |
|  | PTPN6 |
|  | AURKA |
|  | APP |
|  | KCNA5 |
|  | ADRA1A |
|  | PTPN2 |
|  | PDE10A |
|  | ROS1 |
|  | PTGFR |
|  | PTGER1 |
|  | SRC |
|  | PKN1 |
|  | SELE |
|  | CDK2 |
|  | ACKR3 |
|  | ABCB1 |
|  | CNR1 |
|  | AHR |
|  | PDE4B |
|  | CHRM2 |
|  | HSP90AA1 |
|  | DYRK1B |
|  | PIM1 |
|  | PDE3B |
|  | DYRK2 |
|  | PDE11A |
|  | CA7 |
|  | CBR1 |
|  | RPS6KB1 |
|  | MMP9 |
|  | NEK6 |
|  | PARP1 |
|  | NR1I3 |
|  | NR3C1 |
|  | TOP1 |
|  | DRD1 |
|  | CLK1 |
|  | CDC25A |
|  | KDM4E |
|  | OPRM1 |
| Kushen | TERT |
|  | FAAH |
|  | DPP8 |
|  | CA3 |
|  | PTGER3 |
|  | ECE1 |
|  | MMP2 |
|  | HCK |
|  | AURKB |
|  | WNT3A |
|  | GABRB3 |
|  | UGCG |
|  | ROCK1 |
|  | MTOR |
|  | ERCC5 |
|  | CHRM4 |
|  | SLC6A2 |
|  | NR3C2 |
|  | NUAK1 |
|  | XDH |
|  | PLG |
|  | TYR |
|  | ADRA2C |
|  | CCNA1 |
|  | MGAM |
|  | SERPINE1 |
|  | HSD17B1 |
|  | KDM1A |
|  | BRD4 |
|  | CYP19A1 |
|  | STS |
|  | HTR3B |
|  | HRH2 |
|  | HSD17B2 |
|  | KCNK2 |
|  | KDR |
|  | NTRK1 |
|  | PTAFR |
|  | PLA2G2C |
|  | ABCG2 |
|  | CDK6 |
|  | NOX1 |
|  | PDGFRB |
|  | ADRB1 |
|  | MMP7 |
|  | GRM3 |
|  | EP300 |
|  | GLO1 |
|  | CTSS |
|  | CTSB |
|  | CHRNB1 |
|  | NOS2 |
|  | TAS2R31 |
|  | CAMK2B |
|  | CA13 |
|  | TNF |
|  | FLT1 |
|  | INSR |
|  | TNKS2 |
|  | MMP13 |
|  | HTR1A |
|  | LCK |
|  | SIRT2 |
|  | TLR9 |
|  | RAF1 |
|  | MAP3K8 |
|  | CCNB1 |
|  | ALOX12 |
|  | PDK1 |
|  | SLC6A4 |
|  | GRM6 |
|  | PTPRS |
|  | FNTA |
|  | PTPN1 |
|  | YWHAG |
|  | MAP3K7 |
|  | CMA1 |
|  | MMP3 |
|  | FGFR1 |
|  | CHRM5 |
|  | CRHR1 |
|  | MET |
|  | ALPG |
|  | AKR1C1 |
|  | DYRK1A |
|  | AXL |
|  | CDK5 |
|  | PPARA |
|  | DRD2 |
|  | CNR2 |
|  | GRM2 |
|  | CA5B |
|  | AKR1C4 |
|  | CHRNA6 |
|  | ST3GAL3 |
|  | RPS6KA1 |
|  | AMY1A |
|  | SGK1 |
|  | ALOX15B |
|  | PI4KB |
|  | MPO |
|  | FAP |
|  | ALOX5 |
|  | KLK2 |
|  | KCNJ1 |
|  | JAK3 |
|  | PAK4 |
|  | DUSP3 |
|  | ADRA1B |
|  | HRH3 |
|  | TTR |
|  | JAK1 |
|  | HTR2B |
|  | CXCR1 |
|  | ADAM17 |
|  | GABRG2 |
|  | FCER2 |
|  | SLC22A2 |
|  | ITGA2B |
|  | CHRNA3 |
|  | MAOB |
|  | RPS6KA5 |
|  | MAPKAPK2 |
|  | PTGER2 |
|  | PCNA |
|  | BACE1 |
|  | TRPM8 |
|  | MMP8 |
|  | KIT |
|  | HSD11B2 |
|  | AKR1B10 |
|  | HDAC4 |
|  | HTR1F |
|  | FNTB |
|  | ADAMTS4 |
|  | CYP2D6 |
|  | ACVRL1 |
|  | GABRA5 |
|  | CCND2 |
|  | DPP7 |
|  | EGLN1 |
|  | AKT1 |
|  | RXRA |
|  | GRIN2B |
|  | PIK3R1 |
|  | GRM8 |
|  | CYP2C19 |
|  | PDGFRA |
|  | GRK6 |
|  | TBK1 |
|  | CA14 |
|  | PDPK1 |
|  | DGAT1 |
|  | ADRB3 |
|  | CA4 |
|  | ROCK2 |
|  | MAPK1 |
|  | SLC6A9 |
|  | PGF |
|  | SPHK1 |
|  | HSD17B3 |
|  | IMPDH2 |
|  | CISD1 |
|  | PYGL |
|  | ELANE |
|  | TXK |
|  | CHRNA1 |
|  | TNNT2 |
|  | LAP3 |
|  | IMPDH1 |
|  | PRKACA |
|  | F2 |
|  | ALOX5AP |
|  | SLC22A12 |
|  | XIAP |
|  | DNMT1 |
|  | CA9 |
|  | HTR1B |
|  | TBXAS1 |
|  | ADAMTS1 |
|  | TRPV3 |
|  | NEK1 |
|  | PLA2G1B |
|  | LNPEP |
|  | ADRA2B |
|  | PTGS2 |
|  | ARG1 |
|  | HDAC10 |
|  | NAE1 |
|  | PLA2G5 |
|  | HDAC1 |
|  | CHRNA5 |
|  | MMP14 |
|  | GRM4 |
|  | CES2 |
|  | PIK3CG |
|  | MDM2 |
|  | PLK1 |
|  | KLK1 |
|  | HTR2C |
|  | SLC5A2 |
|  | HPGDS |
|  | MIF |
|  | MKNK1 |
|  | EGFR |
|  | ADH1C |
|  | SIRT1 |
|  | IKBKB |
|  | ABCC1 |
|  | DPP4 |
|  | HTR7 |
|  | NCOR2 |
|  | ITGB3 |
|  | CD38 |
|  | CYP2C9 |
|  | DRD5 |
|  | NOX4 |
|  | REN |
|  | GRK2 |
|  | CHRM3 |
|  | UTS2R |
|  | MYLK |
|  | BAD |
|  | CCNE1 |
|  | EIF4A1 |
|  | GCGR |
|  | PLA2G2A |
|  | S1PR1 |
|  | CA5A |
|  | CCND1 |
|  | HRH4 |
|  | CTSK |
|  | TNKS |
|  | PIK3CA |
|  | GPBAR1 |
|  | FLT3 |
|  | PRKDC |
|  | ALK |
|  | MAOA |
|  | TSPO |
|  | OPRL1 |
|  | CCNB2 |
|  | ADORA1 |
|  | HDAC5 |
|  | ALDH2 |
|  | DNPEP |
|  | IL2 |
|  | ESR1 |
|  | ERBB2 |
|  | QDPR |
|  | HDAC7 |
|  | HSD11B1 |
|  | PON1 |
|  | SYK |
|  | PFKFB3 |
|  | AKR1C3 |
|  | ATP4A |
|  | SRD5A1 |
|  | CDK4 |
|  | CDK1 |
|  | AGTR1 |
|  | PNMT |
|  | GRIN1 |
|  | PARP2 |
|  | PTK2 |
|  | NAAA |
|  | TYMS |
|  | SHBG |
|  | CHRNA4 |
|  | P2RX3 |
|  | DNM1 |
|  | PTK6 |
|  | PIM3 |
|  | GBA |
|  | PTGS1 |
|  | CHEK1 |
|  | SLC18A2 |
|  | F3 |
|  | HTR5A |
|  | GRM1 |
|  | DCTPP1 |
|  | BCHE |
|  | PDE4D |
|  | HTR6 |
|  | HTR2A |
|  | FUT7 |
|  | SNCA |
|  | NR1I2 |
|  | BCL2 |
|  | MMP16 |
|  | ITK |
|  | MAP3K14 |
|  | ESRRB |
|  | DRD3 |
|  | GRM5 |
|  | PDE3A |
|  | HSD17B7 |
|  | CCKBR |
|  | PIK3CB |
|  | AKR1A1 |
|  | ADRB2 |
|  | CTSD |
|  | HPGD |
|  | OPRD1 |
|  | SREBF2 |
|  | VEGFA |
|  | STAT6 |
|  | MMP15 |
|  | TUBB3 |
|  | ADRA2A |
|  | PIK3CD |
|  | EPHX1 |
|  | SCARB1 |
|  | PAOX |
|  | DAO |
|  | PDE5A |
|  | TOP2A |
|  | ADORA2A |
|  | MME |
|  | EDNRA |
|  | APEX1 |
|  | FEN1 |
|  | IGF1R |
|  | KCNH2 |
|  | EEF2K |
|  | HDAC8 |
|  | GPR35 |
|  | F10 |
|  | CHRNB3 |
|  | CA1 |
|  | GSTM2 |
|  | ADRA1D |
|  | ADCY5 |
|  | ESRRA |
|  | ERN1 |
|  | MTAP |
|  | AVPR2 |
|  | BCL2L1 |
|  | TNNI3 |
|  | ADORA3 |
|  | BIRC2 |
|  | TACR1 |
|  | DHODH |
|  | ADAMTS5 |
|  | ABL1 |
|  | MAPK3 |
|  | CCNA2 |
|  | CA6 |
|  | HTR3A |
|  | HSD17B14 |
|  | CCNE2 |
|  | MAPK10 |
|  | CHRNG |
|  | AR |
|  | TNNI3K |
|  | PDE7A |
|  | HDAC2 |
|  | GABRA6 |
|  | CHRND |
|  | AKR1B1 |
|  | MMP26 |
|  | CTSL |
|  | EZR |
|  | FLT4 |
|  | PGD |
|  | CA2 |
|  | MPG |
|  | MAP3K12 |
|  | ESR2 |
|  | HSP90AB1 |
|  | GABRA3 |
|  | VCP |
|  | PTGES |
|  | FFAR1 |
|  | CSNK2A1 |
|  | BRAF |
|  | ATP4B |
|  | HSP90B1 |
|  | OPRK1 |
|  | DPP9 |
|  | ACHE |
|  | CHRM1 |
|  | CCNB3 |
|  | GSK3B |
|  | PPARG |
|  | CHRNB2 |
|  | PLAA |
|  | MERTK |
|  | GUSB |
|  | SORD |
|  | FUT4 |
|  | HRH1 |
|  | SLC47A1 |
|  | DAPK1 |
|  | HNF4A |
|  | PLA2G10 |
|  | ALOX15 |
|  | RNPEP |
|  | CHEK2 |
|  | FKBP1A |
|  | SIGMAR1 |
|  | DRD4 |
|  | MCL1 |
|  | CDK5R1 |
|  | FUCA1 |
|  | RELA |
|  | PRKCA |
|  | PIM2 |
|  | CES1 |
|  | MMP1 |
|  | EPHB4 |
|  | CCND3 |
|  | CA12 |
|  | TYMP |
|  | TNNC1 |
|  | STAT1 |
|  | AKR1C2 |
|  | CDK3 |
|  | CYP51A1 |
|  | RET |
|  | MAPT |
|  | GSTP1 |
|  | NEK2 |
|  | ANPEP |
|  | ITGAL |
|  | ADH1A |
|  | TUBB1 |
|  | POLB |
|  | SCN9A |
|  | SCN5A |
|  | EPHX2 |
|  | MMP12 |
|  | CFTR |
|  | HDAC11 |
|  | DBF4 |
|  | CDC7 |
|  | GABRA1 |
|  | AURKA |
|  | APP |
|  | CYP1B1 |
|  | KCNA5 |
|  | ALPL |
|  | SRD5A2 |
|  | ADRA1A |
|  | COMT |
|  | SPHK2 |
|  | RPS6KA3 |
|  | PDE10A |
|  | CHRNB4 |
|  | HDAC9 |
|  | ROS1 |
|  | PTGER4 |
|  | H1F0 |
|  | METAP1 |
|  | KISS1R |
|  | WEE1 |
|  | ADCY1 |
|  | PKN1 |
|  | SRC |
|  | PTGER1 |
|  | SLC6A3 |
|  | CDK2 |
|  | CNR1 |
|  | ABCB1 |
|  | HDAC6 |
|  | AHR |
|  | TRPV1 |
|  | DHFR |
|  | PGR |
|  | LTA4H |
|  | PDE4B |
|  | CHRNA2 |
|  | CHRM2 |
|  | PNP |
|  | TDP1 |
|  | HSP90AA1 |
|  | S1PR3 |
|  | CHRNA7 |
|  | NR1H4 |
|  | CSNK1G1 |
|  | ODC1 |
|  | PIM1 |
|  | DYRK1B |
|  | ADORA2B |
|  | PDE3B |
|  | GBA2 |
|  | PDE11A |
|  | CA7 |
|  | CBR1 |
|  | PRMT3 |
|  | RPS6KB1 |
|  | TAB1 |
|  | NEK6 |
|  | MMP9 |
|  | PARP1 |
|  | NR3C1 |
|  | TOP1 |
|  | CCR4 |
|  | DRD1 |
|  | HTR1D |
|  | CLK1 |
|  | CTSC |
|  | HDAC3 |
|  | KDM4E |
|  | OPRM1 |
| Jianghuang | PREP |
|  | POLB |
|  | VDR |
|  | SQLE |
|  | PPARD |
|  | CYP17A1 |
|  | HMGCR |
|  | CES2 |
|  | G6PD |
|  | SREBF2 |
|  | ESR2 |
|  | GLRA1 |
|  | PTPN6 |
|  | SHH |
|  | PTGES |
|  | SLC6A4 |
|  | NPC1L1 |
|  | PTPN2 |
|  | SLC6A2 |
|  | SHBG |
|  | PTPN1 |
|  | ACHE |
|  | CYP2C19 |
|  | PPARG |
|  | NR1H2 |
|  | PTGER1 |
|  | SERPINA6 |
|  | NR1H3 |
|  | BCHE |
|  | SIGMAR1 |
|  | CYP19A1 |
|  | CHRM2 |
|  | PPARA |
|  | UGT2B7 |
|  | RORA |
|  | RORC |
|  | TBXAS1 |
|  | NR1I3 |
|  | AR |
|  | CYP51A1 |
|  | FDFT1 |
|  | ESR1 |
|  | NOS2 |
|  | PTGER2 |
|  | HSD11B1 |
|  | DHCR7 |
| Chaihu | TERT |
|  | VDR |
|  | FAAH |
|  | CA3 |
|  | PPARD |
|  | HMGCR |
|  | CYP17A1 |
|  | GPR119 |
|  | ECE1 |
|  | MMP2 |
|  | NPY5R |
|  | HCK |
|  | AURKB |
|  | MTOR |
|  | ROCK1 |
|  | CHRM4 |
|  | PRCP |
|  | SLC6A2 |
|  | TGM1 |
|  | NUAK1 |
|  | XDH |
|  | PLG |
|  | TYR |
|  | KCNJ6 |
|  | ADRA2C |
|  | TACR3 |
|  | CCNA1 |
|  | HSD17B1 |
|  | BRD4 |
|  | CYP19A1 |
|  | HSD17B2 |
|  | HRH2 |
|  | KDR |
|  | FABP3 |
|  | NTRK1 |
|  | ABCG2 |
|  | PTAFR |
|  | PORCN |
|  | CDK6 |
|  | P2RY1 |
|  | MMP7 |
|  | GLO1 |
|  | CTSS |
|  | NOS2 |
|  | TAS2R31 |
|  | CA13 |
|  | CAMK2B |
|  | TNF |
|  | TNKS2 |
|  | INSR |
|  | MMP13 |
|  | LCK |
|  | HTR1A |
|  | CCNB1 |
|  | ALOX12 |
|  | SLC6A4 |
|  | PTPRS |
|  | FNTA |
|  | PTPN1 |
|  | KCNK3 |
|  | CMA1 |
|  | MMP3 |
|  | FGFR1 |
|  | HCRTR1 |
|  | PDE2A |
|  | MET |
|  | SERPINA6 |
|  | NMUR2 |
|  | AKR1C1 |
|  | AXL |
|  | CASP6 |
|  | CDK5 |
|  | DRD2 |
|  | PPARA |
|  | CNR2 |
|  | AKR1C4 |
|  | F13A1 |
|  | MGLL |
|  | AMY1A |
|  | RORA |
|  | PI4KB |
|  | CDK9 |
|  | MPO |
|  | FAP |
|  | ALOX5 |
|  | JAK3 |
|  | TTR |
|  | APH1B |
|  | ADAM17 |
|  | JAK1 |
|  | CXCR1 |
|  | FCER2 |
|  | ITGAV |
|  | NAMPT |
|  | PTGER2 |
|  | BACE1 |
|  | KIT |
|  | AKR1B10 |
|  | HSD11B2 |
|  | FNTB |
|  | FABP4 |
|  | CCND2 |
|  | RBP4 |
|  | TGM2 |
|  | AKT1 |
|  | JAK2 |
|  | GRIN2B |
|  | PIK3R1 |
|  | ACACB |
|  | CYP2C19 |
|  | NOS1 |
|  | GRK6 |
|  | CA14 |
|  | SMO |
|  | NR1H2 |
|  | MGAT2 |
|  | DGAT1 |
|  | CPT1B |
|  | CA4 |
|  | CASP1 |
|  | NR1H3 |
|  | ROCK2 |
|  | PABPC1 |
|  | ACACA |
|  | SLC5A1 |
|  | PYGL |
|  | ELANE |
|  | TXK |
|  | UGT2B7 |
|  | F2 |
|  | SLC22A12 |
|  | ALOX5AP |
|  | LYPLA1 |
|  | CA9 |
|  | RORC |
|  | TBXAS1 |
|  | PLA2G1B |
|  | FDFT1 |
|  | PTGS2 |
|  | ARG1 |
|  | NAE1 |
|  | PSENEN |
|  | SQLE |
|  | GRM4 |
|  | MMP14 |
|  | PIK3CG |
|  | PLK1 |
|  | KCNK9 |
|  | KMO |
|  | ADAM10 |
|  | NPC1L1 |
|  | MAP2K1 |
|  | EGFR |
|  | SIRT1 |
|  | ABCC1 |
|  | HTR7 |
|  | ITGB3 |
|  | CD38 |
|  | NOX4 |
|  | CHRM3 |
|  | MYLK |
|  | NCSTN |
|  | CCNE1 |
|  | PLA2G2A |
|  | CCND1 |
|  | CA5A |
|  | CTSK |
|  | TNKS |
|  | PIK3CA |
|  | FLT3 |
|  | PRKDC |
|  | ALK |
|  | MAOA |
|  | TSPO |
|  | CCNB2 |
|  | ADORA1 |
|  | ST6GAL1 |
|  | ALDH2 |
|  | IL2 |
|  | DAPK3 |
|  | ESR1 |
|  | HSD11B1 |
|  | SYK |
|  | PFKFB3 |
|  | F2RL1 |
|  | AKR1C3 |
|  | CDK4 |
|  | CDK1 |
|  | GRIN1 |
|  | PTK2 |
|  | SHBG |
|  | P2RX3 |
|  | APH1A |
|  | CPT1A |
|  | PREP |
|  | CHEK1 |
|  | BCHE |
|  | HTR2A |
|  | HTR6 |
|  | PDE4D |
|  | SNCA |
|  | HCRTR2 |
|  | KCNJ5 |
|  | ITK |
|  | PPIA |
|  | CASP7 |
|  | GRM5 |
|  | BDKRB1 |
|  | PIK3CB |
|  | AKR1A1 |
|  | LYPLA2 |
|  | PSEN1 |
|  | DHCR7 |
|  | OPRD1 |
|  | SREBF2 |
|  | G6PD |
|  | ELOVL6 |
|  | GCK |
|  | P2RX7 |
|  | EPHX1 |
|  | ADRA2A |
|  | PIK3CD |
|  | PDE5A |
|  | TOP2A |
|  | MAPK8 |
|  | ADORA2A |
|  | MME |
|  | APEX1 |
|  | IGF1R |
|  | PSEN2 |
|  | GPR35 |
|  | F10 |
|  | CALCRL |
|  | CA1 |
|  | F2R |
|  | ESRRA |
|  | ERN1 |
|  | CSF1R |
|  | AVPR2 |
|  | ADORA3 |
|  | CAPN1 |
|  | CTSV |
|  | ABL1 |
|  | CCNA2 |
|  | CA6 |
|  | CCNE2 |
|  | CYP11B2 |
|  | MAPK10 |
|  | AR |
|  | PDE7A |
|  | HDAC2 |
|  | AKR1B1 |
|  | CTSL |
|  | CA2 |
|  | MPG |
|  | ESR2 |
|  | SOAT1 |
|  | PTGES |
|  | CSNK2A1 |
|  | KCNJ3 |
|  | BRAF |
|  | ABHD6 |
|  | CHRM1 |
|  | ACHE |
|  | CCNB3 |
|  | PPARG |
|  | GSK3B |
|  | DAPK1 |
|  | ALOX15 |
|  | CHEK2 |
|  | TDP2 |
|  | FBP1 |
|  | DRD4 |
|  | CDK5R1 |
|  | MCL1 |
|  | SSTR4 |
|  | NQO2 |
|  | MMP1 |
|  | EPHB4 |
|  | CCND3 |
|  | CA12 |
|  | ADA |
|  | AKR1C2 |
|  | CFD |
|  | CYP51A1 |
|  | MAPT |
|  | NEK2 |
|  | POLB |
|  | CYP11B1 |
|  | SCN9A |
|  | MMP12 |
|  | CFTR |
|  | DBF4 |
|  | CDC7 |
|  | CYP1B1 |
|  | PTPN6 |
|  | APP |
|  | CPT2 |
|  | MAPK9 |
|  | PTPN2 |
|  | PDE10A |
|  | RPS6KA3 |
|  | WEE1 |
|  | PTGER1 |
|  | SRC |
|  | PKN1 |
|  | SLC6A3 |
|  | CDK2 |
|  | CNR1 |
|  | ABCB1 |
|  | AHR |
|  | TRPV1 |
|  | PDE4B |
|  | CHRM2 |
|  | PNP |
|  | CASP3 |
|  | ODC1 |
|  | PIM1 |
|  | CA7 |
|  | MMP9 |
|  | NEK6 |
|  | PARP1 |
|  | NR1I3 |
|  | TOP1 |
|  | KDM4E |
|  | OPRM1 |
| Jixueteng | AR |
|  | FAAH |
|  | VDR |
|  | CA3 |
|  | PPARD |
|  | HMGCR |
|  | CYP17A1 |
|  | PTGER3 |
|  | MMP2 |
|  | UBA2 |
|  | NPY5R |
|  | AURKB |
|  | TLR4 |
|  | ROCK1 |
|  | MTOR |
|  | GABRB3 |
|  | CHRM4 |
|  | ERCC5 |
|  | SLC6A2 |
|  | NR3C2 |
|  | NUAK1 |
|  | XDH |
|  | PLG |
|  | NMBR |
|  | PDE9A |
|  | TYR |
|  | ADRA2C |
|  | TACR3 |
|  | CCNA1 |
|  | MGAM |
|  | HSD17B1 |
|  | KDM1A |
|  | BRD4 |
|  | CYP19A1 |
|  | STS |
|  | MAPK14 |
|  | EIF2AK2 |
|  | HSD17B2 |
|  | KDR |
|  | FABP3 |
|  | PLAU |
|  | IDO1 |
|  | ABCG2 |
|  | CDK6 |
|  | NOX1 |
|  | PDGFRB |
|  | CSK |
|  | MALT1 |
|  | MMP7 |
|  | GLO1 |
|  | CTSS |
|  | EPHA3 |
|  | COQ8B |
|  | NOS2 |
|  | CAMK2B |
|  | CA13 |
|  | FLT1 |
|  | TNF |
|  | INSR |
|  | MMP13 |
|  | TNKS2 |
|  | LCK |
|  | HTR1A |
|  | TLR9 |
|  | RAF1 |
|  | CCNB1 |
|  | ALOX12 |
|  | PDK1 |
|  | SLC6A4 |
|  | PTPRS |
|  | FNTA |
|  | PTPN1 |
|  | ACP1 |
|  | KCNK3 |
|  | FGR |
|  | MMP3 |
|  | FYN |
|  | CHRM5 |
|  | PRKCZ |
|  | MET |
|  | PDE2A |
|  | HCRTR1 |
|  | SERPINA6 |
|  | AKR1C1 |
|  | MTNR1B |
|  | AXL |
|  | CDK5 |
|  | DRD2 |
|  | PPARA |
|  | CNR2 |
|  | AKR1C4 |
|  | CA5B |
|  | AMY1A |
|  | RORA |
|  | ALOX15B |
|  | MPO |
|  | ALOX5 |
|  | JAK3 |
|  | ADRA1B |
|  | PLK2 |
|  | DUSP3 |
|  | HRH3 |
|  | TTR |
|  | APH1B |
|  | CXCR1 |
|  | JAK1 |
|  | HTR2B |
|  | ADAM17 |
|  | GABRG2 |
|  | CHRNA3 |
|  | MAOB |
|  | ATP12A |
|  | FASN |
|  | MAPKAPK2 |
|  | NAMPT |
|  | PTGER2 |
|  | SLC5A7 |
|  | TRPM8 |
|  | MMP8 |
|  | BACE1 |
|  | HDAC4 |
|  | LYN |
|  | AKR1B10 |
|  | HSD11B2 |
|  | GLRA1 |
|  | FABP4 |
|  | FNTB |
|  | CDC25B |
|  | CYP2D6 |
|  | CCND2 |
|  | GABRA5 |
|  | EGLN1 |
|  | AKT1 |
|  | PRKCB |
|  | PRKCH |
|  | PIK3R1 |
|  | JAK2 |
|  | CYP2C19 |
|  | NOS1 |
|  | GRK6 |
|  | PDGFRA |
|  | CA14 |
|  | NR1H2 |
|  | PDPK1 |
|  | CA4 |
|  | MPEG1 |
|  | NR1H3 |
|  | MAPK1 |
|  | UPP1 |
|  | ROCK2 |
|  | SLC6A9 |
|  | BPTF |
|  | EPHA2 |
|  | HSD17B3 |
|  | PYGL |
|  | SLC5A1 |
|  | ELANE |
|  | TXK |
|  | IMPDH1 |
|  | UGT2B7 |
|  | PRKACA |
|  | F2 |
|  | KCNQ3 |
|  | GRIA1 |
|  | XIAP |
|  | CA9 |
|  | TBXAS1 |
|  | RORC |
|  | TRPA1 |
|  | NEK1 |
|  | PLA2G1B |
|  | IDH1 |
|  | PTPN11 |
|  | LNPEP |
|  | TAAR1 |
|  | FDFT1 |
|  | ADRA2B |
|  | PTGS2 |
|  | ARG1 |
|  | HDAC10 |
|  | PSENEN |
|  | HDAC1 |
|  | SQLE |
|  | FABP1 |
|  | MMP14 |
|  | GRM4 |
|  | CES2 |
|  | PIK3CG |
|  | PLAT |
|  | PLK1 |
|  | MDM2 |
|  | KCNK9 |
|  | HTR2C |
|  | ADAM10 |
|  | SLC5A2 |
|  | NPC1L1 |
|  | HPGDS |
|  | PTPRF |
|  | MIF |
|  | MAP2K1 |
|  | EGFR |
|  | IKBKB |
|  | ABCC1 |
|  | TYK2 |
|  | HTR7 |
|  | NCOR2 |
|  | CD38 |
|  | NOX4 |
|  | CHRM3 |
|  | NCSTN |
|  | CCNE1 |
|  | PLA2G2A |
|  | S1PR1 |
|  | CCND1 |
|  | CXCR3 |
|  | CA5A |
|  | CTSK |
|  | TNKS |
|  | PIK3CA |
|  | BMX |
|  | FLT3 |
|  | ALK |
|  | MAOA |
|  | SAE1 |
|  | TSPO |
|  | HTT |
|  | CCNB2 |
|  | ADORA1 |
|  | HDAC5 |
|  | ALDH2 |
|  | IL2 |
|  | NPY1R |
|  | ESR1 |
|  | PGGT1B |
|  | HDAC7 |
|  | HSD11B1 |
|  | PFKFB3 |
|  | PON1 |
|  | SYK |
|  | PRF1 |
|  | AKR1C3 |
|  | CDK1 |
|  | CDK4 |
|  | PNMT |
|  | PTK2 |
|  | NAAA |
|  | KCNA3 |
|  | TYMS |
|  | CHRNA4 |
|  | SHBG |
|  | P2RX3 |
|  | FABP5 |
|  | DNM1 |
|  | GABRA2 |
|  | APH1A |
|  | CPT1A |
|  | PIM3 |
|  | PTK6 |
|  | PTGS1 |
|  | SCN2A |
|  | PREP |
|  | CHEK1 |
|  | SLC18A2 |
|  | F3 |
|  | BCHE |
|  | HTR6 |
|  | HTR2A |
|  | PDE4D |
|  | KIF11 |
|  | EPHB3 |
|  | SNCA |
|  | NR1I2 |
|  | BCL2 |
|  | MMP16 |
|  | HCRTR2 |
|  | ITK |
|  | KCNQ2 |
|  | PPIA |
|  | CASP7 |
|  | ESRRB |
|  | DRD3 |
|  | GRM5 |
|  | SCN10A |
|  | BDKRB1 |
|  | EPHA6 |
|  | AKR1A1 |
|  | ITGB2 |
|  | SLC22A6 |
|  | PSEN1 |
|  | GLUL |
|  | DHCR7 |
|  | CTSD |
|  | G6PD |
|  | SREBF2 |
|  | THRB |
|  | P2RX7 |
|  | MMP15 |
|  | ADRA2A |
|  | PIK3CD |
|  | PAOX |
|  | TOP2A |
|  | LIMK1 |
|  | ADORA2A |
|  | MAPK8 |
|  | FEN1 |
|  | KCNH2 |
|  | IGF1R |
|  | PDE4C |
|  | HDAC8 |
|  | PSEN2 |
|  | GPR35 |
|  | F10 |
|  | ICAM1 |
|  | CALCRL |
|  | CA1 |
|  | GSTM2 |
|  | ADCY5 |
|  | ESRRA |
|  | ERN1 |
|  | AVPR2 |
|  | BCL2L1 |
|  | ADORA3 |
|  | DHODH |
|  | PTGIR |
|  | CAPN1 |
|  | ABL1 |
|  | MAPK3 |
|  | NCOR1 |
|  | GNRHR |
|  | CCNA2 |
|  | CA6 |
|  | BMP1 |
|  | HTR3A |
|  | OXTR |
|  | HSD17B14 |
|  | CCNE2 |
|  | EPHB2 |
|  | TYRO3 |
|  | CYP11B2 |
|  | MAPK10 |
|  | HDAC2 |
|  | PDE7A |
|  | AKR1B1 |
|  | CTSL |
|  | MMP26 |
|  | EZR |
|  | CA2 |
|  | EPHA5 |
|  | HSP90AB1 |
|  | ESR2 |
|  | GABRA3 |
|  | YES1 |
|  | VCP |
|  | PTGES |
|  | CSNK2A1 |
|  | BRAF |
|  | OPRK1 |
|  | CHRM1 |
|  | ACHE |
|  | CCNB3 |
|  | PLAA |
|  | CHRNB2 |
|  | GSK3B |
|  | PPARG |
|  | GUSB |
|  | HRH1 |
|  | DAPK1 |
|  | ALOX15 |
|  | CHEK2 |
|  | DRD4 |
|  | SIGMAR1 |
|  | MTNR1A |
|  | CYP24A1 |
|  | PLA2G7 |
|  | BLK |
|  | EPHA4 |
|  | CDK5R1 |
|  | MCL1 |
|  | TERT |
|  | PRKCA |
|  | SF3B3 |
|  | PIM2 |
|  | CES1 |
|  | MMP1 |
|  | CCND3 |
|  | CA12 |
|  | AKR1C2 |
|  | CFD |
|  | CDK3 |
|  | RET |
|  | NOS3 |
|  | CYP51A1 |
|  | NEK2 |
|  | GSTP1 |
|  | IL6 |
|  | ANPEP |
|  | ITGAL |
|  | PDE4A |
|  | POLB |
|  | CYP11B1 |
|  | SCN5A |
|  | SCN9A |
|  | MMP12 |
|  | CFTR |
|  | HDAC11 |
|  | CDC7 |
|  | KAT2B |
|  | GABRA1 |
|  | AURKA |
|  | APP |
|  | CYP1B1 |
|  | PTPN6 |
|  | ALPL |
|  | CPT2 |
|  | SRD5A2 |
|  | MAPK9 |
|  | COMT |
|  | CACNA1G |
|  | PTPN2 |
|  | PDE10A |
|  | CHRNB4 |
|  | HDAC9 |
|  | MAST3 |
|  | SLC29A1 |
|  | PTGFR |
|  | LSS |
|  | EPHA1 |
|  | WEE1 |
|  | PKN1 |
|  | SRC |
|  | SLC6A3 |
|  | PTGER1 |
|  | MDM4 |
|  | CDK2 |
|  | ABCB1 |
|  | CNR1 |
|  | AHR |
|  | HDAC6 |
|  | PGR |
|  | PDE4B |
|  | CHRM2 |
|  | CASP3 |
|  | HSP90AA1 |
|  | NR1H4 |
|  | CHRNA7 |
|  | S1PR3 |
|  | ODC1 |
|  | DYRK1B |
|  | ADORA2B |
|  | PIM1 |
|  | CA7 |
|  | CBR1 |
|  | RPS6KB1 |
|  | NEK6 |
|  | FPR1 |
|  | MMP9 |
|  | PARP1 |
|  | NR3C1 |
|  | NR1I3 |
|  | ITGB7 |
|  | TOP1 |
|  | CLK1 |
|  | THRA |
|  | CDC25A |
|  | KDM4E |
|  | HDAC3 |
|  | ITGA4 |
|  | BTK |

| **Table S5. A 100% binding possibility between component and target** | | |
| --- | --- | --- |
| **Component ID** | **Target** | **Probability** |
| MOL000211 | SAE1 | 1 |
|  | UBA2 | 1 |
|  | POLB | 1 |
|  | AKR1B10 | 1 |
| MOL000354 | XDH | 1 |
|  | CA2 | 1 |
|  | CA7 | 1 |
|  | CA12 | 1 |
|  | CA4 | 1 |
|  | CYP1B1 | 1 |
| MOL000392 | IL2 | 1 |
| MOL000422 | NOX4 | 1 |
|  | AKR1B1 | 1 |
|  | XDH | 1 |
|  | TYR | 1 |
|  | FLT3 | 1 |
|  | CA2 | 1 |
|  | ALOX5 | 1 |
|  | CA7 | 1 |
|  | HSD17B2 | 1 |
|  | ABCC1 | 1 |
|  | HSD17B1 | 1 |
|  | AHR | 1 |
|  | CA12 | 1 |
|  | ESRRA | 1 |
|  | ABCB1 | 1 |
|  | CYP1B1 | 1 |
|  | ABCG2 | 1 |
| MOL000098 | NOX4 | 1 |
|  | AVPR2 | 1 |
|  | AKR1B1 | 1 |
|  | XDH | 1 |
|  | MAOA | 1 |
|  | IGF1R | 1 |
|  | FLT3 | 1 |
|  | CYP19A1 | 1 |
|  | EGFR | 1 |
|  | F2 | 1 |
|  | CA2 | 1 |
|  | PIM1 | 1 |
|  | ALOX5 | 1 |
|  | AURKB | 1 |
|  | DRD4 | 1 |
|  | ADORA1 | 1 |
|  | CA7 | 1 |
|  | GLO1 | 1 |
|  | MPO | 1 |
|  | PIK3R1 | 1 |
|  | ADORA2A | 1 |
|  | DAPK1 | 1 |
|  | PYGL | 1 |
|  | CA1 | 1 |
|  | GSK3B | 1 |
|  | SRC | 1 |
|  | PTK2 | 1 |
|  | HSD17B2 | 1 |
|  | KDR | 1 |
|  | MMP13 | 1 |
|  | MMP3 | 1 |
|  | CA3 | 1 |
|  | ALOX15 | 1 |
|  | ABCC1 | 1 |
|  | PLK1 | 1 |
|  | CA6 | 1 |
|  | CDK1 | 1 |
|  | MMP9 | 1 |
|  | CA12 | 1 |
|  | MMP2 | 1 |
|  | PKN1 | 1 |
|  | CA14 | 1 |
|  | CA9 | 1 |
|  | CSNK2A1 | 1 |
|  | ALOX12 | 1 |
|  | MET | 1 |
|  | CA4 | 1 |
|  | NEK2 | 1 |
|  | CXCR1 | 1 |
|  | CAMK2B | 1 |
|  | ALK | 1 |
|  | AKT1 | 1 |
|  | ABCB1 | 1 |
|  | NEK6 | 1 |
|  | PLA2G1B | 1 |
|  | CA5A | 1 |
|  | BACE1 | 1 |
|  | CYP1B1 | 1 |
|  | AXL | 1 |
|  | ABCG2 | 1 |
|  | NUAK1 | 1 |
|  | AKR1C2 | 1 |
|  | AKR1C1 | 1 |
|  | AKR1C3 | 1 |
|  | AKR1C4 | 1 |
|  | CA13 | 1 |
|  | AKR1A1 | 1 |
| MOL001002 | GPR35 | 1 |
|  | ERBB2 | 1 |
|  | AKR1B1 | 1 |
|  | CCND1 | 1 |
|  | CDK4 | 1 |
|  | PDGFRB | 1 |
|  | FLT4 | 1 |
|  | IGF1R | 1 |
|  | INSR | 1 |
|  | EGFR | 1 |
|  | CA2 | 1 |
|  | CDK2 | 1 |
|  | CCNA1 | 1 |
|  | CCNA2 | 1 |
|  | AURKB | 1 |
|  | CA7 | 1 |
|  | CA1 | 1 |
|  | GSK3B | 1 |
|  | SRC | 1 |
|  | PTK2 | 1 |
|  | KDR | 1 |
|  | PLK1 | 1 |
|  | CA6 | 1 |
|  | CA12 | 1 |
|  | CA14 | 1 |
|  | CA9 | 1 |
|  | CSNK2A1 | 1 |
|  | MET | 1 |
|  | CA4 | 1 |
|  | PLK4 | 1 |
|  | CA13 | 1 |
|  | TEK | 1 |
|  | AKT1 | 1 |
|  | AURKA | 1 |
|  | CA5A | 1 |
|  | BACE1 | 1 |
|  | MAP3K8 | 1 |
|  | BRAF | 1 |
|  | EPHB4 | 1 |
|  | HSPA1A | 1 |
|  | NUAK1 | 1 |
|  | SQLE | 1 |
|  | FGR | 1 |
|  | LYN | 1 |
| MOL000569 | POLA1 | 1 |
|  | POLB | 1 |
| MOL000006 | NOX4 | 1 |
|  | AKR1B1 | 1 |
|  | CDK5R1 | 1 |
|  | CDK5 | 1 |
|  | XDH | 1 |
|  | MAOA | 1 |
|  | FLT3 | 1 |
|  | CA2 | 1 |
|  | CCNB3 | 1 |
|  | CDK1 | 1 |
|  | CCNB1 | 1 |
|  | CCNB2 | 1 |
|  | ALOX5 | 1 |
|  | ADORA1 | 1 |
|  | CA7 | 1 |
|  | GLO1 | 1 |
|  | APP | 1 |
|  | SYK | 1 |
|  | GSK3B | 1 |
|  | PARP1 | 1 |
|  | TTR | 1 |
|  | MMP9 | 1 |
|  | CA12 | 1 |
|  | MMP2 | 1 |
|  | CA4 | 1 |
|  | MMP12 | 1 |
|  | CD38 | 1 |
|  | CYP1B1 | 1 |
|  | ABCG2 | 1 |
|  | AKR1B10 | 1 |
|  | TNKS2 | 1 |
|  | TNKS | 1 |
|  | TOP1 | 1 |
|  | ARG1 | 1 |
| MOL006821 | MAPT | 1 |
|  | DNMT1 | 1 |
|  | DYRK1A | 1 |
|  | APP | 1 |
|  | MAPK14 | 1 |
|  | TERT | 1 |
|  | MMP2 | 1 |
|  | PGD | 1 |
|  | MET | 1 |
|  | MMP14 | 1 |
|  | ABCB1 | 1 |
|  | BACE1 | 1 |
|  | BCL2 | 1 |
|  | STAT1 | 1 |
|  | SQLE | 1 |
| MOL003347 | NR1I2 | 1 |
| MOL000507 | PPARA | 1 |

| **Table S6. VMC-related symbols identified in GeneCards** | | | | | | |
| --- | --- | --- | --- | --- | --- | --- |
| **Code** | **Symbol** | **Description** | **Category** | **GIFtS** | **GC id** | **Score** |
| 1 | STAT1 | Signal Transducer And Activator Of Transcription 1 | Protein Coding | 53 | GC02M190908 | 48.82 |
| 2 | TLR3 | Toll Like Receptor 3 | Protein Coding | 52 | GC04P186059 | 44.31 |
| 3 | TNF | Tumor Necrosis Factor | Protein Coding | 51 | GC06P047305 | 36.46 |
| 4 | IL6 | Interleukin 6 | Protein Coding | 50 | GC07P022765 | 31.33 |
| 5 | IFNG | Interferon Gamma | Protein Coding | 48 | GC12M068064 | 30.92 |
| 6 | CD8A | CD8a Molecule | Protein Coding | 46 | GC02M086784 | 30.01 |
| 7 | CCR5 | C-C Motif Chemokine Receptor 5 | Protein Coding | 46 | GC03P046383 | 29.92 |
| 8 | IL10 | Interleukin 10 | Protein Coding | 47 | GC01M206767 | 29.1 |
| 9 | CD40LG | CD40 Ligand | Protein Coding | 47 | GC0XP136649 | 28.89 |
| 10 | IFNA1 | Interferon Alpha 1 | Protein Coding | 39 | GC09P021494 | 28.47 |
| 11 | CD4 | CD4 Molecule | Protein Coding | 49 | GC12P006786 | 27.84 |
| 12 | TP53 | Tumor Protein P53 | Protein Coding | 54 | GC17M007661 | 26.2 |
| 13 | IFIH1 | Interferon Induced With Helicase C Domain 1 | Protein Coding | 47 | GC02M162267 | 26.14 |
| 14 | HLA-B | Major Histocompatibility Complex, Class I, B | Protein Coding | 45 | GC06M031315 | 25.87 |
| 15 | IL1B | Interleukin 1 Beta | Protein Coding | 48 | GC02M112829 | 25.83 |
| 16 | MYD88 | MYD88 Innate Immune Signal Transduction Adaptor | Protein Coding | 50 | GC03P038179 | 25.51 |
| 17 | CXCL8 | C-X-C Motif Chemokine Ligand 8 | Protein Coding | 41 | GC04P073740 | 25.34 |
| 18 | IL2 | Interleukin 2 | Protein Coding | 45 | GC04M122451 | 24.61 |
| 19 | IL4 | Interleukin 4 | Protein Coding | 46 | GC05P132673 | 23.87 |
| 20 | PTPRC | Protein Tyrosine Phosphatase Receptor Type C | Protein Coding | 51 | GC01P198607 | 23.72 |
| 21 | TLR4 | Toll Like Receptor 4 | Protein Coding | 51 | GC09P117704 | 23.02 |
| 22 | CCL2 | C-C Motif Chemokine Ligand 2 | Protein Coding | 48 | GC17P034255 | 22.78 |
| 23 | PRF1 | Perforin 1 | Protein Coding | 45 | GC10M070597 | 22.41 |
| 24 | ADA | Adenosine Deaminase | Protein Coding | 51 | GC20M044620 | 22.23 |
| 25 | CASP8 | Caspase 8 | Protein Coding | 52 | GC02P201233 | 22.06 |
| 26 | CRP | C-Reactive Protein | Protein Coding | 46 | GC01M159716 | 22 |
| 27 | CPT2 | Carnitine Palmitoyltransferase 2 | Protein Coding | 48 | GC01P053196 | 22 |
| 28 | IFNB1 | Interferon Beta 1 | Protein Coding | 41 | GC09M021077 | 21.27 |
| 29 | CCL5 | C-C Motif Chemokine Ligand 5 | Protein Coding | 43 | GC17M035871 | 21.03 |
| 30 | ISG15 | ISG15 Ubiquitin Like Modifier | Protein Coding | 47 | GC01P001001 | 20.84 |
| 31 | ICAM1 | Intercellular Adhesion Molecule 1 | Protein Coding | 50 | GC19P010270 | 20.64 |
| 32 | IL18BP | Interleukin 18 Binding Protein | Protein Coding | 39 | GC11P071998 | 20.5 |
| 33 | TNNI3 | Troponin I3, Cardiac Type | Protein Coding | 48 | GC19M055151 | 20.14 |
| 34 | TLR2 | Toll Like Receptor 2 | Protein Coding | 51 | GC04P153684 | 19.94 |
| 35 | IRF3 | Interferon Regulatory Factor 3 | Protein Coding | 47 | GC19M049659 | 19.76 |
| 36 | CXCL10 | C-X-C Motif Chemokine Ligand 10 | Protein Coding | 44 | GC04M076021 | 19.49 |
| 37 | DDX58 | DExD/H-Box Helicase 58 | Protein Coding | 47 | GC09M032455 | 19.21 |
| 38 | HLA-DRB1 | Major Histocompatibility Complex, Class II, DR Beta 1 | Protein Coding | 46 | GC06M032578 | 19.17 |
| 39 | IL7R | Interleukin 7 Receptor | Protein Coding | 47 | GC05P035852 | 18.8 |
| 40 | EIF2AK2 | Eukaryotic Translation Initiation Factor 2 Alpha Kinase 2 | Protein Coding | 45 | GC02M037099 | 18.76 |
| 41 | GPT | Glutamic--Pyruvic Transaminase | Protein Coding | 41 | GC08P144502 | 18.64 |
| 42 | CCL3 | C-C Motif Chemokine Ligand 3 | Protein Coding | 39 | GC17M036088 | 18.62 |
| 43 | HADHA | Hydroxyacyl-CoA Dehydrogenase Trifunctional Multienzyme Complex Subunit Alpha | Protein Coding | 45 | GC02M026190 | 18.6 |
| 44 | STAT3 | Signal Transducer And Activator Of Transcription 3 | Protein Coding | 52 | GC17M042313 | 18.52 |
| 45 | MX1 | MX Dynamin Like GTPase 1 | Protein Coding | 41 | GC21P041420 | 18.37 |
| 46 | ALB | Albumin | Protein Coding | 50 | GC04P073397 | 18.12 |
| 47 | IL17A | Interleukin 17A | Protein Coding | 42 | GC06P052186 | 18.03 |
| 48 | ACADVL | Acyl-CoA Dehydrogenase Very Long Chain | Protein Coding | 45 | GC17P007219 | 17.8 |
| 49 | RANBP2 | RAN Binding Protein 2 | Protein Coding | 45 | GC02P108719 | 17.57 |
| 50 | IL18 | Interleukin 18 | Protein Coding | 44 | GC11M112143 | 17.33 |
| 51 | DMD | Dystrophin | Protein Coding | 46 | GC0XM031047 | 17.28 |
| 52 | CXADR | CXADR Ig-Like Cell Adhesion Molecule | Protein Coding | 43 | GC21P017512 | 17.14 |
| 53 | FAS | Fas Cell Surface Death Receptor | Protein Coding | 50 | GC10P088969 | 16.85 |
| 54 | HLA-DQB1 | Major Histocompatibility Complex, Class II, DQ Beta 1 | Protein Coding | 44 | GC06M032804 | 16.81 |
| 55 | IFITM3 | Interferon Induced Transmembrane Protein 3 | Protein Coding | 41 | GC11M000319 | 16.53 |
| 56 | CTNNB1 | Catenin Beta 1 | Protein Coding | 53 | GC03P041236 | 16.52 |
| 57 | KIT | KIT Proto-Oncogene, Receptor Tyrosine Kinase | Protein Coding | 53 | GC04P054657 | 16.38 |
| 58 | CTLA4 | Cytotoxic T-Lymphocyte Associated Protein 4 | Protein Coding | 45 | GC02P203867 | 16.35 |
| 59 | CIITA | Class II Major Histocompatibility Complex Transactivator | Protein Coding | 45 | GC16P010879 | 16.14 |
| 60 | DSP | Desmoplakin | Protein Coding | 49 | GC06P007541 | 15.99 |
| 61 | HRAS | HRas Proto-Oncogene, GTPase | Protein Coding | 52 | GC11M000635 | 15.98 |
| 62 | CASP3 | Caspase 3 | Protein Coding | 50 | GC04M184627 | 15.86 |
| 63 | MMP1 | Matrix Metallopeptidase 1 | Protein Coding | 51 | GC11M102810 | 15.82 |
| 64 | HFE | Homeostatic Iron Regulator | Protein Coding | 43 | GC06P026087 | 15.55 |
| 65 | CD86 | CD86 Molecule | Protein Coding | 43 | GC03P122055 | 15.49 |
| 66 | IRF7 | Interferon Regulatory Factor 7 | Protein Coding | 46 | GC11M000612 | 15.39 |
| 67 | FOXP3 | Forkhead Box P3 | Protein Coding | 46 | GC0XM049250 | 15.34 |
| 68 | SLC22A5 | Solute Carrier Family 22 Member 5 | Protein Coding | 46 | GC05P132369 | 15.3 |
| 69 | CCND1 | Cyclin D1 | Protein Coding | 52 | GC11P069641 | 15.11 |
| 70 | MIR155 | MicroRNA 155 | RNA Gene | 18 | GC21P025573 | 15.08 |
| 71 | TLR5 | Toll Like Receptor 5 | Protein Coding | 47 | GC01M223109 | 15.04 |
| 72 | MYH6 | Myosin Heavy Chain 6 | Protein Coding | 45 | GC14M023380 | 15.01 |
| 73 | RAF1 | Raf-1 Proto-Oncogene, Serine/Threonine Kinase | Protein Coding | 54 | GC03M012583 | 14.9 |
| 74 | CD274 | CD274 Molecule | Protein Coding | 44 | GC09P005450 | 14.82 |
| 75 | MIR21 | MicroRNA 21 | RNA Gene | 24 | GC17P059841 | 14.66 |
| 76 | SAMHD1 | SAM And HD Domain Containing Deoxynucleoside Triphosphate Triphosphohydrolase 1 | Protein Coding | 43 | GC20M036890 | 14.61 |
| 77 | PIK3C2A | Phosphatidylinositol-4-Phosphate 3-Kinase Catalytic Subunit Type 2 Alpha | Protein Coding | 47 | GC11M017191 | 14.57 |
| 78 | CD55 | CD55 Molecule (Cromer Blood Group) | Protein Coding | 47 | GC01P207321 | 14.09 |
| 79 | ACE | Angiotensin I Converting Enzyme | Protein Coding | 49 | GC17P063477 | 14.09 |
| 80 | ELANE | Elastase, Neutrophil Expressed | Protein Coding | 46 | GC19P000854 | 14.05 |
| 81 | DES | Desmin | Protein Coding | 48 | GC02P219418 | 13.96 |
| 82 | SLC17A5 | Solute Carrier Family 17 Member 5 | Protein Coding | 44 | GC06M073593 | 13.94 |
| 83 | IL13 | Interleukin 13 | Protein Coding | 44 | GC05P132656 | 13.86 |
| 84 | MBL2 | Mannose Binding Lectin 2 | Protein Coding | 47 | GC10M052760 | 13.78 |
| 85 | SOD1 | Superoxide Dismutase 1 | Protein Coding | 51 | GC21P031659 | 13.68 |
| 86 | MB | Myoglobin | Protein Coding | 43 | GC22M035606 | 13.6 |
| 87 | TLR9 | Toll Like Receptor 9 | Protein Coding | 45 | GC03M052222 | 13.59 |
| 88 | NRAS | NRAS Proto-Oncogene, GTPase | Protein Coding | 50 | GC01M114704 | 13.53 |
| 89 | AKT1 | AKT Serine/Threonine Kinase 1 | Protein Coding | 54 | GC14M104769 | 13.39 |
| 90 | PTPN11 | Protein Tyrosine Phosphatase Non-Receptor Type 11 | Protein Coding | 53 | GC12P112418 | 13.38 |
| 91 | CTPS1 | CTP Synthase 1 | Protein Coding | 44 | GC01P040979 | 13.35 |
| 92 | CD40 | CD40 Molecule | Protein Coding | 48 | GC20P046118 | 13.33 |
| 93 | CCL4 | C-C Motif Chemokine Ligand 4 | Protein Coding | 40 | GC17P036103 | 13.32 |
| 94 | TGFB1 | Transforming Growth Factor Beta 1 | Protein Coding | 52 | GC19M041301 | 13.28 |
| 95 | CCR6 | C-C Motif Chemokine Receptor 6 | Protein Coding | 44 | GC06P167111 | 13.23 |
| 96 | TNFRSF1A | TNF Receptor Superfamily Member 1A | Protein Coding | 49 | GC12M006328 | 13.21 |
| 97 | DAG1 | Dystroglycan 1 | Protein Coding | 46 | GC03P049482 | 13.21 |
| 98 | F2 | Coagulation Factor II, Thrombin | Protein Coding | 48 | GC11P046720 | 13.19 |
| 99 | MIR146A | MicroRNA 146a | RNA Gene | 22 | GC05P160485 | 13.05 |
| 100 | PIK3CA | Phosphatidylinositol-4,5-Bisphosphate 3-Kinase Catalytic Subunit Alpha | Protein Coding | 52 | GC03P179148 | 13.03 |
| 101 | NOS2 | Nitric Oxide Synthase 2 | Protein Coding | 49 | GC17M027756 | 12.89 |
| 102 | VCAM1 | Vascular Cell Adhesion Molecule 1 | Protein Coding | 45 | GC01P100719 | 12.82 |
| 103 | MAVS | Mitochondrial Antiviral Signaling Protein | Protein Coding | 40 | GC20P003827 | 12.78 |
| 104 | GPX4 | Glutathione Peroxidase 4 | Protein Coding | 47 | GC19P001103 | 12.74 |
| 105 | IL1A | Interleukin 1 Alpha | Protein Coding | 44 | GC02M112773 | 12.56 |
| 106 | FOS | Fos Proto-Oncogene, AP-1 Transcription Factor Subunit | Protein Coding | 50 | GC14P075278 | 12.49 |
| 107 | MYH7 | Myosin Heavy Chain 7 | Protein Coding | 47 | GC14M023412 | 12.4 |
| 108 | FASLG | Fas Ligand | Protein Coding | 47 | GC01P172628 | 12.31 |
| 109 | FMR1 | FMRP Translational Regulator 1 | Protein Coding | 44 | GC0XP147913 | 12.23 |
| 110 | B2M | Beta-2-Microglobulin | Protein Coding | 48 | GC15P044711 | 12.15 |
| 111 | IL2RA | Interleukin 2 Receptor Subunit Alpha | Protein Coding | 50 | GC10M006010 | 12.14 |
| 112 | CD80 | CD80 Molecule | Protein Coding | 41 | GC03M119524 | 12.05 |
| 113 | TLR7 | Toll Like Receptor 7 | Protein Coding | 46 | GC0XP012867 | 11.97 |
| 114 | H2AC18 | H2A Clustered Histone 18 | Protein Coding | 26 | GC01M149961 | 11.95 |
| 115 | HMOX1 | Heme Oxygenase 1 | Protein Coding | 52 | GC22P035380 | 11.81 |
| 116 | BST2 | Bone Marrow Stromal Cell Antigen 2 | Protein Coding | 39 | GC19M017403 | 11.7 |
| 117 | MYC | MYC Proto-Oncogene, BHLH Transcription Factor | Protein Coding | 51 | GC08P127735 | 11.54 |
| 118 | TTN | Titin | Protein Coding | 47 | GC02M178525 | 11.44 |
| 119 | PDCD1 | Programmed Cell Death 1 | Protein Coding | 48 | GC02M241849 | 11.43 |
| 120 | NPPB | Natriuretic Peptide B | Protein Coding | 44 | GC01M011858 | 11.43 |
| 121 | MMP9 | Matrix Metallopeptidase 9 | Protein Coding | 52 | GC20P046008 | 11.33 |
| 122 | IL5 | Interleukin 5 | Protein Coding | 44 | GC05M132541 | 11.32 |
| 123 | HLA-DQA1 | Major Histocompatibility Complex, Class II, DQ Alpha 1 | Protein Coding | 42 | GC06P047340 | 11.16 |
| 124 | LCK | LCK Proto-Oncogene, Src Family Tyrosine Kinase | Protein Coding | 52 | GC01P032251 | 11.11 |
| 125 | CD247 | CD247 Molecule | Protein Coding | 49 | GC01M167399 | 11.06 |
| 126 | HLA-A | Major Histocompatibility Complex, Class I, A | Protein Coding | 46 | GC06P047265 | 11 |
| 127 | TXN | Thioredoxin | Protein Coding | 45 | GC09M110243 | 10.92 |
| 128 | ICOSLG | Inducible T Cell Costimulator Ligand | Protein Coding | 39 | GC21M044222 | 10.89 |
| 129 | ITGB2 | Integrin Subunit Beta 2 | Protein Coding | 50 | GC21M044885 | 10.72 |
| 130 | VEGFA | Vascular Endothelial Growth Factor A | Protein Coding | 48 | GC06P043770 | 10.71 |
| 131 | MIR221 | MicroRNA 221 | RNA Gene | 20 | GC0XM045746 | 10.7 |
| 132 | NFKB2 | Nuclear Factor Kappa B Subunit 2 | Protein Coding | 52 | GC10P102394 | 10.57 |
| 133 | IVNS1ABP | Influenza Virus NS1A Binding Protein | Protein Coding | 39 | GC01M185295 | 10.46 |
| 134 | MIR223 | MicroRNA 223 | RNA Gene | 21 | GC0XP066018 | 10.36 |
| 135 | CD68 | CD68 Molecule | Protein Coding | 40 | GC17P007579 | 10.33 |
| 136 | TLR8 | Toll Like Receptor 8 | Protein Coding | 47 | GC0XP012924 | 10.32 |
| 137 | CXCL9 | C-X-C Motif Chemokine Ligand 9 | Protein Coding | 39 | GC04M076001 | 10.27 |
| 138 | SRC | SRC Proto-Oncogene, Non-Receptor Tyrosine Kinase | Protein Coding | 51 | GC20P037344 | 10.22 |
| 139 | CAT | Catalase | Protein Coding | 50 | GC11P034460 | 10.17 |
| 140 | CSF3 | Colony Stimulating Factor 3 | Protein Coding | 40 | GC17P040015 | 10.16 |
| 141 | CSF2 | Colony Stimulating Factor 2 | Protein Coding | 44 | GC05P132073 | 10.14 |
| 142 | IFNA2 | Interferon Alpha 2 | Protein Coding | 41 | GC09M021384 | 10.08 |
| 143 | RELA | RELA Proto-Oncogene, NF-KB Subunit | Protein Coding | 50 | GC11M065653 | 10.04 |
| 144 | HSPD1 | Heat Shock Protein Family D (Hsp60) Member 1 | Protein Coding | 47 | GC02M197486 | 10.02 |
| 145 | EGFR | Epidermal Growth Factor Receptor | Protein Coding | 54 | GC07P055019 | 10.01 |
| 146 | HGF | Hepatocyte Growth Factor | Protein Coding | 52 | GC07M081699 | 9.97 |
| 147 | MIR222 | MicroRNA 222 | RNA Gene | 21 | GC0XM045747 | 9.89 |
| 148 | SDHA | Succinate Dehydrogenase Complex Flavoprotein Subunit A | Protein Coding | 46 | GC05P000208 | 9.87 |
| 149 | F3 | Coagulation Factor III, Tissue Factor | Protein Coding | 45 | GC01M094530 | 9.85 |
| 150 | PPARG | Peroxisome Proliferator Activated Receptor Gamma | Protein Coding | 52 | GC03P012287 | 9.7 |
| 151 | CASP9 | Caspase 9 | Protein Coding | 48 | GC01M015491 | 9.64 |
| 152 | HLA-DRB4 | Major Histocompatibility Complex, Class II, DR Beta 4 | Protein Coding | 30 | GC06Mo03851 | 9.54 |
| 153 | EIF2S1 | Eukaryotic Translation Initiation Factor 2 Subunit Alpha | Protein Coding | 44 | GC14P067359 | 9.5 |
| 154 | IL12RB1 | Interleukin 12 Receptor Subunit Beta 1 | Protein Coding | 44 | GC19M018058 | 9.47 |
| 155 | BAX | BCL2 Associated X, Apoptosis Regulator | Protein Coding | 48 | GC19P048954 | 9.46 |
| 156 | ERBB2 | Erb-B2 Receptor Tyrosine Kinase 2 | Protein Coding | 54 | GC17P039687 | 9.46 |
| 157 | PML | PML Nuclear Body Scaffold | Protein Coding | 45 | GC15P073994 | 9.45 |
| 158 | SCN5A | Sodium Voltage-Gated Channel Alpha Subunit 5 | Protein Coding | 50 | GC03M038549 | 9.37 |
| 159 | SPP1 | Secreted Phosphoprotein 1 | Protein Coding | 46 | GC04P087975 | 9.34 |
| 160 | TMPO | Thymopoietin | Protein Coding | 45 | GC12P098515 | 9.32 |
| 161 | CCL11 | C-C Motif Chemokine Ligand 11 | Protein Coding | 43 | GC17P034285 | 9.32 |
| 162 | SP1 | Sp1 Transcription Factor | Protein Coding | 44 | GC12P053380 | 9.28 |
| 163 | INS | Insulin | Protein Coding | 48 | GC11M002159 | 9.21 |
| 164 | MAPK14 | Mitogen-Activated Protein Kinase 14 | Protein Coding | 51 | GC06P047451 | 9.2 |
| 165 | IL15 | Interleukin 15 | Protein Coding | 40 | GC04P141636 | 9.17 |
| 166 | IKBKG | Inhibitor Of Nuclear Factor Kappa B Kinase Regulatory Subunit Gamma | Protein Coding | 48 | GC0XP154541 | 9.16 |
| 167 | MYOM2 | Myomesin 2 | Protein Coding | 39 | GC08P002045 | 9.11 |
| 168 | NFKBIA | NFKB Inhibitor Alpha | Protein Coding | 50 | GC14M035401 | 9.07 |
| 169 | MIR34A | MicroRNA 34a | RNA Gene | 22 | GC01M009151 | 9.05 |
| 170 | TIMP1 | TIMP Metallopeptidase Inhibitor 1 | Protein Coding | 45 | GC0XP047583 | 9.04 |
| 171 | SERPINA3 | Serpin Family A Member 3 | Protein Coding | 43 | GC14P094612 | 9.04 |
| 172 | MET | MET Proto-Oncogene, Receptor Tyrosine Kinase | Protein Coding | 54 | GC07P116672 | 9.01 |
| 173 | BCL2 | BCL2 Apoptosis Regulator | Protein Coding | 51 | GC18M063123 | 8.96 |
| 174 | IL1RN | Interleukin 1 Receptor Antagonist | Protein Coding | 48 | GC02P115307 | 8.92 |
| 175 | CASP1 | Caspase 1 | Protein Coding | 50 | GC11M105025 | 8.8 |
| 176 | HLA-C | Major Histocompatibility Complex, Class I, C | Protein Coding | 44 | GC06M031272 | 8.8 |
| 177 | PSEN2 | Presenilin 2 | Protein Coding | 49 | GC01P226870 | 8.73 |
| 178 | FURIN | Furin, Paired Basic Amino Acid Cleaving Enzyme | Protein Coding | 46 | GC15P090868 | 8.69 |
| 179 | EGF | Epidermal Growth Factor | Protein Coding | 51 | GC04P109912 | 8.67 |
| 180 | TAZ | Tafazzin | Protein Coding | 44 | GC0XP154411 | 8.66 |
| 181 | ACTC1 | Actin Alpha Cardiac Muscle 1 | Protein Coding | 42 | GC15M034788 | 8.65 |
| 182 | AKT3 | AKT Serine/Threonine Kinase 3 | Protein Coding | 52 | GC01M243488 | 8.65 |
| 183 | RNASE3 | Ribonuclease A Family Member 3 | Protein Coding | 40 | GC14P020891 | 8.62 |
| 184 | AKT2 | AKT Serine/Threonine Kinase 2 | Protein Coding | 54 | GC19M040230 | 8.61 |
| 185 | NEU1 | Neuraminidase 1 | Protein Coding | 44 | GC06M031857 | 8.59 |
| 186 | EIF4G1 | Eukaryotic Translation Initiation Factor 4 Gamma 1 | Protein Coding | 45 | GC03P184314 | 8.57 |
| 187 | SERPINA1 | Serpin Family A Member 1 | Protein Coding | 49 | GC14M094376 | 8.5 |
| 188 | PTX3 | Pentraxin 3 | Protein Coding | 41 | GC03P157436 | 8.5 |
| 189 | MAPK1 | Mitogen-Activated Protein Kinase 1 | Protein Coding | 51 | GC22M021754 | 8.5 |
| 190 | SELL | Selectin L | Protein Coding | 42 | GC01M169690 | 8.41 |
| 191 | MIR29A | MicroRNA 29a | RNA Gene | 21 | GC07M130876 | 8.32 |
| 192 | RSAD2 | Radical S-Adenosyl Methionine Domain Containing 2 | Protein Coding | 40 | GC02P006865 | 8.31 |
| 193 | MIR19B1 | MicroRNA 19b-1 | RNA Gene | 17 | GC13P091435 | 8.29 |
| 194 | TSC2 | TSC Complex Subunit 2 | Protein Coding | 50 | GC16P002436 | 8.28 |
| 195 | TLR1 | Toll Like Receptor 1 | Protein Coding | 47 | GC04M038797 | 8.27 |
| 196 | SOD2 | Superoxide Dismutase 2 | Protein Coding | 51 | GC06M159669 | 8.22 |
| 197 | CDKN2A | Cyclin Dependent Kinase Inhibitor 2A | Protein Coding | 51 | GC09M021967 | 8.21 |
| 198 | GJA1 | Gap Junction Protein Alpha 1 | Protein Coding | 50 | GC06P121436 | 8.2 |
| 199 | IL2RB | Interleukin 2 Receptor Subunit Beta | Protein Coding | 48 | GC22M037125 | 8.16 |
| 200 | C3 | Complement C3 | Protein Coding | 47 | GC19M006677 | 8.11 |
| 201 | IDO1 | Indoleamine 2,3-Dioxygenase 1 | Protein Coding | 45 | GC08P039891 | 8.1 |
| 202 | MIR196A2 | MicroRNA 196a-2 | RNA Gene | 21 | GC12P054200 | 7.97 |
| 203 | CALR | Calreticulin | Protein Coding | 51 | GC19P012938 | 7.96 |
| 204 | GSN | Gelsolin | Protein Coding | 48 | GC09P121201 | 7.96 |
| 205 | HMGB1 | High Mobility Group Box 1 | Protein Coding | 44 | GC13M030456 | 7.93 |
| 206 | GNAS | GNAS Complex Locus | Protein Coding | 50 | GC20P058839 | 7.93 |
| 207 | IL16 | Interleukin 16 | Protein Coding | 42 | GC15P081159 | 7.92 |
| 208 | ADORA2A | Adenosine A2a Receptor | Protein Coding | 45 | GC22P024417 | 7.89 |
| 209 | SCO2 | Synthesis Of Cytochrome C Oxidase 2 | Protein Coding | 45 | GC22M050523 | 7.89 |
| 210 | IFITM1 | Interferon Induced Transmembrane Protein 1 | Protein Coding | 41 | GC11P000313 | 7.89 |
| 211 | IL10RA | Interleukin 10 Receptor Subunit Alpha | Protein Coding | 45 | GC11P117987 | 7.87 |
| 212 | MMP3 | Matrix Metallopeptidase 3 | Protein Coding | 51 | GC11M102835 | 7.85 |
| 213 | MPO | Myeloperoxidase | Protein Coding | 50 | GC17M058269 | 7.82 |
| 214 | HARS1 | Histidyl-TRNA Synthetase 1 | Protein Coding | 36 | GC05M140673 | 7.79 |
| 215 | MIR195 | MicroRNA 195 | RNA Gene | 19 | GC17M007018 | 7.79 |
| 216 | MIR452 | MicroRNA 452 | RNA Gene | 15 | GC0XM151959 | 7.79 |
| 217 | GAPDH | Glyceraldehyde-3-Phosphate Dehydrogenase | Protein Coding | 48 | GC12P008161 | 7.78 |
| 218 | IFITM2 | Interferon Induced Transmembrane Protein 2 | Protein Coding | 37 | GC11P000300 | 7.76 |
| 219 | JUN | Jun Proto-Oncogene, AP-1 Transcription Factor Subunit | Protein Coding | 49 | GC01M058780 | 7.75 |
| 220 | IKBKB | Inhibitor Of Nuclear Factor Kappa B Kinase Subunit Beta | Protein Coding | 52 | GC08P042271 | 7.75 |
| 221 | XPO1 | Exportin 1 | Protein Coding | 45 | GC02M061445 | 7.74 |
| 222 | MIR590 | MicroRNA 590 | RNA Gene | 18 | GC07P074191 | 7.71 |
| 223 | DNAJC3 | DnaJ Heat Shock Protein Family (Hsp40) Member C3 | Protein Coding | 40 | GC13P095677 | 7.68 |
| 224 | TMPRSS2 | Transmembrane Serine Protease 2 | Protein Coding | 44 | GC21M041464 | 7.65 |
| 225 | CD14 | CD14 Molecule | Protein Coding | 44 | GC05M140631 | 7.65 |
| 226 | NFKB1 | Nuclear Factor Kappa B Subunit 1 | Protein Coding | 52 | GC04P102501 | 7.59 |
| 227 | HP | Haptoglobin | Protein Coding | 44 | GC16P072089 | 7.59 |
| 228 | ARF1 | ADP Ribosylation Factor 1 | Protein Coding | 46 | GC01P228082 | 7.57 |
| 229 | MMP2 | Matrix Metallopeptidase 2 | Protein Coding | 53 | GC16P055390 | 7.55 |
| 230 | LTA | Lymphotoxin Alpha | Protein Coding | 42 | GC06P047303 | 7.51 |
| 231 | GSR | Glutathione-Disulfide Reductase | Protein Coding | 48 | GC08M030678 | 7.46 |
| 232 | CHUK | Component Of Inhibitor Of Nuclear Factor Kappa B Kinase Complex | Protein Coding | 52 | GC10M100188 | 7.42 |
| 233 | ITGA4 | Integrin Subunit Alpha 4 | Protein Coding | 48 | GC02P181456 | 7.4 |
| 234 | TRAF3 | TNF Receptor Associated Factor 3 | Protein Coding | 47 | GC14P104639 | 7.38 |
| 235 | MTOR | Mechanistic Target Of Rapamycin Kinase | Protein Coding | 54 | GC01M011106 | 7.31 |
| 236 | RNASEL | Ribonuclease L | Protein Coding | 44 | GC01M182542 | 7.29 |
| 237 | HSPA5 | Heat Shock Protein Family A (Hsp70) Member 5 | Protein Coding | 47 | GC09M125234 | 7.26 |
| 238 | CREB1 | CAMP Responsive Element Binding Protein 1 | Protein Coding | 48 | GC02P207529 | 7.26 |
| 239 | PTMA | Prothymosin Alpha | Protein Coding | 39 | GC02P231707 | 7.25 |
| 240 | TP63 | Tumor Protein P63 | Protein Coding | 48 | GC03P189598 | 7.25 |
| 241 | TLR10 | Toll Like Receptor 10 | Protein Coding | 40 | GC04M038773 | 7.24 |
| 242 | CXCL13 | C-X-C Motif Chemokine Ligand 13 | Protein Coding | 41 | GC04P077511 | 7.23 |
| 243 | FCGR2A | Fc Fragment Of IgG Receptor IIa | Protein Coding | 45 | GC01P161505 | 7.2 |
| 244 | NLRP3 | NLR Family Pyrin Domain Containing 3 | Protein Coding | 47 | GC01P247415 | 7.19 |
| 245 | IRF1 | Interferon Regulatory Factor 1 | Protein Coding | 47 | GC05M132481 | 7.17 |
| 246 | HSP90AA1 | Heat Shock Protein 90 Alpha Family Class A Member 1 | Protein Coding | 48 | GC14M102080 | 7.13 |
| 247 | IL1R1 | Interleukin 1 Receptor Type 1 | Protein Coding | 45 | GC02P102136 | 7.11 |
| 248 | MIF | Macrophage Migration Inhibitory Factor | Protein Coding | 49 | GC22P023894 | 7.05 |
| 249 | MIR148A | MicroRNA 148a | RNA Gene | 18 | GC07M025993 | 7.03 |
| 250 | TIMP2 | TIMP Metallopeptidase Inhibitor 2 | Protein Coding | 44 | GC17M078852 | 6.96 |
| 251 | ACTB | Actin Beta | Protein Coding | 49 | GC07M005527 | 6.96 |
| 252 | TLR6 | Toll Like Receptor 6 | Protein Coding | 44 | GC04M038828 | 6.94 |
| 253 | CXCL1 | C-X-C Motif Chemokine Ligand 1 | Protein Coding | 43 | GC04P073869 | 6.94 |
| 254 | DPYSL5 | Dihydropyrimidinase Like 5 | Protein Coding | 37 | GC02P026847 | 6.87 |
| 255 | IL3 | Interleukin 3 | Protein Coding | 44 | GC05P132060 | 6.85 |
| 256 | CD46 | CD46 Molecule | Protein Coding | 46 | GC01P207752 | 6.75 |
| 257 | PRODH | Proline Dehydrogenase 1 | Protein Coding | 45 | GC22M018912 | 6.74 |
| 258 | ACTG1 | Actin Gamma 1 | Protein Coding | 50 | GC17M081509 | 6.74 |
| 259 | LCN2 | Lipocalin 2 | Protein Coding | 43 | GC09P128149 | 6.68 |
| 260 | CACNA1S | Calcium Voltage-Gated Channel Subunit Alpha1 S | Protein Coding | 48 | GC01M201008 | 6.68 |
| 261 | ACTN2 | Actinin Alpha 2 | Protein Coding | 47 | GC01P236686 | 6.68 |
| 262 | CSRP3 | Cysteine And Glycine Rich Protein 3 | Protein Coding | 41 | GC11M019160 | 6.68 |
| 263 | FKTN | Fukutin | Protein Coding | 39 | GC09P105558 | 6.68 |
| 264 | CYP3A4 | Cytochrome P450 Family 3 Subfamily A Member 4 | Protein Coding | 48 | GC07M099759 | 6.68 |
| 265 | G6PD | Glucose-6-Phosphate Dehydrogenase | Protein Coding | 50 | GC0XM154531 | 6.64 |
| 266 | POMC | Proopiomelanocortin | Protein Coding | 48 | GC02M025160 | 6.62 |
| 267 | IGHE | Immunoglobulin Heavy Constant Epsilon | Protein Coding | 26 | GC14M109515 | 6.62 |
| 268 | ABCB7 | ATP Binding Cassette Subfamily B Member 7 | Protein Coding | 43 | GC0XM075053 | 6.55 |
| 269 | CXCL12 | C-X-C Motif Chemokine Ligand 12 | Protein Coding | 45 | GC10M044294 | 6.55 |
| 270 | LBR | Lamin B Receptor | Protein Coding | 47 | GC01M225401 | 6.55 |
| 271 | TSLP | Thymic Stromal Lymphopoietin | Protein Coding | 38 | GC05P111070 | 6.49 |
| 272 | TNFSF13B | TNF Superfamily Member 13b | Protein Coding | 45 | GC13P108251 | 6.48 |
| 273 | CCL26 | C-C Motif Chemokine Ligand 26 | Protein Coding | 37 | GC07M075769 | 6.46 |
| 274 | HLA-DRA | Major Histocompatibility Complex, Class II, DR Alpha | Protein Coding | 47 | GC06P032439 | 6.44 |
| 275 | IRF4 | Interferon Regulatory Factor 4 | Protein Coding | 43 | GC06P000391 | 6.43 |
| 276 | TH | Tyrosine Hydroxylase | Protein Coding | 51 | GC11M002163 | 6.39 |
| 277 | MIR214 | MicroRNA 214 | RNA Gene | 20 | GC01M172234 | 6.33 |
| 278 | SMAD2 | SMAD Family Member 2 | Protein Coding | 47 | GC18M047809 | 6.28 |
| 279 | HLA-E | Major Histocompatibility Complex, Class I, E | Protein Coding | 41 | GC06P047281 | 6.27 |
| 280 | ITGAV | Integrin Subunit Alpha V | Protein Coding | 46 | GC02P186589 | 6.27 |
| 281 | NOD2 | Nucleotide Binding Oligomerization Domain Containing 2 | Protein Coding | 48 | GC16P050693 | 6.25 |
| 282 | MIR379 | MicroRNA 379 | RNA Gene | 16 | GC14P104795 | 6.24 |
| 283 | CXCR3 | C-X-C Motif Chemokine Receptor 3 | Protein Coding | 44 | GC0XM071615 | 6.24 |
| 284 | IGF1 | Insulin Like Growth Factor 1 | Protein Coding | 50 | GC12M102395 | 6.23 |
| 285 | CXCL2 | C-X-C Motif Chemokine Ligand 2 | Protein Coding | 40 | GC04M074097 | 6.2 |
| 286 | RPS27A | Ribosomal Protein S27a | Protein Coding | 43 | GC02P055231 | 6.2 |
| 287 | MEFV | MEFV Innate Immuity Regulator, Pyrin | Protein Coding | 43 | GC16M003281 | 6.19 |
| 288 | ITGB3 | Integrin Subunit Beta 3 | Protein Coding | 49 | GC17P047254 | 6.18 |
| 289 | CD19 | CD19 Molecule | Protein Coding | 49 | GC16P029083 | 6.17 |
| 290 | ITGAM | Integrin Subunit Alpha M | Protein Coding | 46 | GC16P031550 | 6.15 |
| 291 | SOCS3 | Suppressor Of Cytokine Signaling 3 | Protein Coding | 44 | GC17M078356 | 6.11 |
| 292 | APOE | Apolipoprotein E | Protein Coding | 50 | GC19P044906 | 6.1 |
| 293 | CCR7 | C-C Motif Chemokine Receptor 7 | Protein Coding | 45 | GC17M040556 | 6.08 |
| 294 | MAPK3 | Mitogen-Activated Protein Kinase 3 | Protein Coding | 49 | GC16M030117 | 6.05 |
| 295 | CTSL | Cathepsin L | Protein Coding | 46 | GC09P087725 | 6.02 |
| 296 | CD34 | CD34 Molecule | Protein Coding | 43 | GC01M207880 | 6.01 |
| 297 | IL9 | Interleukin 9 | Protein Coding | 43 | GC05M135891 | 6 |
| 298 | KPNA1 | Karyopherin Subunit Alpha 1 | Protein Coding | 42 | GC03M122421 | 6 |
| 299 | ITGB1 | Integrin Subunit Beta 1 | Protein Coding | 50 | GC10M032900 | 5.97 |
| 300 | MUC1 | Mucin 1, Cell Surface Associated | Protein Coding | 47 | GC01M155185 | 5.96 |
| 301 | KPNA3 | Karyopherin Subunit Alpha 3 | Protein Coding | 43 | GC13M049699 | 5.93 |
| 302 | HAVCR1 | Hepatitis A Virus Cellular Receptor 1 | Protein Coding | 41 | GC05M157028 | 5.89 |
| 303 | THPO | Thrombopoietin | Protein Coding | 42 | GC03M184371 | 5.89 |
| 304 | KRT7 | Keratin 7 | Protein Coding | 41 | GC12P052232 | 5.85 |
| 305 | DEFB4A | Defensin Beta 4A | Protein Coding | 37 | GC08P007895 | 5.85 |
| 306 | TRIM25 | Tripartite Motif Containing 25 | Protein Coding | 44 | GC17M056836 | 5.83 |
| 307 | TNNT2 | Troponin T2, Cardiac Type | Protein Coding | 48 | GC01M201359 | 5.82 |
| 308 | CD70 | CD70 Molecule | Protein Coding | 43 | GC19M006583 | 5.82 |
| 309 | TNC | Tenascin C | Protein Coding | 48 | GC09M115019 | 5.81 |
| 310 | CD2 | CD2 Molecule | Protein Coding | 44 | GC01P116754 | 5.8 |
| 311 | SELE | Selectin E | Protein Coding | 44 | GC01M169722 | 5.79 |
| 312 | CDH2 | Cadherin 2 | Protein Coding | 50 | GC18M027950 | 5.79 |
| 313 | CLU | Clusterin | Protein Coding | 46 | GC08M027596 | 5.77 |
| 314 | CD28 | CD28 Molecule | Protein Coding | 47 | GC02P203706 | 5.77 |
| 315 | RTRAF | RNA Transcription, Translation And Transport Factor | Protein Coding | 28 | GC14P051992 | 5.77 |
| 316 | ITK | IL2 Inducible T Cell Kinase | Protein Coding | 51 | GC05P157158 | 5.74 |
| 317 | ADAM17 | ADAM Metallopeptidase Domain 17 | Protein Coding | 51 | GC02M009488 | 5.74 |
| 318 | NOTCH1 | Notch Receptor 1 | Protein Coding | 51 | GC09M136602 | 5.72 |
| 319 | PRDM10 | PR/SET Domain 10 | Protein Coding | 34 | GC11M129899 | 5.71 |
| 320 | CCL7 | C-C Motif Chemokine Ligand 7 | Protein Coding | 41 | GC17P034270 | 5.7 |
| 321 | CASP7 | Caspase 7 | Protein Coding | 50 | GC10P113679 | 5.7 |
| 322 | HERC5 | HECT And RLD Domain Containing E3 Ubiquitin Protein Ligase 5 | Protein Coding | 39 | GC04P088457 | 5.7 |
| 323 | STAU1 | Staufen Double-Stranded RNA Binding Protein 1 | Protein Coding | 37 | GC20M049113 | 5.69 |
| 324 | ARG1 | Arginase 1 | Protein Coding | 50 | GC06P131473 | 5.68 |
| 325 | SRF | Serum Response Factor | Protein Coding | 41 | GC06P043171 | 5.66 |
| 326 | TYR | Tyrosinase | Protein Coding | 47 | GC11P089177 | 5.66 |
| 327 | KPNB1 | Karyopherin Subunit Beta 1 | Protein Coding | 43 | GC17P047649 | 5.66 |
| 328 | GZMB | Granzyme B | Protein Coding | 45 | GC14M024630 | 5.65 |
| 329 | CD79A | CD79a Molecule | Protein Coding | 46 | GC19P041877 | 5.64 |
| 330 | AIRE | Autoimmune Regulator | Protein Coding | 44 | GC21P044285 | 5.63 |
| 331 | MIR146B | MicroRNA 146b | RNA Gene | 19 | GC10P102436 | 5.62 |
| 332 | HIF1A | Hypoxia Inducible Factor 1 Subunit Alpha | Protein Coding | 47 | GC14P061695 | 5.62 |
| 333 | ITGA5 | Integrin Subunit Alpha 5 | Protein Coding | 48 | GC12M054396 | 5.59 |
| 334 | SMAD3 | SMAD Family Member 3 | Protein Coding | 49 | GC15P067063 | 5.57 |
| 335 | ACE2 | Angiotensin I Converting Enzyme 2 | Protein Coding | 48 | GC0XM015562 | 5.57 |
| 336 | IL11 | Interleukin 11 | Protein Coding | 41 | GC19M055364 | 5.57 |
| 337 | ELOB | Elongin B | Protein Coding | 32 | GC16M002772 | 5.56 |
| 338 | SERPINE1 | Serpin Family E Member 1 | Protein Coding | 50 | GC07P101127 | 5.55 |
| 339 | KARS1 | Lysyl-TRNA Synthetase 1 | Protein Coding | 38 | GC16M075628 | 5.54 |
| 340 | ENO1 | Enolase 1 | Protein Coding | 47 | GC01M008861 | 5.51 |
| 341 | RARB | Retinoic Acid Receptor Beta | Protein Coding | 50 | GC03P024830 | 5.5 |
| 342 | DNTT | DNA Nucleotidylexotransferase | Protein Coding | 42 | GC10P096304 | 5.47 |
| 343 | ELOC | Elongin C | Protein Coding | 33 | GC08M073939 | 5.44 |
| 344 | IFNAR1 | Interferon Alpha And Beta Receptor Subunit 1 | Protein Coding | 45 | GC21P033324 | 5.43 |
| 345 | MX2 | MX Dynamin Like GTPase 2 | Protein Coding | 38 | GC21P041361 | 5.42 |
| 346 | NPPA | Natriuretic Peptide A | Protein Coding | 46 | GC01M011846 | 5.42 |
| 347 | FCN2 | Ficolin 2 | Protein Coding | 40 | GC09P134864 | 5.37 |
| 348 | ODC1 | Ornithine Decarboxylase 1 | Protein Coding | 47 | GC02M010432 | 5.36 |
| 349 | MIR30A | MicroRNA 30a | RNA Gene | 20 | GC06M071403 | 5.33 |
| 350 | TGFBR1 | Transforming Growth Factor Beta Receptor 1 | Protein Coding | 52 | GC09P099104 | 5.32 |
| 351 | MIR130A | MicroRNA 130a | RNA Gene | 20 | GC11P057641 | 5.32 |
| 352 | PPARA | Peroxisome Proliferator Activated Receptor Alpha | Protein Coding | 45 | GC22P046150 | 5.29 |
| 353 | DPP6 | Dipeptidyl Peptidase Like 6 | Protein Coding | 43 | GC07P153748 | 5.28 |
| 354 | TICAM1 | Toll Like Receptor Adaptor Molecule 1 | Protein Coding | 44 | GC19M004815 | 5.28 |
| 355 | ITGAL | Integrin Subunit Alpha L | Protein Coding | 45 | GC16P030472 | 5.27 |
| 356 | TGFB2 | Transforming Growth Factor Beta 2 | Protein Coding | 50 | GC01P218345 | 5.27 |
| 357 | CD58 | CD58 Molecule | Protein Coding | 40 | GC01M116514 | 5.24 |
| 358 | ADRB2 | Adrenoceptor Beta 2 | Protein Coding | 48 | GC05P148825 | 5.24 |
| 359 | TNFRSF4 | TNF Receptor Superfamily Member 4 | Protein Coding | 43 | GC01M001211 | 5.23 |
| 360 | NLRC4 | NLR Family CARD Domain Containing 4 | Protein Coding | 44 | GC02M032224 | 5.23 |
| 361 | PRL | Prolactin | Protein Coding | 44 | GC06M022230 | 5.23 |
| 362 | KPNA5 | Karyopherin Subunit Alpha 5 | Protein Coding | 39 | GC06P116681 | 5.2 |
| 363 | NUP98 | Nucleoporin 98 And 96 Precursor | Protein Coding | 42 | GC11M003671 | 5.18 |
| 364 | CD5 | CD5 Molecule | Protein Coding | 41 | GC11P061114 | 5.17 |
| 365 | PHC3 | Polyhomeotic Homolog 3 | Protein Coding | 38 | GC03M170086 | 5.15 |
| 366 | NPY | Neuropeptide Y | Protein Coding | 45 | GC07P024290 | 5.15 |
| 367 | VWF | Von Willebrand Factor | Protein Coding | 48 | GC12M005917 | 5.14 |
| 368 | ABL2 | ABL Proto-Oncogene 2, Non-Receptor Tyrosine Kinase | Protein Coding | 45 | GC01M179108 | 5.13 |
| 369 | MIR210 | MicroRNA 210 | RNA Gene | 21 | GC11M000674 | 5.11 |
| 370 | AMPH | Amphiphysin | Protein Coding | 44 | GC07M038666 | 5.11 |
| 371 | CANX | Calnexin | Protein Coding | 44 | GC05P179678 | 5.09 |
| 372 | CDC42 | Cell Division Cycle 42 | Protein Coding | 51 | GC01P022057 | 5.07 |
| 373 | LAMA2 | Laminin Subunit Alpha 2 | Protein Coding | 43 | GC06P128863 | 5.07 |
| 374 | THBD | Thrombomodulin | Protein Coding | 44 | GC20M023026 | 5.05 |
| 375 | CXCL6 | C-X-C Motif Chemokine Ligand 6 | Protein Coding | 40 | GC04P073837 | 5.04 |
| 376 | TNFRSF1B | TNF Receptor Superfamily Member 1B | Protein Coding | 47 | GC01P012167 | 5.03 |
| 377 | MIR199A1 | MicroRNA 199a-1 | RNA Gene | 18 | GC19M010792 | 5.03 |
| 378 | CR1 | Complement C3b/C4b Receptor 1 (Knops Blood Group) | Protein Coding | 44 | GC01P207496 | 5.03 |
| 379 | FLT1 | Fms Related Receptor Tyrosine Kinase 1 | Protein Coding | 51 | GC13M028300 | 4.99 |
| 380 | CHD6 | Chromodomain Helicase DNA Binding Protein 6 | Protein Coding | 37 | GC20M041402 | 4.98 |
| 381 | CCL17 | C-C Motif Chemokine Ligand 17 | Protein Coding | 38 | GC16P057400 | 4.95 |
| 382 | MASP2 | Mannan Binding Lectin Serine Peptidase 2 | Protein Coding | 44 | GC01M011026 | 4.94 |
| 383 | ABCB1 | ATP Binding Cassette Subfamily B Member 1 | Protein Coding | 51 | GC07M087504 | 4.94 |
| 384 | CR2 | Complement C3d Receptor 2 | Protein Coding | 44 | GC01P207454 | 4.91 |
| 385 | HLA-G | Major Histocompatibility Complex, Class I, G | Protein Coding | 44 | GC06P047256 | 4.89 |
| 386 | ADM | Adrenomedullin | Protein Coding | 44 | GC11P010304 | 4.89 |
| 387 | CD27 | CD27 Molecule | Protein Coding | 45 | GC12P008144 | 4.89 |
| 388 | IL1RAPL2 | Interleukin 1 Receptor Accessory Protein Like 2 | Protein Coding | 37 | GC0XP104566 | 4.89 |
| 389 | VDAC1 | Voltage Dependent Anion Channel 1 | Protein Coding | 45 | GC05M133975 | 4.89 |
| 390 | LIF | LIF Interleukin 6 Family Cytokine | Protein Coding | 43 | GC22M030240 | 4.89 |
| 391 | PRKCE | Protein Kinase C Epsilon | Protein Coding | 50 | GC02P045651 | 4.85 |
| 392 | FLNC | Filamin C | Protein Coding | 44 | GC07P128830 | 4.83 |
| 393 | CPSF4 | Cleavage And Polyadenylation Specific Factor 4 | Protein Coding | 40 | GC07P099438 | 4.79 |
| 394 | EDN1 | Endothelin 1 | Protein Coding | 47 | GC06P012290 | 4.78 |
| 395 | TARS1 | Threonyl-TRNA Synthetase 1 | Protein Coding | 36 | GC05P033441 | 4.78 |
| 396 | DEFA1 | Defensin Alpha 1 | Protein Coding | 40 | GC08M006977 | 4.76 |
| 397 | MORC3 | MORC Family CW-Type Zinc Finger 3 | Protein Coding | 36 | GC21P036320 | 4.74 |
| 398 | MIR132 | MicroRNA 132 | RNA Gene | 21 | GC17M002049 | 4.74 |
| 399 | MIR199B | MicroRNA 199b | RNA Gene | 19 | GC09M128244 | 4.74 |
| 400 | TF | Transferrin | Protein Coding | 49 | GC03P133666 | 4.72 |
| 401 | RRP1B | Ribosomal RNA Processing 1B | Protein Coding | 35 | GC21P043659 | 4.72 |
| 402 | TREM1 | Triggering Receptor Expressed On Myeloid Cells 1 | Protein Coding | 41 | GC06M041267 | 4.71 |
| 403 | TGFBR2 | Transforming Growth Factor Beta Receptor 2 | Protein Coding | 51 | GC03P030623 | 4.71 |
| 404 | KITLG | KIT Ligand | Protein Coding | 44 | GC12M088492 | 4.71 |
| 405 | HLA-DPB1 | Major Histocompatibility Complex, Class II, DP Beta 1 | Protein Coding | 43 | GC06P047346 | 4.71 |
| 406 | CREB3 | CAMP Responsive Element Binding Protein 3 | Protein Coding | 38 | GC09P035722 | 4.69 |
| 407 | PAF1 | PAF1 Homolog, Paf1/RNA Polymerase II Complex Component | Protein Coding | 36 | GC19M039385 | 4.69 |
| 408 | APLNR | Apelin Receptor | Protein Coding | 43 | GC11M057233 | 4.68 |
| 409 | LTF | Lactotransferrin | Protein Coding | 43 | GC03M046435 | 4.68 |
| 410 | NXF1 | Nuclear RNA Export Factor 1 | Protein Coding | 40 | GC11M063441 | 4.68 |
| 411 | IGLON5 | IgLON Family Member 5 | Protein Coding | 33 | GC19P051311 | 4.68 |
| 412 | FCGR2B | Fc Fragment Of IgG Receptor IIb | Protein Coding | 47 | GC01P161663 | 4.62 |
| 413 | SLC25A6 | Solute Carrier Family 25 Member 6 | Protein Coding | 43 | GC0XM001386 | 4.58 |
| 414 | MIR125A | MicroRNA 125a | RNA Gene | 21 | GC19P051720 | 4.57 |
| 415 | TRAF6 | TNF Receptor Associated Factor 6 | Protein Coding | 47 | GC11M036467 | 4.56 |
| 416 | FHL2 | Four And A Half LIM Domains 2 | Protein Coding | 45 | GC02M105343 | 4.56 |
| 417 | ABCC1 | ATP Binding Cassette Subfamily C Member 1 | Protein Coding | 47 | GC16P015949 | 4.55 |
| 418 | KCNJ12 | Potassium Inwardly Rectifying Channel Subfamily J Member 12 | Protein Coding | 43 | GC17P026750 | 4.55 |
| 419 | ITGA2 | Integrin Subunit Alpha 2 | Protein Coding | 45 | GC05P052989 | 4.51 |
| 420 | PRKCA | Protein Kinase C Alpha | Protein Coding | 50 | GC17P066302 | 4.51 |
| 421 | SMU1 | SMU1 DNA Replication Regulator And Spliceosomal Factor | Protein Coding | 36 | GC09M033041 | 4.51 |
| 422 | RAC1 | Rac Family Small GTPase 1 | Protein Coding | 49 | GC07P006380 | 4.49 |
| 423 | RASSF1 | Ras Association Domain Family Member 1 | Protein Coding | 44 | GC03M050329 | 4.49 |
| 424 | IL37 | Interleukin 37 | Protein Coding | 37 | GC02P115304 | 4.49 |
| 425 | C4A | Complement C4A (Rodgers Blood Group) | Protein Coding | 42 | GC06P047332 | 4.48 |
| 426 | UBE2N | Ubiquitin Conjugating Enzyme E2 N | Protein Coding | 47 | GC12M093406 | 4.48 |
| 427 | HLA-F | Major Histocompatibility Complex, Class I, F | Protein Coding | 40 | GC06P047251 | 4.48 |
| 428 | IL6ST | Interleukin 6 Signal Transducer | Protein Coding | 45 | GC05M055935 | 4.48 |
| 429 | PMP2 | Peripheral Myelin Protein 2 | Protein Coding | 43 | GC08M081440 | 4.47 |
| 430 | POLI | DNA Polymerase Iota | Protein Coding | 41 | GC18P054274 | 4.47 |
| 431 | MUC5B | Mucin 5B, Oligomeric Mucus/Gel-Forming | Protein Coding | 41 | GC11P001244 | 4.47 |
| 432 | PRTN3 | Proteinase 3 | Protein Coding | 44 | GC19P000840 | 4.46 |
| 433 | TIMP3 | TIMP Metallopeptidase Inhibitor 3 | Protein Coding | 45 | GC22P032800 | 4.44 |
| 434 | APOH | Apolipoprotein H | Protein Coding | 44 | GC17M066212 | 4.42 |
| 435 | MIR139 | MicroRNA 139 | RNA Gene | 19 | GC11M072615 | 4.42 |
| 436 | IL9R | Interleukin 9 Receptor | Protein Coding | 37 | GC0XP155997 | 4.39 |
| 437 | BAG3 | BAG Cochaperone 3 | Protein Coding | 44 | GC10P119651 | 4.38 |
| 438 | PNMA2 | PNMA Family Member 2 | Protein Coding | 36 | GC08M026504 | 4.38 |
| 439 | HLA-DPA1 | Major Histocompatibility Complex, Class II, DP Alpha 1 | Protein Coding | 40 | GC06M033064 | 4.37 |
| 440 | HAMP | Hepcidin Antimicrobial Peptide | Protein Coding | 44 | GC19P038216 | 4.37 |
| 441 | REN | Renin | Protein Coding | 48 | GC01M204154 | 4.37 |
| 442 | FYN | FYN Proto-Oncogene, Src Family Tyrosine Kinase | Protein Coding | 48 | GC06M111660 | 4.36 |
| 443 | NCAM1 | Neural Cell Adhesion Molecule 1 | Protein Coding | 45 | GC11P112961 | 4.34 |
| 444 | KCNQ1 | Potassium Voltage-Gated Channel Subfamily Q Member 1 | Protein Coding | 49 | GC11P002444 | 4.34 |
| 445 | CARTPT | CART Prepropeptide | Protein Coding | 44 | GC05P071719 | 4.32 |
| 446 | MRAP | Melanocortin 2 Receptor Accessory Protein | Protein Coding | 37 | GC21P032291 | 4.32 |
| 447 | NRSN1 | Neurensin 1 | Protein Coding | 35 | GC06P024126 | 4.32 |
| 448 | MIR148B | MicroRNA 148b | RNA Gene | 20 | GC12P054337 | 4.32 |
| 449 | POSTN | Periostin | Protein Coding | 43 | GC13M037562 | 4.27 |
| 450 | TRAPPC9 | Trafficking Protein Particle Complex 9 | Protein Coding | 39 | GC08M139728 | 4.27 |
| 451 | MAPK8 | Mitogen-Activated Protein Kinase 8 | Protein Coding | 50 | GC10P048306 | 4.26 |
| 452 | CCL22 | C-C Motif Chemokine Ligand 22 | Protein Coding | 38 | GC16P057359 | 4.25 |
| 453 | CD44 | CD44 Molecule (Indian Blood Group) | Protein Coding | 47 | GC11P035139 | 4.24 |
| 454 | CDH5 | Cadherin 5 | Protein Coding | 47 | GC16P066366 | 4.24 |
| 455 | HSPA4 | Heat Shock Protein Family A (Hsp70) Member 4 | Protein Coding | 41 | GC05P133051 | 4.24 |
| 456 | VCL | Vinculin | Protein Coding | 47 | GC10P073995 | 4.22 |
| 457 | ELN | Elastin | Protein Coding | 44 | GC07P074027 | 4.22 |
| 458 | CHD4 | Chromodomain Helicase DNA Binding Protein 4 | Protein Coding | 44 | GC12M006570 | 4.21 |
| 459 | TRIM21 | Tripartite Motif Containing 21 | Protein Coding | 42 | GC11M004384 | 4.2 |
| 460 | PSMB11 | Proteasome Subunit Beta 11 | Protein Coding | 33 | GC14P025489 | 4.2 |
| 461 | NOD1 | Nucleotide Binding Oligomerization Domain Containing 1 | Protein Coding | 44 | GC07M030424 | 4.19 |
| 462 | HAVCR2 | Hepatitis A Virus Cellular Receptor 2 | Protein Coding | 43 | GC05M157063 | 4.18 |
| 463 | SNRPA | Small Nuclear Ribonucleoprotein Polypeptide A | Protein Coding | 40 | GC19P040750 | 4.17 |
| 464 | DNAH8 | Dynein Axonemal Heavy Chain 8 | Protein Coding | 37 | GC06P047481 | 4.17 |
| 465 | WDR20 | WD Repeat Domain 20 | Protein Coding | 35 | GC14P104636 | 4.17 |
| 466 | CALCOCO2 | Calcium Binding And Coiled-Coil Domain 2 | Protein Coding | 39 | GC17P048830 | 4.17 |
| 467 | SGCD | Sarcoglycan Delta | Protein Coding | 45 | GC05P155686 | 4.15 |
| 468 | SYP | Synaptophysin | Protein Coding | 43 | GC0XM049187 | 4.15 |
| 469 | IFNGR1 | Interferon Gamma Receptor 1 | Protein Coding | 50 | GC06M137197 | 4.14 |
| 470 | ITGAX | Integrin Subunit Alpha X | Protein Coding | 44 | GC16P031570 | 4.12 |
| 471 | CFLAR | CASP8 And FADD Like Apoptosis Regulator | Protein Coding | 46 | GC02P201117 | 4.11 |
| 472 | SERPING1 | Serpin Family G Member 1 | Protein Coding | 46 | GC11P057597 | 4.1 |
| 473 | PPIG | Peptidylprolyl Isomerase G | Protein Coding | 41 | GC02P169584 | 4.1 |
| 474 | CTTN | Cortactin | Protein Coding | 43 | GC11P070398 | 4.1 |
| 475 | PGF | Placental Growth Factor | Protein Coding | 43 | GC14M074941 | 4.09 |
| 476 | HSPA1A | Heat Shock Protein Family A (Hsp70) Member 1A | Protein Coding | 43 | GC06P047326 | 4.09 |
| 477 | PIK3CG | Phosphatidylinositol-4,5-Bisphosphate 3-Kinase Catalytic Subunit Gamma | Protein Coding | 48 | GC07P106865 | 4.09 |
| 478 | NLRX1 | NLR Family Member X1 | Protein Coding | 40 | GC11P119166 | 4.09 |
| 479 | FN1 | Fibronectin 1 | Protein Coding | 50 | GC02M215360 | 4.08 |
| 480 | TGFB3 | Transforming Growth Factor Beta 3 | Protein Coding | 47 | GC14M075958 | 4.07 |
| 481 | CABIN1 | Calcineurin Binding Protein 1 | Protein Coding | 41 | GC22P024011 | 4.06 |
| 482 | CYSLTR1 | Cysteinyl Leukotriene Receptor 1 | Protein Coding | 43 | GC0XM078271 | 4.05 |
| 483 | MT-CO1 | Mitochondrially Encoded Cytochrome C Oxidase I | Protein Coding | 32 | GCMTP005906 | 4.05 |
| 484 | SOS1 | SOS Ras/Rac Guanine Nucleotide Exchange Factor 1 | Protein Coding | 47 | GC02M038981 | 4.04 |
| 485 | FKBP1A | FKBP Prolyl Isomerase 1A | Protein Coding | 46 | GC20M001369 | 4.04 |
| 486 | RPS6KB1 | Ribosomal Protein S6 Kinase B1 | Protein Coding | 49 | GC17P059893 | 4.04 |
| 487 | MIR145 | MicroRNA 145 | RNA Gene | 21 | GC05P149430 | 4.03 |
| 488 | CAMP | Cathelicidin Antimicrobial Peptide | Protein Coding | 41 | GC03P048266 | 4.03 |
| 489 | NKX2-1 | NK2 Homeobox 1 | Protein Coding | 46 | GC14M036516 | 4.03 |
| 490 | LMNA | Lamin A/C | Protein Coding | 47 | GC01P156082 | 4.02 |
| 491 | RN7SL1 | RNA Component Of Signal Recognition Particle 7SL1 | RNA Gene | 17 | GC14P049630 | 4.02 |
| 492 | IK | IK Cytokine | Protein Coding | 38 | GC05P143020 | 4.01 |
| 493 | ADAMTS13 | ADAM Metallopeptidase With Thrombospondin Type 1 Motif 13 | Protein Coding | 45 | GC09P133414 | 4.01 |
| 494 | RET | Ret Proto-Oncogene | Protein Coding | 53 | GC10P043081 | 4 |
| 495 | TSPO | Translocator Protein | Protein Coding | 43 | GC22P043151 | 4 |
| 496 | ATP2A2 | ATPase Sarcoplasmic/Endoplasmic Reticulum Ca2+ Transporting 2 | Protein Coding | 51 | GC12P110280 | 3.99 |
| 497 | FCN3 | Ficolin 3 | Protein Coding | 41 | GC01M027379 | 3.98 |
| 498 | TG | Thyroglobulin | Protein Coding | 42 | GC08P132866 | 3.97 |
| 499 | RYR1 | Ryanodine Receptor 1 | Protein Coding | 47 | GC19P038528 | 3.97 |
| 500 | CLMP | CXADR Like Membrane Protein | Protein Coding | 40 | GC11M123069 | 3.95 |
| 501 | GARS1 | Glycyl-TRNA Synthetase 1 | Protein Coding | 36 | GC07P030595 | 3.94 |
| 502 | PGD | Phosphogluconate Dehydrogenase | Protein Coding | 47 | GC01P010398 | 3.93 |
| 503 | AFF2 | AF4/FMR2 Family Member 2 | Protein Coding | 41 | GC0XP148500 | 3.93 |
| 504 | PLEKHM2 | Pleckstrin Homology And RUN Domain Containing M2 | Protein Coding | 35 | GC01P015691 | 3.93 |
| 505 | C1S | Complement C1s | Protein Coding | 45 | GC12P008238 | 3.92 |
| 506 | TPX2 | TPX2 Microtubule Nucleation Factor | Protein Coding | 41 | GC20P031739 | 3.92 |
| 507 | CACNA1D | Calcium Voltage-Gated Channel Subunit Alpha1 D | Protein Coding | 47 | GC03P053328 | 3.89 |
| 508 | SRA1 | Steroid Receptor RNA Activator 1 | Protein Coding | 37 | GC05M140537 | 3.89 |
| 509 | MIR154 | MicroRNA 154 | RNA Gene | 18 | GC14P104778 | 3.89 |
| 510 | MHRT | Myosin Heavy Chain Associated RNA Transcript | RNA Gene | 10 | GC14P025365 | 3.89 |
| 511 | CTF1 | Cardiotrophin 1 | Protein Coding | 39 | GC16P030910 | 3.86 |
| 512 | CKM | Creatine Kinase, M-Type | Protein Coding | 44 | GC19M045306 | 3.81 |
| 513 | BECN1 | Beclin 1 | Protein Coding | 46 | GC17M042810 | 3.8 |
| 514 | KCNJ11 | Potassium Inwardly Rectifying Channel Subfamily J Member 11 | Protein Coding | 47 | GC11M017364 | 3.77 |
| 515 | PABPN1 | Poly(A) Binding Protein Nuclear 1 | Protein Coding | 44 | GC14P025379 | 3.77 |
| 516 | TFAM | Transcription Factor A, Mitochondrial | Protein Coding | 43 | GC10P058385 | 3.76 |
| 517 | ENO2 | Enolase 2 | Protein Coding | 47 | GC12P006913 | 3.76 |
| 518 | CALB1 | Calbindin 1 | Protein Coding | 41 | GC08M090058 | 3.75 |
| 519 | CACNA1C | Calcium Voltage-Gated Channel Subunit Alpha1 C | Protein Coding | 48 | GC12P001970 | 3.75 |
| 520 | XCL1 | X-C Motif Chemokine Ligand 1 | Protein Coding | 37 | GC01P168576 | 3.75 |
| 521 | SYNE1 | Spectrin Repeat Containing Nuclear Envelope Protein 1 | Protein Coding | 41 | GC06M152121 | 3.74 |
| 522 | CYCS | Cytochrome C, Somatic | Protein Coding | 48 | GC07M025118 | 3.73 |
| 523 | SDHB | Succinate Dehydrogenase Complex Iron Sulfur Subunit B | Protein Coding | 47 | GC01M017020 | 3.73 |
| 524 | AGFG1 | ArfGAP With FG Repeats 1 | Protein Coding | 39 | GC02P227473 | 3.72 |
| 525 | HRC | Histidine Rich Calcium Binding Protein | Protein Coding | 36 | GC19M049151 | 3.71 |
| 526 | EIF4G2 | Eukaryotic Translation Initiation Factor 4 Gamma 2 | Protein Coding | 42 | GC11M010866 | 3.7 |
| 527 | SST | Somatostatin | Protein Coding | 42 | GC03M187668 | 3.68 |
| 528 | CHGA | Chromogranin A | Protein Coding | 42 | GC14P092923 | 3.67 |
| 529 | MDH2 | Malate Dehydrogenase 2 | Protein Coding | 48 | GC07P076048 | 3.66 |
| 530 | PPARGC1A | PPARG Coactivator 1 Alpha | Protein Coding | 46 | GC04M023755 | 3.65 |
| 531 | CHD3 | Chromodomain Helicase DNA Binding Protein 3 | Protein Coding | 41 | GC17P008060 | 3.63 |
| 532 | APOA1 | Apolipoprotein A1 | Protein Coding | 48 | GC11M116835 | 3.63 |
| 533 | SDHC | Succinate Dehydrogenase Complex Subunit C | Protein Coding | 44 | GC01P161314 | 3.63 |
| 534 | NOS3 | Nitric Oxide Synthase 3 | Protein Coding | 51 | GC07P150990 | 3.62 |
| 535 | PCNA | Proliferating Cell Nuclear Antigen | Protein Coding | 51 | GC20M005114 | 3.62 |
| 536 | FGF2 | Fibroblast Growth Factor 2 | Protein Coding | 47 | GC04P122826 | 3.62 |
| 537 | PARP1 | Poly(ADP-Ribose) Polymerase 1 | Protein Coding | 49 | GC01M226360 | 3.61 |
| 538 | RAC2 | Rac Family Small GTPase 2 | Protein Coding | 51 | GC22M037227 | 3.6 |
| 539 | SLC25A4 | Solute Carrier Family 25 Member 4 | Protein Coding | 48 | GC04P185143 | 3.6 |
| 540 | NMT1 | N-Myristoyltransferase 1 | Protein Coding | 43 | GC17P045052 | 3.59 |
| 541 | AGTR1 | Angiotensin II Receptor Type 1 | Protein Coding | 51 | GC03P148697 | 3.59 |
| 542 | JUP | Junction Plakoglobin | Protein Coding | 47 | GC17M041754 | 3.57 |
| 543 | CD69 | CD69 Molecule | Protein Coding | 40 | GC12M013857 | 3.57 |
| 544 | TACR1 | Tachykinin Receptor 1 | Protein Coding | 45 | GC02M075010 | 3.55 |
| 545 | DHPS | Deoxyhypusine Synthase | Protein Coding | 41 | GC19M012684 | 3.55 |
| 546 | CAV1 | Caveolin 1 | Protein Coding | 48 | GC07P116524 | 3.53 |
| 547 | NCR3 | Natural Cytotoxicity Triggering Receptor 3 | Protein Coding | 39 | GC06M031588 | 3.52 |
| 548 | CS | Citrate Synthase | Protein Coding | 44 | GC12M056271 | 3.52 |
| 549 | WASL | WASP Like Actin Nucleation Promoting Factor | Protein Coding | 41 | GC07M123681 | 3.51 |
| 550 | U2AF1 | U2 Small Nuclear RNA Auxiliary Factor 1 | Protein Coding | 41 | GC21M043092 | 3.51 |
| 551 | NAIP | NLR Family Apoptosis Inhibitory Protein | Protein Coding | 40 | GC05M070968 | 3.51 |
| 552 | SERPINC1 | Serpin Family C Member 1 | Protein Coding | 48 | GC01M174153 | 3.49 |
| 553 | TUT1 | Terminal Uridylyl Transferase 1, U6 SnRNA-Specific | Protein Coding | 35 | GC11M063404 | 3.48 |
| 554 | CARD9 | Caspase Recruitment Domain Family Member 9 | Protein Coding | 44 | GC09M136361 | 3.47 |
| 555 | ITGA2B | Integrin Subunit Alpha 2b | Protein Coding | 50 | GC17M044388 | 3.47 |
| 556 | ADCY10 | Adenylate Cyclase 10 | Protein Coding | 45 | GC01M167809 | 3.46 |
| 557 | INHA | Inhibin Subunit Alpha | Protein Coding | 43 | GC02P219569 | 3.45 |
| 558 | SLC27A1 | Solute Carrier Family 27 Member 1 | Protein Coding | 41 | GC19P023305 | 3.45 |
| 559 | PRG2 | Proteoglycan 2, Pro Eosinophil Major Basic Protein | Protein Coding | 40 | GC11M057386 | 3.45 |
| 560 | GATA4 | GATA Binding Protein 4 | Protein Coding | 48 | GC08P011676 | 3.45 |
| 561 | F7 | Coagulation Factor VII | Protein Coding | 47 | GC13P113105 | 3.45 |
| 562 | FBN1 | Fibrillin 1 | Protein Coding | 45 | GC15M048408 | 3.45 |
| 563 | NDUFA9 | NADH:Ubiquinone Oxidoreductase Subunit A9 | Protein Coding | 43 | GC12P004649 | 3.45 |
| 564 | MIR335 | MicroRNA 335 | RNA Gene | 18 | GC07P130496 | 3.43 |
| 565 | MIR495 | MicroRNA 495 | RNA Gene | 16 | GC14P104815 | 3.43 |
| 566 | KCNE1 | Potassium Voltage-Gated Channel Subfamily E Regulatory Subunit 1 | Protein Coding | 44 | GC21M034446 | 3.41 |
| 567 | ABL1 | ABL Proto-Oncogene 1, Non-Receptor Tyrosine Kinase | Protein Coding | 52 | GC09P130713 | 3.41 |
| 568 | DRD2 | Dopamine Receptor D2 | Protein Coding | 50 | GC11M113409 | 3.4 |
| 569 | MIR409 | MicroRNA 409 | RNA Gene | 18 | GC14P104806 | 3.4 |
| 570 | CACNG1 | Calcium Voltage-Gated Channel Auxiliary Subunit Gamma 1 | Protein Coding | 41 | GC17P067044 | 3.39 |
| 571 | CALB2 | Calbindin 2 | Protein Coding | 40 | GC16P071392 | 3.39 |
| 572 | BLK | BLK Proto-Oncogene, Src Family Tyrosine Kinase | Protein Coding | 51 | GC08P011486 | 3.39 |
| 573 | CD22 | CD22 Molecule | Protein Coding | 45 | GC19P035319 | 3.39 |
| 574 | HLA-DMA | Major Histocompatibility Complex, Class II, DM Alpha | Protein Coding | 40 | GC06M032950 | 3.38 |
| 575 | DNAJC19 | DnaJ Heat Shock Protein Family (Hsp40) Member C19 | Protein Coding | 41 | GC03M180983 | 3.38 |
| 576 | EDNRB | Endothelin Receptor Type B | Protein Coding | 49 | GC13M077895 | 3.37 |
| 577 | MIR369 | MicroRNA 369 | RNA Gene | 18 | GC14P104789 | 3.37 |
| 578 | MIR501 | MicroRNA 501 | RNA Gene | 16 | GC0XP050074 | 3.37 |
| 579 | MIR382 | MicroRNA 382 | RNA Gene | 15 | GC14P104799 | 3.37 |
| 580 | SLC6A2 | Solute Carrier Family 6 Member 2 | Protein Coding | 48 | GC16P055656 | 3.34 |
| 581 | KCNJ2 | Potassium Inwardly Rectifying Channel Subfamily J Member 2 | Protein Coding | 48 | GC17P070168 | 3.34 |
| 582 | MTM1 | Myotubularin 1 | Protein Coding | 44 | GC0XP150562 | 3.34 |
| 583 | SDHD | Succinate Dehydrogenase Complex Subunit D | Protein Coding | 44 | GC11P112087 | 3.34 |
| 584 | MAP4K3 | Mitogen-Activated Protein Kinase Kinase Kinase Kinase 3 | Protein Coding | 42 | GC02M039249 | 3.34 |
| 585 | CACNA2D2 | Calcium Voltage-Gated Channel Auxiliary Subunit Alpha2delta 2 | Protein Coding | 41 | GC03M050385 | 3.34 |
| 586 | MYPN | Myopalladin | Protein Coding | 41 | GC10P068106 | 3.34 |
| 587 | NEBL | Nebulette | Protein Coding | 39 | GC10M020779 | 3.34 |
| 588 | TXNDC15 | Thioredoxin Domain Containing 15 | Protein Coding | 37 | GC05P134873 | 3.34 |
| 589 | FOXD4 | Forkhead Box D4 | Protein Coding | 33 | GC09M000116 | 3.34 |
| 590 | MIR99B | MicroRNA 99b | RNA Gene | 18 | GC19P051692 | 3.34 |
| 591 | MIR376C | MicroRNA 376c | RNA Gene | 17 | GC14P104793 | 3.34 |
| 592 | MIR381 | MicroRNA 381 | RNA Gene | 17 | GC14P104797 | 3.34 |
| 593 | MIR487B | MicroRNA 487b | RNA Gene | 17 | GC14P104812 | 3.34 |
| 594 | MIR299 | MicroRNA 299 | RNA Gene | 16 | GC14P104780 | 3.34 |
| 595 | MIR362 | MicroRNA 362 | RNA Gene | 14 | GC0XP050072 | 3.34 |
| 596 | CXCL11 | C-X-C Motif Chemokine Ligand 11 | Protein Coding | 40 | GC04M076033 | 3.29 |
| 597 | IFN1@ | Interferon, Type 1, Cluster | Gene Cluster | 4 | GC09U990039 | 3.28 |
| 598 | SOCS1 | Suppressor Of Cytokine Signaling 1 | Protein Coding | 43 | GC16M011255 | 3.26 |
| 599 | MIR1-2 | MicroRNA 1-2 | RNA Gene | 17 | GC18M021828 | 3.22 |
| 600 | EEF1A1 | Eukaryotic Translation Elongation Factor 1 Alpha 1 | Protein Coding | 43 | GC06M073515 | 3.21 |
| 601 | SELP | Selectin P | Protein Coding | 45 | GC01M169558 | 3.2 |
| 602 | PSMC4 | Proteasome 26S Subunit, ATPase 4 | Protein Coding | 40 | GC19P039972 | 3.16 |
| 603 | GATA3 | GATA Binding Protein 3 | Protein Coding | 49 | GC10P008045 | 3.14 |
| 604 | RPLP0 | Ribosomal Protein Lateral Stalk Subunit P0 | Protein Coding | 41 | GC12M120196 | 3.11 |
| 605 | CFH | Complement Factor H | Protein Coding | 45 | GC01P196621 | 3.09 |
| 606 | SLC25A5 | Solute Carrier Family 25 Member 5 | Protein Coding | 44 | GC0XP119468 | 3.06 |
| 607 | IRAK1 | Interleukin 1 Receptor Associated Kinase 1 | Protein Coding | 50 | GC0XM154010 | 3.03 |
| 608 | VDR | Vitamin D Receptor | Protein Coding | 51 | GC12M047841 | 2.95 |
| 609 | BIRC5 | Baculoviral IAP Repeat Containing 5 | Protein Coding | 47 | GC17P078214 | 2.9 |
| 610 | FOXO3 | Forkhead Box O3 | Protein Coding | 44 | GC06P108559 | 2.89 |
| 611 | S100A8 | S100 Calcium Binding Protein A8 | Protein Coding | 42 | GC01M153391 | 2.88 |
| 612 | TDP2 | Tyrosyl-DNA Phosphodiesterase 2 | Protein Coding | 40 | GC06M024651 | 2.87 |
| 613 | PTK2B | Protein Tyrosine Kinase 2 Beta | Protein Coding | 49 | GC08P027311 | 2.86 |
| 614 | RBM38 | RNA Binding Motif Protein 38 | Protein Coding | 37 | GC20P057391 | 2.82 |
| 615 | LILRB1 | Leukocyte Immunoglobulin Like Receptor B1 | Protein Coding | 43 | GC19P055408 | 2.81 |
| 616 | PTK2 | Protein Tyrosine Kinase 2 | Protein Coding | 47 | GC08M140657 | 2.78 |
| 617 | MYBPC1 | Myosin Binding Protein C1 | Protein Coding | 43 | GC12P101568 | 2.78 |
| 618 | SGCA | Sarcoglycan Alpha | Protein Coding | 41 | GC17P050164 | 2.78 |
| 619 | BMP6 | Bone Morphogenetic Protein 6 | Protein Coding | 43 | GC06P007726 | 2.76 |
| 620 | DLD | Dihydrolipoamide Dehydrogenase | Protein Coding | 50 | GC07P107890 | 2.75 |
| 621 | PDGFC | Platelet Derived Growth Factor C | Protein Coding | 43 | GC04M156760 | 2.75 |
| 622 | SGCB | Sarcoglycan Beta | Protein Coding | 40 | GC04M052019 | 2.73 |
| 623 | PPA2 | Inorganic Pyrophosphatase 2 | Protein Coding | 41 | GC04M105369 | 2.71 |
| 624 | TNNT1 | Troponin T1, Slow Skeletal Type | Protein Coding | 43 | GC19M055132 | 2.71 |
| 625 | EYA4 | EYA Transcriptional Coactivator And Phosphatase 4 | Protein Coding | 43 | GC06P133240 | 2.64 |
| 626 | ITCH | Itchy E3 Ubiquitin Protein Ligase | Protein Coding | 45 | GC20P034363 | 2.62 |
| 627 | SGCG | Sarcoglycan Gamma | Protein Coding | 42 | GC13P023160 | 2.62 |
| 628 | LGALS1 | Galectin 1 | Protein Coding | 43 | GC22P037675 | 2.61 |
| 629 | HSPA8 | Heat Shock Protein Family A (Hsp70) Member 8 | Protein Coding | 47 | GC11M123057 | 2.61 |
| 630 | TJP1 | Tight Junction Protein 1 | Protein Coding | 43 | GC15M029699 | 2.6 |
| 631 | BCL6 | BCL6 Transcription Repressor | Protein Coding | 45 | GC03M187721 | 2.56 |
| 632 | LPL | Lipoprotein Lipase | Protein Coding | 49 | GC08P019901 | 2.56 |
| 633 | LGALS3 | Galectin 3 | Protein Coding | 44 | GC14P055124 | 2.55 |
| 634 | PSME3 | Proteasome Activator Subunit 3 | Protein Coding | 41 | GC17P042824 | 2.55 |
| 635 | EMD | Emerin | Protein Coding | 45 | GC0XP154379 | 2.54 |
| 636 | TNFRSF9 | TNF Receptor Superfamily Member 9 | Protein Coding | 44 | GC01M007915 | 2.52 |
| 637 | PRKACA | Protein Kinase CAMP-Activated Catalytic Subunit Alpha | Protein Coding | 51 | GC19M014092 | 2.5 |
| 638 | EGR1 | Early Growth Response 1 | Protein Coding | 44 | GC05P138465 | 2.49 |
| 639 | IL17RA | Interleukin 17 Receptor A | Protein Coding | 44 | GC22P017086 | 2.47 |
| 640 | HLA-DOB | Major Histocompatibility Complex, Class II, DO Beta | Protein Coding | 40 | GC06M032823 | 2.46 |
| 641 | MIR1-1 | MicroRNA 1-1 | RNA Gene | 19 | GC20P062893 | 2.43 |
| 642 | PLAT | Plasminogen Activator, Tissue Type | Protein Coding | 49 | GC08M042174 | 2.42 |
| 643 | IL18R1 | Interleukin 18 Receptor 1 | Protein Coding | 43 | GC02P102311 | 2.42 |
| 644 | PSG2 | Pregnancy Specific Beta-1-Glycoprotein 2 | Protein Coding | 35 | GC19M043064 | 2.42 |
| 645 | HLA-DOA | Major Histocompatibility Complex, Class II, DO Alpha | Protein Coding | 40 | GC06M033004 | 2.41 |
| 646 | CHRM2 | Cholinergic Receptor Muscarinic 2 | Protein Coding | 48 | GC07P136868 | 2.41 |
| 647 | HLA-DQA2 | Major Histocompatibility Complex, Class II, DQ Alpha 2 | Protein Coding | 37 | GC06P032741 | 2.41 |
| 648 | HLA-DMB | Major Histocompatibility Complex, Class II, DM Beta | Protein Coding | 41 | GC06M032934 | 2.41 |
| 649 | UBE2D1 | Ubiquitin Conjugating Enzyme E2 D1 | Protein Coding | 45 | GC10P058334 | 2.4 |
| 650 | MIR499A | MicroRNA 499a | RNA Gene | 21 | GC20P034990 | 2.38 |
| 651 | TUBB | Tubulin Beta Class I | Protein Coding | 49 | GC06P030720 | 2.38 |
| 652 | MDK | Midkine | Protein Coding | 43 | GC11P046402 | 2.37 |
| 653 | OSM | Oncostatin M | Protein Coding | 43 | GC22M030262 | 2.37 |
| 654 | TPM1 | Tropomyosin 1 | Protein Coding | 48 | GC15P073930 | 2.35 |
| 655 | BID | BH3 Interacting Domain Death Agonist | Protein Coding | 45 | GC22M017734 | 2.35 |
| 656 | GZMA | Granzyme A | Protein Coding | 42 | GC05P055102 | 2.35 |
| 657 | CUX2 | Cut Like Homeobox 2 | Protein Coding | 40 | GC12P111034 | 2.33 |
| 658 | FASN | Fatty Acid Synthase | Protein Coding | 49 | GC17M082078 | 2.32 |
| 659 | THBS2 | Thrombospondin 2 | Protein Coding | 45 | GC06M169215 | 2.3 |
| 660 | S100A9 | S100 Calcium Binding Protein A9 | Protein Coding | 43 | GC01P153357 | 2.29 |
| 661 | IL12B | Interleukin 12B | Protein Coding | 44 | GC05M159314 | 2.29 |
| 662 | HLA-DRB3 | Major Histocompatibility Complex, Class II, DR Beta 3 | Protein Coding | 28 | GC06Mn03715 | 2.29 |
| 663 | KRT78 | Keratin 78 | Protein Coding | 34 | GC12M052837 | 2.29 |
| 664 | ACTA1 | Actin Alpha 1, Skeletal Muscle | Protein Coding | 47 | GC01M229431 | 2.28 |
| 665 | HLA-DRB5 | Major Histocompatibility Complex, Class II, DR Beta 5 | Protein Coding | 40 | GC06M032683 | 2.27 |
| 666 | MIR374B | MicroRNA 374b | RNA Gene | 16 | GC0XM074227 | 2.27 |
| 667 | HSF1 | Heat Shock Transcription Factor 1 | Protein Coding | 44 | GC08P144291 | 2.25 |
| 668 | IFNGR2 | Interferon Gamma Receptor 2 | Protein Coding | 43 | GC21P033402 | 2.24 |
| 669 | TBX21 | T-Box Transcription Factor 21 | Protein Coding | 45 | GC17P047733 | 2.23 |
| 670 | VHL | Von Hippel-Lindau Tumor Suppressor | Protein Coding | 47 | GC03P010211 | 2.23 |
| 671 | DSG2 | Desmoglein 2 | Protein Coding | 45 | GC18P031498 | 2.23 |
| 672 | LAMP2 | Lysosomal Associated Membrane Protein 2 | Protein Coding | 44 | GC0XM120426 | 2.22 |
| 673 | EIF4G3 | Eukaryotic Translation Initiation Factor 4 Gamma 3 | Protein Coding | 42 | GC01M020806 | 2.22 |
| 674 | MYBPC3 | Myosin Binding Protein C3 | Protein Coding | 46 | GC11M061124 | 2.21 |
| 675 | SARDH | Sarcosine Dehydrogenase | Protein Coding | 42 | GC09M133663 | 2.2 |
| 676 | NGF | Nerve Growth Factor | Protein Coding | 50 | GC01M115285 | 2.19 |
| 677 | AMD1 | Adenosylmethionine Decarboxylase 1 | Protein Coding | 43 | GC06P110814 | 2.15 |
| 678 | SLC11A1 | Solute Carrier Family 11 Member 1 | Protein Coding | 47 | GC02P218382 | 2.14 |
| 679 | DNAJB1 | DnaJ Heat Shock Protein Family (Hsp40) Member B1 | Protein Coding | 44 | GC19M014514 | 2.14 |
| 680 | PSEN1 | Presenilin 1 | Protein Coding | 52 | GC14P073136 | 2.13 |
| 681 | DDIT3 | DNA Damage Inducible Transcript 3 | Protein Coding | 45 | GC12M057516 | 2.12 |
| 682 | CYP1A1 | Cytochrome P450 Family 1 Subfamily A Member 1 | Protein Coding | 47 | GC15M074719 | 2.11 |
| 683 | ANKRD1 | Ankyrin Repeat Domain 1 | Protein Coding | 42 | GC10M090912 | 2.1 |
| 684 | NEXN | Nexilin F-Actin Binding Protein | Protein Coding | 39 | GC01P077898 | 2.1 |
| 685 | PMP22 | Peripheral Myelin Protein 22 | Protein Coding | 40 | GC17M015229 | 2.1 |
| 686 | PLG | Plasminogen | Protein Coding | 48 | GC06P160702 | 2.07 |
| 687 | SRM | Spermidine Synthase | Protein Coding | 41 | GC01M011054 | 2.07 |
| 688 | CYP51A1 | Cytochrome P450 Family 51 Subfamily A Member 1 | Protein Coding | 44 | GC07M092112 | 2.07 |
| 689 | ACTN1 | Actinin Alpha 1 | Protein Coding | 50 | GC14M068874 | 2.06 |
| 690 | ANXA6 | Annexin A6 | Protein Coding | 43 | GC05M151077 | 2.06 |
| 691 | MMP8 | Matrix Metallopeptidase 8 | Protein Coding | 47 | GC11M102617 | 2.06 |
| 692 | ADRB1 | Adrenoceptor Beta 1 | Protein Coding | 48 | GC10P114044 | 2.06 |
| 693 | PSMD4 | Proteasome 26S Subunit, Non-ATPase 4 | Protein Coding | 44 | GC01P151227 | 2.05 |
| 694 | MICA | MHC Class I Polypeptide-Related Sequence A | Protein Coding | 39 | GC06P031399 | 2.04 |
| 695 | TNFSF9 | TNF Superfamily Member 9 | Protein Coding | 37 | GC19P006531 | 2.04 |
| 696 | NKX2-5 | NK2 Homeobox 5 | Protein Coding | 44 | GC05M173232 | 2.03 |
| 697 | LAMA1 | Laminin Subunit Alpha 1 | Protein Coding | 45 | GC18M006941 | 2.02 |
| 698 | SRSF2 | Serine And Arginine Rich Splicing Factor 2 | Protein Coding | 40 | GC17M076734 | 2 |
| 699 | HNRNPD | Heterogeneous Nuclear Ribonucleoprotein D | Protein Coding | 42 | GC04M082352 | 1.99 |
| 700 | ANGPT1 | Angiopoietin 1 | Protein Coding | 45 | GC08M107246 | 1.97 |
| 701 | DEFA5 | Defensin Alpha 5 | Protein Coding | 38 | GC08M007057 | 1.96 |
| 702 | PLAUR | Plasminogen Activator, Urokinase Receptor | Protein Coding | 44 | GC19M043646 | 1.96 |
| 703 | CISH | Cytokine Inducible SH2 Containing Protein | Protein Coding | 44 | GC03M050618 | 1.96 |
| 704 | GAS5 | Growth Arrest Specific 5 | RNA Gene | 23 | GC01M173947 | 1.94 |
| 705 | KNG1 | Kininogen 1 | Protein Coding | 44 | GC03P186717 | 1.93 |
| 706 | ANGPT2 | Angiopoietin 2 | Protein Coding | 44 | GC08M006499 | 1.92 |
| 707 | EIF4B | Eukaryotic Translation Initiation Factor 4B | Protein Coding | 41 | GC12P053006 | 1.92 |
| 708 | EIF3B | Eukaryotic Translation Initiation Factor 3 Subunit B | Protein Coding | 38 | GC07P002354 | 1.9 |
| 709 | RNPC3 | RNA Binding Region (RNP1, RRM) Containing 3 | Protein Coding | 35 | GC01P103525 | 1.89 |
| 710 | PSMC6 | Proteasome 26S Subunit, ATPase 6 | Protein Coding | 40 | GC14P052707 | 1.88 |
| 711 | CTH | Cystathionine Gamma-Lyase | Protein Coding | 50 | GC01P070411 | 1.87 |
| 712 | ITGB6 | Integrin Subunit Beta 6 | Protein Coding | 47 | GC02M160099 | 1.86 |
| 713 | ICOS | Inducible T Cell Costimulator | Protein Coding | 43 | GC02P203937 | 1.86 |
| 714 | GAB1 | GRB2 Associated Binding Protein 1 | Protein Coding | 44 | GC04P143336 | 1.86 |
| 715 | SREBF1 | Sterol Regulatory Element Binding Transcription Factor 1 | Protein Coding | 44 | GC17M017810 | 1.86 |
| 716 | OGN | Osteoglycin | Protein Coding | 39 | GC09M092383 | 1.85 |
| 717 | ADIPOQ | Adiponectin, C1Q And Collagen Domain Containing | Protein Coding | 45 | GC03P186842 | 1.85 |
| 718 | GP6 | Glycoprotein VI Platelet | Protein Coding | 44 | GC19M055013 | 1.84 |
| 719 | ACTA2 | Actin Alpha 2, Smooth Muscle | Protein Coding | 48 | GC10M088935 | 1.84 |
| 720 | MIR133B | MicroRNA 133b | RNA Gene | 21 | GC06P052148 | 1.83 |
| 721 | CCL19 | C-C Motif Chemokine Ligand 19 | Protein Coding | 41 | GC09M034692 | 1.82 |
| 722 | GLG1 | Golgi Glycoprotein 1 | Protein Coding | 37 | GC16M074448 | 1.82 |
| 723 | LOC102723407 | Immunoglobulin Heavy Variable 4-38-2-Like | Protein Coding | 9 | GC14P8B0041 | 1.81 |
| 724 | PROC | Protein C, Inactivator Of Coagulation Factors Va And VIIIa | Protein Coding | 49 | GC02P127418 | 1.81 |
| 725 | RPS6KA5 | Ribosomal Protein S6 Kinase A5 | Protein Coding | 45 | GC14M090847 | 1.8 |
| 726 | RUNX1 | RUNX Family Transcription Factor 1 | Protein Coding | 48 | GC21M034787 | 1.79 |
| 727 | EDNRA | Endothelin Receptor Type A | Protein Coding | 49 | GC04P147480 | 1.79 |
| 728 | AGT | Angiotensinogen | Protein Coding | 49 | GC01M230702 | 1.77 |
| 729 | PRKG1 | Protein Kinase CGMP-Dependent 1 | Protein Coding | 51 | GC10P050991 | 1.76 |
| 730 | CTSG | Cathepsin G | Protein Coding | 44 | GC14M024573 | 1.74 |
| 731 | CMTM7 | CKLF Like MARVEL Transmembrane Domain Containing 7 | Protein Coding | 37 | GC03P032409 | 1.74 |
| 732 | NDUFV1 | NADH:Ubiquinone Oxidoreductase Core Subunit V1 | Protein Coding | 45 | GC11P067632 | 1.73 |
| 733 | BNIP3 | BCL2 Interacting Protein 3 | Protein Coding | 42 | GC10M131966 | 1.73 |
| 734 | GAA | Glucosidase Alpha, Acid | Protein Coding | 47 | GC17P080101 | 1.71 |
| 735 | NTRK1 | Neurotrophic Receptor Tyrosine Kinase 1 | Protein Coding | 48 | GC01P156786 | 1.69 |
| 736 | MIR142 | MicroRNA 142 | RNA Gene | 20 | GC17M058331 | 1.69 |
| 737 | NEB | Nebulin | Protein Coding | 41 | GC02M151485 | 1.65 |
| 738 | NFATC1 | Nuclear Factor Of Activated T Cells 1 | Protein Coding | 47 | GC18P079395 | 1.65 |
| 739 | TBPL1 | TATA-Box Binding Protein Like 1 | Protein Coding | 40 | GC06P133897 | 1.65 |
| 740 | APOB | Apolipoprotein B | Protein Coding | 45 | GC02M020956 | 1.64 |
| 741 | LDHA | Lactate Dehydrogenase A | Protein Coding | 51 | GC11P018394 | 1.64 |
| 742 | TLN1 | Talin 1 | Protein Coding | 41 | GC09M035687 | 1.63 |
| 743 | CFL1 | Cofilin 1 | Protein Coding | 45 | GC11M065823 | 1.63 |
| 744 | CTNNA1 | Catenin Alpha 1 | Protein Coding | 47 | GC05P138613 | 1.63 |
| 745 | ITGB5 | Integrin Subunit Beta 5 | Protein Coding | 45 | GC03M124761 | 1.62 |
| 746 | RAC3 | Rac Family Small GTPase 3 | Protein Coding | 45 | GC17P082031 | 1.62 |
| 747 | CUL2 | Cullin 2 | Protein Coding | 42 | GC10M035046 | 1.61 |
| 748 | COL3A1 | Collagen Type III Alpha 1 Chain | Protein Coding | 47 | GC02P188974 | 1.6 |
| 749 | SLC6A4 | Solute Carrier Family 6 Member 4 | Protein Coding | 47 | GC17M030194 | 1.59 |
| 750 | KCNH2 | Potassium Voltage-Gated Channel Subfamily H Member 2 | Protein Coding | 49 | GC07M150944 | 1.59 |
| 751 | PYCARD | PYD And CARD Domain Containing | Protein Coding | 43 | GC16M031201 | 1.59 |
| 752 | ISL1 | ISL LIM Homeobox 1 | Protein Coding | 45 | GC05P051383 | 1.58 |
| 753 | LSM2 | LSM2 Homolog, U6 Small Nuclear RNA And MRNA Degradation Associated | Protein Coding | 40 | GC06M032632 | 1.58 |
| 754 | BDKRB2 | Bradykinin Receptor B2 | Protein Coding | 44 | GC14P096205 | 1.57 |
| 755 | TMX3 | Thioredoxin Related Transmembrane Protein 3 | Protein Coding | 38 | GC18M068673 | 1.57 |
| 756 | TRB | T Cell Receptor Beta Locus | Protein Coding | 14 | GC07P145138 | 1.57 |
| 757 | PIK3C3 | Phosphatidylinositol 3-Kinase Catalytic Subunit Type 3 | Protein Coding | 49 | GC18P041955 | 1.56 |
| 758 | SIX3 | SIX Homeobox 3 | Protein Coding | 42 | GC02P044941 | 1.55 |
| 759 | FDFT1 | Farnesyl-Diphosphate Farnesyltransferase 1 | Protein Coding | 44 | GC08P011795 | 1.55 |
| 760 | SNRNP70 | Small Nuclear Ribonucleoprotein U1 Subunit 70 | Protein Coding | 38 | GC19P049085 | 1.55 |
| 761 | HSPA9 | Heat Shock Protein Family A (Hsp70) Member 9 | Protein Coding | 47 | GC05M138554 | 1.54 |
| 762 | EPHA5 | EPH Receptor A5 | Protein Coding | 45 | GC04M065319 | 1.54 |
| 763 | PTPN22 | Protein Tyrosine Phosphatase Non-Receptor Type 22 | Protein Coding | 46 | GC01M113813 | 1.53 |
| 764 | GPX1 | Glutathione Peroxidase 1 | Protein Coding | 48 | GC03M049368 | 1.52 |
| 765 | NFAT5 | Nuclear Factor Of Activated T Cells 5 | Protein Coding | 42 | GC16P069565 | 1.52 |
| 766 | TSHR | Thyroid Stimulating Hormone Receptor | Protein Coding | 47 | GC14P080954 | 1.52 |
| 767 | CAV3 | Caveolin 3 | Protein Coding | 43 | GC03P008733 | 1.51 |
| 768 | BLZF1 | Basic Leucine Zipper Nuclear Factor 1 | Protein Coding | 39 | GC01P169367 | 1.51 |
| 769 | RPN2 | Ribophorin II | Protein Coding | 41 | GC20P037178 | 1.51 |
| 770 | LIFR | LIF Receptor Subunit Alpha | Protein Coding | 47 | GC05M038475 | 1.5 |
| 771 | MIR126 | MicroRNA 126 | RNA Gene | 22 | GC09P136670 | 1.5 |
| 772 | CRYZ | Crystallin Zeta | Protein Coding | 43 | GC01M074705 | 1.5 |
| 773 | CKB | Creatine Kinase B | Protein Coding | 45 | GC14M103519 | 1.49 |
| 774 | TPM4 | Tropomyosin 4 | Protein Coding | 40 | GC19P023264 | 1.49 |
| 775 | FCN1 | Ficolin 1 | Protein Coding | 39 | GC09M134975 | 1.49 |
| 776 | FBLN5 | Fibulin 5 | Protein Coding | 44 | GC14M091869 | 1.48 |
| 777 | HNRNPC | Heterogeneous Nuclear Ribonucleoprotein C | Protein Coding | 41 | GC14M021210 | 1.48 |
| 778 | TCFL5 | Transcription Factor Like 5 | Protein Coding | 36 | GC20M062841 | 1.48 |
| 779 | CCL23 | C-C Motif Chemokine Ligand 23 | Protein Coding | 34 | GC17M036013 | 1.47 |
| 780 | HNRNPDL | Heterogeneous Nuclear Ribonucleoprotein D Like | Protein Coding | 39 | GC04M082422 | 1.46 |
| 781 | PPP1R12A | Protein Phosphatase 1 Regulatory Subunit 12A | Protein Coding | 42 | GC12M079773 | 1.46 |
| 782 | CRLF2 | Cytokine Receptor Like Factor 2 | Protein Coding | 39 | GC0XM001190 | 1.46 |
| 783 | RUNX2 | RUNX Family Transcription Factor 2 | Protein Coding | 47 | GC06P047549 | 1.46 |
| 784 | SLC1A2 | Solute Carrier Family 1 Member 2 | Protein Coding | 48 | GC11M035272 | 1.46 |
| 785 | EEF1G | Eukaryotic Translation Elongation Factor 1 Gamma | Protein Coding | 40 | GC11M063390 | 1.45 |
| 786 | HSPG2 | Heparan Sulfate Proteoglycan 2 | Protein Coding | 45 | GC01M021822 | 1.45 |
| 787 | MYOD1 | Myogenic Differentiation 1 | Protein Coding | 46 | GC11P017741 | 1.45 |
| 788 | ABCD1 | ATP Binding Cassette Subfamily D Member 1 | Protein Coding | 45 | GC0XP153724 | 1.43 |
| 789 | BYSL | Bystin Like | Protein Coding | 38 | GC06P047504 | 1.43 |
| 790 | S1PR1 | Sphingosine-1-Phosphate Receptor 1 | Protein Coding | 44 | GC01P101236 | 1.41 |
| 791 | PKP2 | Plakophilin 2 | Protein Coding | 45 | GC12M032790 | 1.4 |
| 792 | TPI1 | Triosephosphate Isomerase 1 | Protein Coding | 48 | GC12P008208 | 1.4 |
| 793 | COTL1 | Coactosin Like F-Actin Binding Protein 1 | Protein Coding | 40 | GC16M084566 | 1.4 |
| 794 | MAP3K5 | Mitogen-Activated Protein Kinase Kinase Kinase 5 | Protein Coding | 47 | GC06M136557 | 1.4 |
| 795 | PRKAG2 | Protein Kinase AMP-Activated Non-Catalytic Subunit Gamma 2 | Protein Coding | 50 | GC07M151556 | 1.39 |
| 796 | APLN | Apelin | Protein Coding | 37 | GC0XM129645 | 1.39 |
| 797 | KIN | Kin17 DNA And RNA Binding Protein | Protein Coding | 37 | GC10M007750 | 1.39 |
| 798 | ADCY3 | Adenylate Cyclase 3 | Protein Coding | 47 | GC02M024819 | 1.38 |
| 799 | CRYAB | Crystallin Alpha B | Protein Coding | 45 | GC11M111908 | 1.38 |
| 800 | LAMA4 | Laminin Subunit Alpha 4 | Protein Coding | 44 | GC06M112107 | 1.38 |
| 801 | ITGA7 | Integrin Subunit Alpha 7 | Protein Coding | 47 | GC12M055684 | 1.38 |
| 802 | KCNJ5 | Potassium Inwardly Rectifying Channel Subfamily J Member 5 | Protein Coding | 47 | GC11P128891 | 1.38 |
| 803 | NFATC4 | Nuclear Factor Of Activated T Cells 4 | Protein Coding | 44 | GC14P024365 | 1.37 |
| 804 | KLRG1 | Killer Cell Lectin Like Receptor G1 | Protein Coding | 37 | GC12P008950 | 1.37 |
| 805 | NR3C2 | Nuclear Receptor Subfamily 3 Group C Member 2 | Protein Coding | 48 | GC04M148078 | 1.36 |
| 806 | VASP | Vasodilator Stimulated Phosphoprotein | Protein Coding | 43 | GC19P045507 | 1.34 |
| 807 | NF1 | Neurofibromin 1 | Protein Coding | 48 | GC17P031094 | 1.33 |
| 808 | SUOX | Sulfite Oxidase | Protein Coding | 45 | GC12P055997 | 1.33 |
| 809 | SLC18A1 | Solute Carrier Family 18 Member A1 | Protein Coding | 45 | GC08M020144 | 1.33 |
| 810 | CACNG5 | Calcium Voltage-Gated Channel Auxiliary Subunit Gamma 5 | Protein Coding | 40 | GC17P066835 | 1.33 |
| 811 | HRH2 | Histamine Receptor H2 | Protein Coding | 44 | GC05P175659 | 1.32 |
| 812 | PTPN3 | Protein Tyrosine Phosphatase Non-Receptor Type 3 | Protein Coding | 44 | GC09M109375 | 1.32 |
| 813 | PNMT | Phenylethanolamine N-Methyltransferase | Protein Coding | 44 | GC17P039667 | 1.31 |
| 814 | P2RX1 | Purinergic Receptor P2X 1 | Protein Coding | 43 | GC17M003896 | 1.31 |
| 815 | CSN1S1 | Casein Alpha S1 | Protein Coding | 34 | GC04P069932 | 1.3 |
| 816 | ADRA1D | Adrenoceptor Alpha 1D | Protein Coding | 45 | GC20M004220 | 1.29 |
| 817 | RAB1A | RAB1A, Member RAS Oncogene Family | Protein Coding | 41 | GC02M065048 | 1.29 |
| 818 | LNX1 | Ligand Of Numb-Protein X 1 | Protein Coding | 43 | GC04M053459 | 1.29 |
| 819 | PPL | Periplakin | Protein Coding | 40 | GC16M004872 | 1.28 |
| 820 | SUN2 | Sad1 And UNC84 Domain Containing 2 | Protein Coding | 37 | GC22M045390 | 1.28 |
| 821 | SLC25A3 | Solute Carrier Family 25 Member 3 | Protein Coding | 45 | GC12P098593 | 1.28 |
| 822 | MYOG | Myogenin | Protein Coding | 39 | GC01M203083 | 1.27 |
| 823 | SERPINH1 | Serpin Family H Member 1 | Protein Coding | 45 | GC11P075562 | 1.24 |
| 824 | VSIG4 | V-Set And Immunoglobulin Domain Containing 4 | Protein Coding | 36 | GC0XM066021 | 1.24 |
| 825 | TMEM43 | Transmembrane Protein 43 | Protein Coding | 40 | GC03P014124 | 1.23 |
| 826 | LRP6 | LDL Receptor Related Protein 6 | Protein Coding | 48 | GC12M013893 | 1.23 |
| 827 | HPR | Haptoglobin-Related Protein | Protein Coding | 40 | GC16P072097 | 1.23 |
| 828 | CYP2C19 | Cytochrome P450 Family 2 Subfamily C Member 19 | Protein Coding | 46 | GC10P094762 | 1.21 |
| 829 | COL1A1 | Collagen Type I Alpha 1 Chain | Protein Coding | 50 | GC17M050183 | 1.21 |
| 830 | LMO7 | LIM Domain 7 | Protein Coding | 40 | GC13P075620 | 1.21 |
| 831 | MEF2A | Myocyte Enhancer Factor 2A | Protein Coding | 47 | GC15P099565 | 1.21 |
| 832 | APOL1 | Apolipoprotein L1 | Protein Coding | 42 | GC22P036253 | 1.19 |
| 833 | CAMK2D | Calcium/Calmodulin Dependent Protein Kinase II Delta | Protein Coding | 48 | GC04M113452 | 1.19 |
| 834 | TPM3 | Tropomyosin 3 | Protein Coding | 47 | GC01M154127 | 1.19 |
| 835 | RAB1B | RAB1B, Member RAS Oncogene Family | Protein Coding | 41 | GC11P066270 | 1.19 |
| 836 | SNRPD3 | Small Nuclear Ribonucleoprotein D3 Polypeptide | Protein Coding | 36 | GC22P024555 | 1.18 |
| 837 | MEN1 | Menin 1 | Protein Coding | 46 | GC11M064803 | 1.17 |
| 838 | CFL2 | Cofilin 2 | Protein Coding | 44 | GC14M034706 | 1.17 |
| 839 | MAPK7 | Mitogen-Activated Protein Kinase 7 | Protein Coding | 48 | GC17P019379 | 1.17 |
| 840 | GDF15 | Growth Differentiation Factor 15 | Protein Coding | 41 | GC19P023329 | 1.17 |
| 841 | GNB3 | G Protein Subunit Beta 3 | Protein Coding | 47 | GC12P006839 | 1.16 |
| 842 | MYH14 | Myosin Heavy Chain 14 | Protein Coding | 45 | GC19P050192 | 1.16 |
| 843 | SNRPN | Small Nuclear Ribonucleoprotein Polypeptide N | Protein Coding | 44 | GC15P024823 | 1.16 |
| 844 | EPHX2 | Epoxide Hydrolase 2 | Protein Coding | 47 | GC08P027490 | 1.15 |
| 845 | THY1 | Thy-1 Cell Surface Antigen | Protein Coding | 44 | GC11M119417 | 1.15 |
| 846 | SEC22B | SEC22 Homolog B, Vesicle Trafficking Protein | Protein Coding | 35 | GC01M120150 | 1.15 |
| 847 | PKD1 | Polycystin 1, Transient Receptor Potential Channel Interacting | Protein Coding | 45 | GC16M002348 | 1.15 |
| 848 | ATP2A3 | ATPase Sarcoplasmic/Endoplasmic Reticulum Ca2+ Transporting 3 | Protein Coding | 44 | GC17M003923 | 1.15 |
| 849 | PER2 | Period Circadian Regulator 2 | Protein Coding | 43 | GC02M238244 | 1.15 |
| 850 | RTN4 | Reticulon 4 | Protein Coding | 44 | GC02M054934 | 1.15 |
| 851 | PIGK | Phosphatidylinositol Glycan Anchor Biosynthesis Class K | Protein Coding | 42 | GC01M077088 | 1.15 |
| 852 | IL3RA | Interleukin 3 Receptor Subunit Alpha | Protein Coding | 42 | GC0XP001336 | 1.15 |
| 853 | MYL2 | Myosin Light Chain 2 | Protein Coding | 49 | GC12M110910 | 1.14 |
| 854 | MYH11 | Myosin Heavy Chain 11 | Protein Coding | 45 | GC16M015704 | 1.14 |
| 855 | TPM2 | Tropomyosin 2 | Protein Coding | 44 | GC09M035672 | 1.13 |
| 856 | MTPN | Myotrophin | Protein Coding | 36 | GC07M135926 | 1.13 |
| 857 | AGER | Advanced Glycosylation End-Product Specific Receptor | Protein Coding | 44 | GC06M032180 | 1.13 |
| 858 | GATA6 | GATA Binding Protein 6 | Protein Coding | 47 | GC18P022169 | 1.12 |
| 859 | FBXO32 | F-Box Protein 32 | Protein Coding | 40 | GC08M123498 | 1.12 |
| 860 | ARPC4 | Actin Related Protein 2/3 Complex Subunit 4 | Protein Coding | 38 | GC03P009792 | 1.12 |
| 861 | PDLIM3 | PDZ And LIM Domain 3 | Protein Coding | 37 | GC04M185500 | 1.12 |
| 862 | BMPR2 | Bone Morphogenetic Protein Receptor Type 2 | Protein Coding | 50 | GC02P202376 | 1.12 |
| 863 | ADCY8 | Adenylate Cyclase 8 | Protein Coding | 44 | GC08M130780 | 1.12 |
| 864 | GLMN | Glomulin, FKBP Associated Protein | Protein Coding | 40 | GC01M092246 | 1.12 |
| 865 | ACVRL1 | Activin A Receptor Like Type 1 | Protein Coding | 50 | GC12P051906 | 1.11 |
| 866 | METAP2 | Methionyl Aminopeptidase 2 | Protein Coding | 44 | GC12P095473 | 1.11 |
| 867 | ZBTB17 | Zinc Finger And BTB Domain Containing 17 | Protein Coding | 41 | GC01M015943 | 1.11 |
| 868 | SIVA1 | SIVA1 Apoptosis Inducing Factor | Protein Coding | 36 | GC14P104853 | 1.11 |
| 869 | PLA2G7 | Phospholipase A2 Group VII | Protein Coding | 50 | GC06M046704 | 1.11 |
| 870 | PGM1 | Phosphoglucomutase 1 | Protein Coding | 48 | GC01P063593 | 1.11 |
| 871 | ITGA1 | Integrin Subunit Alpha 1 | Protein Coding | 41 | GC05P052788 | 1.11 |
| 872 | SYNE2 | Spectrin Repeat Containing Nuclear Envelope Protein 2 | Protein Coding | 39 | GC14P063761 | 1.11 |
| 873 | MAPKAPK3 | MAPK Activated Protein Kinase 3 | Protein Coding | 49 | GC03P050648 | 1.1 |
| 874 | RYR2 | Ryanodine Receptor 2 | Protein Coding | 47 | GC01P237042 | 1.1 |
| 875 | CACNA2D1 | Calcium Voltage-Gated Channel Auxiliary Subunit Alpha2delta 1 | Protein Coding | 45 | GC07M081946 | 1.1 |
| 876 | KLF5 | Kruppel Like Factor 5 | Protein Coding | 44 | GC13P073054 | 1.1 |
| 877 | EFEMP2 | EGF Containing Fibulin Extracellular Matrix Protein 2 | Protein Coding | 43 | GC11M065867 | 1.1 |
| 878 | KCND3 | Potassium Voltage-Gated Channel Subfamily D Member 3 | Protein Coding | 46 | GC01M111770 | 1.09 |
| 879 | FABP3 | Fatty Acid Binding Protein 3 | Protein Coding | 44 | GC01M031365 | 1.09 |
| 880 | MYLK3 | Myosin Light Chain Kinase 3 | Protein Coding | 41 | GC16M046714 | 1.09 |
| 881 | HBS1L | HBS1 Like Translational GTPase | Protein Coding | 39 | GC06M134960 | 1.09 |
| 882 | PPCS | Phosphopantothenoylcysteine Synthetase | Protein Coding | 39 | GC01P042456 | 1.09 |
| 883 | CLEC7A | C-Type Lectin Domain Containing 7A | Protein Coding | 44 | GC12M013863 | 1.09 |
| 884 | SOX9 | SRY-Box Transcription Factor 9 | Protein Coding | 47 | GC17P072121 | 1.08 |
| 885 | SAA1 | Serum Amyloid A1 | Protein Coding | 42 | GC11P018267 | 1.07 |
| 886 | ADCY1 | Adenylate Cyclase 1 | Protein Coding | 48 | GC07P045580 | 1.07 |
| 887 | TUBB1 | Tubulin Beta 1 Class VI | Protein Coding | 47 | GC20P059020 | 1.07 |
| 888 | CTNNA3 | Catenin Alpha 3 | Protein Coding | 39 | GC10M065912 | 1.07 |
| 889 | LNX2 | Ligand Of Numb-Protein X 2 | Protein Coding | 37 | GC13M027545 | 1.07 |
| 890 | ADCY6 | Adenylate Cyclase 6 | Protein Coding | 47 | GC12M048766 | 1.06 |
| 891 | LOX | Lysyl Oxidase | Protein Coding | 44 | GC05M122063 | 1.06 |
| 892 | AKAP9 | A-Kinase Anchoring Protein 9 | Protein Coding | 43 | GC07P091940 | 1.06 |
| 893 | PLEC | Plectin | Protein Coding | 42 | GC08M143916 | 1.06 |
| 894 | TRPV2 | Transient Receptor Potential Cation Channel Subfamily V Member 2 | Protein Coding | 41 | GC17P016415 | 1.06 |
| 895 | MYOM1 | Myomesin 1 | Protein Coding | 38 | GC18M003066 | 1.06 |
| 896 | TNNC2 | Troponin C2, Fast Skeletal Type | Protein Coding | 38 | GC20M045823 | 1.06 |
| 897 | TBX5 | T-Box Transcription Factor 5 | Protein Coding | 45 | GC12M114353 | 1.06 |
| 898 | RRAGC | Ras Related GTP Binding C | Protein Coding | 41 | GC01M038844 | 1.06 |
| 899 | CACNA2D3 | Calcium Voltage-Gated Channel Auxiliary Subunit Alpha2delta 3 | Protein Coding | 40 | GC03P054156 | 1.06 |
| 900 | LTBP4 | Latent Transforming Growth Factor Beta Binding Protein 4 | Protein Coding | 40 | GC19P040592 | 1.06 |
| 901 | NPPC | Natriuretic Peptide C | Protein Coding | 40 | GC02M231921 | 1.06 |
| 902 | KCNIP2 | Potassium Voltage-Gated Channel Interacting Protein 2 | Protein Coding | 40 | GC10M101825 | 1.06 |
| 903 | CHGB | Chromogranin B | Protein Coding | 39 | GC20P005911 | 1.06 |
| 904 | ADCY5 | Adenylate Cyclase 5 | Protein Coding | 48 | GC03M123282 | 1.06 |
| 905 | ILK | Integrin Linked Kinase | Protein Coding | 46 | GC11P006581 | 1.06 |
| 906 | ADCY7 | Adenylate Cyclase 7 | Protein Coding | 45 | GC16P050363 | 1.06 |
| 907 | ADCY9 | Adenylate Cyclase 9 | Protein Coding | 44 | GC16M003953 | 1.06 |
| 908 | ADCY2 | Adenylate Cyclase 2 | Protein Coding | 44 | GC05P007396 | 1.06 |
| 909 | PPIF | Peptidylprolyl Isomerase F | Protein Coding | 43 | GC10P083661 | 1.06 |
| 910 | MYOCD | Myocardin | Protein Coding | 41 | GC17P012665 | 1.06 |
| 911 | LIMS1 | LIM Zinc Finger Domain Containing 1 | Protein Coding | 40 | GC02P108609 | 1.06 |
| 912 | BAHD1 | Bromo Adjacent Homology Domain Containing 1 | Protein Coding | 35 | GC15P040439 | 1.06 |
| 913 | ATP2A1 | ATPase Sarcoplasmic/Endoplasmic Reticulum Ca2+ Transporting 1 | Protein Coding | 48 | GC16P029020 | 1.05 |
| 914 | TXNRD2 | Thioredoxin Reductase 2 | Protein Coding | 46 | GC22M019863 | 1.05 |
| 915 | TNNC1 | Troponin C1, Slow Skeletal And Cardiac Type | Protein Coding | 45 | GC03M052452 | 1.05 |
| 916 | ITPKC | Inositol-Trisphosphate 3-Kinase C | Protein Coding | 43 | GC19P040718 | 1.05 |
| 917 | OBSCN | Obscurin, Cytoskeletal Calmodulin And Titin-Interacting RhoGEF | Protein Coding | 40 | GC01P228208 | 1.05 |
| 918 | MATN1 | Matrilin 1 | Protein Coding | 39 | GC01M030711 | 1.05 |
| 919 | S100A12 | S100 Calcium Binding Protein A12 | Protein Coding | 39 | GC01M153373 | 1.05 |
| 920 | FGB | Fibrinogen Beta Chain | Protein Coding | 45 | GC04P154564 | 1.05 |
| 921 | C3AR1 | Complement C3a Receptor 1 | Protein Coding | 43 | GC12M008058 | 1.04 |
| 922 | COMP | Cartilage Oligomeric Matrix Protein | Protein Coding | 47 | GC19M018783 | 1.04 |
| 923 | SLC18A2 | Solute Carrier Family 18 Member A2 | Protein Coding | 47 | GC10P117241 | 1.04 |
| 924 | DTNA | Dystrobrevin Alpha | Protein Coding | 42 | GC18P034493 | 1.04 |
| 925 | ALMS1 | ALMS1 Centrosome And Basal Body Associated Protein | Protein Coding | 41 | GC02P073385 | 1.04 |
| 926 | GATA5 | GATA Binding Protein 5 | Protein Coding | 39 | GC20M062464 | 1.04 |
| 927 | ADRB3 | Adrenoceptor Beta 3 | Protein Coding | 45 | GC08M037962 | 1.03 |
| 928 | SLC8A1 | Solute Carrier Family 8 Member A1 | Protein Coding | 44 | GC02M040078 | 1.03 |
| 929 | ALG6 | ALG6 Alpha-1,3-Glucosyltransferase | Protein Coding | 41 | GC01P063367 | 1.03 |
| 930 | TRDN | Triadin | Protein Coding | 41 | GC06M123198 | 1.03 |
| 931 | FKRP | Fukutin Related Protein | Protein Coding | 40 | GC19P046746 | 1.03 |
| 932 | B4GALNT2 | Beta-1,4-N-Acetyl-Galactosaminyltransferase 2 | Protein Coding | 40 | GC17P049132 | 1.03 |
| 933 | SYNM | Synemin | Protein Coding | 37 | GC15P099098 | 1.03 |
| 934 | TMEM127 | Transmembrane Protein 127 | Protein Coding | 37 | GC02M096248 | 1.03 |
| 935 | MYOM3 | Myomesin 3 | Protein Coding | 33 | GC01M024056 | 1.03 |
| 936 | MIR10A | MicroRNA 10a | RNA Gene | 21 | GC17M048579 | 1.03 |
| 937 | MIR92B | MicroRNA 92b | RNA Gene | 16 | GC01P155195 | 1.03 |
| 938 | GOLGA2 | Golgin A2 | Protein Coding | 40 | GC09M128255 | 1.01 |
| 939 | PECAM1 | Platelet And Endothelial Cell Adhesion Molecule 1 | Protein Coding | 40 | GC17M064319 | 0.98 |
| 940 | KCNA4 | Potassium Voltage-Gated Channel Subfamily A Member 4 | Protein Coding | 43 | GC11M030009 | 0.98 |
| 941 | TBXT | T-Box Transcription Factor T | Protein Coding | 32 | GC06M166158 | 0.95 |
| 942 | HRH1 | Histamine Receptor H1 | Protein Coding | 45 | GC03P011113 | 0.93 |
| 943 | ST2 | Suppression Of Tumorigenicity 2 | Genetic Locus | 6 | GC11U990127 | 0.93 |
| 944 | RASA1 | RAS P21 Protein Activator 1 | Protein Coding | 46 | GC05P087267 | 0.91 |
| 945 | HDAC11 | Histone Deacetylase 11 | Protein Coding | 42 | GC03P013478 | 0.91 |
| 946 | ALOX5 | Arachidonate 5-Lipoxygenase | Protein Coding | 48 | GC10P045374 | 0.89 |
| 947 | SLC11A2 | Solute Carrier Family 11 Member 2 | Protein Coding | 47 | GC12M050952 | 0.87 |
| 948 | ACHE | Acetylcholinesterase (Cartwright Blood Group) | Protein Coding | 45 | GC07M100889 | 0.84 |
| 949 | LRP1 | LDL Receptor Related Protein 1 | Protein Coding | 47 | GC12P057128 | 0.83 |
| 950 | CHRNA5 | Cholinergic Receptor Nicotinic Alpha 5 Subunit | Protein Coding | 44 | GC15P078565 | 0.83 |
| 951 | ATP1A2 | ATPase Na+/K+ Transporting Subunit Alpha 2 | Protein Coding | 47 | GC01P160115 | 0.77 |
| 952 | SEMA4D | Semaphorin 4D | Protein Coding | 44 | GC09M089360 | 0.77 |
| 953 | MGP | Matrix Gla Protein | Protein Coding | 42 | GC12M014881 | 0.74 |
| 954 | DBT | Dihydrolipoamide Branched Chain Transacylase E2 | Protein Coding | 43 | GC01M100186 | 0.67 |
| 955 | CASP2 | Caspase 2 | Protein Coding | 49 | GC07P144979 | 0.62 |
| 956 | CCN1 | Cellular Communication Network Factor 1 | Protein Coding | 31 | GC01P085581 | 0.57 |
| 957 | LTB4R | Leukotriene B4 Receptor | Protein Coding | 44 | GC14P024311 | 0.56 |
| 958 | ADAM9 | ADAM Metallopeptidase Domain 9 | Protein Coding | 47 | GC08P038996 | 0.55 |
| 959 | TMEM39A | Transmembrane Protein 39A | Protein Coding | 35 | GC03M119428 | 0.55 |
| 960 | HYOU1 | Hypoxia Up-Regulated 1 | Protein Coding | 43 | GC11M119045 | 0.54 |
| 961 | SEMA4A | Semaphorin 4A | Protein Coding | 45 | GC01P156119 | 0.53 |
| 962 | FGF4 | Fibroblast Growth Factor 4 | Protein Coding | 44 | GC11M069762 | 0.53 |
| 963 | CXADRP1 | CXADR Pseudogene 1 | Pseudogene | 5 | GC21P013675 | 0.53 |
| 964 | SP7 | Sp7 Transcription Factor | Protein Coding | 41 | GC12M053326 | 0.49 |
| 965 | IGHV1-69 | Immunoglobulin Heavy Variable 1-69 | Protein Coding | 19 | GC14M109641 | 0.49 |
| 966 | P2RY2 | Purinergic Receptor P2Y2 | Protein Coding | 45 | GC11P073217 | 0.48 |
| 967 | DDAH2 | Dimethylarginine Dimethylaminohydrolase 2 | Protein Coding | 42 | GC06M031727 | 0.44 |
| 968 | MIRLET7I | MicroRNA Let-7i | RNA Gene | 20 | GC12P062606 | 0.43 |
| 969 | ABCC8 | ATP Binding Cassette Subfamily C Member 8 | Protein Coding | 45 | GC11M017392 | 0.41 |
| 970 | HSPB2 | Heat Shock Protein Family B (Small) Member 2 | Protein Coding | 40 | GC11P111913 | 0.41 |
| 971 | KCNB1 | Potassium Voltage-Gated Channel Subfamily B Member 1 | Protein Coding | 47 | GC20M049293 | 0.39 |
| 972 | CETP | Cholesteryl Ester Transfer Protein | Protein Coding | 46 | GC16P056961 | 0.39 |
| 973 | TNXB | Tenascin XB | Protein Coding | 43 | GC06M032635 | 0.39 |
| 974 | GCLC | Glutamate-Cysteine Ligase Catalytic Subunit | Protein Coding | 43 | GC06M053497 | 0.38 |
| 975 | BGN | Biglycan | Protein Coding | 43 | GC0XP153494 | 0.37 |
| 976 | TFPI | Tissue Factor Pathway Inhibitor | Protein Coding | 44 | GC02M187464 | 0.37 |
| 977 | MIR208B | MicroRNA 208b | RNA Gene | 19 | GC14M023417 | 0.37 |
| 978 | P2RY6 | Pyrimidinergic Receptor P2Y6 | Protein Coding | 42 | GC11P073264 | 0.36 |
| 979 | CMA1 | Chymase 1 | Protein Coding | 43 | GC14M024506 | 0.32 |
| 980 | KLF2 | Kruppel Like Factor 2 | Protein Coding | 40 | GC19P023268 | 0.28 |
| 981 | RHOU | Ras Homolog Family Member U | Protein Coding | 37 | GC01P228644 | 0.28 |
| 982 | P2RY1 | Purinergic Receptor P2Y1 | Protein Coding | 45 | GC03P152835 | 0.27 |
| 983 | SPRED1 | Sprouty Related EVH1 Domain Containing 1 | Protein Coding | 42 | GC15P038252 | 0.27 |
| 984 | FTMT | Ferritin Mitochondrial | Protein Coding | 38 | GC05P121851 | 0.21 |

| **Table S7. The common and unique VMC-related targets of different herbal strategies** | | |
| --- | --- | --- |
| **Herb strategy** | **Number of VMC-related targets** | **VMC-related target** |
| CH HQ JH JXT KS YGZ | 9 | SLC6A2 |
|  |  | NOS2 |
|  |  | SLC6A4 |
|  |  | PPARA |
|  |  | ACHE |
|  |  | CYP2C19 |
|  |  | PPARG |
|  |  | CYP51A1 |
|  |  | CHRM2 |
| CH HQ JXT KS YGZ | 23 | MMP2 |
|  |  | PTK2 |
|  |  | MTOR |
|  |  | PLG |
|  |  | TYR |
|  |  | ADORA2A |
|  |  | MMP3 |
|  |  | MET |
|  |  | DRD2 |
|  |  | MPO |
|  |  | ALOX5 |
|  |  | AKT1 |
|  |  | F2 |
|  |  | ARG1 |
|  |  | PIK3CG |
|  |  | EGFR |
|  |  | ABCC1 |
|  |  | SRC |
|  |  | ABCB1 |
|  |  | CCND1 |
|  |  | PIK3CA |
|  |  | MMP9 |
|  |  | PARP1 |
| CH HQ JH JXT YGZ | 2 | VDR |
|  |  | G6PD |
| HQ JXT KS YGZ | 3 | FLT1 |
|  |  | HSP90AA1 |
|  |  | RPS6KB1 |
| CH HQ JXT YGZ | 1 | FABP3 |
| CH HQ JXT KS | 7 | LCK |
|  |  | ABL1 |
|  |  | ADAM17 |
|  |  | ELANE |
|  |  | MMP1 |
|  |  | ODC1 |
|  |  | IL2 |
| CH HQ JH JXT | 1 | FDFT1 |
| HQ KS YGZ | 1 | TUBB1 |
| HQ JXT YGZ | 1 | PTPN11 |
| CH HQ KS | 3 | NTRK1 |
|  |  | CMA1 |
|  |  | KIT |
| HQ JXT KS | 9 | F3 |
|  |  | TLR9 |
|  |  | RAF1 |
|  |  | MMP8 |
|  |  | MAPK1 |
|  |  | PRKCA |
|  |  | RET |
|  |  | HDAC11 |
|  |  | MIF |
| CH HQ JXT | 2 | CASP7 |
|  |  | CASP3 |
| JXT KS YGZ | 3 | BCL2 |
|  |  | KCNH2 |
|  |  | MAPK3 |
| CH JXT YGZ | 3 | PSEN1 |
|  |  | MAPK8 |
|  |  | PSEN2 |
| CH JXT KS | 4 | ITK |
|  |  | TNF |
|  |  | CTSL |
|  |  | TSPO |
| HQ KS | 1 | EPHX2 |
| HQ JXT | 2 | PRF1 |
|  |  | PLAT |
| KS YGZ | 10 | SERPINE1 |
|  |  | VEGFA |
|  |  | ADRA1D |
|  |  | TNNI3 |
|  |  | PGD |
|  |  | PGF |
|  |  | TNNT2 |
|  |  | TNNC1 |
|  |  | STAT1 |
|  |  | ERBB2 |
| CH YGZ | 1 | P2RY1 |
| JXT YGZ | 3 | MAPK14 |
|  |  | IDO1 |
|  |  | ICAM1 |
| CH KS | 2 | HRH2 |
|  |  | ITGB3 |
| JXT KS | 10 | PNMT |
|  |  | NR3C2 |
|  |  | SLC18A2 |
|  |  | ADCY5 |
|  |  | HRH1 |
|  |  | PRKACA |
|  |  | ITGAL |
|  |  | SCN5A |
|  |  | IKBKB |
|  |  | S1PR1 |
| CH JXT | 1 | CPT2 |
| HQ | 9 | HSPA5 |
|  |  | SLC27A1 |
|  |  | HRAS |
|  |  | BDKRB2 |
|  |  | CYP1A1 |
|  |  | JUN |
|  |  | CTSG |
|  |  | HSF1 |
|  |  | TGFBR1 |
| YGZ | 7 | GSR |
|  |  | TNFRSF1A |
|  |  | STAT3 |
|  |  | HIF1A |
|  |  | SELL |
|  |  | SELE |
|  |  | HSPA1A |
| KS | 15 | AGTR1 |
|  |  | ADRB1 |
|  |  | ADRB2 |
|  |  | EDNRA |
|  |  | TACR1 |
|  |  | ITGA2B |
|  |  | RPS6KA5 |
|  |  | PCNA |
|  |  | ACVRL1 |
|  |  | ADRB3 |
|  |  | FKBP1A |
|  |  | RELA |
|  |  | CHRNA5 |
|  |  | ADCY1 |
|  |  | REN |
| CH | 5 | KCNJ5 |
|  |  | ITGAV |
|  |  | CASP1 |
|  |  | TDP2 |
|  |  | ADA |
| JXT | 12 | TLR4 |
|  |  | EIF2AK2 |
|  |  | ITGB2 |
|  |  | FYN |
|  |  | FASN |
|  |  | EPHA5 |
|  |  | PLA2G7 |
|  |  | BLK |
|  |  | NOS3 |
|  |  | IL6 |
|  |  | CXCR3 |
|  |  | ITGA4 |

| **Table S8. TOP 10 elements significantly enriched by whole target and VMC-related targets** | | | | | | | | | | |
| --- | --- | --- | --- | --- | --- | --- | --- | --- | --- | --- |
| **Herb** | **Target type** | **GO** | **Category** | **Description** | **LogP** | **Enrichment** | **Z-score** | **GeneID** | **Hits** | **Log(q-value)** |
| HQ | whole targets | GO:0016773 | GO Molecular Functions | phosphotransferase activity, alcohol group as acceptor | -68.79894781 | 9.945332013 | 28.7838215 | 25|132|207|238|558|595|673|816|891|896|983|1017|1019|1020|1021|1022|1024|1025|1111|1195|1196|1198|1263|1453|1457|1459|1612|1956|2048|2081|2260|2321|2322|2475|2870|2932|3098|3099|3480|3643|3718|3791|3815|3932|4233|4638|4751|4914|5156|5159|5163|5209|5290|5291|5292|5293|5294|5347|5578|5585|5586|5588|5594|5604|5747|5894|5979|6093|6195|6196|6198|6446|6714|6790|6850|7046|7150|7272|7465|8317|8445|8569|8851|9149|9212|9261|9475|9641|9891|10461|10769|10783|11040|23476|53944|57396|65220|120892|415116 | ABL1|ADK|AKT1|ALK|AXL|CCND1|BRAF|CAMK2B|CCNB1|CCND3|CDK1|CDK2|CDK4|CDK5|CDK6|CDK7|CDK8|CDK9|CHEK1|CLK1|CLK2|CLK3|PLK3|CSNK1D|CSNK2A1|CSNK2A2|DAPK1|EGFR|EPHB2|ERN1|FGFR1|FLT1|FLT3|MTOR|GRK6|GSK3B|HK1|HK2|IGF1R|INSR|JAK3|KDR|KIT|LCK|MET|MYLK|NEK2|NTRK1|PDGFRA|PDGFRB|PDK1|PFKFB3|PIK3CA|PIK3CB|PIM1|PIK3CD|PIK3CG|PLK1|PRKCA|PKN1|PKN2|PRKCQ|MAPK1|MAP2K1|PTK2|RAF1|RET|ROCK1|RPS6KA1|RPS6KA2|RPS6KB1|SGK1|SRC|AURKA|SYK|TGFBR1|TOP1|TTK|WEE1|CDC7|DYRK2|MKNK1|CDK5R1|DYRK1B|AURKB|MAPKAPK2|ROCK2|IKBKE|NUAK1|MERTK|PLK2|NEK6|PIM2|BRD4|CSNK1G1|CLK4|NADK|LRRK2|PIM3 | -64.30653667 |
|  |  | GO:0004672 | GO Molecular Functions | protein kinase activity | -67.50787891 | 10.93998709 | 29.34095183 | 25\|207\|238\|558\|595\|673\|816\|891\|896\|983\|1017\|1019\|1020\|1021\|1022\|1024\|1025\|1111\|1195\|1196\|1198\|1263\|1453\|1457\|1459\|1612\|1956\|2048\|2081\|2260\|2321\|2322\|2475\|2870\|2932\|3480\|3643\|3718\|3791\|3815\|3932\|4233\|4638\|4751\|4914\|5156\|5159\|5163\|5290\|5292\|5294\|5347\|5578\|5585\|5586\|5588\|5594\|5604\|5747\|5894\|5979\|6093\|6195\|6196\|6198\|6446\|6714\|6790\|6850\|7046\|7150\|7272\|7465\|8317\|8445\|8569\|8851\|9149\|9212\|9261\|9475\|9641\|9891\|10461\|10769\|10783\|11040\|23476\|53944\|57396\|120892\|415116 | ABL1\|AKT1\|ALK\|AXL\|CCND1\|BRAF\|CAMK2B\|CCNB1\|CCND3\|CDK1\|CDK2\|CDK4\|CDK5\|CDK6\|CDK7\|CDK8\|CDK9\|CHEK1\|CLK1\|CLK2\|CLK3\|PLK3\|CSNK1D\|CSNK2A1\|CSNK2A2\|DAPK1\|EGFR\|EPHB2\|ERN1\|FGFR1\|FLT1\|FLT3\|MTOR\|GRK6\|GSK3B\|IGF1R\|INSR\|JAK3\|KDR\|KIT\|LCK\|MET\|MYLK\|NEK2\|NTRK1\|PDGFRA\|PDGFRB\|PDK1\|PIK3CA\|PIM1\|PIK3CG\|PLK1\|PRKCA\|PKN1\|PKN2\|PRKCQ\|MAPK1\|MAP2K1\|PTK2\|RAF1\|RET\|ROCK1\|RPS6KA1\|RPS6KA2\|RPS6KB1\|SGK1\|SRC\|AURKA\|SYK\|TGFBR1\|TOP1\|TTK\|WEE1\|CDC7\|DYRK2\|MKNK1\|CDK5R1\|DYRK1B\|AURKB\|MAPKAPK2\|ROCK2\|IKBKE\|NUAK1\|MERTK\|PLK2\|NEK6\|PIM2\|BRD4\|CSNK1G1\|CLK4\|LRRK2\|PIM3 | -63.31649776 |
|  |  | GO:0016301 | GO Molecular Functions | kinase activity | -67.1185189 | 9.252185921 | 27.84049447 | 25\|132\|207\|238\|558\|595\|673\|816\|891\|896\|898\|983\|1017\|1019\|1020\|1021\|1022\|1024\|1025\|1111\|1195\|1196\|1198\|1263\|1453\|1457\|1459\|1612\|1956\|2048\|2081\|2260\|2321\|2322\|2475\|2870\|2932\|3098\|3099\|3480\|3643\|3718\|3791\|3815\|3932\|4233\|4638\|4751\|4914\|5156\|5159\|5163\|5209\|5230\|5290\|5291\|5292\|5293\|5294\|5347\|5578\|5585\|5586\|5588\|5594\|5604\|5747\|5894\|5979\|6093\|6195\|6196\|6198\|6446\|6714\|6790\|6850\|7046\|7150\|7272\|7465\|8317\|8445\|8569\|8851\|9149\|9212\|9261\|9475\|9641\|9891\|10461\|10769\|10783\|11040\|23476\|53944\|57396\|65220\|120892\|415116 | ABL1\|ADK\|AKT1\|ALK\|AXL\|CCND1\|BRAF\|CAMK2B\|CCNB1\|CCND3\|CCNE1\|CDK1\|CDK2\|CDK4\|CDK5\|CDK6\|CDK7\|CDK8\|CDK9\|CHEK1\|CLK1\|CLK2\|CLK3\|PLK3\|CSNK1D\|CSNK2A1\|CSNK2A2\|DAPK1\|EGFR\|EPHB2\|ERN1\|FGFR1\|FLT1\|FLT3\|MTOR\|GRK6\|GSK3B\|HK1\|HK2\|IGF1R\|INSR\|JAK3\|KDR\|KIT\|LCK\|MET\|MYLK\|NEK2\|NTRK1\|PDGFRA\|PDGFRB\|PDK1\|PFKFB3\|PGK1\|PIK3CA\|PIK3CB\|PIM1\|PIK3CD\|PIK3CG\|PLK1\|PRKCA\|PKN1\|PKN2\|PRKCQ\|MAPK1\|MAP2K1\|PTK2\|RAF1\|RET\|ROCK1\|RPS6KA1\|RPS6KA2\|RPS6KB1\|SGK1\|SRC\|AURKA\|SYK\|TGFBR1\|TOP1\|TTK\|WEE1\|CDC7\|DYRK2\|MKNK1\|CDK5R1\|DYRK1B\|AURKB\|MAPKAPK2\|ROCK2\|IKBKE\|NUAK1\|MERTK\|PLK2\|NEK6\|PIM2\|BRD4\|CSNK1G1\|CLK4\|NADK\|LRRK2\|PIM3 | -63.10322902 |
|  |  | GO:0071900 | GO Biological Processes | regulation of protein serine/threonine kinase activity | -48.29682951 | 9.414906752 | 23.83139583 | 25\|134\|136\|207\|238\|595\|673\|836\|890\|891\|892\|894\|896\|898\|902\|904\|983\|993\|995\|1019\|1022\|1139\|1815\|1845\|1956\|1991\|2048\|2081\|2260\|2321\|2322\|2911\|2914\|3064\|3156\|3265\|3326\|3356\|3480\|3643\|3815\|4282\|4914\|5159\|5291\|5294\|5319\|5347\|5585\|5594\|5604\|5770\|5777\|5781\|5894\|5979\|5999\|6714\|6850\|6868\|7046\|8654\|8851\|8900\|9133\|9134\|9261\|10926\|50507\|54106\|85417\|120892\|376497 | ABL1\|ADORA1\|ADORA2B\|AKT1\|ALK\|CCND1\|BRAF\|CASP3\|CCNA2\|CCNB1\|CCNC\|CCND2\|CCND3\|CCNE1\|CCNH\|CCNT1\|CDK1\|CDC25A\|CDC25C\|CDK4\|CDK7\|CHRNA7\|DRD4\|DUSP3\|EGFR\|ELANE\|EPHB2\|ERN1\|FGFR1\|FLT1\|FLT3\|GRM1\|GRM4\|HTT\|HMGCR\|HRAS\|HSP90AB1\|HTR2A\|IGF1R\|INSR\|KIT\|MIF\|NTRK1\|PDGFRB\|PIK3CB\|PIK3CG\|PLA2G1B\|PLK1\|PKN1\|MAPK1\|MAP2K1\|PTPN1\|PTPN6\|PTPN11\|RAF1\|RET\|RGS4\|SRC\|SYK\|ADAM17\|TGFBR1\|PDE5A\|CDK5R1\|CCNA1\|CCNB2\|CCNE2\|MAPKAPK2\|DBF4\|NOX4\|TLR9\|CCNB3\|LRRK2\|SLC27A1 | -44.40647836 |
|  |  | GO:0004674 | GO Molecular Functions | protein serine/threonine kinase activity | -47.42229693 | 10.51957419 | 24.39229765 | 207\|673\|816\|896\|983\|1017\|1019\|1020\|1021\|1022\|1024\|1025\|1111\|1195\|1196\|1198\|1263\|1453\|1457\|1459\|1612\|1956\|2081\|2475\|2870\|2932\|4638\|4751\|5163\|5290\|5292\|5294\|5347\|5578\|5585\|5586\|5588\|5594\|5604\|5894\|6093\|6195\|6196\|6198\|6446\|6790\|6850\|7046\|7150\|7272\|8317\|8445\|8569\|9149\|9212\|9261\|9475\|9641\|9891\|10769\|10783\|11040\|23476\|53944\|57396\|120892\|415116 | AKT1\|BRAF\|CAMK2B\|CCND3\|CDK1\|CDK2\|CDK4\|CDK5\|CDK6\|CDK7\|CDK8\|CDK9\|CHEK1\|CLK1\|CLK2\|CLK3\|PLK3\|CSNK1D\|CSNK2A1\|CSNK2A2\|DAPK1\|EGFR\|ERN1\|MTOR\|GRK6\|GSK3B\|MYLK\|NEK2\|PDK1\|PIK3CA\|PIM1\|PIK3CG\|PLK1\|PRKCA\|PKN1\|PKN2\|PRKCQ\|MAPK1\|MAP2K1\|RAF1\|ROCK1\|RPS6KA1\|RPS6KA2\|RPS6KB1\|SGK1\|AURKA\|SYK\|TGFBR1\|TOP1\|TTK\|CDC7\|DYRK2\|MKNK1\|DYRK1B\|AURKB\|MAPKAPK2\|ROCK2\|IKBKE\|NUAK1\|PLK2\|NEK6\|PIM2\|BRD4\|CSNK1G1\|CLK4\|LRRK2\|PIM3 | -43.62885579 |
|  |  | GO:1901699 | GO Biological Processes | cellular response to nitrogen compound | -45.46925946 | 7.932704764 | 22.02147139 | 25\|142\|196\|207\|231\|328\|351\|383\|760\|836\|840\|890\|896\|1017\|1019\|1020\|1080\|1129\|1139\|1394\|1584\|1956\|2101\|2348\|2350\|2358\|2475\|2554\|2561\|2562\|2566\|2741\|2932\|3066\|3164\|3297\|3309\|3480\|3643\|3718\|4313\|4314\|4363\|4914\|4988\|5138\|5139\|5140\|5144\|5156\|5290\|5294\|5295\|5319\|5468\|5588\|5594\|5743\|5747\|5770\|5771\|5781\|6093\|6198\|6532\|6714\|7184\|7430\|8851\|9475\|9734\|9971\|10014\|22933\|23621\|50507\|120892 | ABL1\|PARP1\|AHR\|AKT1\|AKR1B1\|APEX1\|APP\|ARG1\|CA2\|CASP3\|CASP7\|CCNA2\|CCND3\|CDK2\|CDK4\|CDK5\|CFTR\|CHRM2\|CHRNA7\|CRHR1\|CYP11B1\|EGFR\|ESRRA\|FOLR1\|FOLR2\|FPR2\|MTOR\|GABRA1\|GABRB2\|GABRB3\|GABRG2\|GLRA1\|GSK3B\|HDAC2\|NR4A1\|HSF1\|HSPA5\|IGF1R\|INSR\|JAK3\|MMP2\|MMP3\|ABCC1\|NTRK1\|OPRM1\|PDE2A\|PDE3A\|PDE3B\|PDE4D\|PDGFRA\|PIK3CA\|PIK3CG\|PIK3R1\|PLA2G1B\|PPARG\|PRKCQ\|MAPK1\|PTGS2\|PTK2\|PTPN1\|PTPN2\|PTPN11\|ROCK1\|RPS6KB1\|SLC6A4\|SRC\|HSP90B1\|EZR\|CDK5R1\|ROCK2\|HDAC9\|NR1H4\|HDAC5\|SIRT2\|BACE1\|NOX4\|LRRK2 | -41.75499957 |
|  |  | hsa05200 | KEGG Pathway | Pathways in cancer | -44.81368705 | 8.591508951 | 22.3690048 | 25\|207\|231\|238\|367\|572\|595\|624\|673\|816\|836\|840\|894\|896\|898\|1017\|1019\|1021\|1612\|1956\|2033\|2099\|2100\|2147\|2260\|2322\|2475\|2932\|3065\|3066\|3265\|3320\|3326\|3480\|3558\|3718\|3725\|3815\|4233\|4312\|4313\|4318\|4843\|4914\|5156\|5159\|5290\|5291\|5292\|5293\|5295\|5467\|5468\|5578\|5594\|5604\|5731\|5732\|5743\|5747\|5894\|5979\|6093\|6198\|6513\|7015\|7046\|7184\|8900\|9134\|9475\|11040 | ABL1\|AKT1\|AKR1B1\|ALK\|AR\|BAD\|CCND1\|BDKRB2\|BRAF\|CAMK2B\|CASP3\|CASP7\|CCND2\|CCND3\|CCNE1\|CDK2\|CDK4\|CDK6\|DAPK1\|EGFR\|EP300\|ESR1\|ESR2\|F2\|FGFR1\|FLT3\|MTOR\|GSK3B\|HDAC1\|HDAC2\|HRAS\|HSP90AA1\|HSP90AB1\|IGF1R\|IL2\|JAK3\|JUN\|KIT\|MET\|MMP1\|MMP2\|MMP9\|NOS2\|NTRK1\|PDGFRA\|PDGFRB\|PIK3CA\|PIK3CB\|PIM1\|PIK3CD\|PIK3R1\|PPARD\|PPARG\|PRKCA\|MAPK1\|MAP2K1\|PTGER1\|PTGER2\|PTGS2\|PTK2\|RAF1\|RET\|ROCK1\|RPS6KB1\|SLC2A1\|TERT\|TGFBR1\|HSP90B1\|CCNA1\|CCNE2\|ROCK2\|PIM2 | -41.16637395 |
|  |  | GO:0071417 | GO Biological Processes | cellular response to organonitrogen compound | -43.37699858 | 8.192566579 | 21.71446034 | 25\|142\|196\|207\|231\|328\|351\|383\|760\|836\|840\|890\|896\|1019\|1020\|1080\|1394\|1584\|1956\|2101\|2348\|2350\|2358\|2475\|2554\|2561\|2562\|2566\|2741\|2932\|3066\|3164\|3297\|3309\|3480\|3643\|3718\|4313\|4363\|4914\|4988\|5138\|5139\|5140\|5144\|5156\|5290\|5294\|5295\|5319\|5468\|5588\|5594\|5743\|5747\|5770\|5771\|5781\|6093\|6198\|6532\|6714\|7184\|7430\|9475\|9734\|9971\|10014\|22933\|23621\|50507\|120892 | ABL1\|PARP1\|AHR\|AKT1\|AKR1B1\|APEX1\|APP\|ARG1\|CA2\|CASP3\|CASP7\|CCNA2\|CCND3\|CDK4\|CDK5\|CFTR\|CRHR1\|CYP11B1\|EGFR\|ESRRA\|FOLR1\|FOLR2\|FPR2\|MTOR\|GABRA1\|GABRB2\|GABRB3\|GABRG2\|GLRA1\|GSK3B\|HDAC2\|NR4A1\|HSF1\|HSPA5\|IGF1R\|INSR\|JAK3\|MMP2\|ABCC1\|NTRK1\|OPRM1\|PDE2A\|PDE3A\|PDE3B\|PDE4D\|PDGFRA\|PIK3CA\|PIK3CG\|PIK3R1\|PLA2G1B\|PPARG\|PRKCQ\|MAPK1\|PTGS2\|PTK2\|PTPN1\|PTPN2\|PTPN11\|ROCK1\|RPS6KB1\|SLC6A4\|SRC\|HSP90B1\|EZR\|ROCK2\|HDAC9\|NR1H4\|HDAC5\|SIRT2\|BACE1\|NOX4\|LRRK2 | -39.78767743 |
|  |  | GO:0051347 | GO Biological Processes | positive regulation of transferase activity | -43.05881613 | 7.492196304 | 21.09314246 | 25\|134\|136\|207\|238\|558\|572\|595\|673\|891\|894\|896\|904\|983\|994\|1020\|1139\|1813\|1815\|1956\|1991\|2048\|2081\|2147\|2260\|2321\|2322\|2358\|2475\|2911\|2914\|3265\|3320\|3326\|3356\|3480\|3558\|3643\|3791\|3815\|4137\|4233\|4282\|4751\|4914\|5156\|5159\|5290\|5291\|5294\|5319\|5347\|5585\|5588\|5594\|5604\|5747\|5770\|5781\|5894\|5979\|6714\|6850\|6868\|7046\|8654\|8658\|8851\|9212\|9261\|10461\|10926\|50507\|54106\|120892\|376497 | ABL1\|ADORA1\|ADORA2B\|AKT1\|ALK\|AXL\|BAD\|CCND1\|BRAF\|CCNB1\|CCND2\|CCND3\|CCNT1\|CDK1\|CDC25B\|CDK5\|CHRNA7\|DRD2\|DRD4\|EGFR\|ELANE\|EPHB2\|ERN1\|F2\|FGFR1\|FLT1\|FLT3\|FPR2\|MTOR\|GRM1\|GRM4\|HRAS\|HSP90AA1\|HSP90AB1\|HTR2A\|IGF1R\|IL2\|INSR\|KDR\|KIT\|MAPT\|MET\|MIF\|NEK2\|NTRK1\|PDGFRA\|PDGFRB\|PIK3CA\|PIK3CB\|PIK3CG\|PLA2G1B\|PLK1\|PKN1\|PRKCQ\|MAPK1\|MAP2K1\|PTK2\|PTPN1\|PTPN11\|RAF1\|RET\|SRC\|SYK\|ADAM17\|TGFBR1\|PDE5A\|TNKS\|CDK5R1\|AURKB\|MAPKAPK2\|MERTK\|DBF4\|NOX4\|TLR9\|LRRK2\|SLC27A1 | -39.5206475 |
|  |  | GO:0018105 | GO Biological Processes | peptidyl-serine phosphorylation | -42.31686563 | 12.17322897 | 24.05778077 | 207\|351\|624\|673\|891\|983\|1017\|1020\|1195\|1453\|1457\|1459\|1956\|2081\|2475\|2932\|3320\|3326\|4282\|4985\|5144\|5290\|5347\|5578\|5585\|5586\|5588\|5594\|5743\|5894\|5979\|6093\|6198\|6446\|6714\|6790\|6850\|7046\|7150\|7272\|8317\|8445\|8569\|8658\|8851\|9149\|9212\|9261\|9475\|9641\|10013\|10769\|10783\|53944\|120892 | AKT1\|APP\|BDKRB2\|BRAF\|CCNB1\|CDK1\|CDK2\|CDK5\|CLK1\|CSNK1D\|CSNK2A1\|CSNK2A2\|EGFR\|ERN1\|MTOR\|GSK3B\|HSP90AA1\|HSP90AB1\|MIF\|OPRD1\|PDE4D\|PIK3CA\|PLK1\|PRKCA\|PKN1\|PKN2\|PRKCQ\|MAPK1\|PTGS2\|RAF1\|RET\|ROCK1\|RPS6KB1\|SGK1\|SRC\|AURKA\|SYK\|TGFBR1\|TOP1\|TTK\|CDC7\|DYRK2\|MKNK1\|TNKS\|CDK5R1\|DYRK1B\|AURKB\|MAPKAPK2\|ROCK2\|IKBKE\|HDAC6\|PLK2\|NEK6\|CSNK1G1\|LRRK2 | -38.82445449 |
|  | VMC-related targets | hsa05200 | KEGG Pathway | Pathways in cancer | -29.11734357 | 19.07934195 | 22.55140531 | 25\|207\|595\|624\|836\|840\|1956\|2147\|2475\|3265\|3320\|3558\|3725\|3815\|4233\|4312\|4313\|4318\|4843\|4914\|5290\|5468\|5578\|5594\|5747\|5894\|5979\|6198\|7046 | ABL1\|AKT1\|CCND1\|BDKRB2\|CASP3\|CASP7\|EGFR\|F2\|MTOR\|HRAS\|HSP90AA1\|IL2\|JUN\|KIT\|MET\|MMP1\|MMP2\|MMP9\|NOS2\|NTRK1\|PIK3CA\|PPARG\|PRKCA\|MAPK1\|PTK2\|RAF1\|RET\|RPS6KB1\|TGFBR1 | -24.66890007 |
|  |  | GO:0033674 | GO Biological Processes | positive regulation of kinase activity | -26.80872266 | 17.22329158 | 20.94356533 | 25\|207\|595\|1813\|1956\|1991\|2147\|2321\|2475\|3265\|3320\|3558\|3815\|4233\|4282\|4914\|5290\|5294\|5594\|5747\|5781\|5894\|5979\|6714\|6868\|7046\|54106\|376497 | ABL1\|AKT1\|CCND1\|DRD2\|EGFR\|ELANE\|F2\|FLT1\|MTOR\|HRAS\|HSP90AA1\|IL2\|KIT\|MET\|MIF\|NTRK1\|PIK3CA\|PIK3CG\|MAPK1\|PTK2\|PTPN11\|RAF1\|RET\|SRC\|ADAM17\|TGFBR1\|TLR9\|SLC27A1 | -22.66130916 |
|  |  | GO:0051347 | GO Biological Processes | positive regulation of transferase activity | -25.32927919 | 15.21885679 | 19.55630312 | 25\|207\|595\|1813\|1956\|1991\|2147\|2321\|2475\|3265\|3320\|3558\|3815\|4233\|4282\|4914\|5290\|5294\|5594\|5747\|5781\|5894\|5979\|6714\|6868\|7046\|54106\|376497 | ABL1\|AKT1\|CCND1\|DRD2\|EGFR\|ELANE\|F2\|FLT1\|MTOR\|HRAS\|HSP90AA1\|IL2\|KIT\|MET\|MIF\|NTRK1\|PIK3CA\|PIK3CG\|MAPK1\|PTK2\|PTPN11\|RAF1\|RET\|SRC\|ADAM17\|TGFBR1\|TLR9\|SLC27A1 | -21.35795694 |
|  |  | GO:0045860 | GO Biological Processes | positive regulation of protein kinase activity | -23.92274184 | 17.48139082 | 19.92876841 | 25\|207\|595\|1813\|1956\|1991\|2321\|2475\|3265\|3320\|3815\|4282\|4914\|5290\|5294\|5594\|5747\|5781\|5894\|5979\|6714\|6868\|7046\|54106\|376497 | ABL1\|AKT1\|CCND1\|DRD2\|EGFR\|ELANE\|FLT1\|MTOR\|HRAS\|HSP90AA1\|KIT\|MIF\|NTRK1\|PIK3CA\|PIK3CG\|MAPK1\|PTK2\|PTPN11\|RAF1\|RET\|SRC\|ADAM17\|TGFBR1\|TLR9\|SLC27A1 | -20.07635833 |
|  |  | GO:0009611 | GO Biological Processes | response to wounding | -23.74758356 | 14.42659185 | 18.62924925 | 135\|240\|383\|836\|1543\|1813\|1956\|2147\|2152\|2475\|3265\|3725\|3932\|4914\|5290\|5294\|5327\|5340\|5465\|5578\|5594\|5747\|5781\|5894\|6714\|6868\|7046 | ADORA2A\|ALOX5\|ARG1\|CASP3\|CYP1A1\|DRD2\|EGFR\|F2\|F3\|MTOR\|HRAS\|JUN\|LCK\|NTRK1\|PIK3CA\|PIK3CG\|PLAT\|PLG\|PPARA\|PRKCA\|MAPK1\|PTK2\|PTPN11\|RAF1\|SRC\|ADAM17\|TGFBR1 | -19.99811007 |
|  |  | GO:0007169 | GO Biological Processes | transmembrane receptor protein tyrosine kinase signaling pathway | -21.71997379 | 13.06205807 | 17.27389419 | 25\|207\|624\|836\|1956\|2152\|2321\|3265\|3320\|3815\|3932\|4233\|4313\|4318\|4914\|5290\|5327\|5578\|5594\|5747\|5781\|5894\|5979\|6198\|6714\|6868 | ABL1\|AKT1\|BDKRB2\|CASP3\|EGFR\|F3\|FLT1\|HRAS\|HSP90AA1\|KIT\|LCK\|MET\|MMP2\|MMP9\|NTRK1\|PIK3CA\|PLAT\|PRKCA\|MAPK1\|PTK2\|PTPN11\|RAF1\|RET\|RPS6KB1\|SRC\|ADAM17 | -18.04968154 |
|  |  | GO:1901699 | GO Biological Processes | cellular response to nitrogen compound | -20.3840592 | 13.63233504 | 16.98633562 | 25\|142\|207\|383\|836\|840\|1129\|1956\|2475\|3297\|3309\|4313\|4314\|4363\|4914\|5290\|5294\|5468\|5594\|5747\|5781\|6198\|6532\|6714 | ABL1\|PARP1\|AKT1\|ARG1\|CASP3\|CASP7\|CHRM2\|EGFR\|MTOR\|HSF1\|HSPA5\|MMP2\|MMP3\|ABCC1\|NTRK1\|PIK3CA\|PIK3CG\|PPARG\|MAPK1\|PTK2\|PTPN11\|RPS6KB1\|SLC6A4\|SRC | -16.90216014 |
|  |  | hsa01522 | KEGG Pathway | Endocrine resistance | -20.35060364 | 55.16835586 | 27.37017728 | 207\|595\|1956\|2475\|3265\|3725\|4313\|4318\|5290\|5594\|5747\|5894\|6198\|6714 | AKT1\|CCND1\|EGFR\|MTOR\|HRAS\|JUN\|MMP2\|MMP9\|PIK3CA\|MAPK1\|PTK2\|RAF1\|RPS6KB1\|SRC | -16.90216014 |
|  |  | hsa05205 | KEGG Pathway | Proteoglycans in cancer | -19.93013835 | 29.50024795 | 21.74827964 | 207\|595\|836\|1956\|2475\|3265\|4233\|4313\|4318\|5290\|5578\|5594\|5747\|5781\|5894\|6198\|6714 | AKT1\|CCND1\|CASP3\|EGFR\|MTOR\|HRAS\|MET\|MMP2\|MMP9\|PIK3CA\|PRKCA\|MAPK1\|PTK2\|PTPN11\|RAF1\|RPS6KB1\|SRC | -16.52308754 |
|  |  | GO:0071902 | GO Biological Processes | positive regulation of protein serine/threonine kinase activity | -19.35649612 | 20.65416278 | 18.99375771 | 207\|595\|1956\|1991\|2321\|3265\|3815\|4282\|4914\|5294\|5594\|5781\|5894\|5979\|6714\|6868\|7046\|54106\|376497 | AKT1\|CCND1\|EGFR\|ELANE\|FLT1\|HRAS\|KIT\|MIF\|NTRK1\|PIK3CG\|MAPK1\|PTPN11\|RAF1\|RET\|SRC\|ADAM17\|TGFBR1\|TLR9\|SLC27A1 | -15.98723387 |
| YGZ | whole targets | GO:0004672 | GO Molecular Functions | protein kinase activity | -54.35942806 | 11.04682576 | 26.42874142 | 207|238|558|595|673|816|891|983|1017|1019|1020|1021|1024|1111|1195|1196|1198|1326|1432|1457|1612|1859|1956|2050|2064|2268|2321|2322|2324|2475|2870|2932|3480|3643|3716|3717|3718|3791|4067|4233|4638|4751|5159|5170|5290|5292|5294|5347|5585|5591|5595|5599|5602|5604|5747|6098|6198|6714|6790|6850|7010|7150|7297|7786|8444|8445|8851|9149|9212|9891|10188|10733|10783|57396 | AKT1|ALK|AXL|CCND1|BRAF|CAMK2B|CCNB1|CDK1|CDK2|CDK4|CDK5|CDK6|CDK8|CHEK1|CLK1|CLK2|CLK3|MAP3K8|MAPK14|CSNK2A1|DAPK1|DYRK1A|EGFR|EPHB4|ERBB2|FGR|FLT1|FLT3|FLT4|MTOR|GRK6|GSK3B|IGF1R|INSR|JAK1|JAK2|JAK3|KDR|LYN|MET|MYLK|NEK2|PDGFRB|PDPK1|PIK3CA|PIM1|PIK3CG|PLK1|PKN1|PRKDC|MAPK3|MAPK8|MAPK10|MAP2K1|PTK2|ROS1|RPS6KB1|SRC|AURKA|SYK|TEK|TOP1|TYK2|MAP3K12|DYRK3|DYRK2|CDK5R1|DYRK1B|AURKB|NUAK1|TNK2|PLK4|NEK6|CLK4 | -49.91098456 |
|  |  | GO:0016773 | GO Molecular Functions | phosphotransferase activity, alcohol group as acceptor | -53.55867916 | 9.836837482 | 25.3400863 | 207\|238\|558\|595\|673\|816\|891\|983\|1017\|1019\|1020\|1021\|1024\|1111\|1195\|1196\|1198\|1326\|1432\|1457\|1612\|1859\|1956\|2050\|2064\|2268\|2321\|2322\|2324\|2475\|2645\|2870\|2932\|3480\|3643\|3716\|3717\|3718\|3791\|4067\|4233\|4638\|4751\|5159\|5170\|5209\|5290\|5291\|5292\|5293\|5294\|5347\|5585\|5591\|5595\|5599\|5602\|5604\|5747\|6098\|6198\|6714\|6790\|6850\|7010\|7150\|7297\|7786\|8444\|8445\|8851\|9149\|9212\|9891\|10188\|10733\|10783\|57396 | AKT1\|ALK\|AXL\|CCND1\|BRAF\|CAMK2B\|CCNB1\|CDK1\|CDK2\|CDK4\|CDK5\|CDK6\|CDK8\|CHEK1\|CLK1\|CLK2\|CLK3\|MAP3K8\|MAPK14\|CSNK2A1\|DAPK1\|DYRK1A\|EGFR\|EPHB4\|ERBB2\|FGR\|FLT1\|FLT3\|FLT4\|MTOR\|GCK\|GRK6\|GSK3B\|IGF1R\|INSR\|JAK1\|JAK2\|JAK3\|KDR\|LYN\|MET\|MYLK\|NEK2\|PDGFRB\|PDPK1\|PFKFB3\|PIK3CA\|PIK3CB\|PIM1\|PIK3CD\|PIK3CG\|PLK1\|PKN1\|PRKDC\|MAPK3\|MAPK8\|MAPK10\|MAP2K1\|PTK2\|ROS1\|RPS6KB1\|SRC\|AURKA\|SYK\|TEK\|TOP1\|TYK2\|MAP3K12\|DYRK3\|DYRK2\|CDK5R1\|DYRK1B\|AURKB\|NUAK1\|TNK2\|PLK4\|NEK6\|CLK4 | -49.41126566 |
|  |  | GO:0016301 | GO Molecular Functions | kinase activity | -51.61967987 | 9.085040567 | 24.30800835 | 207\|238\|558\|595\|673\|816\|891\|898\|983\|1017\|1019\|1020\|1021\|1024\|1111\|1195\|1196\|1198\|1326\|1432\|1457\|1612\|1859\|1956\|2050\|2064\|2268\|2321\|2322\|2324\|2475\|2645\|2870\|2932\|3480\|3643\|3716\|3717\|3718\|3791\|4067\|4233\|4638\|4751\|5159\|5170\|5209\|5290\|5291\|5292\|5293\|5294\|5347\|5585\|5591\|5595\|5599\|5602\|5604\|5747\|6098\|6198\|6714\|6790\|6850\|7010\|7150\|7297\|7786\|8444\|8445\|8851\|9149\|9212\|9891\|10188\|10733\|10783\|57396 | AKT1\|ALK\|AXL\|CCND1\|BRAF\|CAMK2B\|CCNB1\|CCNE1\|CDK1\|CDK2\|CDK4\|CDK5\|CDK6\|CDK8\|CHEK1\|CLK1\|CLK2\|CLK3\|MAP3K8\|MAPK14\|CSNK2A1\|DAPK1\|DYRK1A\|EGFR\|EPHB4\|ERBB2\|FGR\|FLT1\|FLT3\|FLT4\|MTOR\|GCK\|GRK6\|GSK3B\|IGF1R\|INSR\|JAK1\|JAK2\|JAK3\|KDR\|LYN\|MET\|MYLK\|NEK2\|PDGFRB\|PDPK1\|PFKFB3\|PIK3CA\|PIK3CB\|PIM1\|PIK3CD\|PIK3CG\|PLK1\|PKN1\|PRKDC\|MAPK3\|MAPK8\|MAPK10\|MAP2K1\|PTK2\|ROS1\|RPS6KB1\|SRC\|AURKA\|SYK\|TEK\|TOP1\|TYK2\|MAP3K12\|DYRK3\|DYRK2\|CDK5R1\|DYRK1B\|AURKB\|NUAK1\|TNK2\|PLK4\|NEK6\|CLK4 | -47.64835763 |
|  |  | GO:1901699 | GO Biological Processes | cellular response to nitrogen compound | -46.44590553 | 9.182601063 | 23.16296969 | 142\|196\|207\|231\|328\|351\|383\|760\|890\|1017\|1019\|1020\|1080\|1129\|1394\|1786\|1812\|1956\|2101\|2475\|2529\|2554\|2561\|2562\|2566\|2645\|2741\|2742\|2932\|3351\|3383\|3480\|3643\|3717\|3718\|4067\|4193\|4313\|4314\|4363\|4988\|5028\|5138\|5139\|5140\|5142\|5144\|5170\|5290\|5294\|5295\|5319\|5468\|5591\|5595\|5663\|5724\|5743\|5747\|5770\|5771\|5781\|6198\|6532\|6622\|6714\|6772\|6774\|8851\|23621\|50507 | PARP1\|AHR\|AKT1\|AKR1B1\|APEX1\|APP\|ARG1\|CA2\|CCNA2\|CDK2\|CDK4\|CDK5\|CFTR\|CHRM2\|CRHR1\|DNMT1\|DRD1\|EGFR\|ESRRA\|MTOR\|FUT7\|GABRA1\|GABRB2\|GABRB3\|GABRG2\|GCK\|GLRA1\|GLRA2\|GSK3B\|HTR1B\|ICAM1\|IGF1R\|INSR\|JAK2\|JAK3\|LYN\|MDM2\|MMP2\|MMP3\|ABCC1\|OPRM1\|P2RY1\|PDE2A\|PDE3A\|PDE3B\|PDE4B\|PDE4D\|PDPK1\|PIK3CA\|PIK3CG\|PIK3R1\|PLA2G1B\|PPARG\|PRKDC\|MAPK3\|PSEN1\|PTAFR\|PTGS2\|PTK2\|PTPN1\|PTPN2\|PTPN11\|RPS6KB1\|SLC6A4\|SNCA\|SRC\|STAT1\|STAT3\|CDK5R1\|BACE1\|NOX4 | -42.59952203 |
|  |  | GO:0071417 | GO Biological Processes | cellular response to organonitrogen compound | -44.88534721 | 9.570598291 | 23.05873737 | 142\|196\|207\|231\|328\|351\|383\|760\|890\|1019\|1020\|1080\|1394\|1786\|1812\|1956\|2101\|2475\|2529\|2554\|2561\|2562\|2566\|2645\|2741\|2742\|2932\|3351\|3383\|3480\|3643\|3717\|3718\|4067\|4193\|4313\|4363\|4988\|5028\|5138\|5139\|5140\|5142\|5144\|5170\|5290\|5294\|5295\|5319\|5468\|5591\|5595\|5663\|5724\|5743\|5747\|5770\|5771\|5781\|6198\|6532\|6622\|6714\|6772\|6774\|23621\|50507 | PARP1\|AHR\|AKT1\|AKR1B1\|APEX1\|APP\|ARG1\|CA2\|CCNA2\|CDK4\|CDK5\|CFTR\|CRHR1\|DNMT1\|DRD1\|EGFR\|ESRRA\|MTOR\|FUT7\|GABRA1\|GABRB2\|GABRB3\|GABRG2\|GCK\|GLRA1\|GLRA2\|GSK3B\|HTR1B\|ICAM1\|IGF1R\|INSR\|JAK2\|JAK3\|LYN\|MDM2\|MMP2\|ABCC1\|OPRM1\|P2RY1\|PDE2A\|PDE3A\|PDE3B\|PDE4B\|PDE4D\|PDPK1\|PIK3CA\|PIK3CG\|PIK3R1\|PLA2G1B\|PPARG\|PRKDC\|MAPK3\|PSEN1\|PTAFR\|PTGS2\|PTK2\|PTPN1\|PTPN2\|PTPN11\|RPS6KB1\|SLC6A4\|SNCA\|SRC\|STAT1\|STAT3\|BACE1\|NOX4 | -41.13587371 |
|  |  | GO:0051347 | GO Biological Processes | positive regulation of transferase activity | -42.97868803 | 8.53928382 | 21.82925775 | 134\|148\|150\|151\|152\|207\|238\|558\|595\|673\|891\|975\|983\|994\|1020\|1326\|1432\|1813\|1815\|1956\|2050\|2064\|2147\|2246\|2268\|2321\|2322\|2324\|2475\|2911\|2914\|3320\|3326\|3356\|3357\|3480\|3643\|3717\|3791\|4067\|4137\|4233\|4751\|5159\|5170\|5290\|5291\|5294\|5319\|5347\|5585\|5595\|5602\|5604\|5663\|5747\|5770\|5781\|6098\|6622\|6714\|6850\|7010\|7422\|7786\|8658\|8851\|9212\|50507 | ADORA1\|ADRA1A\|ADRA2A\|ADRA2B\|ADRA2C\|AKT1\|ALK\|AXL\|CCND1\|BRAF\|CCNB1\|CD81\|CDK1\|CDC25B\|CDK5\|MAP3K8\|MAPK14\|DRD2\|DRD4\|EGFR\|EPHB4\|ERBB2\|F2\|FGF1\|FGR\|FLT1\|FLT3\|FLT4\|MTOR\|GRM1\|GRM4\|HSP90AA1\|HSP90AB1\|HTR2A\|HTR2B\|IGF1R\|INSR\|JAK2\|KDR\|LYN\|MAPT\|MET\|NEK2\|PDGFRB\|PDPK1\|PIK3CA\|PIK3CB\|PIK3CG\|PLA2G1B\|PLK1\|PKN1\|MAPK3\|MAPK10\|MAP2K1\|PSEN1\|PTK2\|PTPN1\|PTPN11\|ROS1\|SNCA\|SRC\|SYK\|TEK\|VEGFA\|MAP3K12\|TNKS\|CDK5R1\|AURKB\|NOX4 | -39.37534257 |
|  |  | GO:0033674 | GO Biological Processes | positive regulation of kinase activity | -42.12144501 | 9.103739837 | 22.02337774 | 134\|148\|150\|151\|152\|207\|238\|558\|595\|673\|891\|975\|983\|994\|1020\|1326\|1432\|1813\|1815\|1956\|2050\|2064\|2147\|2246\|2268\|2321\|2322\|2324\|2475\|2911\|2914\|3320\|3326\|3356\|3357\|3480\|3643\|3717\|3791\|4067\|4137\|4233\|5159\|5170\|5290\|5291\|5294\|5319\|5585\|5595\|5602\|5604\|5663\|5747\|5770\|5781\|6098\|6622\|6714\|6850\|7010\|7422\|7786\|8851\|50507 | ADORA1\|ADRA1A\|ADRA2A\|ADRA2B\|ADRA2C\|AKT1\|ALK\|AXL\|CCND1\|BRAF\|CCNB1\|CD81\|CDK1\|CDC25B\|CDK5\|MAP3K8\|MAPK14\|DRD2\|DRD4\|EGFR\|EPHB4\|ERBB2\|F2\|FGF1\|FGR\|FLT1\|FLT3\|FLT4\|MTOR\|GRM1\|GRM4\|HSP90AA1\|HSP90AB1\|HTR2A\|HTR2B\|IGF1R\|INSR\|JAK2\|KDR\|LYN\|MAPT\|MET\|PDGFRB\|PDPK1\|PIK3CA\|PIK3CB\|PIK3CG\|PLA2G1B\|PKN1\|MAPK3\|MAPK10\|MAP2K1\|PSEN1\|PTK2\|PTPN1\|PTPN11\|ROS1\|SNCA\|SRC\|SYK\|TEK\|VEGFA\|MAP3K12\|CDK5R1\|NOX4 | -38.5760915 |
|  |  | hsa05200 | KEGG Pathway | Pathways in cancer | -39.53307826 | 9.137840803 | 21.36913669 | 207\|231\|238\|367\|595\|596\|673\|816\|898\|1017\|1019\|1021\|1612\|1956\|2064\|2099\|2100\|2147\|2246\|2322\|2324\|2475\|2932\|3091\|3320\|3326\|3480\|3716\|3717\|3718\|4193\|4233\|4313\|4318\|4843\|5159\|5228\|5290\|5291\|5292\|5293\|5295\|5467\|5468\|5595\|5599\|5602\|5604\|5731\|5732\|5733\|5743\|5747\|6198\|6608\|6772\|6774\|7015\|7422\|8900\|9134 | AKT1\|AKR1B1\|ALK\|AR\|CCND1\|BCL2\|BRAF\|CAMK2B\|CCNE1\|CDK2\|CDK4\|CDK6\|DAPK1\|EGFR\|ERBB2\|ESR1\|ESR2\|F2\|FGF1\|FLT3\|FLT4\|MTOR\|GSK3B\|HIF1A\|HSP90AA1\|HSP90AB1\|IGF1R\|JAK1\|JAK2\|JAK3\|MDM2\|MET\|MMP2\|MMP9\|NOS2\|PDGFRB\|PGF\|PIK3CA\|PIK3CB\|PIM1\|PIK3CD\|PIK3R1\|PPARD\|PPARG\|MAPK3\|MAPK8\|MAPK10\|MAP2K1\|PTGER1\|PTGER2\|PTGER3\|PTGS2\|PTK2\|RPS6KB1\|SMO\|STAT1\|STAT3\|TERT\|VEGFA\|CCNA1\|CCNE2 | -36.0604895 |
|  |  | GO:0018108 | GO Biological Processes | peptidyl-tyrosine phosphorylation | -39.508933 | 11.94410667 | 23.12393005 | 134\|148\|150\|238\|351\|558\|975\|1195\|1196\|1198\|1859\|1956\|2050\|2064\|2268\|2321\|2322\|2324\|2475\|3356\|3383\|3480\|3643\|3716\|3717\|3718\|3791\|4067\|4233\|5159\|5595\|5604\|5663\|5747\|5770\|5771\|5777\|5781\|6098\|6714\|6774\|6850\|7010\|7132\|7297\|7422\|8444\|8445\|9149\|10188\|50507\|57396 | ADORA1\|ADRA1A\|ADRA2A\|ALK\|APP\|AXL\|CD81\|CLK1\|CLK2\|CLK3\|DYRK1A\|EGFR\|EPHB4\|ERBB2\|FGR\|FLT1\|FLT3\|FLT4\|MTOR\|HTR2A\|ICAM1\|IGF1R\|INSR\|JAK1\|JAK2\|JAK3\|KDR\|LYN\|MET\|PDGFRB\|MAPK3\|MAP2K1\|PSEN1\|PTK2\|PTPN1\|PTPN2\|PTPN6\|PTPN11\|ROS1\|SRC\|STAT3\|SYK\|TEK\|TNFRSF1A\|TYK2\|VEGFA\|DYRK3\|DYRK2\|DYRK1B\|TNK2\|NOX4\|CLK4 | -36.0604895 |
|  |  | GO:0018212 | GO Biological Processes | peptidyl-tyrosine modification | -39.32823723 | 11.84931217 | 23.01639961 | 134\|148\|150\|238\|351\|558\|975\|1195\|1196\|1198\|1859\|1956\|2050\|2064\|2268\|2321\|2322\|2324\|2475\|3356\|3383\|3480\|3643\|3716\|3717\|3718\|3791\|4067\|4233\|5159\|5595\|5604\|5663\|5747\|5770\|5771\|5777\|5781\|6098\|6714\|6774\|6850\|7010\|7132\|7297\|7422\|8444\|8445\|9149\|10188\|50507\|57396 | ADORA1\|ADRA1A\|ADRA2A\|ALK\|APP\|AXL\|CD81\|CLK1\|CLK2\|CLK3\|DYRK1A\|EGFR\|EPHB4\|ERBB2\|FGR\|FLT1\|FLT3\|FLT4\|MTOR\|HTR2A\|ICAM1\|IGF1R\|INSR\|JAK1\|JAK2\|JAK3\|KDR\|LYN\|MET\|PDGFRB\|MAPK3\|MAP2K1\|PSEN1\|PTK2\|PTPN1\|PTPN2\|PTPN6\|PTPN11\|ROS1\|SRC\|STAT3\|SYK\|TEK\|TNFRSF1A\|TYK2\|VEGFA\|DYRK3\|DYRK2\|DYRK1B\|TNK2\|NOX4\|CLK4 | -35.92118642 |
|  | VMC-related targets | hsa01522 | KEGG Pathway | Endocrine resistance | -22.99379861 | 65.28451493 | 30.90334097 | 207\|595\|596\|1432\|1956\|2064\|2475\|4313\|4318\|5290\|5595\|5599\|5747\|6198\|6714 | AKT1\|CCND1\|BCL2\|MAPK14\|EGFR\|ERBB2\|MTOR\|MMP2\|MMP9\|PIK3CA\|MAPK3\|MAPK8\|PTK2\|RPS6KB1\|SRC | -19.02247636 |
|  |  | hsa05205 | KEGG Pathway | Proteoglycans in cancer | -22.43904619 | 34.49897302 | 24.32063271 | 207\|595\|1432\|1956\|2064\|2475\|3091\|4233\|4313\|4318\|5290\|5595\|5747\|5781\|6198\|6714\|6774\|7422 | AKT1\|CCND1\|MAPK14\|EGFR\|ERBB2\|MTOR\|HIF1A\|MET\|MMP2\|MMP9\|PIK3CA\|MAPK3\|PTK2\|PTPN11\|RPS6KB1\|SRC\|STAT3\|VEGFA | -18.59266268 |
|  |  | hsa05200 | KEGG Pathway | Pathways in cancer | -21.63984277 | 16.71283582 | 18.64715884 | 207\|595\|596\|1956\|2064\|2147\|2475\|3091\|3320\|4233\|4313\|4318\|4843\|5228\|5290\|5468\|5595\|5599\|5747\|6198\|6772\|6774\|7422 | AKT1\|CCND1\|BCL2\|EGFR\|ERBB2\|F2\|MTOR\|HIF1A\|HSP90AA1\|MET\|MMP2\|MMP9\|NOS2\|PGF\|PIK3CA\|PPARG\|MAPK3\|MAPK8\|PTK2\|RPS6KB1\|STAT1\|STAT3\|VEGFA | -17.89036927 |
|  |  | GO:0008015 | GO Biological Processes | blood circulation | -20.6855265 | 16.77383157 | 18.26576769 | 135\|146\|207\|1129\|1813\|1956\|2475\|3383\|3757\|4843\|5028\|5290\|5294\|5465\|5468\|6532\|6714\|6772\|7134\|7137\|7139\|7422 | ADORA2A\|ADRA1D\|AKT1\|CHRM2\|DRD2\|EGFR\|MTOR\|ICAM1\|KCNH2\|NOS2\|P2RY1\|PIK3CA\|PIK3CG\|PPARA\|PPARG\|SLC6A4\|SRC\|STAT1\|TNNC1\|TNNI3\|TNNT2\|VEGFA | -17.08218104 |
|  |  | GO:0003013 | GO Biological Processes | circulatory system process | -20.49942137 | 16.44375617 | 18.06576427 | 135\|146\|207\|1129\|1813\|1956\|2475\|3383\|3757\|4843\|5028\|5290\|5294\|5465\|5468\|6532\|6714\|6772\|7134\|7137\|7139\|7422 | ADORA2A\|ADRA1D\|AKT1\|CHRM2\|DRD2\|EGFR\|MTOR\|ICAM1\|KCNH2\|NOS2\|P2RY1\|PIK3CA\|PIK3CG\|PPARA\|PPARG\|SLC6A4\|SRC\|STAT1\|TNNC1\|TNNI3\|TNNT2\|VEGFA | -16.95406786 |
|  |  | hsa04933 | KEGG Pathway | AGE-RAGE signaling pathway in diabetic complications | -20.31300723 | 54.66815455 | 27.24316955 | 207\|595\|596\|1432\|3383\|4313\|5054\|5290\|5595\|5599\|6401\|6772\|6774\|7422 | AKT1\|CCND1\|BCL2\|MAPK14\|ICAM1\|MMP2\|SERPINE1\|PIK3CA\|MAPK3\|MAPK8\|SELE\|STAT1\|STAT3\|VEGFA | -16.81880624 |
|  |  | GO:1901699 | GO Biological Processes | cellular response to nitrogen compound | -20.20630214 | 14.42925015 | 17.18046581 | 142\|207\|383\|1129\|1956\|2475\|3383\|4313\|4314\|4363\|5028\|5290\|5294\|5468\|5595\|5663\|5747\|5781\|6198\|6532\|6714\|6772\|6774 | PARP1\|AKT1\|ARG1\|CHRM2\|EGFR\|MTOR\|ICAM1\|MMP2\|MMP3\|ABCC1\|P2RY1\|PIK3CA\|PIK3CG\|PPARG\|MAPK3\|PSEN1\|PTK2\|PTPN11\|RPS6KB1\|SLC6A4\|SRC\|STAT1\|STAT3 | -16.75785864 |
|  |  | GO:0031667 | GO Biological Processes | response to nutrient levels | -19.84322596 | 17.0373569 | 17.99226868 | 207\|383\|595\|596\|1956\|2475\|2539\|3383\|4353\|5028\|5465\|5468\|5595\|5599\|6198\|6402\|6532\|6714\|6772\|7299\|7421 | AKT1\|ARG1\|CCND1\|BCL2\|EGFR\|MTOR\|G6PD\|ICAM1\|MPO\|P2RY1\|PPARA\|PPARG\|MAPK3\|MAPK8\|RPS6KB1\|SELL\|SLC6A4\|SRC\|STAT1\|TYR\|VDR | -16.43617514 |
|  |  | hsa04066 | KEGG Pathway | HIF-1 signaling pathway | -19.63545145 | 49.15539947 | 25.78463651 | 207\|596\|1956\|2064\|2321\|2475\|3091\|4843\|5054\|5290\|5595\|6198\|6774\|7422 | AKT1\|BCL2\|EGFR\|ERBB2\|FLT1\|MTOR\|HIF1A\|NOS2\|SERPINE1\|PIK3CA\|MAPK3\|RPS6KB1\|STAT3\|VEGFA | -16.27403395 |
|  |  | GO:0034599 | GO Biological Processes | cellular response to oxidative stress | -19.60853409 | 24.10505166 | 20.10182509 | 142\|207\|240\|383\|596\|1956\|2539\|2936\|3091\|3303\|4233\|4313\|4314\|4318\|4353\|5595\|5599\|6714 | PARP1\|AKT1\|ALOX5\|ARG1\|BCL2\|EGFR\|G6PD\|GSR\|HIF1A\|HSPA1A\|MET\|MMP2\|MMP3\|MMP9\|MPO\|MAPK3\|MAPK8\|SRC | -16.27403395 |
| KS | whole targets | hsa04080 | KEGG Pathway | Neuroactive ligand-receptor interaction | -79.88306055 | 14.08314691 | 34.33687266 | 134|135|136|140|146|147|148|150|151|152|153|154|155|185|554|706|887|1128|1129|1131|1132|1133|1134|1135|1136|1137|1138|1139|1140|1141|1142|1143|1144|1146|1268|1269|1394|1812|1813|1814|1815|1816|1901|1903|1909|2147|2554|2556|2558|2559|2562|2566|2642|2837|2859|2902|2904|2908|2911|2912|2913|2914|2915|2916|2918|3269|3274|3350|3351|3352|3355|3356|3357|3358|3361|3362|3363|4985|4986|4987|4988|5024|5340|5724|5731|5732|5733|5734|6869|7442|8973|11255|59340|84634 | ADORA1|ADORA2A|ADORA2B|ADORA3|ADRA1D|ADRA1B|ADRA1A|ADRA2A|ADRA2B|ADRA2C|ADRB1|ADRB2|ADRB3|AGTR1|AVPR2|TSPO|CCKBR|CHRM1|CHRM2|CHRM3|CHRM4|CHRM5|CHRNA1|CHRNA2|CHRNA3|CHRNA4|CHRNA5|CHRNA7|CHRNB1|CHRNB2|CHRNB3|CHRNB4|CHRND|CHRNG|CNR1|CNR2|CRHR1|DRD1|DRD2|DRD3|DRD4|DRD5|S1PR1|S1PR3|EDNRA|F2|GABRA1|GABRA3|GABRA5|GABRA6|GABRB3|GABRG2|GCGR|UTS2R|GPR35|GRIN1|GRIN2B|NR3C1|GRM1|GRM2|GRM3|GRM4|GRM5|GRM6|GRM8|HRH1|HRH2|HTR1A|HTR1B|HTR1D|HTR1F|HTR2A|HTR2B|HTR2C|HTR5A|HTR6|HTR7|OPRD1|OPRK1|OPRL1|OPRM1|P2RX3|PLG|PTAFR|PTGER1|PTGER2|PTGER3|PTGER4|TACR1|TRPV1|CHRNA6|HRH3|HRH4|KISS1R | -75.73564705 |
|  |  | GO:0004672 | GO Molecular Functions | protein kinase activity | -71.17553302 | 9.895437308 | 29.2641682 | 25\|94\|156\|207\|238\|558\|595\|673\|816\|891\|896\|983\|1017\|1018\|1019\|1020\|1021\|1111\|1195\|1326\|1457\|1612\|1859\|1956\|2050\|2064\|2081\|2260\|2321\|2322\|2324\|2475\|2870\|2932\|3055\|3480\|3551\|3643\|3702\|3716\|3718\|3791\|3815\|3932\|4233\|4638\|4750\|4751\|4914\|5156\|5159\|5163\|5170\|5290\|5292\|5294\|5347\|5566\|5578\|5585\|5591\|5594\|5595\|5602\|5747\|5753\|5894\|5979\|6093\|6098\|6195\|6197\|6198\|6446\|6714\|6790\|6850\|6885\|7150\|7294\|7465\|7786\|8317\|8569\|8851\|9020\|9149\|9212\|9252\|9261\|9475\|9891\|10298\|10461\|10783\|11040\|11200\|23476\|29110\|29904\|51086\|53944\|415116 | ABL1\|ACVRL1\|GRK2\|AKT1\|ALK\|AXL\|CCND1\|BRAF\|CAMK2B\|CCNB1\|CCND3\|CDK1\|CDK2\|CDK3\|CDK4\|CDK5\|CDK6\|CHEK1\|CLK1\|MAP3K8\|CSNK2A1\|DAPK1\|DYRK1A\|EGFR\|EPHB4\|ERBB2\|ERN1\|FGFR1\|FLT1\|FLT3\|FLT4\|MTOR\|GRK6\|GSK3B\|HCK\|IGF1R\|IKBKB\|INSR\|ITK\|JAK1\|JAK3\|KDR\|KIT\|LCK\|MET\|MYLK\|NEK1\|NEK2\|NTRK1\|PDGFRA\|PDGFRB\|PDK1\|PDPK1\|PIK3CA\|PIM1\|PIK3CG\|PLK1\|PRKACA\|PRKCA\|PKN1\|PRKDC\|MAPK1\|MAPK3\|MAPK10\|PTK2\|PTK6\|RAF1\|RET\|ROCK1\|ROS1\|RPS6KA1\|RPS6KA3\|RPS6KB1\|SGK1\|SRC\|AURKA\|SYK\|MAP3K7\|TOP1\|TXK\|WEE1\|MAP3K12\|CDC7\|MKNK1\|CDK5R1\|MAP3K14\|DYRK1B\|AURKB\|RPS6KA5\|MAPKAPK2\|ROCK2\|NUAK1\|PAK4\|MERTK\|NEK6\|PIM2\|CHEK2\|BRD4\|TBK1\|EEF2K\|TNNI3K\|CSNK1G1\|PIM3 | -67.20421078 |
|  |  | GO:0016773 | GO Molecular Functions | phosphotransferase activity, alcohol group as acceptor | -70.18336328 | 8.846660771 | 28.1395643 | 25\|94\|156\|207\|238\|558\|595\|673\|816\|891\|896\|983\|1017\|1018\|1019\|1020\|1021\|1111\|1195\|1326\|1457\|1612\|1859\|1956\|2050\|2064\|2081\|2260\|2321\|2322\|2324\|2475\|2870\|2932\|3055\|3480\|3551\|3643\|3702\|3716\|3718\|3791\|3815\|3932\|4233\|4638\|4750\|4751\|4914\|5156\|5159\|5163\|5170\|5209\|5290\|5291\|5292\|5293\|5294\|5298\|5347\|5566\|5578\|5585\|5591\|5594\|5595\|5602\|5747\|5753\|5894\|5979\|6093\|6098\|6195\|6197\|6198\|6446\|6714\|6790\|6850\|6885\|7150\|7294\|7465\|7786\|8317\|8569\|8851\|8877\|9020\|9149\|9212\|9252\|9261\|9475\|9891\|10298\|10461\|10783\|11040\|11200\|23476\|29110\|29904\|51086\|53944\|56848\|415116 | ABL1\|ACVRL1\|GRK2\|AKT1\|ALK\|AXL\|CCND1\|BRAF\|CAMK2B\|CCNB1\|CCND3\|CDK1\|CDK2\|CDK3\|CDK4\|CDK5\|CDK6\|CHEK1\|CLK1\|MAP3K8\|CSNK2A1\|DAPK1\|DYRK1A\|EGFR\|EPHB4\|ERBB2\|ERN1\|FGFR1\|FLT1\|FLT3\|FLT4\|MTOR\|GRK6\|GSK3B\|HCK\|IGF1R\|IKBKB\|INSR\|ITK\|JAK1\|JAK3\|KDR\|KIT\|LCK\|MET\|MYLK\|NEK1\|NEK2\|NTRK1\|PDGFRA\|PDGFRB\|PDK1\|PDPK1\|PFKFB3\|PIK3CA\|PIK3CB\|PIM1\|PIK3CD\|PIK3CG\|PI4KB\|PLK1\|PRKACA\|PRKCA\|PKN1\|PRKDC\|MAPK1\|MAPK3\|MAPK10\|PTK2\|PTK6\|RAF1\|RET\|ROCK1\|ROS1\|RPS6KA1\|RPS6KA3\|RPS6KB1\|SGK1\|SRC\|AURKA\|SYK\|MAP3K7\|TOP1\|TXK\|WEE1\|MAP3K12\|CDC7\|MKNK1\|CDK5R1\|SPHK1\|MAP3K14\|DYRK1B\|AURKB\|RPS6KA5\|MAPKAPK2\|ROCK2\|NUAK1\|PAK4\|MERTK\|NEK6\|PIM2\|CHEK2\|BRD4\|TBK1\|EEF2K\|TNNI3K\|CSNK1G1\|SPHK2\|PIM3 | -66.33697977 |
|  |  | GO:1901699 | GO Biological Processes | cellular response to nitrogen compound | -68.02645941 | 8.82277525 | 27.69395509 | 25\|107\|111\|142\|154\|156\|185\|196\|207\|231\|328\|351\|383\|598\|760\|890\|896\|1017\|1019\|1020\|1080\|1128\|1129\|1131\|1132\|1133\|1136\|1137\|1139\|1141\|1394\|1786\|1812\|1816\|1956\|2101\|2475\|2529\|2554\|2562\|2566\|2642\|2902\|2915\|2932\|2946\|2950\|3066\|3269\|3351\|3480\|3643\|3718\|4193\|4313\|4314\|4363\|4914\|4988\|5024\|5139\|5140\|5142\|5144\|5156\|5170\|5290\|5294\|5295\|5319\|5468\|5566\|5591\|5594\|5595\|5724\|5743\|5747\|5770\|5970\|6093\|6198\|6532\|6622\|6714\|6715\|6772\|6778\|7124\|7184\|7430\|7442\|7532\|8851\|9475\|9734\|9971\|10014\|11255\|22933\|23028\|23411\|23621\|29904\|50507\|59340 | ABL1\|ADCY1\|ADCY5\|PARP1\|ADRB2\|GRK2\|AGTR1\|AHR\|AKT1\|AKR1B1\|APEX1\|APP\|ARG1\|BCL2L1\|CA2\|CCNA2\|CCND3\|CDK2\|CDK4\|CDK5\|CFTR\|CHRM1\|CHRM2\|CHRM3\|CHRM4\|CHRM5\|CHRNA3\|CHRNA4\|CHRNA7\|CHRNB2\|CRHR1\|DNMT1\|DRD1\|DRD5\|EGFR\|ESRRA\|MTOR\|FUT7\|GABRA1\|GABRB3\|GABRG2\|GCGR\|GRIN1\|GRM5\|GSK3B\|GSTM2\|GSTP1\|HDAC2\|HRH1\|HTR1B\|IGF1R\|INSR\|JAK3\|MDM2\|MMP2\|MMP3\|ABCC1\|NTRK1\|OPRM1\|P2RX3\|PDE3A\|PDE3B\|PDE4B\|PDE4D\|PDGFRA\|PDPK1\|PIK3CA\|PIK3CG\|PIK3R1\|PLA2G1B\|PPARG\|PRKACA\|PRKDC\|MAPK1\|MAPK3\|PTAFR\|PTGS2\|PTK2\|PTPN1\|RELA\|ROCK1\|RPS6KB1\|SLC6A4\|SNCA\|SRC\|SRD5A1\|STAT1\|STAT6\|TNF\|HSP90B1\|EZR\|TRPV1\|YWHAG\|CDK5R1\|ROCK2\|HDAC9\|NR1H4\|HDAC5\|HRH3\|SIRT2\|KDM1A\|SIRT1\|BACE1\|EEF2K\|NOX4\|HRH4 | -64.27698591 |
|  |  | GO:0016301 | GO Molecular Functions | kinase activity | -66.93106753 | 8.141125461 | 26.85066872 | 25\|94\|156\|207\|238\|558\|595\|673\|816\|891\|896\|898\|983\|1017\|1018\|1019\|1020\|1021\|1111\|1195\|1326\|1457\|1612\|1859\|1956\|2050\|2064\|2081\|2260\|2321\|2322\|2324\|2475\|2870\|2932\|3055\|3480\|3551\|3643\|3702\|3716\|3718\|3791\|3815\|3932\|4233\|4638\|4750\|4751\|4914\|5156\|5159\|5163\|5170\|5209\|5290\|5291\|5292\|5293\|5294\|5298\|5347\|5566\|5578\|5585\|5591\|5594\|5595\|5602\|5747\|5753\|5894\|5979\|6093\|6098\|6195\|6197\|6198\|6446\|6714\|6790\|6850\|6885\|7150\|7294\|7465\|7786\|8317\|8569\|8851\|8877\|9020\|9149\|9212\|9252\|9261\|9475\|9891\|10298\|10461\|10783\|11040\|11200\|23476\|29110\|29904\|51086\|53944\|56848\|415116 | ABL1\|ACVRL1\|GRK2\|AKT1\|ALK\|AXL\|CCND1\|BRAF\|CAMK2B\|CCNB1\|CCND3\|CCNE1\|CDK1\|CDK2\|CDK3\|CDK4\|CDK5\|CDK6\|CHEK1\|CLK1\|MAP3K8\|CSNK2A1\|DAPK1\|DYRK1A\|EGFR\|EPHB4\|ERBB2\|ERN1\|FGFR1\|FLT1\|FLT3\|FLT4\|MTOR\|GRK6\|GSK3B\|HCK\|IGF1R\|IKBKB\|INSR\|ITK\|JAK1\|JAK3\|KDR\|KIT\|LCK\|MET\|MYLK\|NEK1\|NEK2\|NTRK1\|PDGFRA\|PDGFRB\|PDK1\|PDPK1\|PFKFB3\|PIK3CA\|PIK3CB\|PIM1\|PIK3CD\|PIK3CG\|PI4KB\|PLK1\|PRKACA\|PRKCA\|PKN1\|PRKDC\|MAPK1\|MAPK3\|MAPK10\|PTK2\|PTK6\|RAF1\|RET\|ROCK1\|ROS1\|RPS6KA1\|RPS6KA3\|RPS6KB1\|SGK1\|SRC\|AURKA\|SYK\|MAP3K7\|TOP1\|TXK\|WEE1\|MAP3K12\|CDC7\|MKNK1\|CDK5R1\|SPHK1\|MAP3K14\|DYRK1B\|AURKB\|RPS6KA5\|MAPKAPK2\|ROCK2\|NUAK1\|PAK4\|MERTK\|NEK6\|PIM2\|CHEK2\|BRD4\|TBK1\|EEF2K\|TNNI3K\|CSNK1G1\|SPHK2\|PIM3 | -63.26077528 |
|  |  | GO:0099536 | GO Biological Processes | synaptic signaling | -61.08700114 | 7.780163279 | 25.35321498 | 25\|43\|107\|134\|135\|136\|148\|154\|207\|351\|590\|760\|766\|816\|952\|1020\|1128\|1129\|1131\|1132\|1133\|1134\|1135\|1136\|1137\|1138\|1139\|1140\|1141\|1142\|1143\|1144\|1146\|1268\|1269\|1759\|1812\|1813\|1814\|1815\|1816\|1956\|2475\|2554\|2556\|2558\|2559\|2562\|2566\|2902\|2904\|2911\|2912\|2913\|2914\|2915\|2916\|2918\|2932\|3269\|3274\|3350\|3351\|3352\|3355\|3356\|3357\|3358\|3359\|3361\|3362\|3363\|3815\|4137\|4311\|4914\|4986\|4988\|5024\|5340\|5566\|5594\|5743\|5802\|5970\|6530\|6531\|6532\|6536\|6571\|6582\|6622\|6714\|7124\|7442\|7532\|8973\|9177\|11255\|23621\|27163\|54583\|59340\|89780 | ABL1\|ACHE\|ADCY1\|ADORA1\|ADORA2A\|ADORA2B\|ADRA1A\|ADRB2\|AKT1\|APP\|BCHE\|CA2\|CA7\|CAMK2B\|CD38\|CDK5\|CHRM1\|CHRM2\|CHRM3\|CHRM4\|CHRM5\|CHRNA1\|CHRNA2\|CHRNA3\|CHRNA4\|CHRNA5\|CHRNA7\|CHRNB1\|CHRNB2\|CHRNB3\|CHRNB4\|CHRND\|CHRNG\|CNR1\|CNR2\|DNM1\|DRD1\|DRD2\|DRD3\|DRD4\|DRD5\|EGFR\|MTOR\|GABRA1\|GABRA3\|GABRA5\|GABRA6\|GABRB3\|GABRG2\|GRIN1\|GRIN2B\|GRM1\|GRM2\|GRM3\|GRM4\|GRM5\|GRM6\|GRM8\|GSK3B\|HRH1\|HRH2\|HTR1A\|HTR1B\|HTR1D\|HTR1F\|HTR2A\|HTR2B\|HTR2C\|HTR3A\|HTR5A\|HTR6\|HTR7\|KIT\|MAPT\|MME\|NTRK1\|OPRK1\|OPRM1\|P2RX3\|PLG\|PRKACA\|MAPK1\|PTGS2\|PTPRS\|RELA\|SLC6A2\|SLC6A3\|SLC6A4\|SLC6A9\|SLC18A2\|SLC22A2\|SNCA\|SRC\|TNF\|TRPV1\|YWHAG\|CHRNA6\|HTR3B\|HRH3\|BACE1\|NAAA\|EGLN1\|HRH4\|WNT3A | -57.5647932 |
|  |  | GO:0098916 | GO Biological Processes | anterograde trans-synaptic signaling | -61.05899419 | 7.886280838 | 25.44499588 | 25\|43\|107\|134\|135\|136\|148\|154\|207\|351\|590\|760\|766\|816\|952\|1020\|1128\|1129\|1131\|1132\|1133\|1134\|1135\|1136\|1137\|1138\|1139\|1140\|1141\|1142\|1143\|1144\|1146\|1268\|1269\|1759\|1812\|1813\|1814\|1815\|1816\|1956\|2475\|2554\|2556\|2558\|2559\|2562\|2566\|2902\|2904\|2911\|2912\|2913\|2914\|2915\|2916\|2918\|2932\|3269\|3274\|3350\|3351\|3352\|3355\|3356\|3357\|3358\|3359\|3361\|3362\|3363\|3815\|4137\|4311\|4914\|4986\|4988\|5024\|5340\|5566\|5594\|5743\|5802\|6530\|6531\|6532\|6536\|6571\|6582\|6622\|6714\|7124\|7442\|7532\|8973\|9177\|11255\|23621\|27163\|54583\|59340\|89780 | ABL1\|ACHE\|ADCY1\|ADORA1\|ADORA2A\|ADORA2B\|ADRA1A\|ADRB2\|AKT1\|APP\|BCHE\|CA2\|CA7\|CAMK2B\|CD38\|CDK5\|CHRM1\|CHRM2\|CHRM3\|CHRM4\|CHRM5\|CHRNA1\|CHRNA2\|CHRNA3\|CHRNA4\|CHRNA5\|CHRNA7\|CHRNB1\|CHRNB2\|CHRNB3\|CHRNB4\|CHRND\|CHRNG\|CNR1\|CNR2\|DNM1\|DRD1\|DRD2\|DRD3\|DRD4\|DRD5\|EGFR\|MTOR\|GABRA1\|GABRA3\|GABRA5\|GABRA6\|GABRB3\|GABRG2\|GRIN1\|GRIN2B\|GRM1\|GRM2\|GRM3\|GRM4\|GRM5\|GRM6\|GRM8\|GSK3B\|HRH1\|HRH2\|HTR1A\|HTR1B\|HTR1D\|HTR1F\|HTR2A\|HTR2B\|HTR2C\|HTR3A\|HTR5A\|HTR6\|HTR7\|KIT\|MAPT\|MME\|NTRK1\|OPRK1\|OPRM1\|P2RX3\|PLG\|PRKACA\|MAPK1\|PTGS2\|PTPRS\|SLC6A2\|SLC6A3\|SLC6A4\|SLC6A9\|SLC18A2\|SLC22A2\|SNCA\|SRC\|TNF\|TRPV1\|YWHAG\|CHRNA6\|HTR3B\|HRH3\|BACE1\|NAAA\|EGLN1\|HRH4\|WNT3A | -57.5647932 |
|  |  | GO:0007268 | GO Biological Processes | chemical synaptic transmission | -61.05899419 | 7.886280838 | 25.44499588 | 25\|43\|107\|134\|135\|136\|148\|154\|207\|351\|590\|760\|766\|816\|952\|1020\|1128\|1129\|1131\|1132\|1133\|1134\|1135\|1136\|1137\|1138\|1139\|1140\|1141\|1142\|1143\|1144\|1146\|1268\|1269\|1759\|1812\|1813\|1814\|1815\|1816\|1956\|2475\|2554\|2556\|2558\|2559\|2562\|2566\|2902\|2904\|2911\|2912\|2913\|2914\|2915\|2916\|2918\|2932\|3269\|3274\|3350\|3351\|3352\|3355\|3356\|3357\|3358\|3359\|3361\|3362\|3363\|3815\|4137\|4311\|4914\|4986\|4988\|5024\|5340\|5566\|5594\|5743\|5802\|6530\|6531\|6532\|6536\|6571\|6582\|6622\|6714\|7124\|7442\|7532\|8973\|9177\|11255\|23621\|27163\|54583\|59340\|89780 | ABL1\|ACHE\|ADCY1\|ADORA1\|ADORA2A\|ADORA2B\|ADRA1A\|ADRB2\|AKT1\|APP\|BCHE\|CA2\|CA7\|CAMK2B\|CD38\|CDK5\|CHRM1\|CHRM2\|CHRM3\|CHRM4\|CHRM5\|CHRNA1\|CHRNA2\|CHRNA3\|CHRNA4\|CHRNA5\|CHRNA7\|CHRNB1\|CHRNB2\|CHRNB3\|CHRNB4\|CHRND\|CHRNG\|CNR1\|CNR2\|DNM1\|DRD1\|DRD2\|DRD3\|DRD4\|DRD5\|EGFR\|MTOR\|GABRA1\|GABRA3\|GABRA5\|GABRA6\|GABRB3\|GABRG2\|GRIN1\|GRIN2B\|GRM1\|GRM2\|GRM3\|GRM4\|GRM5\|GRM6\|GRM8\|GSK3B\|HRH1\|HRH2\|HTR1A\|HTR1B\|HTR1D\|HTR1F\|HTR2A\|HTR2B\|HTR2C\|HTR3A\|HTR5A\|HTR6\|HTR7\|KIT\|MAPT\|MME\|NTRK1\|OPRK1\|OPRM1\|P2RX3\|PLG\|PRKACA\|MAPK1\|PTGS2\|PTPRS\|SLC6A2\|SLC6A3\|SLC6A4\|SLC6A9\|SLC18A2\|SLC22A2\|SNCA\|SRC\|TNF\|TRPV1\|YWHAG\|CHRNA6\|HTR3B\|HRH3\|BACE1\|NAAA\|EGLN1\|HRH4\|WNT3A | -57.5647932 |
|  |  | GO:0030594 | GO Molecular Functions | neurotransmitter receptor activity | -60.72103836 | 25.54768833 | 35.75294867 | 134\|153\|706\|1128\|1129\|1131\|1132\|1133\|1134\|1135\|1136\|1137\|1138\|1139\|1140\|1141\|1142\|1143\|1144\|1146\|1812\|1813\|1814\|1815\|1816\|2554\|2556\|2558\|2559\|2562\|2566\|2902\|2904\|2911\|2915\|3269\|3274\|3350\|3351\|3352\|3355\|3356\|3357\|3358\|3359\|3361\|3362\|3363\|4988\|8973\|9177\|11255\|59340 | ADORA1\|ADRB1\|TSPO\|CHRM1\|CHRM2\|CHRM3\|CHRM4\|CHRM5\|CHRNA1\|CHRNA2\|CHRNA3\|CHRNA4\|CHRNA5\|CHRNA7\|CHRNB1\|CHRNB2\|CHRNB3\|CHRNB4\|CHRND\|CHRNG\|DRD1\|DRD2\|DRD3\|DRD4\|DRD5\|GABRA1\|GABRA3\|GABRA5\|GABRA6\|GABRB3\|GABRG2\|GRIN1\|GRIN2B\|GRM1\|GRM5\|HRH1\|HRH2\|HTR1A\|HTR1B\|HTR1D\|HTR1F\|HTR2A\|HTR2B\|HTR2C\|HTR3A\|HTR5A\|HTR6\|HTR7\|OPRM1\|CHRNA6\|HTR3B\|HRH3\|HRH4 | -57.27259486 |
|  |  | GO:0099537 | GO Biological Processes | trans-synaptic signaling | -60.58041468 | 7.800091976 | 25.2686716 | 25\|43\|107\|134\|135\|136\|148\|154\|207\|351\|590\|760\|766\|816\|952\|1020\|1128\|1129\|1131\|1132\|1133\|1134\|1135\|1136\|1137\|1138\|1139\|1140\|1141\|1142\|1143\|1144\|1146\|1268\|1269\|1759\|1812\|1813\|1814\|1815\|1816\|1956\|2475\|2554\|2556\|2558\|2559\|2562\|2566\|2902\|2904\|2911\|2912\|2913\|2914\|2915\|2916\|2918\|2932\|3269\|3274\|3350\|3351\|3352\|3355\|3356\|3357\|3358\|3359\|3361\|3362\|3363\|3815\|4137\|4311\|4914\|4986\|4988\|5024\|5340\|5566\|5594\|5743\|5802\|6530\|6531\|6532\|6536\|6571\|6582\|6622\|6714\|7124\|7442\|7532\|8973\|9177\|11255\|23621\|27163\|54583\|59340\|89780 | ABL1\|ACHE\|ADCY1\|ADORA1\|ADORA2A\|ADORA2B\|ADRA1A\|ADRB2\|AKT1\|APP\|BCHE\|CA2\|CA7\|CAMK2B\|CD38\|CDK5\|CHRM1\|CHRM2\|CHRM3\|CHRM4\|CHRM5\|CHRNA1\|CHRNA2\|CHRNA3\|CHRNA4\|CHRNA5\|CHRNA7\|CHRNB1\|CHRNB2\|CHRNB3\|CHRNB4\|CHRND\|CHRNG\|CNR1\|CNR2\|DNM1\|DRD1\|DRD2\|DRD3\|DRD4\|DRD5\|EGFR\|MTOR\|GABRA1\|GABRA3\|GABRA5\|GABRA6\|GABRB3\|GABRG2\|GRIN1\|GRIN2B\|GRM1\|GRM2\|GRM3\|GRM4\|GRM5\|GRM6\|GRM8\|GSK3B\|HRH1\|HRH2\|HTR1A\|HTR1B\|HTR1D\|HTR1F\|HTR2A\|HTR2B\|HTR2C\|HTR3A\|HTR5A\|HTR6\|HTR7\|KIT\|MAPT\|MME\|NTRK1\|OPRK1\|OPRM1\|P2RX3\|PLG\|PRKACA\|MAPK1\|PTGS2\|PTPRS\|SLC6A2\|SLC6A3\|SLC6A4\|SLC6A9\|SLC18A2\|SLC22A2\|SNCA\|SRC\|TNF\|TRPV1\|YWHAG\|CHRNA6\|HTR3B\|HRH3\|BACE1\|NAAA\|EGLN1\|HRH4\|WNT3A | -57.17336386 |
|  | VMC-related targets | hsa05200 | KEGG Pathway | Pathways in cancer | -37.40857866 | 18.50038261 | 25.38779507 | 25\|107\|111\|185\|207\|595\|596\|1909\|1956\|2064\|2147\|2475\|3320\|3551\|3558\|3674\|3815\|4233\|4312\|4313\|4318\|4843\|4914\|5228\|5290\|5468\|5566\|5578\|5594\|5595\|5747\|5894\|5970\|5979\|6198\|6772\|7422\|9252 | ABL1\|ADCY1\|ADCY5\|AGTR1\|AKT1\|CCND1\|BCL2\|EDNRA\|EGFR\|ERBB2\|F2\|MTOR\|HSP90AA1\|IKBKB\|IL2\|ITGA2B\|KIT\|MET\|MMP1\|MMP2\|MMP9\|NOS2\|NTRK1\|PGF\|PIK3CA\|PPARG\|PRKACA\|PRKCA\|MAPK1\|MAPK3\|PTK2\|RAF1\|RELA\|RET\|RPS6KB1\|STAT1\|VEGFA\|RPS6KA5 | -32.96013516 |
|  |  | GO:0008015 | GO Biological Processes | blood circulation | -30.8479974 | 16.85770073 | 22.44710257 | 94\|135\|146\|153\|154\|155\|185\|207\|1129\|1215\|1813\|1909\|1956\|2475\|3269\|3274\|3558\|3757\|4843\|5290\|5294\|5465\|5468\|5566\|5972\|6331\|6532\|6714\|6772\|7134\|7137\|7139\|7422 | ACVRL1\|ADORA2A\|ADRA1D\|ADRB1\|ADRB2\|ADRB3\|AGTR1\|AKT1\|CHRM2\|CMA1\|DRD2\|EDNRA\|EGFR\|MTOR\|HRH1\|HRH2\|IL2\|KCNH2\|NOS2\|PIK3CA\|PIK3CG\|PPARA\|PPARG\|PRKACA\|REN\|SCN5A\|SLC6A4\|SRC\|STAT1\|TNNC1\|TNNI3\|TNNT2\|VEGFA | -26.7005839 |
|  |  | GO:0003013 | GO Biological Processes | circulatory system process | -30.56591037 | 16.52597496 | 22.20146468 | 94\|135\|146\|153\|154\|155\|185\|207\|1129\|1215\|1813\|1909\|1956\|2475\|3269\|3274\|3558\|3757\|4843\|5290\|5294\|5465\|5468\|5566\|5972\|6331\|6532\|6714\|6772\|7134\|7137\|7139\|7422 | ACVRL1\|ADORA2A\|ADRA1D\|ADRB1\|ADRB2\|ADRB3\|AGTR1\|AKT1\|CHRM2\|CMA1\|DRD2\|EDNRA\|EGFR\|MTOR\|HRH1\|HRH2\|IL2\|KCNH2\|NOS2\|PIK3CA\|PIK3CG\|PPARA\|PPARG\|PRKACA\|REN\|SCN5A\|SLC6A4\|SRC\|STAT1\|TNNC1\|TNNI3\|TNNT2\|VEGFA | -26.59458812 |
|  |  | GO:0033674 | GO Biological Processes | positive regulation of kinase activity | -29.21649725 | 15.02117073 | 21.05146714 | 25\|107\|111\|154\|207\|595\|1813\|1956\|1991\|2064\|2147\|2321\|2475\|3320\|3558\|3690\|3815\|4233\|4282\|4914\|5290\|5294\|5566\|5594\|5595\|5747\|5894\|5979\|6714\|6868\|7124\|7422\|54106 | ABL1\|ADCY1\|ADCY5\|ADRB2\|AKT1\|CCND1\|DRD2\|EGFR\|ELANE\|ERBB2\|F2\|FLT1\|MTOR\|HSP90AA1\|IL2\|ITGB3\|KIT\|MET\|MIF\|NTRK1\|PIK3CA\|PIK3CG\|PRKACA\|MAPK1\|MAPK3\|PTK2\|RAF1\|RET\|SRC\|ADAM17\|TNF\|VEGFA\|TLR9 | -25.37011374 |
|  |  | GO:0051347 | GO Biological Processes | positive regulation of transferase activity | -28.80192749 | 13.67522989 | 20.2751639 | 25\|107\|111\|154\|207\|595\|1813\|1956\|1991\|2064\|2147\|2321\|2475\|3320\|3558\|3690\|3815\|4233\|4282\|4914\|5111\|5290\|5294\|5566\|5594\|5595\|5747\|5894\|5979\|6714\|6868\|7124\|7422\|54106 | ABL1\|ADCY1\|ADCY5\|ADRB2\|AKT1\|CCND1\|DRD2\|EGFR\|ELANE\|ERBB2\|F2\|FLT1\|MTOR\|HSP90AA1\|IL2\|ITGB3\|KIT\|MET\|MIF\|NTRK1\|PCNA\|PIK3CA\|PIK3CG\|PRKACA\|MAPK1\|MAPK3\|PTK2\|RAF1\|RET\|SRC\|ADAM17\|TNF\|VEGFA\|TLR9 | -25.05245399 |
|  |  | hsa04151 | KEGG Pathway | PI3K-Akt signaling pathway | -28.40456994 | 20.62715789 | 23.06502226 | 207\|595\|596\|1129\|1956\|2064\|2321\|2475\|3320\|3551\|3558\|3674\|3690\|3815\|4233\|4914\|5228\|5290\|5294\|5578\|5594\|5595\|5747\|5894\|5970\|6198\|7134\|7422 | AKT1\|CCND1\|BCL2\|CHRM2\|EGFR\|ERBB2\|FLT1\|MTOR\|HSP90AA1\|IKBKB\|IL2\|ITGA2B\|ITGB3\|KIT\|MET\|NTRK1\|PGF\|PIK3CA\|PIK3CG\|PRKCA\|MAPK1\|MAPK3\|PTK2\|RAF1\|RELA\|RPS6KB1\|TNNC1\|VEGFA | -24.73427769 |
|  |  | GO:0045860 | GO Biological Processes | positive regulation of protein kinase activity | -26.83977495 | 15.52347505 | 20.42413132 | 25\|107\|111\|154\|207\|595\|1813\|1956\|1991\|2064\|2321\|2475\|3320\|3690\|3815\|4282\|4914\|5290\|5294\|5566\|5594\|5595\|5747\|5894\|5979\|6714\|6868\|7124\|7422\|54106 | ABL1\|ADCY1\|ADCY5\|ADRB2\|AKT1\|CCND1\|DRD2\|EGFR\|ELANE\|ERBB2\|FLT1\|MTOR\|HSP90AA1\|ITGB3\|KIT\|MIF\|NTRK1\|PIK3CA\|PIK3CG\|PRKACA\|MAPK1\|MAPK3\|PTK2\|RAF1\|RET\|SRC\|ADAM17\|TNF\|VEGFA\|TLR9 | -23.23642949 |
|  |  | hsa01522 | KEGG Pathway | Endocrine resistance | -25.71008961 | 52.48875 | 30.25734059 | 107\|111\|207\|595\|596\|1956\|2064\|2475\|4313\|4318\|5290\|5566\|5594\|5595\|5747\|5894\|6198\|6714 | ADCY1\|ADCY5\|AKT1\|CCND1\|BCL2\|EGFR\|ERBB2\|MTOR\|MMP2\|MMP9\|PIK3CA\|PRKACA\|MAPK1\|MAPK3\|PTK2\|RAF1\|RPS6KB1\|SRC | -22.21588862 |
|  |  | GO:0009611 | GO Biological Processes | response to wounding | -24.67997023 | 12.25725989 | 18.16555653 | 94\|135\|240\|383\|596\|706\|1813\|1956\|2064\|2147\|2152\|2475\|3674\|3690\|3932\|4914\|5054\|5290\|5294\|5340\|5465\|5566\|5578\|5594\|5595\|5747\|5894\|6714\|6868\|7124\|7422 | ACVRL1\|ADORA2A\|ALOX5\|ARG1\|BCL2\|TSPO\|DRD2\|EGFR\|ERBB2\|F2\|F3\|MTOR\|ITGA2B\|ITGB3\|LCK\|NTRK1\|SERPINE1\|PIK3CA\|PIK3CG\|PLG\|PPARA\|PRKACA\|PRKCA\|MAPK1\|MAPK3\|PTK2\|RAF1\|SRC\|ADAM17\|TNF\|VEGFA | -21.27291942 |
|  |  | hsa05205 | KEGG Pathway | Proteoglycans in cancer | -23.63122086 | 26.96669725 | 23.04514437 | 207\|595\|1514\|1956\|2064\|2475\|3690\|4233\|4313\|4318\|5290\|5566\|5578\|5594\|5595\|5747\|5894\|6198\|6714\|7124\|7422 | AKT1\|CCND1\|CTSL\|EGFR\|ERBB2\|MTOR\|ITGB3\|MET\|MMP2\|MMP9\|PIK3CA\|PRKACA\|PRKCA\|MAPK1\|MAPK3\|PTK2\|RAF1\|RPS6KB1\|SRC\|TNF\|VEGFA | -20.29672071 |
| JH | whole targets | GO:0008202 | GO Biological Processes | steroid metabolic process | -26.1409164 | 36.66055526 | 26.51817457 | 866|1557|1586|1588|1595|1717|2099|2222|2539|3156|3290|5467|6095|6097|6469|6713|6721|7364|7421|29881 | SERPINA6|CYP2C19|CYP17A1|CYP19A1|CYP51A1|DHCR7|ESR1|FDFT1|G6PD|HMGCR|HSD11B1|PPARD|RORA|RORC|SHH|SQLE|SREBF2|UGT2B7|VDR|NPC1L1 | -21.6924729 |
|  |  | GO:0098531 | GO Molecular Functions | transcription factor activity, direct ligand regulated sequence-specific DNA binding | -22.7981892 | 140.4381271 | 40.83031581 | 367\|2099\|2100\|5465\|5467\|5468\|6095\|6097\|7376\|7421\|9970\|10062 | AR\|ESR1\|ESR2\|PPARA\|PPARD\|PPARG\|RORA\|RORC\|NR1H2\|VDR\|NR1I3\|NR1H3 | -18.82686696 |
|  |  | GO:0004879 | GO Molecular Functions | nuclear receptor activity | -22.7981892 | 140.4381271 | 40.83031581 | 367\|2099\|2100\|5465\|5467\|5468\|6095\|6097\|7376\|7421\|9970\|10062 | AR\|ESR1\|ESR2\|PPARA\|PPARD\|PPARG\|RORA\|RORC\|NR1H2\|VDR\|NR1I3\|NR1H3 | -18.82686696 |
|  |  | GO:0062012 | GO Biological Processes | regulation of small molecule metabolic process | -16.55544472 | 20.24333363 | 17.27078765 | 1595\|1717\|2222\|3156\|4843\|5465\|5467\|5468\|5771\|6095\|6097\|6713\|6721\|7376\|7421\|10062 | CYP51A1\|DHCR7\|FDFT1\|HMGCR\|NOS2\|PPARA\|PPARD\|PPARG\|PTPN2\|RORA\|RORC\|SQLE\|SREBF2\|NR1H2\|VDR\|NR1H3 | -12.70906121 |
|  |  | GO:0008610 | GO Biological Processes | lipid biosynthetic process | -16.41212093 | 14.94430275 | 15.5205165 | 43\|1586\|1588\|1595\|1717\|2222\|2539\|3156\|3290\|5467\|6713\|6721\|6916\|7376\|7421\|9536\|10062\|29881 | ACHE\|CYP17A1\|CYP19A1\|CYP51A1\|DHCR7\|FDFT1\|G6PD\|HMGCR\|HSD11B1\|PPARD\|SQLE\|SREBF2\|TBXAS1\|NR1H2\|VDR\|PTGES\|NR1H3\|NPC1L1 | -12.66264743 |
|  |  | GO:0006367 | GO Biological Processes | transcription initiation from RNA polymerase II promoter | -15.69744419 | 38.63906142 | 21.06378961 | 367\|2099\|2100\|5465\|5467\|5468\|6095\|6097\|7376\|7421\|9970\|10062 | AR\|ESR1\|ESR2\|PPARA\|PPARD\|PPARG\|RORA\|RORC\|NR1H2\|VDR\|NR1I3\|NR1H3 | -12.02715195 |
|  |  | GO:0006694 | GO Biological Processes | steroid biosynthetic process | -15.4522314 | 36.88274045 | 20.55679088 | 1586\|1588\|1595\|1717\|2222\|2539\|3156\|3290\|6713\|6721\|7421\|29881 | CYP17A1\|CYP19A1\|CYP51A1\|DHCR7\|FDFT1\|G6PD\|HMGCR\|HSD11B1\|SQLE\|SREBF2\|VDR\|NPC1L1 | -11.84888594 |
|  |  | GO:0005496 | GO Molecular Functions | steroid binding | -14.97447836 | 59.08400169 | 23.95914796 | 367\|866\|2099\|2100\|3290\|6095\|6097\|6462\|7421\|10062 | AR\|SERPINA6\|ESR1\|ESR2\|HSD11B1\|RORA\|RORC\|SHBG\|VDR\|NR1H3 | -11.42912485 |
|  |  | GO:0019216 | GO Biological Processes | regulation of lipid metabolic process | -14.28207069 | 19.76777968 | 15.93002875 | 1595\|1717\|2222\|3156\|5465\|5467\|5468\|6095\|6097\|6713\|6721\|7376\|7421\|10062 | CYP51A1\|DHCR7\|FDFT1\|HMGCR\|PPARA\|PPARD\|PPARG\|RORA\|RORC\|SQLE\|SREBF2\|NR1H2\|VDR\|NR1H3 | -10.80365007 |
|  |  | GO:0006352 | GO Biological Processes | DNA-templated transcription, initiation | -14.25209357 | 29.32844421 | 18.21622171 | 367\|2099\|2100\|5465\|5467\|5468\|6095\|6097\|7376\|7421\|9970\|10062 | AR\|ESR1\|ESR2\|PPARA\|PPARD\|PPARG\|RORA\|RORC\|NR1H2\|VDR\|NR1I3\|NR1H3 | -10.80365007 |
|  | VMC-related targets | GO:0062012 | GO Biological Processes | regulation of small molecule metabolic process | -7.67536795 | 29.0997921 | 12.87308509 | 1595\|2222\|4843\|5465\|5468\|7421 | CYP51A1\|FDFT1\|NOS2\|PPARA\|PPARG\|VDR | -3.22692445 |
|  |  | GO:0008202 | GO Biological Processes | steroid metabolic process | -6.773615132 | 35.13303213 | 12.95621338 | 1557\|1595\|2222\|2539\|7421 | CYP2C19\|CYP51A1\|FDFT1\|G6PD\|VDR | -2.723277397 |
|  |  | GO:0032800 | GO Biological Processes | receptor biosynthetic process | -6.660529582 | 241.3275862 | 26.81459122 | 43\|5465\|5468 | ACHE\|PPARA\|PPARG | -2.723277397 |
|  |  | GO:0001505 | GO Biological Processes | regulation of neurotransmitter levels | -6.569660906 | 31.956621 | 12.32802108 | 43\|1129\|4843\|6530\|6532 | ACHE\|CHRM2\|NOS2\|SLC6A2\|SLC6A4 | -2.723277397 |
|  |  | GO:0046165 | GO Biological Processes | alcohol biosynthetic process | -6.266105032 | 56.89837398 | 14.86758619 | 1595\|2222\|2539\|7421 | CYP51A1\|FDFT1\|G6PD\|VDR | -2.561372971 |
|  |  | GO:0019216 | GO Biological Processes | regulation of lipid metabolic process | -6.212935584 | 27.06303171 | 11.29214104 | 1595\|2222\|5465\|5468\|7421 | CYP51A1\|FDFT1\|PPARA\|PPARG\|VDR | -2.561372971 |
|  |  | GO:0046621 | GO Biological Processes | negative regulation of organ growth | -6.16471843 | 166.6309524 | 22.24516695 | 2539\|5465\|6532 | G6PD\|PPARA\|SLC6A4 | -2.561372971 |
|  |  | GO:0016709 | GO Molecular Functions | oxidoreductase activity, acting on paired donors, with incorporation or reduction of molecular oxygen, NAD(P)H as one donor, and incorporation of one atom of oxygen | -6.072992307 | 155.5222222 | 21.4827579 | 1557\|1595\|4843 | CYP2C19\|CYP51A1\|NOS2 | -2.527638794 |
|  |  | GO:0006694 | GO Biological Processes | steroid biosynthetic process | -5.939444016 | 47.12794613 | 13.48905515 | 1595\|2222\|2539\|7421 | CYP51A1\|FDFT1\|G6PD\|VDR | -2.474265221 |
|  |  | GO:0004879 | GO Molecular Functions | nuclear receptor activity | -5.881316036 | 134.5865385 | 19.96694368 | 5465\|5468\|7421 | PPARA\|PPARG\|VDR | -2.474265221 |
| CH | whole targets | GO:0016773 | GO Molecular Functions | phosphotransferase activity, alcohol group as acceptor | -47.79859513 | 9.178030881 | 23.48739954 | 25|207|238|558|595|673|816|891|896|983|1017|1019|1020|1021|1025|1111|1436|1457|1612|1613|1956|2050|2081|2260|2322|2475|2645|2870|2932|3055|3480|3643|3702|3716|3717|3718|3791|3815|3932|4233|4638|4751|4914|5209|5290|5291|5292|5293|5294|5298|5347|5585|5591|5599|5601|5602|5604|5747|6093|6197|6714|6850|7150|7294|7465|8317|8851|9212|9475|9891|10783|11200|23476 | ABL1|AKT1|ALK|AXL|CCND1|BRAF|CAMK2B|CCNB1|CCND3|CDK1|CDK2|CDK4|CDK5|CDK6|CDK9|CHEK1|CSF1R|CSNK2A1|DAPK1|DAPK3|EGFR|EPHB4|ERN1|FGFR1|FLT3|MTOR|GCK|GRK6|GSK3B|HCK|IGF1R|INSR|ITK|JAK1|JAK2|JAK3|KDR|KIT|LCK|MET|MYLK|NEK2|NTRK1|PFKFB3|PIK3CA|PIK3CB|PIM1|PIK3CD|PIK3CG|PI4KB|PLK1|PKN1|PRKDC|MAPK8|MAPK9|MAPK10|MAP2K1|PTK2|ROCK1|RPS6KA3|SRC|SYK|TOP1|TXK|WEE1|CDC7|CDK5R1|AURKB|ROCK2|NUAK1|NEK6|CHEK2|BRD4 | -43.35015163 |
|  |  | GO:0004672 | GO Molecular Functions | protein kinase activity | -47.19465882 | 10.11999872 | 24.02798525 | 25\|207\|238\|558\|595\|673\|816\|891\|896\|983\|1017\|1019\|1020\|1021\|1025\|1111\|1436\|1457\|1612\|1613\|1956\|2050\|2081\|2260\|2322\|2475\|2870\|2932\|3055\|3480\|3643\|3702\|3716\|3717\|3718\|3791\|3815\|3932\|4233\|4638\|4751\|4914\|5290\|5292\|5294\|5347\|5585\|5591\|5599\|5601\|5602\|5604\|5747\|6093\|6197\|6714\|6850\|7150\|7294\|7465\|8317\|8851\|9212\|9475\|9891\|10783\|11200\|23476 | ABL1\|AKT1\|ALK\|AXL\|CCND1\|BRAF\|CAMK2B\|CCNB1\|CCND3\|CDK1\|CDK2\|CDK4\|CDK5\|CDK6\|CDK9\|CHEK1\|CSF1R\|CSNK2A1\|DAPK1\|DAPK3\|EGFR\|EPHB4\|ERN1\|FGFR1\|FLT3\|MTOR\|GRK6\|GSK3B\|HCK\|IGF1R\|INSR\|ITK\|JAK1\|JAK2\|JAK3\|KDR\|KIT\|LCK\|MET\|MYLK\|NEK2\|NTRK1\|PIK3CA\|PIM1\|PIK3CG\|PLK1\|PKN1\|PRKDC\|MAPK8\|MAPK9\|MAPK10\|MAP2K1\|PTK2\|ROCK1\|RPS6KA3\|SRC\|SYK\|TOP1\|TXK\|WEE1\|CDC7\|CDK5R1\|AURKB\|ROCK2\|NUAK1\|NEK6\|CHEK2\|BRD4 | -43.04724532 |
|  |  | GO:1901699 | GO Biological Processes | cellular response to nitrogen compound | -46.34852454 | 9.154433575 | 23.11913751 | 25\|142\|196\|207\|231\|328\|351\|383\|760\|836\|839\|840\|890\|896\|1017\|1019\|1020\|1080\|1128\|1129\|1131\|1132\|1584\|1585\|1956\|2101\|2475\|2645\|2902\|2915\|2932\|3066\|3480\|3643\|3717\|3718\|4313\|4314\|4363\|4914\|4988\|5024\|5027\|5028\|5138\|5142\|5144\|5290\|5294\|5295\|5319\|5468\|5591\|5663\|5724\|5743\|5747\|5770\|5771\|6093\|6532\|6622\|6714\|7124\|7442\|8851\|9475\|10135\|23411\|23621\|50507 | ABL1\|PARP1\|AHR\|AKT1\|AKR1B1\|APEX1\|APP\|ARG1\|CA2\|CASP3\|CASP6\|CASP7\|CCNA2\|CCND3\|CDK2\|CDK4\|CDK5\|CFTR\|CHRM1\|CHRM2\|CHRM3\|CHRM4\|CYP11B1\|CYP11B2\|EGFR\|ESRRA\|MTOR\|GCK\|GRIN1\|GRM5\|GSK3B\|HDAC2\|IGF1R\|INSR\|JAK2\|JAK3\|MMP2\|MMP3\|ABCC1\|NTRK1\|OPRM1\|P2RX3\|P2RX7\|P2RY1\|PDE2A\|PDE4B\|PDE4D\|PIK3CA\|PIK3CG\|PIK3R1\|PLA2G1B\|PPARG\|PRKDC\|PSEN1\|PTAFR\|PTGS2\|PTK2\|PTPN1\|PTPN2\|ROCK1\|SLC6A4\|SNCA\|SRC\|TNF\|TRPV1\|CDK5R1\|ROCK2\|NAMPT\|SIRT1\|BACE1\|NOX4 | -42.37720229 |
|  |  | GO:0016301 | GO Molecular Functions | kinase activity | -46.04732951 | 8.483933588 | 22.5357526 | 25\|207\|238\|558\|595\|673\|816\|891\|896\|898\|983\|1017\|1019\|1020\|1021\|1025\|1111\|1436\|1457\|1612\|1613\|1956\|2050\|2081\|2260\|2322\|2475\|2645\|2870\|2932\|3055\|3480\|3643\|3702\|3716\|3717\|3718\|3791\|3815\|3932\|4233\|4638\|4751\|4914\|5209\|5290\|5291\|5292\|5293\|5294\|5298\|5347\|5585\|5591\|5599\|5601\|5602\|5604\|5747\|6093\|6197\|6714\|6850\|7150\|7294\|7465\|8317\|8851\|9212\|9475\|9891\|10783\|11200\|23476 | ABL1\|AKT1\|ALK\|AXL\|CCND1\|BRAF\|CAMK2B\|CCNB1\|CCND3\|CCNE1\|CDK1\|CDK2\|CDK4\|CDK5\|CDK6\|CDK9\|CHEK1\|CSF1R\|CSNK2A1\|DAPK1\|DAPK3\|EGFR\|EPHB4\|ERN1\|FGFR1\|FLT3\|MTOR\|GCK\|GRK6\|GSK3B\|HCK\|IGF1R\|INSR\|ITK\|JAK1\|JAK2\|JAK3\|KDR\|KIT\|LCK\|MET\|MYLK\|NEK2\|NTRK1\|PFKFB3\|PIK3CA\|PIK3CB\|PIM1\|PIK3CD\|PIK3CG\|PI4KB\|PLK1\|PKN1\|PRKDC\|MAPK8\|MAPK9\|MAPK10\|MAP2K1\|PTK2\|ROCK1\|RPS6KA3\|SRC\|SYK\|TOP1\|TXK\|WEE1\|CDC7\|CDK5R1\|AURKB\|ROCK2\|NUAK1\|NEK6\|CHEK2\|BRD4 | -42.200946 |
|  |  | hsa05200 | KEGG Pathway | Pathways in cancer | -42.75783245 | 9.557834089 | 22.50694853 | 25\|207\|231\|238\|367\|595\|623\|673\|816\|836\|840\|894\|896\|898\|1017\|1019\|1021\|1436\|1612\|1613\|1956\|2099\|2100\|2147\|2149\|2260\|2322\|2475\|2932\|3066\|3480\|3558\|3685\|3716\|3717\|3718\|3815\|4233\|4312\|4313\|4318\|4843\|4914\|5290\|5291\|5292\|5293\|5295\|5467\|5468\|5599\|5601\|5602\|5604\|5731\|5732\|5743\|5747\|6093\|6608\|7015\|8900\|9134\|9475 | ABL1\|AKT1\|AKR1B1\|ALK\|AR\|CCND1\|BDKRB1\|BRAF\|CAMK2B\|CASP3\|CASP7\|CCND2\|CCND3\|CCNE1\|CDK2\|CDK4\|CDK6\|CSF1R\|DAPK1\|DAPK3\|EGFR\|ESR1\|ESR2\|F2\|F2R\|FGFR1\|FLT3\|MTOR\|GSK3B\|HDAC2\|IGF1R\|IL2\|ITGAV\|JAK1\|JAK2\|JAK3\|KIT\|MET\|MMP1\|MMP2\|MMP9\|NOS2\|NTRK1\|PIK3CA\|PIK3CB\|PIM1\|PIK3CD\|PIK3R1\|PPARD\|PPARG\|MAPK8\|MAPK9\|MAPK10\|MAP2K1\|PTGER1\|PTGER2\|PTGS2\|PTK2\|ROCK1\|SMO\|TERT\|CCNA1\|CCNE2\|ROCK2 | -39.00835895 |
|  |  | GO:0071417 | GO Biological Processes | cellular response to organonitrogen compound | -41.4748505 | 9.114020898 | 21.86429471 | 25\|142\|196\|207\|231\|328\|351\|383\|760\|836\|839\|840\|890\|896\|1019\|1020\|1080\|1584\|1585\|1956\|2101\|2475\|2645\|2902\|2915\|2932\|3066\|3480\|3643\|3717\|3718\|4313\|4363\|4914\|4988\|5024\|5027\|5028\|5138\|5142\|5144\|5290\|5294\|5295\|5319\|5468\|5591\|5663\|5724\|5743\|5747\|5770\|5771\|6093\|6532\|6622\|6714\|7124\|7442\|9475\|10135\|23411\|23621\|50507 | ABL1\|PARP1\|AHR\|AKT1\|AKR1B1\|APEX1\|APP\|ARG1\|CA2\|CASP3\|CASP6\|CASP7\|CCNA2\|CCND3\|CDK4\|CDK5\|CFTR\|CYP11B1\|CYP11B2\|EGFR\|ESRRA\|MTOR\|GCK\|GRIN1\|GRM5\|GSK3B\|HDAC2\|IGF1R\|INSR\|JAK2\|JAK3\|MMP2\|ABCC1\|NTRK1\|OPRM1\|P2RX3\|P2RX7\|P2RY1\|PDE2A\|PDE4B\|PDE4D\|PIK3CA\|PIK3CG\|PIK3R1\|PLA2G1B\|PPARG\|PRKDC\|PSEN1\|PTAFR\|PTGS2\|PTK2\|PTPN1\|PTPN2\|ROCK1\|SLC6A4\|SNCA\|SRC\|TNF\|TRPV1\|ROCK2\|NAMPT\|SIRT1\|BACE1\|NOX4 | -37.80455825 |
|  |  | GO:0003013 | GO Biological Processes | circulatory system process | -39.07783027 | 9.216940856 | 21.30140361 | 100\|134\|135\|140\|150\|152\|207\|239\|367\|554\|623\|952\|1129\|1131\|1215\|1268\|1584\|1585\|1813\|1889\|1956\|2149\|2150\|2475\|3156\|3274\|3291\|3350\|3356\|3363\|3558\|3717\|3760\|3762\|3777\|4311\|4842\|4843\|5028\|5138\|5142\|5144\|5290\|5294\|5465\|5467\|5468\|5547\|5724\|5732\|5743\|6093\|6532\|6714\|6870\|7442\|8654\|9475\|10135\|50507 | ADA\|ADORA1\|ADORA2A\|ADORA3\|ADRA2A\|ADRA2C\|AKT1\|ALOX12\|AR\|AVPR2\|BDKRB1\|CD38\|CHRM2\|CHRM3\|CMA1\|CNR1\|CYP11B1\|CYP11B2\|DRD2\|ECE1\|EGFR\|F2R\|F2RL1\|MTOR\|HMGCR\|HRH2\|HSD11B2\|HTR1A\|HTR2A\|HTR7\|IL2\|JAK2\|KCNJ3\|KCNJ5\|KCNK3\|MME\|NOS1\|NOS2\|P2RY1\|PDE2A\|PDE4B\|PDE4D\|PIK3CA\|PIK3CG\|PPARA\|PPARD\|PPARG\|PRCP\|PTAFR\|PTGER2\|PTGS2\|ROCK1\|SLC6A4\|SRC\|TACR3\|TRPV1\|PDE5A\|ROCK2\|NAMPT\|NOX4 | -35.47448481 |
|  |  | GO:0008015 | GO Biological Processes | blood circulation | -38.48235988 | 9.245253235 | 21.15920698 | 100\|134\|135\|140\|150\|152\|207\|239\|367\|554\|623\|952\|1129\|1131\|1215\|1268\|1584\|1585\|1813\|1889\|1956\|2149\|2150\|2475\|3156\|3274\|3291\|3350\|3356\|3363\|3558\|3717\|3760\|3762\|3777\|4311\|4842\|4843\|5028\|5138\|5142\|5144\|5290\|5294\|5465\|5467\|5468\|5547\|5724\|5732\|5743\|6093\|6532\|6714\|6870\|7442\|8654\|9475\|10135 | ADA\|ADORA1\|ADORA2A\|ADORA3\|ADRA2A\|ADRA2C\|AKT1\|ALOX12\|AR\|AVPR2\|BDKRB1\|CD38\|CHRM2\|CHRM3\|CMA1\|CNR1\|CYP11B1\|CYP11B2\|DRD2\|ECE1\|EGFR\|F2R\|F2RL1\|MTOR\|HMGCR\|HRH2\|HSD11B2\|HTR1A\|HTR2A\|HTR7\|IL2\|JAK2\|KCNJ3\|KCNJ5\|KCNK3\|MME\|NOS1\|NOS2\|P2RY1\|PDE2A\|PDE4B\|PDE4D\|PIK3CA\|PIK3CG\|PPARA\|PPARD\|PPARG\|PRCP\|PTAFR\|PTGER2\|PTGS2\|ROCK1\|SLC6A4\|SRC\|TACR3\|TRPV1\|PDE5A\|ROCK2\|NAMPT | -34.98815889 |
|  |  | GO:0051347 | GO Biological Processes | positive regulation of transferase activity | -37.66257264 | 7.89619914 | 19.99832572 | 25\|134\|150\|152\|207\|238\|558\|595\|673\|891\|894\|896\|983\|1020\|1436\|1813\|1815\|1956\|1991\|2050\|2081\|2147\|2149\|2260\|2322\|2475\|2914\|2915\|3356\|3480\|3558\|3643\|3690\|3717\|3791\|3815\|4137\|4233\|4751\|4914\|5027\|5290\|5291\|5294\|5319\|5347\|5585\|5602\|5604\|5663\|5747\|5770\|6622\|6714\|6850\|6868\|7124\|8654\|8658\|8851\|9212\|10926\|23411\|50507 | ABL1\|ADORA1\|ADRA2A\|ADRA2C\|AKT1\|ALK\|AXL\|CCND1\|BRAF\|CCNB1\|CCND2\|CCND3\|CDK1\|CDK5\|CSF1R\|DRD2\|DRD4\|EGFR\|ELANE\|EPHB4\|ERN1\|F2\|F2R\|FGFR1\|FLT3\|MTOR\|GRM4\|GRM5\|HTR2A\|IGF1R\|IL2\|INSR\|ITGB3\|JAK2\|KDR\|KIT\|MAPT\|MET\|NEK2\|NTRK1\|P2RX7\|PIK3CA\|PIK3CB\|PIK3CG\|PLA2G1B\|PLK1\|PKN1\|MAPK10\|MAP2K1\|PSEN1\|PTK2\|PTPN1\|SNCA\|SRC\|SYK\|ADAM17\|TNF\|PDE5A\|TNKS\|CDK5R1\|AURKB\|DBF4\|SIRT1\|NOX4 | -34.21412914 |
|  |  | GO:0043269 | GO Biological Processes | regulation of ion transport | -37.21413004 | 7.762365256 | 19.78289995 | 25\|134\|135\|150\|207\|351\|383\|623\|706\|760\|766\|816\|823\|1020\|1080\|1128\|1131\|1268\|1520\|1612\|1813\|1815\|2147\|2149\|2170\|2260\|2475\|2539\|2645\|2859\|2902\|2904\|2915\|3350\|3356\|3690\|3760\|3762\|3763\|4318\|4638\|4842\|4889\|4985\|4988\|5024\|5027\|5028\|5142\|5144\|5243\|5294\|5319\|5663\|5664\|5724\|5743\|5777\|6335\|6532\|6622\|6754\|6850\|8564 | ABL1\|ADORA1\|ADORA2A\|ADRA2A\|AKT1\|APP\|ARG1\|BDKRB1\|TSPO\|CA2\|CA7\|CAMK2B\|CAPN1\|CDK5\|CFTR\|CHRM1\|CHRM3\|CNR1\|CTSS\|DAPK1\|DRD2\|DRD4\|F2\|F2R\|FABP3\|FGFR1\|MTOR\|G6PD\|GCK\|GPR35\|GRIN1\|GRIN2B\|GRM5\|HTR1A\|HTR2A\|ITGB3\|KCNJ3\|KCNJ5\|KCNJ6\|MMP9\|MYLK\|NOS1\|NPY5R\|OPRD1\|OPRM1\|P2RX3\|P2RX7\|P2RY1\|PDE4B\|PDE4D\|ABCB1\|PIK3CG\|PLA2G1B\|PSEN1\|PSEN2\|PTAFR\|PTGS2\|PTPN6\|SCN9A\|SLC6A4\|SNCA\|SSTR4\|SYK\|KMO | -33.80707922 |
|  | VMC-related targets | GO:1901699 | GO Biological Processes | cellular response to nitrogen compound | -19.36881939 | 14.44885511 | 16.81503385 | 25\|142\|207\|383\|836\|840\|1129\|1956\|2475\|4313\|4314\|4363\|4914\|5028\|5290\|5294\|5468\|5663\|5747\|6532\|6714\|7124 | ABL1\|PARP1\|AKT1\|ARG1\|CASP3\|CASP7\|CHRM2\|EGFR\|MTOR\|MMP2\|MMP3\|ABCC1\|NTRK1\|P2RY1\|PIK3CA\|PIK3CG\|PPARG\|PSEN1\|PTK2\|SLC6A4\|SRC\|TNF | -15.18763827 |
|  |  | hsa05200 | KEGG Pathway | Pathways in cancer | -19.33505178 | 15.97483696 | 17.367986 | 25\|207\|595\|836\|840\|1956\|2147\|2475\|3558\|3685\|3815\|4233\|4312\|4313\|4318\|4843\|4914\|5290\|5468\|5599\|5747 | ABL1\|AKT1\|CCND1\|CASP3\|CASP7\|EGFR\|F2\|MTOR\|IL2\|ITGAV\|KIT\|MET\|MMP1\|MMP2\|MMP9\|NOS2\|NTRK1\|PIK3CA\|PPARG\|MAPK8\|PTK2 | -15.18763827 |
|  |  | GO:0010035 | GO Biological Processes | response to inorganic substance | -18.0654571 | 15.40162852 | 16.59907081 | 25\|100\|142\|207\|383\|595\|706\|836\|1813\|1956\|2539\|3815\|4233\|4314\|4318\|4353\|4914\|5599\|6714\|7124 | ABL1\|ADA\|PARP1\|AKT1\|ARG1\|CCND1\|TSPO\|CASP3\|DRD2\|EGFR\|G6PD\|KIT\|MET\|MMP3\|MMP9\|MPO\|NTRK1\|MAPK8\|SRC\|TNF | -14.21907359 |
|  |  | hsa05205 | KEGG Pathway | Proteoglycans in cancer | -17.78392805 | 30.09676032 | 20.64511341 | 207\|595\|836\|1514\|1956\|2475\|3685\|3690\|4233\|4313\|4318\|5290\|5747\|6714\|7124 | AKT1\|CCND1\|CASP3\|CTSL\|EGFR\|MTOR\|ITGAV\|ITGB3\|MET\|MMP2\|MMP9\|PIK3CA\|PTK2\|SRC\|TNF | -14.03445455 |
|  |  | GO:0071417 | GO Biological Processes | cellular response to organonitrogen compound | -17.56064028 | 14.50766998 | 16.0514546 | 25\|142\|207\|383\|836\|840\|1956\|2475\|4313\|4363\|4914\|5028\|5290\|5294\|5468\|5663\|5747\|6532\|6714\|7124 | ABL1\|PARP1\|AKT1\|ARG1\|CASP3\|CASP7\|EGFR\|MTOR\|MMP2\|ABCC1\|NTRK1\|P2RY1\|PIK3CA\|PIK3CG\|PPARG\|PSEN1\|PTK2\|SLC6A4\|SRC\|TNF | -13.89034803 |
|  |  | GO:0009611 | GO Biological Processes | response to wounding | -17.49340305 | 12.97391419 | 15.44763767 | 135\|240\|383\|706\|836\|1813\|1956\|2147\|2475\|3690\|3932\|4914\|5028\|5290\|5294\|5340\|5465\|5747\|6714\|6868\|7124 | ADORA2A\|ALOX5\|ARG1\|TSPO\|CASP3\|DRD2\|EGFR\|F2\|MTOR\|ITGB3\|LCK\|NTRK1\|P2RY1\|PIK3CA\|PIK3CG\|PLG\|PPARA\|PTK2\|SRC\|ADAM17\|TNF | -13.89005759 |
|  |  | GO:0033674 | GO Biological Processes | positive regulation of kinase activity | -17.39471134 | 14.2245935 | 15.87414488 | 25\|207\|595\|1813\|1956\|1991\|2147\|2475\|3558\|3690\|3815\|4233\|4914\|5290\|5294\|5663\|5747\|6714\|6868\|7124 | ABL1\|AKT1\|CCND1\|DRD2\|EGFR\|ELANE\|F2\|MTOR\|IL2\|ITGB3\|KIT\|MET\|NTRK1\|PIK3CA\|PIK3CG\|PSEN1\|PTK2\|SRC\|ADAM17\|TNF | -13.84935783 |
|  |  | GO:0008015 | GO Biological Processes | blood circulation | -17.00547935 | 15.16554516 | 16.03109856 | 100\|135\|207\|1129\|1215\|1813\|1956\|2475\|3274\|3558\|3762\|4843\|5028\|5290\|5294\|5465\|5468\|6532\|6714 | ADA\|ADORA2A\|AKT1\|CHRM2\|CMA1\|DRD2\|EGFR\|MTOR\|HRH2\|IL2\|KCNJ5\|NOS2\|P2RY1\|PIK3CA\|PIK3CG\|PPARA\|PPARG\|SLC6A4\|SRC | -13.54112638 |
|  |  | GO:0006979 | GO Biological Processes | response to oxidative stress | -16.98956988 | 17.07876898 | 16.66309736 | 25\|100\|142\|207\|240\|383\|836\|1956\|2539\|4233\|4313\|4314\|4318\|4353\|5599\|5663\|6714\|7124 | ABL1\|ADA\|PARP1\|AKT1\|ALOX5\|ARG1\|CASP3\|EGFR\|G6PD\|MET\|MMP2\|MMP3\|MMP9\|MPO\|MAPK8\|PSEN1\|SRC\|TNF | -13.54112638 |
|  |  | GO:0003013 | GO Biological Processes | circulatory system process | -16.84618285 | 14.86711762 | 15.85327022 | 100\|135\|207\|1129\|1215\|1813\|1956\|2475\|3274\|3558\|3762\|4843\|5028\|5290\|5294\|5465\|5468\|6532\|6714 | ADA\|ADORA2A\|AKT1\|CHRM2\|CMA1\|DRD2\|EGFR\|MTOR\|HRH2\|IL2\|KCNJ5\|NOS2\|P2RY1\|PIK3CA\|PIK3CG\|PPARA\|PPARG\|SLC6A4\|SRC | -13.43913204 |
| JXT | whole targets | GO:0004672 | GO Molecular Functions | protein kinase activity | -78.37336913 | 10.98877187 | 31.61303054 | 25|207|238|558|595|640|660|673|695|816|891|896|983|1017|1018|1019|1020|1021|1111|1195|1432|1445|1457|1612|1956|1969|2041|2042|2043|2044|2048|2049|2081|2268|2321|2322|2475|2534|2870|2932|3480|3551|3643|3702|3716|3717|3718|3791|3932|3984|4067|4233|4750|4751|5156|5159|5163|5170|5290|5292|5294|5347|5566|5578|5579|5583|5585|5590|5594|5595|5599|5601|5602|5604|5610|5747|5753|5894|5979|6093|6198|6714|6790|6850|7150|7294|7297|7301|7465|7525|8317|8851|9149|9212|9261|9475|9891|10769|10783|11040|11200|23031|23476|79934|285220|415116 | ABL1|AKT1|ALK|AXL|CCND1|BLK|BMX|BRAF|BTK|CAMK2B|CCNB1|CCND3|CDK1|CDK2|CDK3|CDK4|CDK5|CDK6|CHEK1|CLK1|MAPK14|CSK|CSNK2A1|DAPK1|EGFR|EPHA2|EPHA1|EPHA3|EPHA4|EPHA5|EPHB2|EPHB3|ERN1|FGR|FLT1|FLT3|MTOR|FYN|GRK6|GSK3B|IGF1R|IKBKB|INSR|ITK|JAK1|JAK2|JAK3|KDR|LCK|LIMK1|LYN|MET|NEK1|NEK2|PDGFRA|PDGFRB|PDK1|PDPK1|PIK3CA|PIM1|PIK3CG|PLK1|PRKACA|PRKCA|PRKCB|PRKCH|PKN1|PRKCZ|MAPK1|MAPK3|MAPK8|MAPK9|MAPK10|MAP2K1|EIF2AK2|PTK2|PTK6|RAF1|RET|ROCK1|RPS6KB1|SRC|AURKA|SYK|TOP1|TXK|TYK2|TYRO3|WEE1|YES1|CDC7|CDK5R1|DYRK1B|AURKB|MAPKAPK2|ROCK2|NUAK1|PLK2|NEK6|PIM2|CHEK2|MAST3|BRD4|COQ8B|EPHA6|PIM3 | -73.92492563 |
|  |  | GO:0016773 | GO Molecular Functions | phosphotransferase activity, alcohol group as acceptor | -72.76934297 | 9.458497579 | 29.18171932 | 25\|207\|238\|558\|595\|640\|660\|673\|695\|816\|891\|896\|983\|1017\|1018\|1019\|1020\|1021\|1111\|1195\|1432\|1445\|1457\|1612\|1956\|1969\|2041\|2042\|2043\|2044\|2048\|2049\|2081\|2268\|2321\|2322\|2475\|2534\|2870\|2932\|3480\|3551\|3643\|3702\|3716\|3717\|3718\|3791\|3932\|3984\|4067\|4233\|4750\|4751\|5156\|5159\|5163\|5170\|5209\|5290\|5292\|5293\|5294\|5347\|5566\|5578\|5579\|5583\|5585\|5590\|5594\|5595\|5599\|5601\|5602\|5604\|5610\|5747\|5753\|5894\|5979\|6093\|6198\|6714\|6790\|6850\|7150\|7294\|7297\|7301\|7465\|7525\|8317\|8851\|9149\|9212\|9261\|9475\|9891\|10769\|10783\|11040\|11200\|23031\|23476\|79934\|285220\|415116 | ABL1\|AKT1\|ALK\|AXL\|CCND1\|BLK\|BMX\|BRAF\|BTK\|CAMK2B\|CCNB1\|CCND3\|CDK1\|CDK2\|CDK3\|CDK4\|CDK5\|CDK6\|CHEK1\|CLK1\|MAPK14\|CSK\|CSNK2A1\|DAPK1\|EGFR\|EPHA2\|EPHA1\|EPHA3\|EPHA4\|EPHA5\|EPHB2\|EPHB3\|ERN1\|FGR\|FLT1\|FLT3\|MTOR\|FYN\|GRK6\|GSK3B\|IGF1R\|IKBKB\|INSR\|ITK\|JAK1\|JAK2\|JAK3\|KDR\|LCK\|LIMK1\|LYN\|MET\|NEK1\|NEK2\|PDGFRA\|PDGFRB\|PDK1\|PDPK1\|PFKFB3\|PIK3CA\|PIM1\|PIK3CD\|PIK3CG\|PLK1\|PRKACA\|PRKCA\|PRKCB\|PRKCH\|PKN1\|PRKCZ\|MAPK1\|MAPK3\|MAPK8\|MAPK9\|MAPK10\|MAP2K1\|EIF2AK2\|PTK2\|PTK6\|RAF1\|RET\|ROCK1\|RPS6KB1\|SRC\|AURKA\|SYK\|TOP1\|TXK\|TYK2\|TYRO3\|WEE1\|YES1\|CDC7\|CDK5R1\|DYRK1B\|AURKB\|MAPKAPK2\|ROCK2\|NUAK1\|PLK2\|NEK6\|PIM2\|CHEK2\|MAST3\|BRD4\|COQ8B\|EPHA6\|PIM3 | -68.62192947 |
|  |  | GO:0016301 | GO Molecular Functions | kinase activity | -69.56355793 | 8.704899981 | 27.87023329 | 25\|207\|238\|558\|595\|640\|660\|673\|695\|816\|891\|896\|898\|983\|1017\|1018\|1019\|1020\|1021\|1111\|1195\|1432\|1445\|1457\|1612\|1956\|1969\|2041\|2042\|2043\|2044\|2048\|2049\|2081\|2268\|2321\|2322\|2475\|2534\|2870\|2932\|3480\|3551\|3643\|3702\|3716\|3717\|3718\|3791\|3932\|3984\|4067\|4233\|4750\|4751\|5156\|5159\|5163\|5170\|5209\|5290\|5292\|5293\|5294\|5347\|5566\|5578\|5579\|5583\|5585\|5590\|5594\|5595\|5599\|5601\|5602\|5604\|5610\|5747\|5753\|5894\|5979\|6093\|6198\|6714\|6790\|6850\|7150\|7294\|7297\|7301\|7465\|7525\|8317\|8851\|9149\|9212\|9261\|9475\|9891\|10769\|10783\|11040\|11200\|23031\|23476\|79934\|285220\|415116 | ABL1\|AKT1\|ALK\|AXL\|CCND1\|BLK\|BMX\|BRAF\|BTK\|CAMK2B\|CCNB1\|CCND3\|CCNE1\|CDK1\|CDK2\|CDK3\|CDK4\|CDK5\|CDK6\|CHEK1\|CLK1\|MAPK14\|CSK\|CSNK2A1\|DAPK1\|EGFR\|EPHA2\|EPHA1\|EPHA3\|EPHA4\|EPHA5\|EPHB2\|EPHB3\|ERN1\|FGR\|FLT1\|FLT3\|MTOR\|FYN\|GRK6\|GSK3B\|IGF1R\|IKBKB\|INSR\|ITK\|JAK1\|JAK2\|JAK3\|KDR\|LCK\|LIMK1\|LYN\|MET\|NEK1\|NEK2\|PDGFRA\|PDGFRB\|PDK1\|PDPK1\|PFKFB3\|PIK3CA\|PIM1\|PIK3CD\|PIK3CG\|PLK1\|PRKACA\|PRKCA\|PRKCB\|PRKCH\|PKN1\|PRKCZ\|MAPK1\|MAPK3\|MAPK8\|MAPK9\|MAPK10\|MAP2K1\|EIF2AK2\|PTK2\|PTK6\|RAF1\|RET\|ROCK1\|RPS6KB1\|SRC\|AURKA\|SYK\|TOP1\|TXK\|TYK2\|TYRO3\|WEE1\|YES1\|CDC7\|CDK5R1\|DYRK1B\|AURKB\|MAPKAPK2\|ROCK2\|NUAK1\|PLK2\|NEK6\|PIM2\|CHEK2\|MAST3\|BRD4\|COQ8B\|EPHA6\|PIM3 | -65.59223568 |
|  |  | GO:1901699 | GO Biological Processes | cellular response to nitrogen compound | -63.74132109 | 8.891603142 | 26.87661208 | 25\|111\|142\|196\|207\|231\|351\|383\|598\|760\|836\|840\|890\|896\|1017\|1019\|1020\|1080\|1128\|1129\|1131\|1132\|1133\|1136\|1137\|1139\|1141\|1445\|1584\|1585\|1956\|2043\|2101\|2475\|2534\|2554\|2562\|2566\|2741\|2798\|2915\|2932\|2946\|2950\|3066\|3269\|3383\|3480\|3643\|3676\|3717\|3718\|4067\|4193\|4313\|4314\|4363\|5024\|5027\|5138\|5142\|5144\|5156\|5170\|5290\|5294\|5295\|5319\|5468\|5566\|5579\|5590\|5594\|5595\|5663\|5743\|5747\|5770\|5771\|5781\|6093\|6198\|6532\|6622\|6714\|7099\|7124\|7430\|8850\|8851\|9475\|9734\|9971\|10014\|10135\|11255\|23028\|23621\|50507 | ABL1\|ADCY5\|PARP1\|AHR\|AKT1\|AKR1B1\|APP\|ARG1\|BCL2L1\|CA2\|CASP3\|CASP7\|CCNA2\|CCND3\|CDK2\|CDK4\|CDK5\|CFTR\|CHRM1\|CHRM2\|CHRM3\|CHRM4\|CHRM5\|CHRNA3\|CHRNA4\|CHRNA7\|CHRNB2\|CSK\|CYP11B1\|CYP11B2\|EGFR\|EPHA4\|ESRRA\|MTOR\|FYN\|GABRA1\|GABRB3\|GABRG2\|GLRA1\|GNRHR\|GRM5\|GSK3B\|GSTM2\|GSTP1\|HDAC2\|HRH1\|ICAM1\|IGF1R\|INSR\|ITGA4\|JAK2\|JAK3\|LYN\|MDM2\|MMP2\|MMP3\|ABCC1\|P2RX3\|P2RX7\|PDE2A\|PDE4B\|PDE4D\|PDGFRA\|PDPK1\|PIK3CA\|PIK3CG\|PIK3R1\|PLA2G1B\|PPARG\|PRKACA\|PRKCB\|PRKCZ\|MAPK1\|MAPK3\|PSEN1\|PTGS2\|PTK2\|PTPN1\|PTPN2\|PTPN11\|ROCK1\|RPS6KB1\|SLC6A4\|SNCA\|SRC\|TLR4\|TNF\|EZR\|KAT2B\|CDK5R1\|ROCK2\|HDAC9\|NR1H4\|HDAC5\|NAMPT\|HRH3\|KDM1A\|BACE1\|NOX4 | -59.9918476 |
|  |  | GO:0033674 | GO Biological Processes | positive regulation of kinase activity | -55.99658192 | 8.65633382 | 25.0337392 | 25\|111\|134\|136\|150\|151\|152\|207\|238\|558\|595\|673\|891\|894\|896\|983\|994\|1020\|1136\|1139\|1432\|1445\|1813\|1815\|1956\|1969\|1991\|2041\|2042\|2043\|2044\|2048\|2049\|2081\|2147\|2268\|2321\|2322\|2357\|2475\|2534\|2914\|2915\|3320\|3326\|3356\|3357\|3480\|3558\|3643\|3717\|3791\|4067\|4233\|4282\|5027\|5156\|5159\|5170\|5290\|5294\|5319\|5566\|5585\|5590\|5594\|5595\|5602\|5604\|5610\|5663\|5747\|5770\|5781\|5894\|5979\|6622\|6714\|6850\|6868\|7099\|7124\|7301\|8851\|9261\|10892\|50507\|54106\|285220 | ABL1\|ADCY5\|ADORA1\|ADORA2B\|ADRA2A\|ADRA2B\|ADRA2C\|AKT1\|ALK\|AXL\|CCND1\|BRAF\|CCNB1\|CCND2\|CCND3\|CDK1\|CDC25B\|CDK5\|CHRNA3\|CHRNA7\|MAPK14\|CSK\|DRD2\|DRD4\|EGFR\|EPHA2\|ELANE\|EPHA1\|EPHA3\|EPHA4\|EPHA5\|EPHB2\|EPHB3\|ERN1\|F2\|FGR\|FLT1\|FLT3\|FPR1\|MTOR\|FYN\|GRM4\|GRM5\|HSP90AA1\|HSP90AB1\|HTR2A\|HTR2B\|IGF1R\|IL2\|INSR\|JAK2\|KDR\|LYN\|MET\|MIF\|P2RX7\|PDGFRA\|PDGFRB\|PDPK1\|PIK3CA\|PIK3CG\|PLA2G1B\|PRKACA\|PKN1\|PRKCZ\|MAPK1\|MAPK3\|MAPK10\|MAP2K1\|EIF2AK2\|PSEN1\|PTK2\|PTPN1\|PTPN11\|RAF1\|RET\|SNCA\|SRC\|SYK\|ADAM17\|TLR4\|TNF\|TYRO3\|CDK5R1\|MAPKAPK2\|MALT1\|NOX4\|TLR9\|EPHA6 | -52.32628967 |
|  |  | hsa04080 | KEGG Pathway | Neuroactive ligand-receptor interaction | -55.54422001 | 11.80158235 | 27.27225546 | 134\|135\|136\|140\|147\|150\|151\|152\|554\|623\|706\|1128\|1129\|1131\|1132\|1133\|1136\|1137\|1139\|1141\|1143\|1268\|1269\|1813\|1814\|1815\|1901\|1903\|2147\|2357\|2554\|2555\|2556\|2558\|2562\|2566\|2741\|2798\|2859\|2890\|2908\|2914\|2915\|3061\|3062\|3269\|3350\|3356\|3357\|3358\|3362\|3363\|4543\|4544\|4829\|4886\|4889\|4986\|5021\|5024\|5027\|5340\|5731\|5732\|5733\|5737\|5739\|6870\|7067\|7068\|10203\|11255\|134864 | ADORA1\|ADORA2A\|ADORA2B\|ADORA3\|ADRA1B\|ADRA2A\|ADRA2B\|ADRA2C\|AVPR2\|BDKRB1\|TSPO\|CHRM1\|CHRM2\|CHRM3\|CHRM4\|CHRM5\|CHRNA3\|CHRNA4\|CHRNA7\|CHRNB2\|CHRNB4\|CNR1\|CNR2\|DRD2\|DRD3\|DRD4\|S1PR1\|S1PR3\|F2\|FPR1\|GABRA1\|GABRA2\|GABRA3\|GABRA5\|GABRB3\|GABRG2\|GLRA1\|GNRHR\|GPR35\|GRIA1\|NR3C1\|GRM4\|GRM5\|HCRTR1\|HCRTR2\|HRH1\|HTR1A\|HTR2A\|HTR2B\|HTR2C\|HTR6\|HTR7\|MTNR1A\|MTNR1B\|NMBR\|NPY1R\|NPY5R\|OPRK1\|OXTR\|P2RX3\|P2RX7\|PLG\|PTGER1\|PTGER2\|PTGER3\|PTGFR\|PTGIR\|TACR3\|THRA\|THRB\|CALCRL\|HRH3\|TAAR1 | -51.94087455 |
|  |  | GO:0051347 | GO Biological Processes | positive regulation of transferase activity | -55.47746858 | 7.99268715 | 24.35899686 | 25\|111\|134\|136\|150\|151\|152\|207\|238\|558\|595\|673\|891\|894\|896\|983\|994\|1020\|1136\|1139\|1432\|1445\|1813\|1815\|1956\|1969\|1991\|2041\|2042\|2043\|2044\|2048\|2049\|2081\|2147\|2268\|2321\|2322\|2357\|2475\|2534\|2914\|2915\|3320\|3326\|3356\|3357\|3480\|3558\|3643\|3717\|3791\|4067\|4233\|4282\|4751\|5027\|5156\|5159\|5170\|5290\|5294\|5319\|5347\|5566\|5585\|5590\|5594\|5595\|5602\|5604\|5610\|5663\|5747\|5770\|5781\|5894\|5979\|6622\|6714\|6850\|6868\|7099\|7124\|7301\|8658\|8851\|9212\|9261\|10892\|50507\|54106\|285220 | ABL1\|ADCY5\|ADORA1\|ADORA2B\|ADRA2A\|ADRA2B\|ADRA2C\|AKT1\|ALK\|AXL\|CCND1\|BRAF\|CCNB1\|CCND2\|CCND3\|CDK1\|CDC25B\|CDK5\|CHRNA3\|CHRNA7\|MAPK14\|CSK\|DRD2\|DRD4\|EGFR\|EPHA2\|ELANE\|EPHA1\|EPHA3\|EPHA4\|EPHA5\|EPHB2\|EPHB3\|ERN1\|F2\|FGR\|FLT1\|FLT3\|FPR1\|MTOR\|FYN\|GRM4\|GRM5\|HSP90AA1\|HSP90AB1\|HTR2A\|HTR2B\|IGF1R\|IL2\|INSR\|JAK2\|KDR\|LYN\|MET\|MIF\|NEK2\|P2RX7\|PDGFRA\|PDGFRB\|PDPK1\|PIK3CA\|PIK3CG\|PLA2G1B\|PLK1\|PRKACA\|PKN1\|PRKCZ\|MAPK1\|MAPK3\|MAPK10\|MAP2K1\|EIF2AK2\|PSEN1\|PTK2\|PTPN1\|PTPN11\|RAF1\|RET\|SNCA\|SRC\|SYK\|ADAM17\|TLR4\|TNF\|TYRO3\|TNKS\|CDK5R1\|AURKB\|MAPKAPK2\|MALT1\|NOX4\|TLR9\|EPHA6 | -51.93211507 |
|  |  | GO:0099537 | GO Biological Processes | trans-synaptic signaling | -54.53436026 | 7.681320349 | 23.88444065 | 25\|43\|134\|135\|136\|207\|351\|590\|760\|766\|816\|952\|1020\|1128\|1129\|1131\|1132\|1133\|1136\|1137\|1139\|1141\|1143\|1268\|1269\|1759\|1813\|1814\|1815\|1956\|2030\|2043\|2048\|2171\|2475\|2534\|2554\|2555\|2556\|2558\|2562\|2566\|2741\|2752\|2890\|2914\|2915\|2932\|3061\|3062\|3269\|3350\|3356\|3357\|3358\|3359\|3362\|3363\|3717\|3777\|3785\|3786\|4544\|4842\|4889\|4986\|5021\|5024\|5027\|5327\|5340\|5566\|5579\|5590\|5594\|5663\|5743\|5802\|6530\|6531\|6532\|6536\|6571\|6622\|6714\|7124\|8913\|10769\|11255\|23385\|23621\|27163\|54583\|60482 | ABL1\|ACHE\|ADORA1\|ADORA2A\|ADORA2B\|AKT1\|APP\|BCHE\|CA2\|CA7\|CAMK2B\|CD38\|CDK5\|CHRM1\|CHRM2\|CHRM3\|CHRM4\|CHRM5\|CHRNA3\|CHRNA4\|CHRNA7\|CHRNB2\|CHRNB4\|CNR1\|CNR2\|DNM1\|DRD2\|DRD3\|DRD4\|EGFR\|SLC29A1\|EPHA4\|EPHB2\|FABP5\|MTOR\|FYN\|GABRA1\|GABRA2\|GABRA3\|GABRA5\|GABRB3\|GABRG2\|GLRA1\|GLUL\|GRIA1\|GRM4\|GRM5\|GSK3B\|HCRTR1\|HCRTR2\|HRH1\|HTR1A\|HTR2A\|HTR2B\|HTR2C\|HTR3A\|HTR6\|HTR7\|JAK2\|KCNK3\|KCNQ2\|KCNQ3\|MTNR1B\|NOS1\|NPY5R\|OPRK1\|OXTR\|P2RX3\|P2RX7\|PLAT\|PLG\|PRKACA\|PRKCB\|PRKCZ\|MAPK1\|PSEN1\|PTGS2\|PTPRS\|SLC6A2\|SLC6A3\|SLC6A4\|SLC6A9\|SLC18A2\|SNCA\|SRC\|TNF\|CACNA1G\|PLK2\|HRH3\|NCSTN\|BACE1\|NAAA\|EGLN1\|SLC5A7 | -51.04015927 |
|  |  | GO:0099536 | GO Biological Processes | synaptic signaling | -54.05440015 | 7.58802496 | 23.69917772 | 25\|43\|134\|135\|136\|207\|351\|590\|760\|766\|816\|952\|1020\|1128\|1129\|1131\|1132\|1133\|1136\|1137\|1139\|1141\|1143\|1268\|1269\|1759\|1813\|1814\|1815\|1956\|2030\|2043\|2048\|2171\|2475\|2534\|2554\|2555\|2556\|2558\|2562\|2566\|2741\|2752\|2890\|2914\|2915\|2932\|3061\|3062\|3269\|3350\|3356\|3357\|3358\|3359\|3362\|3363\|3717\|3777\|3785\|3786\|4544\|4842\|4889\|4986\|5021\|5024\|5027\|5327\|5340\|5566\|5579\|5590\|5594\|5663\|5743\|5802\|6530\|6531\|6532\|6536\|6571\|6622\|6714\|7124\|8913\|10769\|11255\|23385\|23621\|27163\|54583\|60482 | ABL1\|ACHE\|ADORA1\|ADORA2A\|ADORA2B\|AKT1\|APP\|BCHE\|CA2\|CA7\|CAMK2B\|CD38\|CDK5\|CHRM1\|CHRM2\|CHRM3\|CHRM4\|CHRM5\|CHRNA3\|CHRNA4\|CHRNA7\|CHRNB2\|CHRNB4\|CNR1\|CNR2\|DNM1\|DRD2\|DRD3\|DRD4\|EGFR\|SLC29A1\|EPHA4\|EPHB2\|FABP5\|MTOR\|FYN\|GABRA1\|GABRA2\|GABRA3\|GABRA5\|GABRB3\|GABRG2\|GLRA1\|GLUL\|GRIA1\|GRM4\|GRM5\|GSK3B\|HCRTR1\|HCRTR2\|HRH1\|HTR1A\|HTR2A\|HTR2B\|HTR2C\|HTR3A\|HTR6\|HTR7\|JAK2\|KCNK3\|KCNQ2\|KCNQ3\|MTNR1B\|NOS1\|NPY5R\|OPRK1\|OXTR\|P2RX3\|P2RX7\|PLAT\|PLG\|PRKACA\|PRKCB\|PRKCZ\|MAPK1\|PSEN1\|PTGS2\|PTPRS\|SLC6A2\|SLC6A3\|SLC6A4\|SLC6A9\|SLC18A2\|SNCA\|SRC\|TNF\|CACNA1G\|PLK2\|HRH3\|NCSTN\|BACE1\|NAAA\|EGLN1\|SLC5A7 | -50.60595665 |
|  |  | GO:0071417 | GO Biological Processes | cellular response to organonitrogen compound | -53.49837847 | 8.531005939 | 24.37719874 | 25\|111\|142\|196\|207\|231\|351\|383\|598\|760\|836\|840\|890\|896\|1019\|1020\|1080\|1445\|1584\|1585\|1956\|2043\|2101\|2475\|2534\|2554\|2562\|2566\|2741\|2798\|2915\|2932\|2946\|2950\|3066\|3269\|3383\|3480\|3643\|3676\|3717\|3718\|4067\|4193\|4313\|4363\|5024\|5027\|5138\|5142\|5144\|5156\|5170\|5290\|5294\|5295\|5319\|5468\|5566\|5579\|5590\|5594\|5595\|5663\|5743\|5747\|5770\|5771\|5781\|6093\|6198\|6532\|6622\|6714\|7099\|7124\|7430\|8850\|9475\|9734\|9971\|10014\|10135\|23028\|23621\|50507 | ABL1\|ADCY5\|PARP1\|AHR\|AKT1\|AKR1B1\|APP\|ARG1\|BCL2L1\|CA2\|CASP3\|CASP7\|CCNA2\|CCND3\|CDK4\|CDK5\|CFTR\|CSK\|CYP11B1\|CYP11B2\|EGFR\|EPHA4\|ESRRA\|MTOR\|FYN\|GABRA1\|GABRB3\|GABRG2\|GLRA1\|GNRHR\|GRM5\|GSK3B\|GSTM2\|GSTP1\|HDAC2\|HRH1\|ICAM1\|IGF1R\|INSR\|ITGA4\|JAK2\|JAK3\|LYN\|MDM2\|MMP2\|ABCC1\|P2RX3\|P2RX7\|PDE2A\|PDE4B\|PDE4D\|PDGFRA\|PDPK1\|PIK3CA\|PIK3CG\|PIK3R1\|PLA2G1B\|PPARG\|PRKACA\|PRKCB\|PRKCZ\|MAPK1\|MAPK3\|PSEN1\|PTGS2\|PTK2\|PTPN1\|PTPN2\|PTPN11\|ROCK1\|RPS6KB1\|SLC6A4\|SNCA\|SRC\|TLR4\|TNF\|EZR\|KAT2B\|ROCK2\|HDAC9\|NR1H4\|HDAC5\|NAMPT\|KDM1A\|BACE1\|NOX4 | -50.09132766 |
|  | VMC-related targets | GO:0033674 | GO Biological Processes | positive regulation of kinase activity | -29.88992611 | 15.64705285 | 21.54531357 | 25\|111\|207\|595\|1432\|1813\|1956\|1991\|2044\|2147\|2321\|2475\|2534\|3320\|3558\|4233\|4282\|5290\|5294\|5566\|5594\|5595\|5610\|5663\|5747\|5781\|5894\|5979\|6714\|6868\|7099\|7124\|54106 | ABL1\|ADCY5\|AKT1\|CCND1\|MAPK14\|DRD2\|EGFR\|ELANE\|EPHA5\|F2\|FLT1\|MTOR\|FYN\|HSP90AA1\|IL2\|MET\|MIF\|PIK3CA\|PIK3CG\|PRKACA\|MAPK1\|MAPK3\|EIF2AK2\|PSEN1\|PTK2\|PTPN11\|RAF1\|RET\|SRC\|ADAM17\|TLR4\|TNF\|TLR9 | -25.44148261 |
|  |  | hsa05205 | KEGG Pathway | Proteoglycans in cancer | -28.96546677 | 32.10321101 | 27.04409329 | 207\|595\|836\|1432\|1514\|1956\|2194\|2475\|3569\|4233\|4313\|4318\|5290\|5566\|5578\|5594\|5595\|5747\|5781\|5894\|6198\|6714\|7099\|7124 | AKT1\|CCND1\|CASP3\|MAPK14\|CTSL\|EGFR\|FASN\|MTOR\|IL6\|MET\|MMP2\|MMP9\|PIK3CA\|PRKACA\|PRKCA\|MAPK1\|MAPK3\|PTK2\|PTPN11\|RAF1\|RPS6KB1\|SRC\|TLR4\|TNF | -24.81805326 |
|  |  | GO:0051347 | GO Biological Processes | positive regulation of transferase activity | -28.15042701 | 13.82605963 | 20.10047235 | 25\|111\|207\|595\|1432\|1813\|1956\|1991\|2044\|2147\|2321\|2475\|2534\|3320\|3558\|4233\|4282\|5290\|5294\|5566\|5594\|5595\|5610\|5663\|5747\|5781\|5894\|5979\|6714\|6868\|7099\|7124\|54106 | ABL1\|ADCY5\|AKT1\|CCND1\|MAPK14\|DRD2\|EGFR\|ELANE\|EPHA5\|F2\|FLT1\|MTOR\|FYN\|HSP90AA1\|IL2\|MET\|MIF\|PIK3CA\|PIK3CG\|PRKACA\|MAPK1\|MAPK3\|EIF2AK2\|PSEN1\|PTK2\|PTPN11\|RAF1\|RET\|SRC\|ADAM17\|TLR4\|TNF\|TLR9 | -24.24929463 |
|  |  | hsa05200 | KEGG Pathway | Pathways in cancer | -28.0320276 | 15.72126812 | 20.92315357 | 25\|111\|207\|595\|596\|836\|840\|1956\|2147\|2194\|2475\|3320\|3551\|3558\|3569\|4233\|4312\|4313\|4318\|4843\|5290\|5468\|5566\|5578\|5594\|5595\|5599\|5747\|5894\|5979\|6198 | ABL1\|ADCY5\|AKT1\|CCND1\|BCL2\|CASP3\|CASP7\|EGFR\|F2\|FASN\|MTOR\|HSP90AA1\|IKBKB\|IL2\|IL6\|MET\|MMP1\|MMP2\|MMP9\|NOS2\|PIK3CA\|PPARG\|PRKACA\|PRKCA\|MAPK1\|MAPK3\|MAPK8\|PTK2\|RAF1\|RET\|RPS6KB1 | -24.24929463 |
|  |  | GO:0050900 | GO Biological Processes | leukocyte migration | -27.99876812 | 16.8882722 | 21.41108134 | 207\|240\|1432\|1901\|1991\|2147\|2321\|2534\|2833\|3269\|3383\|3569\|3676\|3683\|3689\|3932\|4282\|4312\|5290\|5294\|5340\|5594\|5595\|5747\|5781\|5979\|6714\|6868\|7124\|7941 | AKT1\|ALOX5\|MAPK14\|S1PR1\|ELANE\|F2\|FLT1\|FYN\|CXCR3\|HRH1\|ICAM1\|IL6\|ITGA4\|ITGAL\|ITGB2\|LCK\|MIF\|MMP1\|PIK3CA\|PIK3CG\|PLG\|MAPK1\|MAPK3\|PTK2\|PTPN11\|RET\|SRC\|ADAM17\|TNF\|PLA2G7 | -24.24929463 |
|  |  | GO:0009611 | GO Biological Processes | response to wounding | -26.59300845 | 13.17984934 | 19.25583746 | 135\|240\|383\|596\|640\|706\|836\|1813\|1956\|2147\|2152\|2475\|2534\|3569\|3932\|4846\|5290\|5294\|5327\|5340\|5465\|5566\|5578\|5594\|5595\|5747\|5781\|5894\|6714\|6868\|7099\|7124 | ADORA2A\|ALOX5\|ARG1\|BCL2\|BLK\|TSPO\|CASP3\|DRD2\|EGFR\|F2\|F3\|MTOR\|FYN\|IL6\|LCK\|NOS3\|PIK3CA\|PIK3CG\|PLAT\|PLG\|PPARA\|PRKACA\|PRKCA\|MAPK1\|MAPK3\|PTK2\|PTPN11\|RAF1\|SRC\|ADAM17\|TLR4\|TNF | -22.9227162 |
|  |  | GO:1901699 | GO Biological Processes | cellular response to nitrogen compound | -26.09522656 | 13.57316692 | 19.26423089 | 25\|111\|142\|207\|383\|836\|840\|1129\|1956\|2475\|2534\|3269\|3383\|3676\|4313\|4314\|4363\|5290\|5294\|5468\|5566\|5594\|5595\|5663\|5747\|5781\|6198\|6532\|6714\|7099\|7124 | ABL1\|ADCY5\|PARP1\|AKT1\|ARG1\|CASP3\|CASP7\|CHRM2\|EGFR\|MTOR\|FYN\|HRH1\|ICAM1\|ITGA4\|MMP2\|MMP3\|ABCC1\|PIK3CA\|PIK3CG\|PPARG\|PRKACA\|MAPK1\|MAPK3\|PSEN1\|PTK2\|PTPN11\|RPS6KB1\|SLC6A4\|SRC\|TLR4\|TNF | -22.64905155 |
|  |  | GO:0045860 | GO Biological Processes | positive regulation of protein kinase activity | -26.05961485 | 15.63127696 | 20.15857191 | 25\|111\|207\|595\|1432\|1813\|1956\|1991\|2321\|2475\|2534\|3320\|4282\|5290\|5294\|5566\|5594\|5595\|5610\|5663\|5747\|5781\|5894\|5979\|6714\|6868\|7099\|7124\|54106 | ABL1\|ADCY5\|AKT1\|CCND1\|MAPK14\|DRD2\|EGFR\|ELANE\|FLT1\|MTOR\|FYN\|HSP90AA1\|MIF\|PIK3CA\|PIK3CG\|PRKACA\|MAPK1\|MAPK3\|EIF2AK2\|PSEN1\|PTK2\|PTPN11\|RAF1\|RET\|SRC\|ADAM17\|TLR4\|TNF\|TLR9 | -22.64905155 |
|  |  | hsa01522 | KEGG Pathway | Endocrine resistance | -26.05610236 | 54.67578125 | 30.90304395 | 111\|207\|595\|596\|1432\|1956\|2475\|4313\|4318\|5290\|5566\|5594\|5595\|5599\|5747\|5894\|6198\|6714 | ADCY5\|AKT1\|CCND1\|BCL2\|MAPK14\|EGFR\|MTOR\|MMP2\|MMP9\|PIK3CA\|PRKACA\|MAPK1\|MAPK3\|MAPK8\|PTK2\|RAF1\|RPS6KB1\|SRC | -22.64905155 |
|  |  | GO:0071417 | GO Biological Processes | cellular response to organonitrogen compound | -24.72199539 | 14.02408098 | 18.96599378 | 25\|111\|142\|207\|383\|836\|840\|1956\|2475\|2534\|3269\|3383\|3676\|4313\|4363\|5290\|5294\|5468\|5566\|5594\|5595\|5663\|5747\|5781\|6198\|6532\|6714\|7099\|7124 | ABL1\|ADCY5\|PARP1\|AKT1\|ARG1\|CASP3\|CASP7\|EGFR\|MTOR\|FYN\|HRH1\|ICAM1\|ITGA4\|MMP2\|ABCC1\|PIK3CA\|PIK3CG\|PPARG\|PRKACA\|MAPK1\|MAPK3\|PSEN1\|PTK2\|PTPN11\|RPS6KB1\|SLC6A4\|SRC\|TLR4\|TNF | -21.35273313 |

| **Table S9. Target details in PPI network** | | | | | | |
| --- | --- | --- | --- | --- | --- | --- |
| **Herb** | **Target type** | **Symbol** | **MCODE score** | **MCODE cluster ID** | **MCODE type** | **DEGREE** |
| HQ | whole-targets | HSP90AA1 | 2.75 | 4 | Clustered | 132 |
|  |  | HSP90AB1 | 6.666666667 | 1 | Clustered | 126 |
|  |  | APP | 6.666666667 | 1 | Clustered | 110 |
|  |  | EGFR | 6.666666667 | 1 | Clustered | 104 |
|  |  | SRC | 6.666666667 | 1 | Clustered | 102 |
|  |  | MAPK1 | 7.605263158 | 2 | Clustered | 101 |
|  |  | CDK2 | 7.605263158 | 2 | Seed | 99 |
|  |  | JUN | 7.605263158 | 2 | Clustered | 93 |
|  |  | PRKCA | 7.605263158 | 2 | Clustered | 88 |
|  |  | AKT1 | 7.605263158 | 2 | Clustered | 85 |
|  |  | GSK3B | 7.605263158 | 2 | Clustered | 85 |
|  |  | CDK1 | 2.931034483 | 3 | Clustered | 82 |
|  |  | NTRK1 | 2.75 | 4 | Clustered | 78 |
|  |  | VCP | 6.666666667 | 1 | Clustered | 73 |
|  |  | EP300 | 2.931034483 | 3 | Clustered | 73 |
|  |  | ESR1 | 7.605263158 | 2 | Clustered | 71 |
|  |  | HSPA5 | 7.605263158 | 2 | Clustered | 70 |
|  |  | CSNK2A1 | 6.666666667 | 1 | Clustered | 64 |
|  |  | PIK3R1 | 7.605263158 | 2 | Clustered | 62 |
|  |  | PTK2 | 7.605263158 | 2 | Clustered | 57 |
|  |  | AR | 7.605263158 | 2 | Clustered | 56 |
|  |  | PPP1CA | 7.605263158 | 2 | Clustered | 55 |
|  |  | CSNK2A2 | 6.666666667 | 1 | Clustered | 51 |
|  |  | ESR2 | 0 | 0 |  | 51 |
|  |  | CDK5 | 2.931034483 | 3 | Clustered | 50 |
|  |  | CSNK1D | 7.605263158 | 2 | Clustered | 49 |
|  |  | HRAS | 2.75 | 4 | Clustered | 49 |
|  |  | ABL1 | 7.605263158 | 2 | Clustered | 48 |
|  |  | SYK | 7.605263158 | 2 | Clustered | 47 |
|  |  | MAPT | 7.605263158 | 2 | Clustered | 46 |
|  |  | PLK1 | 2 | 5 | Clustered | 45 |
|  |  | HK1 | 7.605263158 | 2 | Clustered | 44 |
|  |  | NCOR1 | 0 | 0 |  | 42 |
|  |  | APEX1 | 0 | 0 |  | 42 |
|  |  | PTPN11 | 7.605263158 | 2 | Clustered | 41 |
|  |  | PPARG | 0 | 0 |  | 41 |
|  |  | AURKA | 7.605263158 | 2 | Clustered | 40 |
|  |  | HDAC2 | 2 | 5 | Clustered | 40 |
|  |  | TUBB3 | 7.605263158 | 2 | Clustered | 39 |
|  |  | HDAC1 | 2.931034483 | 3 | Clustered | 39 |
|  |  | PARP1 | 2 | 5 | Clustered | 39 |
|  |  | ALDH2 | 7.605263158 | 2 | Clustered | 38 |
|  |  | HSP90B1 | 7.605263158 | 2 | Clustered | 38 |
|  |  | CDK4 | 2.931034483 | 3 | Clustered | 38 |
|  |  | LCK | 2.75 | 4 | Clustered | 38 |
|  |  | IGF1R | 7.605263158 | 2 | Clustered | 37 |
|  |  | INSR | 7.605263158 | 2 | Clustered | 37 |
|  |  | LRRK2 | 7.605263158 | 2 | Clustered | 37 |
|  |  | HDAC3 | 2.931034483 | 3 | Clustered | 37 |
|  |  | HDAC6 | 6.666666667 | 1 | Clustered | 36 |
|  |  | HDAC4 | 2.931034483 | 3 | Clustered | 36 |
|  |  | CDK7 | 2.931034483 | 3 | Clustered | 35 |
|  |  | RAF1 | 2 | 5 | Clustered | 35 |
|  |  | CHEK1 | 7.605263158 | 2 | Clustered | 34 |
|  |  | NCOR2 | 2.931034483 | 3 | Clustered | 34 |
|  |  | PTPN1 | 2.75 | 4 | Clustered | 34 |
|  |  | RPS6KA1 | 2.75 | 4 | Clustered | 33 |
|  |  | PIK3CA | 2.75 | 4 | Clustered | 33 |
|  |  | CCND1 | 0 | 0 |  | 33 |
|  |  | EZR | 2.931034483 | 3 | Clustered | 32 |
|  |  | HDAC5 | 0 | 0 |  | 32 |
|  |  | PGK1 | 7.605263158 | 2 | Clustered | 31 |
|  |  | BDKRB2 | 6.666666667 | 1 | Clustered | 31 |
|  |  | RPS6KB1 | 2.75 | 4 | Clustered | 31 |
|  |  | CFTR | 0 | 0 |  | 31 |
|  |  | ATIC | 7.605263158 | 2 | Clustered | 30 |
|  |  | EIF4A1 | 2.75 | 4 | Seed | 30 |
|  |  | CDK6 | 2.931034483 | 3 | Clustered | 29 |
|  |  | NR3C1 | 2.75 | 4 | Clustered | 29 |
|  |  | CCNB1 | 2 | 5 | Clustered | 29 |
|  |  | PYGL | 7.605263158 | 2 | Clustered | 28 |
|  |  | CDK9 | 2.931034483 | 3 | Clustered | 28 |
|  |  | MTOR | 2.931034483 | 3 | Clustered | 28 |
|  |  | MET | 2.75 | 4 | Clustered | 28 |
|  |  | CDC25A | 2 | 5 | Clustered | 28 |
|  |  | PYGM | 7.605263158 | 2 | Clustered | 27 |
|  |  | TUBB1 | 7.605263158 | 2 | Clustered | 27 |
|  |  | FPR2 | 6.666666667 | 1 | Clustered | 27 |
|  |  | CDK8 | 2 | 5 | Clustered | 27 |
|  |  | CCNC | 0 | 0 |  | 27 |
|  |  | HTT | 0 | 0 |  | 27 |
|  |  | CCND2 | 2.931034483 | 3 | Clustered | 26 |
|  |  | TOP1 | 2.931034483 | 3 | Clustered | 26 |
|  |  | PDGFRB | 2.75 | 4 | Clustered | 26 |
|  |  | CCNA2 | 2 | 5 | Clustered | 26 |
|  |  | PIK3CB | 7.605263158 | 2 | Clustered | 25 |
|  |  | OPRM1 | 6.666666667 | 1 | Clustered | 25 |
|  |  | GRM1 | 6.666666667 | 1 | Clustered | 25 |
|  |  | MAP2K1 | 2.931034483 | 3 | Clustered | 25 |
|  |  | PTPN6 | 7.605263158 | 2 | Clustered | 24 |
|  |  | CCNE1 | 2.931034483 | 3 | Clustered | 24 |
|  |  | CDC25C | 2.75 | 4 | Clustered | 24 |
|  |  | TOP2A | 2.931034483 | 3 | Seed | 23 |
|  |  | BRAF | 2.75 | 4 | Clustered | 23 |
|  |  | KDR | 0 | 0 |  | 23 |
|  |  | AURKB | 0 | 0 |  | 23 |
|  |  | GART | 7.605263158 | 2 | Clustered | 22 |
|  |  | KIT | 7.605263158 | 2 | Clustered | 22 |
|  |  | SGK1 | 2.931034483 | 3 | Clustered | 22 |
|  |  | FGFR1 | 2.75 | 4 | Clustered | 22 |
|  |  | CDC25B | 2 | 5 | Clustered | 22 |
|  |  | RPS6KA2 | 0 | 0 |  | 22 |
|  |  | RET | 7.605263158 | 2 | Clustered | 21 |
|  |  | PDGFRA | 7.605263158 | 2 | Clustered | 21 |
|  |  | DRD2 | 6.666666667 | 1 | Clustered | 21 |
|  |  | HSF1 | 2.931034483 | 3 | Clustered | 21 |
|  |  | PDK1 | 0 | 0 |  | 21 |
|  |  | IMPDH2 | 0 | 0 |  | 21 |
|  |  | CASP3 | 0 | 0 |  | 21 |
|  |  | BAD | 0 | 0 |  | 21 |
|  |  | AKR1B10 | 6.666666667 | 1 | Clustered | 20 |
|  |  | CHRM2 | 6.666666667 | 1 | Clustered | 20 |
|  |  | F2 | 6.666666667 | 1 | Clustered | 20 |
|  |  | CDC7 | 2.931034483 | 3 | Clustered | 20 |
|  |  | MAPKAPK2 | 2 | 5 | Clustered | 20 |
|  |  | CCNH | 2 | 5 | Clustered | 20 |
|  |  | GABBR1 | 6.666666667 | 1 | Clustered | 19 |
|  |  | CNR1 | 6.666666667 | 1 | Clustered | 19 |
|  |  | OPRD1 | 6.666666667 | 1 | Clustered | 19 |
|  |  | ADORA1 | 6.666666667 | 1 | Clustered | 19 |
|  |  | PPARD | 1 | 9 | Clustered | 19 |
|  |  | AKR1A1 | 6.666666667 | 1 | Clustered | 18 |
|  |  | TERT | 2 | 5 | Clustered | 18 |
|  |  | CCND3 | 0 | 0 |  | 18 |
|  |  | ROCK1 | 0 | 0 |  | 18 |
|  |  | AKR1C4 | 6.666666667 | 1 | Clustered | 17 |
|  |  | CNR2 | 6.666666667 | 1 | Clustered | 17 |
|  |  | DRD4 | 6.666666667 | 1 | Clustered | 17 |
|  |  | CXCR1 | 6.666666667 | 1 | Clustered | 17 |
|  |  | VDR | 2.931034483 | 3 | Clustered | 17 |
|  |  | CCNB2 | 2 | 5 | Clustered | 17 |
|  |  | JAK3 | 0 | 0 |  | 17 |
|  |  | PTPRF | 0 | 0 |  | 17 |
|  |  | PSMB5 | 0 | 0 |  | 17 |
|  |  | NR4A1 | 0 | 0 |  | 17 |
|  |  | LDHB | 0 | 0 |  | 17 |
|  |  | ACP1 | 0 | 0 |  | 17 |
|  |  | IKBKE | 0 | 0 |  | 17 |
|  |  | ADORA3 | 6.666666667 | 1 | Clustered | 16 |
|  |  | NPBWR1 | 6.666666667 | 1 | Clustered | 16 |
|  |  | AKR1C2 | 6.666666667 | 1 | Clustered | 16 |
|  |  | CCNA1 | 2.931034483 | 3 | Clustered | 16 |
|  |  | FEN1 | 2.931034483 | 3 | Clustered | 16 |
|  |  | TTK | 0 | 0 |  | 16 |
|  |  | DAPK1 | 0 | 0 |  | 16 |
|  |  | PTPN2 | 0 | 0 |  | 16 |
|  |  | ROCK2 | 0 | 0 |  | 16 |
|  |  | AKR1B1 | 0 | 0 |  | 16 |
|  |  | GRM4 | 6.666666667 | 1 | Clustered | 15 |
|  |  | DNM1 | 2.931034483 | 3 | Clustered | 15 |
|  |  | AXL | 2.75 | 4 | Clustered | 15 |
|  |  | ALK | 0 | 0 |  | 15 |
|  |  | NEK2 | 0 | 0 |  | 15 |
|  |  | PIK3CG | 0 | 0 |  | 15 |
|  |  | PKN1 | 0 | 0 |  | 15 |
|  |  | CCNE2 | 0 | 0 |  | 15 |
|  |  | MCL1 | 0 | 0 |  | 15 |
|  |  | TAS2R31 | 6.666666667 | 1 | Seed | 14 |
|  |  | AKR1C1 | 6.666666667 | 1 | Clustered | 14 |
|  |  | WEE1 | 2.931034483 | 3 | Clustered | 14 |
|  |  | BRD4 | 0 | 0 |  | 14 |
|  |  | PIM1 | 0 | 0 |  | 14 |
|  |  | ESRRB | 0 | 0 |  | 14 |
|  |  | CDK5R1 | 0 | 0 |  | 14 |
|  |  | ADK | 7.605263158 | 2 | Clustered | 13 |
|  |  | AVPR1A | 6.666666667 | 1 | Clustered | 13 |
|  |  | HCRTR1 | 6.666666667 | 1 | Clustered | 13 |
|  |  | HTR2A | 6.666666667 | 1 | Clustered | 13 |
|  |  | PTGER1 | 6.666666667 | 1 | Clustered | 13 |
|  |  | AHR | 2.931034483 | 3 | Clustered | 13 |
|  |  | CCNT1 | 2.931034483 | 3 | Clustered | 13 |
|  |  | CYP1A1 | 2.857142857 | 7 | Clustered | 13 |
|  |  | BRD2 | 0 | 0 |  | 13 |
|  |  | PKN2 | 0 | 0 |  | 13 |
|  |  | TGFBR1 | 0 | 0 |  | 13 |
|  |  | PRKCQ | 0 | 0 |  | 13 |
|  |  | PPARA | 0 | 0 |  | 13 |
|  |  | MGMT | 0 | 0 |  | 13 |
|  |  | NR1H3 | 0 | 0 |  | 13 |
|  |  | HDAC9 | 0 | 0 |  | 13 |
|  |  | HCRTR2 | 6.666666667 | 1 | Clustered | 12 |
|  |  | FFAR1 | 6.666666667 | 1 | Clustered | 12 |
|  |  | HTR2C | 6.666666667 | 1 | Clustered | 12 |
|  |  | OXTR | 6.666666667 | 1 | Clustered | 12 |
|  |  | AVPR2 | 2.857142857 | 6 | Clustered | 12 |
|  |  | CYP51A1 | 2.857142857 | 7 | Clustered | 12 |
|  |  | IL2 | 0 | 0 |  | 12 |
|  |  | HDAC7 | 0 | 0 |  | 12 |
|  |  | MYLK | 0 | 0 |  | 12 |
|  |  | SF3B3 | 0 | 0 |  | 12 |
|  |  | PLK3 | 0 | 0 |  | 11 |
|  |  | PLAA | 0 | 0 |  | 11 |
|  |  | MIF | 0 | 0 |  | 11 |
|  |  | NOS2 | 0 | 0 |  | 11 |
|  |  | FLT3 | 0 | 0 |  | 11 |
|  |  | IMPDH1 | 0 | 0 |  | 11 |
|  |  | GABRG2 | 0 | 0 |  | 11 |
|  |  | MME | 0 | 0 |  | 11 |
|  |  | EPHB2 | 0 | 0 |  | 11 |
|  |  | FABP4 | 2.931034483 | 3 | Clustered | 10 |
|  |  | ADORA2A | 2.857142857 | 6 | Clustered | 10 |
|  |  | DYRK2 | 2 | 5 | Seed | 10 |
|  |  | SCD | 0 | 0 |  | 10 |
|  |  | G6PD | 0 | 0 |  | 10 |
|  |  | CASP7 | 0 | 0 |  | 10 |
|  |  | SIRT2 | 0 | 0 |  | 10 |
|  |  | SREBF2 | 0 | 0 |  | 10 |
|  |  | NR1H2 | 0 | 0 |  | 10 |
|  |  | PDE4D | 0 | 0 |  | 10 |
|  |  | MMP1 | 0 | 0 |  | 10 |
|  |  | ADAM17 | 0 | 0 |  | 10 |
|  |  | CYP1B1 | 2.857142857 | 7 | Seed | 9 |
|  |  | MMP7 | 1 | 9 | Seed | 9 |
|  |  | PLAU | 1 | 9 | Clustered | 9 |
|  |  | MMP2 | 0 | 0 |  | 9 |
|  |  | PIK3CD | 0 | 0 |  | 9 |
|  |  | HMGCR | 0 | 0 |  | 9 |
|  |  | SAE1 | 0 | 0 |  | 9 |
|  |  | PLG | 0 | 0 |  | 9 |
|  |  | BRD3 | 0 | 0 |  | 9 |
|  |  | ERCC5 | 0 | 0 |  | 9 |
|  |  | PTGS2 | 0 | 0 |  | 9 |
|  |  | CYP19A1 | 2.857142857 | 7 | Clustered | 8 |
|  |  | CYP17A1 | 2.857142857 | 7 | Clustered | 8 |
|  |  | PTGER2 | 2.857142857 | 6 | Clustered | 8 |
|  |  | GABRA3 | 1.5 | 8 | Clustered | 8 |
|  |  | GABRB3 | 1.5 | 8 | Clustered | 8 |
|  |  | GABRB2 | 1.5 | 8 | Clustered | 8 |
|  |  | PLK2 | 0 | 0 |  | 8 |
|  |  | MKNK1 | 0 | 0 |  | 8 |
|  |  | GABRA1 | 0 | 0 |  | 8 |
|  |  | CA9 | 0 | 0 |  | 8 |
|  |  | DYRK1B | 0 | 0 |  | 8 |
|  |  | GRK6 | 0 | 0 |  | 8 |
|  |  | MPG | 0 | 0 |  | 8 |
|  |  | ESRRA | 0 | 0 |  | 8 |
|  |  | ALOX5 | 0 | 0 |  | 8 |
|  |  | SLC29A1 | 0 | 0 |  | 8 |
|  |  | TLR9 | 0 | 0 |  | 8 |
|  |  | ADORA2B | 2.857142857 | 6 | Clustered | 7 |
|  |  | ARG1 | 0 | 0 |  | 7 |
|  |  | FABP5 | 0 | 0 |  | 7 |
|  |  | PIM2 | 0 | 0 |  | 7 |
|  |  | ABCB1 | 0 | 0 |  | 7 |
|  |  | COMT | 0 | 0 |  | 7 |
|  |  | CLK3 | 0 | 0 |  | 7 |
|  |  | NR1H4 | 0 | 0 |  | 7 |
|  |  | NDUFA4 | 0 | 0 |  | 7 |
|  |  | HK2 | 0 | 0 |  | 7 |
|  |  | CLK2 | 0 | 0 |  | 7 |
|  |  | ABCC1 | 0 | 0 |  | 7 |
|  |  | PLAT | 0 | 0 |  | 7 |
|  |  | AKR1C3 | 0 | 0 |  | 7 |
|  |  | CAMK2B | 0 | 0 |  | 7 |
|  |  | CBR1 | 0 | 0 |  | 7 |
|  |  | CYP2C19 | 2.857142857 | 7 | Clustered | 6 |
|  |  | CYP11B1 | 2.857142857 | 7 | Clustered | 6 |
|  |  | CRHR1 | 2.857142857 | 6 | Clustered | 6 |
|  |  | GABRA5 | 1.5 | 8 | Seed | 6 |
|  |  | HSD17B3 | 0 | 0 |  | 6 |
|  |  | DUSP3 | 0 | 0 |  | 6 |
|  |  | NUAK1 | 0 | 0 |  | 6 |
|  |  | FABP1 | 0 | 0 |  | 6 |
|  |  | NAE1 | 0 | 0 |  | 6 |
|  |  | GLO1 | 0 | 0 |  | 6 |
|  |  | CLK1 | 0 | 0 |  | 6 |
|  |  | FDFT1 | 0 | 0 |  | 6 |
|  |  | GABRA6 | 0 | 0 |  | 6 |
|  |  | FLT1 | 0 | 0 |  | 6 |
|  |  | CCNB3 | 0 | 0 |  | 6 |
|  |  | TAAR1 | 2.857142857 | 6 | Seed | 5 |
|  |  | GPBAR1 | 2.857142857 | 6 | Clustered | 5 |
|  |  | PFKFB3 | 0 | 0 |  | 5 |
|  |  | RORC | 0 | 0 |  | 5 |
|  |  | NEK6 | 0 | 0 |  | 5 |
|  |  | MMP3 | 0 | 0 |  | 5 |
|  |  | TYMS | 0 | 0 |  | 5 |
|  |  | POLB | 0 | 0 |  | 5 |
|  |  | TNKS | 0 | 0 |  | 5 |
|  |  | DHCR7 | 0 | 0 |  | 5 |
|  |  | BACE1 | 0 | 0 |  | 5 |
|  |  | HDAC10 | 0 | 0 |  | 5 |
|  |  | ADAM10 | 0 | 0 |  | 5 |
|  |  | GABRA2 | 0 | 0 |  | 5 |
|  |  | NR1I3 | 0 | 0 |  | 5 |
|  |  | MERTK | 0 | 0 |  | 5 |
|  |  | TTR | 0 | 0 |  | 4 |
|  |  | SLC6A3 | 0 | 0 |  | 4 |
|  |  | UBA2 | 0 | 0 |  | 4 |
|  |  | ALPL | 0 | 0 |  | 4 |
|  |  | CA2 | 0 | 0 |  | 4 |
|  |  | F10 | 0 | 0 |  | 4 |
|  |  | SQLE | 0 | 0 |  | 4 |
|  |  | HTR3A | 0 | 0 |  | 4 |
|  |  | ERN1 | 0 | 0 |  | 4 |
|  |  | FABP3 | 0 | 0 |  | 4 |
|  |  | PTPRS | 0 | 0 |  | 4 |
|  |  | QPCTL | 0 | 0 |  | 4 |
|  |  | PDE3B | 0 | 0 |  | 4 |
|  |  | MMP9 | 0 | 0 |  | 4 |
|  |  | SLC6A4 | 0 | 0 |  | 4 |
|  |  | SIGMAR1 | 0 | 0 |  | 4 |
|  |  | ELANE | 0 | 0 |  | 4 |
|  |  | ABCG2 | 0 | 0 |  | 4 |
|  |  | F3 | 0 | 0 |  | 3 |
|  |  | NADK | 0 | 0 |  | 3 |
|  |  | LNPEP | 0 | 0 |  | 3 |
|  |  | FOLR1 | 0 | 0 |  | 3 |
|  |  | SLC2A1 | 0 | 0 |  | 3 |
|  |  | HDAC8 | 0 | 0 |  | 3 |
|  |  | PIM3 | 0 | 0 |  | 3 |
|  |  | AMY1A | 0 | 0 |  | 3 |
|  |  | ALOX5AP | 0 | 0 |  | 3 |
|  |  | RGS4 | 0 | 0 |  | 3 |
|  |  | BRPF1 | 0 | 0 |  | 3 |
|  |  | RORA | 0 | 0 |  | 3 |
|  |  | CTSG | 0 | 0 |  | 3 |
|  |  | PDE3A | 0 | 0 |  | 3 |
|  |  | CSNK1G1 | 0 | 0 |  | 3 |
|  |  | CHRNA7 | 0 | 0 |  | 3 |
|  |  | PREP | 0 | 0 |  | 3 |
|  |  | TNKS2 | 0 | 0 |  | 3 |
|  |  | MBD2 | 0 | 0 |  | 3 |
|  |  | DBF4 | 0 | 0 |  | 2 |
|  |  | TBXAS1 | 0 | 0 |  | 2 |
|  |  | CA3 | 0 | 0 |  | 2 |
|  |  | HDAC11 | 0 | 0 |  | 2 |
|  |  | PRF1 | 0 | 0 |  | 2 |
|  |  | FAAH | 0 | 0 |  | 2 |
|  |  | ODC1 | 0 | 0 |  | 2 |
|  |  | GLRA1 | 0 | 0 |  | 2 |
|  |  | MPO | 0 | 0 |  | 2 |
|  |  | NQO2 | 0 | 0 |  | 2 |
|  |  | CLK4 | 0 | 0 |  | 2 |
|  |  | UGT2B7 | 0 | 0 |  | 2 |
|  |  | DHFR | 0 | 0 |  | 2 |
|  |  | ANPEP | 0 | 0 |  | 2 |
|  |  | MMP13 | 0 | 0 |  | 2 |
|  |  | SLC1A3 | 0 | 0 |  | 2 |
|  |  | XDH | 0 | 0 |  | 2 |
|  |  | CA14 | 0 | 0 |  | 2 |
|  |  | STS | 0 | 0 |  | 1 |
|  |  | SLC27A1 | 0 | 0 |  | 1 |
|  |  | ALOX15B | 0 | 0 |  | 1 |
|  |  | HSD17B1 | 0 | 0 |  | 1 |
|  |  | SHBG | 0 | 0 |  | 1 |
|  |  | FPGS | 0 | 0 |  | 1 |
|  |  | CD38 | 0 | 0 |  | 1 |
|  |  | CMA1 | 0 | 0 |  | 1 |
|  |  | CTSS | 0 | 0 |  | 1 |
|  |  | PDE4C | 0 | 0 |  | 1 |
|  |  | PTGES | 0 | 0 |  | 1 |
|  |  | NOX4 | 0 | 0 |  | 1 |
|  |  | MAOB | 0 | 0 |  | 1 |
|  |  | ACHE | 0 | 0 |  | 1 |
|  |  | CES2 | 0 | 0 |  | 1 |
|  |  | SLC5A1 | 0 | 0 |  | 1 |
|  |  | HSD11B1 | 0 | 0 |  | 1 |
|  |  | MAOA | 0 | 0 |  | 1 |
|  |  | EPHX2 | 0 | 0 |  | 1 |
|  |  | TRPM8 | 0 | 0 |  | 1 |
|  |  | ALOX15 | 0 | 0 |  | 1 |
|  |  | PDE10A | 0 | 0 |  | 1 |
|  |  | SLC19A1 | 0 | 0 |  | 1 |
|  |  | CA5A | 0 | 0 |  | 1 |
|  |  | BMP1 | 0 | 0 |  | 1 |
|  |  | CA4 | 0 | 0 |  | 1 |
|  |  | DAO | 0 | 0 |  | 1 |
|  |  | ALPG | 0 | 0 |  | 1 |
|  |  | PTGS1 | 0 | 0 |  | 1 |
|  |  | DHODH | 0 | 0 |  | 1 |
|  |  | MMP12 | 0 | 0 |  | 1 |
|  | VMC-related targets | SRC | 4.1 | 1 | Clustered | 28 |
|  |  | MAPK1 | 4.1 | 1 | Clustered | 28 |
|  |  | HSP90AA1 | 1.857142857 | 2 | Clustered | 28 |
|  |  | PRKCA | 4.1 | 1 | Clustered | 27 |
|  |  | EGFR | 4.1 | 1 | Clustered | 26 |
|  |  | HRAS | 4.1 | 1 | Clustered | 21 |
|  |  | JUN | 0 | 0 |  | 21 |
|  |  | NTRK1 | 4.1 | 1 | Clustered | 20 |
|  |  | PTK2 | 4.1 | 1 | Clustered | 20 |
|  |  | PTPN11 | 4.1 | 1 | Clustered | 19 |
|  |  | AKT1 | 1.857142857 | 2 | Clustered | 18 |
|  |  | LCK | 1.857142857 | 2 | Clustered | 16 |
|  |  | HSPA5 | 1.857142857 | 2 | Clustered | 15 |
|  |  | MET | 4.1 | 1 | Clustered | 14 |
|  |  | RET | 4.1 | 1 | Seed | 14 |
|  |  | PIK3CA | 1.857142857 | 2 | Clustered | 14 |
|  |  | ABL1 | 1.857142857 | 2 | Clustered | 14 |
|  |  | PARP1 | 0 | 0 |  | 12 |
|  |  | RAF1 | 1.857142857 | 2 | Seed | 11 |
|  |  | PPARG | 0 | 0 |  | 10 |
|  |  | KIT | 0 | 0 |  | 9 |
|  |  | MTOR | 0 | 0 |  | 9 |
|  |  | MMP1 | 0 | 0 |  | 9 |
|  |  | PIK3CG | 0 | 0 |  | 8 |
|  |  | CASP3 | 0 | 0 |  | 8 |
|  |  | RPS6KB1 | 0 | 0 |  | 7 |
|  |  | VDR | 0 | 0 |  | 6 |
|  |  | BDKRB2 | 0 | 0 |  | 6 |
|  |  | IL2 | 0 | 0 |  | 6 |
|  |  | F2 | 0 | 0 |  | 6 |
|  |  | TUBB1 | 0 | 0 |  | 6 |
|  |  | CYP1A1 | 0 | 0 |  | 5 |
|  |  | NOS2 | 0 | 0 |  | 5 |
|  |  | ADAM17 | 0 | 0 |  | 5 |
|  |  | PLG | 0 | 0 |  | 5 |
|  |  | MMP2 | 0 | 0 |  | 5 |
|  |  | PPARA | 0 | 0 |  | 5 |
|  |  | DRD2 | 0 | 0 |  | 4 |
|  |  | CYP51A1 | 0 | 0 |  | 4 |
|  |  | CASP7 | 0 | 0 |  | 4 |
|  |  | HSF1 | 0 | 0 |  | 4 |
|  |  | CCND1 | 0 | 0 |  | 4 |
|  |  | FDFT1 | 0 | 0 |  | 3 |
|  |  | MMP9 | 0 | 0 |  | 3 |
|  |  | ELANE | 0 | 0 |  | 3 |
|  |  | FLT1 | 0 | 0 |  | 3 |
|  |  | CHRM2 | 0 | 0 |  | 3 |
|  |  | MIF | 0 | 0 |  | 3 |
|  |  | G6PD | 0 | 0 |  | 3 |
|  |  | TLR9 | 0 | 0 |  | 3 |
|  |  | ARG1 | 0 | 0 |  | 3 |
|  |  | CTSG | 0 | 0 |  | 3 |
|  |  | PLAT | 0 | 0 |  | 3 |
|  |  | SLC6A4 | 0 | 0 |  | 3 |
|  |  | PRF1 | 0 | 0 |  | 2 |
|  |  | ABCC1 | 0 | 0 |  | 2 |
|  |  | CYP2C19 | 0 | 0 |  | 2 |
|  |  | ABCB1 | 0 | 0 |  | 2 |
|  |  | F3 | 0 | 0 |  | 2 |
|  |  | ADORA2A | 0 | 0 |  | 2 |
|  |  | TGFBR1 | 0 | 0 |  | 2 |
|  |  | MMP3 | 0 | 0 |  | 2 |
|  |  | MPO | 0 | 0 |  | 1 |
|  |  | CMA1 | 0 | 0 |  | 1 |
|  |  | ALOX5 | 0 | 0 |  | 1 |
|  |  | FABP3 | 0 | 0 |  | 1 |
| YGZ | whole targets | APP | 9.952380952 | 3 | Clustered | 104 |
|  |  | HSP90AA1 | 5.551724138 | 1 | Clustered | 101 |
|  |  | EGFR | 4.583333333 | 2 | Clustered | 97 |
|  |  | SRC | 6.111111111 | 4 | Clustered | 94 |
|  |  | HSP90AB1 | 4.583333333 | 2 | Clustered | 91 |
|  |  | HSPA1A | 4.583333333 | 2 | Clustered | 72 |
|  |  | AKT1 | 5.551724138 | 1 | Clustered | 66 |
|  |  | GSK3B | 5.551724138 | 1 | Clustered | 66 |
|  |  | CDK1 | 4.583333333 | 2 | Clustered | 64 |
|  |  | STAT3 | 5.551724138 | 1 | Clustered | 62 |
|  |  | MAPK3 | 5.551724138 | 1 | Clustered | 61 |
|  |  | CDK2 | 4.583333333 | 2 | Clustered | 59 |
|  |  | PTK2 | 5.551724138 | 1 | Clustered | 55 |
|  |  | PIK3R1 | 5.551724138 | 1 | Clustered | 53 |
|  |  | ERBB2 | 6.111111111 | 4 | Clustered | 48 |
|  |  | ESR1 | 6.111111111 | 4 | Clustered | 48 |
|  |  | MAPK14 | 5.551724138 | 1 | Clustered | 47 |
|  |  | CSNK2A1 | 5.551724138 | 1 | Clustered | 43 |
|  |  | MDM2 | 5.551724138 | 1 | Clustered | 43 |
|  |  | STAT1 | 4.583333333 | 2 | Clustered | 43 |
|  |  | PTPN11 | 5.551724138 | 1 | Clustered | 41 |
|  |  | CDK5 | 1.5 | 5 | Clustered | 41 |
|  |  | INSR | 4.583333333 | 2 | Clustered | 40 |
|  |  | AR | 5.551724138 | 1 | Clustered | 39 |
|  |  | PRKDC | 5.551724138 | 1 | Clustered | 38 |
|  |  | LYN | 4.583333333 | 2 | Seed | 38 |
|  |  | IGF1R | 5.551724138 | 1 | Clustered | 36 |
|  |  | MAPT | 5.551724138 | 1 | Clustered | 35 |
|  |  | SYK | 4.583333333 | 2 | Clustered | 35 |
|  |  | CDK4 | 1 | 11 | Clustered | 34 |
|  |  | AURKA | 5.551724138 | 1 | Seed | 32 |
|  |  | HIF1A | 5.551724138 | 1 | Clustered | 32 |
|  |  | RPS6KB1 | 4.583333333 | 2 | Clustered | 32 |
|  |  | MAPK8 | 4.583333333 | 2 | Clustered | 32 |
|  |  | PLK1 | 1.5 | 5 | Clustered | 32 |
|  |  | JAK2 | 5.551724138 | 1 | Clustered | 31 |
|  |  | PIK3CA | 5.551724138 | 1 | Clustered | 31 |
|  |  | APEX1 | 0 | 0 |  | 31 |
|  |  | ESR2 | 1.5 | 5 | Clustered | 30 |
|  |  | PARP1 | 1.5 | 5 | Clustered | 30 |
|  |  | PTPN1 | 1.5 | 5 | Clustered | 30 |
|  |  | CHEK1 | 1.5 | 5 | Clustered | 29 |
|  |  | PPARG | 1.5 | 5 | Clustered | 29 |
|  |  | MET | 4.583333333 | 2 | Clustered | 28 |
|  |  | NR3C1 | 4.583333333 | 2 | Clustered | 28 |
|  |  | PTPN6 | 5.551724138 | 1 | Clustered | 27 |
|  |  | VEGFA | 4.583333333 | 2 | Clustered | 27 |
|  |  | MAP2K1 | 4.583333333 | 2 | Clustered | 27 |
|  |  | CDK6 | 4.583333333 | 2 | Clustered | 27 |
|  |  | PDGFRB | 5.551724138 | 1 | Clustered | 26 |
|  |  | KDR | 4.583333333 | 2 | Clustered | 26 |
|  |  | DRD2 | 9.952380952 | 3 | Clustered | 25 |
|  |  | CHRM2 | 9.952380952 | 3 | Clustered | 25 |
|  |  | ADORA1 | 9.952380952 | 3 | Clustered | 25 |
|  |  | PTGER3 | 9.952380952 | 3 | Clustered | 25 |
|  |  | SNCA | 4.583333333 | 2 | Clustered | 25 |
|  |  | MTOR | 4.583333333 | 2 | Clustered | 25 |
|  |  | JAK1 | 4.583333333 | 2 | Clustered | 25 |
|  |  | CCND1 | 1.5 | 5 | Clustered | 24 |
|  |  | CNR1 | 9.952380952 | 3 | Clustered | 23 |
|  |  | F2 | 6.111111111 | 4 | Clustered | 23 |
|  |  | GRM1 | 6.111111111 | 4 | Clustered | 23 |
|  |  | PIK3CB | 4.583333333 | 2 | Clustered | 23 |
|  |  | ACKR3 | 9.952380952 | 3 | Clustered | 22 |
|  |  | OPRM1 | 9.952380952 | 3 | Clustered | 22 |
|  |  | PGD | 5.551724138 | 1 | Clustered | 22 |
|  |  | CCNB1 | 1.6 | 10 | Clustered | 22 |
|  |  | BCL2 | 1.5 | 5 | Clustered | 22 |
|  |  | FGF1 | 0 | 0 |  | 22 |
|  |  | ADRA2A | 9.952380952 | 3 | Clustered | 21 |
|  |  | DRD4 | 9.952380952 | 3 | Clustered | 21 |
|  |  | DRD3 | 9.952380952 | 3 | Clustered | 21 |
|  |  | CXCR1 | 9.952380952 | 3 | Clustered | 21 |
|  |  | CDC25A | 1.6 | 10 | Clustered | 21 |
|  |  | FKBP5 | 1.5 | 5 | Clustered | 21 |
|  |  | HTR1A | 9.952380952 | 3 | Clustered | 20 |
|  |  | ADRA2B | 9.952380952 | 3 | Clustered | 20 |
|  |  | CNR2 | 9.952380952 | 3 | Clustered | 20 |
|  |  | GRM4 | 9.952380952 | 3 | Clustered | 20 |
|  |  | OPRK1 | 9.952380952 | 3 | Clustered | 20 |
|  |  | HTR1B | 9.952380952 | 3 | Clustered | 20 |
|  |  | ADRA2C | 9.952380952 | 3 | Clustered | 20 |
|  |  | ADORA3 | 9.952380952 | 3 | Clustered | 20 |
|  |  | AXL | 4.583333333 | 2 | Clustered | 20 |
|  |  | CDK8 | 0 | 0 |  | 20 |
|  |  | CFTR | 0 | 0 |  | 20 |
|  |  | TAS2R31 | 9.952380952 | 3 | Seed | 19 |
|  |  | GCK | 5.551724138 | 1 | Clustered | 19 |
|  |  | CCNA2 | 1.6 | 10 | Clustered | 19 |
|  |  | JAK3 | 0 | 0 |  | 19 |
|  |  | ALK | 0 | 0 |  | 19 |
|  |  | PDPK1 | 0 | 0 |  | 19 |
|  |  | DYRK1A | 0 | 0 |  | 19 |
|  |  | PSEN1 | 0 | 0 |  | 19 |
|  |  | PTAFR | 6.111111111 | 4 | Clustered | 18 |
|  |  | HTR2A | 6.111111111 | 4 | Clustered | 18 |
|  |  | FGR | 4.583333333 | 2 | Clustered | 18 |
|  |  | TNFRSF1A | 4.583333333 | 2 | Clustered | 18 |
|  |  | CCNE1 | 1.5 | 5 | Clustered | 18 |
|  |  | CCNC | 1 | 11 | Clustered | 18 |
|  |  | PTGER1 | 6.111111111 | 4 | Clustered | 17 |
|  |  | TOP1 | 0 | 0 |  | 17 |
|  |  | TUBB1 | 0 | 0 |  | 17 |
|  |  | PTPN2 | 0 | 0 |  | 17 |
|  |  | HCRTR1 | 6.111111111 | 4 | Clustered | 16 |
|  |  | P2RY1 | 6.111111111 | 4 | Clustered | 16 |
|  |  | PYGL | 5.551724138 | 1 | Clustered | 16 |
|  |  | BRAF | 0 | 0 |  | 16 |
|  |  | DNMT1 | 0 | 0 |  | 16 |
|  |  | PGF | 0 | 0 |  | 16 |
|  |  | TNK2 | 0 | 0 |  | 16 |
|  |  | CCNB2 | 0 | 0 |  | 16 |
|  |  | TERT | 0 | 0 |  | 16 |
|  |  | AVPR1B | 6.111111111 | 4 | Clustered | 15 |
|  |  | PTGFR | 6.111111111 | 4 | Clustered | 15 |
|  |  | OXTR | 6.111111111 | 4 | Clustered | 15 |
|  |  | HTR2C | 6.111111111 | 4 | Clustered | 15 |
|  |  | ADRA1A | 6.111111111 | 4 | Clustered | 15 |
|  |  | HTR2B | 6.111111111 | 4 | Clustered | 15 |
|  |  | ADRA1D | 6.111111111 | 4 | Clustered | 15 |
|  |  | CDC25B | 1.5 | 5 | Clustered | 15 |
|  |  | PPM1A | 0 | 0 |  | 15 |
|  |  | TOP2A | 0 | 0 |  | 15 |
|  |  | PIM1 | 0 | 0 |  | 15 |
|  |  | UTS2R | 6.111111111 | 4 | Seed | 14 |
|  |  | AURKB | 0 | 0 |  | 14 |
|  |  | TYK2 | 0 | 0 |  | 14 |
|  |  | ACP1 | 0 | 0 |  | 14 |
|  |  | TEK | 0 | 0 |  | 14 |
|  |  | FLT3 | 0 | 0 |  | 14 |
|  |  | AKR1C2 | 5.551724138 | 1 | Clustered | 13 |
|  |  | AKR1A1 | 5.551724138 | 1 | Clustered | 13 |
|  |  | AKR1C1 | 5.551724138 | 1 | Clustered | 13 |
|  |  | AKR1B10 | 5.551724138 | 1 | Clustered | 13 |
|  |  | CCNA1 | 1.6 | 10 | Clustered | 13 |
|  |  | NOS2 | 0 | 0 |  | 13 |
|  |  | PIK3CG | 0 | 0 |  | 13 |
|  |  | AKR1C4 | 5.551724138 | 1 | Clustered | 12 |
|  |  | DNM1 | 0 | 0 |  | 12 |
|  |  | FLT4 | 0 | 0 |  | 12 |
|  |  | PSEN2 | 1.8 | 9 | Clustered | 11 |
|  |  | FLT1 | 0 | 0 |  | 11 |
|  |  | AKR1B1 | 0 | 0 |  | 11 |
|  |  | NR1H3 | 0 | 0 |  | 11 |
|  |  | POLA1 | 0 | 0 |  | 11 |
|  |  | ROS1 | 0 | 0 |  | 11 |
|  |  | PKN1 | 0 | 0 |  | 11 |
|  |  | PDE4D | 0 | 0 |  | 11 |
|  |  | CDK5R1 | 0 | 0 |  | 11 |
|  |  | CYP17A1 | 2.333333333 | 7 | Clustered | 10 |
|  |  | NCSTN | 1.8 | 9 | Clustered | 10 |
|  |  | DAPK1 | 0 | 0 |  | 10 |
|  |  | PPARA | 0 | 0 |  | 10 |
|  |  | SCD | 0 | 0 |  | 10 |
|  |  | PTPRF | 0 | 0 |  | 10 |
|  |  | PTGS2 | 0 | 0 |  | 10 |
|  |  | CYP51A1 | 0 | 0 |  | 10 |
|  |  | DYRK2 | 0 | 0 |  | 10 |
|  |  | AVPR2 | 2.5 | 6 | Clustered | 9 |
|  |  | DRD1 | 2.5 | 6 | Clustered | 9 |
|  |  | PLK4 | 1 | 11 | Clustered | 9 |
|  |  | PPARD | 0 | 0 |  | 9 |
|  |  | EPHB4 | 0 | 0 |  | 9 |
|  |  | CA9 | 0 | 0 |  | 9 |
|  |  | MAPK10 | 0 | 0 |  | 9 |
|  |  | MAP3K8 | 0 | 0 |  | 9 |
|  |  | VDR | 0 | 0 |  | 9 |
|  |  | G6PD | 0 | 0 |  | 9 |
|  |  | HTR6 | 2.5 | 6 | Clustered | 8 |
|  |  | ADORA2A | 2.5 | 6 | Clustered | 8 |
|  |  | GABRG2 | 2 | 8 | Clustered | 8 |
|  |  | GABRB2 | 2 | 8 | Clustered | 8 |
|  |  | AHR | 0 | 0 |  | 8 |
|  |  | CLK3 | 0 | 0 |  | 8 |
|  |  | CAMK2B | 0 | 0 |  | 8 |
|  |  | NEK2 | 0 | 0 |  | 8 |
|  |  | GRK6 | 0 | 0 |  | 8 |
|  |  | PTGER2 | 2.5 | 6 | Clustered | 7 |
|  |  | CYP19A1 | 2.333333333 | 7 | Clustered | 7 |
|  |  | GABRA3 | 2 | 8 | Clustered | 7 |
|  |  | CCNB3 | 1.6 | 10 | Clustered | 7 |
|  |  | HSD17B3 | 0 | 0 |  | 7 |
|  |  | MYLK | 0 | 0 |  | 7 |
|  |  | PIK3CD | 0 | 0 |  | 7 |
|  |  | FABP5 | 0 | 0 |  | 7 |
|  |  | YARS1 | 0 | 0 |  | 7 |
|  |  | ESRRA | 0 | 0 |  | 7 |
|  |  | CCNE2 | 0 | 0 |  | 7 |
|  |  | DYRK1B | 0 | 0 |  | 7 |
|  |  | FABP4 | 0 | 0 |  | 7 |
|  |  | PLG | 0 | 0 |  | 7 |
|  |  | CYP2C19 | 2.333333333 | 7 | Clustered | 6 |
|  |  | CYP1B1 | 2.333333333 | 7 | Clustered | 6 |
|  |  | CYP2D6 | 2.333333333 | 7 | Clustered | 6 |
|  |  | GABRB3 | 2 | 8 | Clustered | 6 |
|  |  | PSENEN | 1.8 | 9 | Clustered | 6 |
|  |  | SERPINE1 | 0 | 0 |  | 6 |
|  |  | ICAM1 | 0 | 0 |  | 6 |
|  |  | MMP14 | 0 | 0 |  | 6 |
|  |  | OGA | 0 | 0 |  | 6 |
|  |  | NR1H2 | 0 | 0 |  | 6 |
|  |  | AKR1C3 | 0 | 0 |  | 6 |
|  |  | ALOX5 | 0 | 0 |  | 6 |
|  |  | MMP2 | 0 | 0 |  | 6 |
|  |  | CLK2 | 0 | 0 |  | 6 |
|  |  | CBR1 | 0 | 0 |  | 6 |
|  |  | BACE1 | 0 | 0 |  | 6 |
|  |  | MAP3K12 | 0 | 0 |  | 6 |
|  |  | CRHR1 | 2.5 | 6 | Clustered | 5 |
|  |  | CYP24A1 | 2.333333333 | 7 | Seed | 5 |
|  |  | GABRA5 | 2 | 8 | Seed | 5 |
|  |  | APH1A | 1.8 | 9 | Seed | 5 |
|  |  | APH1B | 1.8 | 9 | Clustered | 5 |
|  |  | PFKFB3 | 0 | 0 |  | 5 |
|  |  | GABRA1 | 0 | 0 |  | 5 |
|  |  | ABCB1 | 0 | 0 |  | 5 |
|  |  | CLK1 | 0 | 0 |  | 5 |
|  |  | ARG1 | 0 | 0 |  | 5 |
|  |  | HMGCR | 0 | 0 |  | 5 |
|  |  | SLC6A4 | 0 | 0 |  | 5 |
|  |  | ABCG2 | 0 | 0 |  | 5 |
|  |  | NUAK1 | 0 | 0 |  | 5 |
|  |  | GLO1 | 0 | 0 |  | 5 |
|  |  | CD81 | 0 | 0 |  | 5 |
|  |  | GABRA2 | 0 | 0 |  | 5 |
|  |  | MPG | 0 | 0 |  | 5 |
|  |  | GSR | 0 | 0 |  | 5 |
|  |  | MMP3 | 0 | 0 |  | 5 |
|  |  | TTR | 0 | 0 |  | 4 |
|  |  | ABCC1 | 0 | 0 |  | 4 |
|  |  | KCNA5 | 0 | 0 |  | 4 |
|  |  | SREBF2 | 0 | 0 |  | 4 |
|  |  | IDO1 | 0 | 0 |  | 4 |
|  |  | SMO | 0 | 0 |  | 4 |
|  |  | DHCR7 | 0 | 0 |  | 4 |
|  |  | NEK6 | 0 | 0 |  | 4 |
|  |  | SQLE | 0 | 0 |  | 4 |
|  |  | TNNC1 | 1 | 12 | Seed | 3 |
|  |  | TNNT2 | 1 | 12 | Clustered | 3 |
|  |  | TNNI3 | 1 | 12 | Clustered | 3 |
|  |  | RORC | 0 | 0 |  | 3 |
|  |  | PDE3B | 0 | 0 |  | 3 |
|  |  | FABP1 | 0 | 0 |  | 3 |
|  |  | PREP | 0 | 0 |  | 3 |
|  |  | DYRK3 | 0 | 0 |  | 3 |
|  |  | NR1I3 | 0 | 0 |  | 3 |
|  |  | KLK2 | 0 | 0 |  | 3 |
|  |  | KCNH2 | 0 | 0 |  | 3 |
|  |  | FNTB | 0 | 0 |  | 3 |
|  |  | PTPRS | 0 | 0 |  | 3 |
|  |  | SELE | 0 | 0 |  | 3 |
|  |  | MMP13 | 0 | 0 |  | 3 |
|  |  | PDE4B | 0 | 0 |  | 2 |
|  |  | FNTA | 0 | 0 |  | 2 |
|  |  | POLB | 0 | 0 |  | 2 |
|  |  | UGT2B7 | 0 | 0 |  | 2 |
|  |  | CA3 | 0 | 0 |  | 2 |
|  |  | ST3GAL3 | 0 | 0 |  | 2 |
|  |  | TNKS | 0 | 0 |  | 2 |
|  |  | FABP3 | 0 | 0 |  | 2 |
|  |  | CA2 | 0 | 0 |  | 2 |
|  |  | NOX4 | 0 | 0 |  | 2 |
|  |  | MAOA | 0 | 0 |  | 2 |
|  |  | HPSE | 0 | 0 |  | 2 |
|  |  | F10 | 0 | 0 |  | 2 |
|  |  | AMY1A | 0 | 0 |  | 1 |
|  |  | ACHE | 0 | 0 |  | 1 |
|  |  | CES2 | 0 | 0 |  | 1 |
|  |  | HSD11B2 | 0 | 0 |  | 1 |
|  |  | ALOX15 | 0 | 0 |  | 1 |
|  |  | SHBG | 0 | 0 |  | 1 |
|  |  | SLC6A2 | 0 | 0 |  | 1 |
|  |  | PDE10A | 0 | 0 |  | 1 |
|  |  | STS | 0 | 0 |  | 1 |
|  |  | TBXAS1 | 0 | 0 |  | 1 |
|  |  | TYR | 0 | 0 |  | 1 |
|  |  | ENGASE | 0 | 0 |  | 1 |
|  |  | RORA | 0 | 0 |  | 1 |
|  |  | SELL | 0 | 0 |  | 1 |
|  |  | HSD11B1 | 0 | 0 |  | 1 |
|  |  | HSD17B1 | 0 | 0 |  | 1 |
|  |  | TNKS2 | 0 | 0 |  | 1 |
|  |  | XDH | 0 | 0 |  | 1 |
|  |  | CA14 | 0 | 0 |  | 1 |
|  |  | GLRA1 | 0 | 0 |  | 1 |
|  |  | PDE3A | 0 | 0 |  | 1 |
|  |  | PTGES | 0 | 0 |  | 1 |
|  |  | MPO | 0 | 0 |  | 1 |
|  |  | MAOB | 0 | 0 |  | 1 |
|  |  | PTGS1 | 0 | 0 |  | 1 |
|  |  | PDE4A | 0 | 0 |  | 1 |
|  |  | LYPLA2 | 0 | 0 |  | 1 |
|  |  | MMP12 | 0 | 0 |  | 1 |
|  | VMC-related targets | EGFR | 4.928571429 | 1 | Clustered | 26 |
|  |  | SRC | 4.928571429 | 1 | Clustered | 25 |
|  |  | HSP90AA1 | 0 | 0 |  | 25 |
|  |  | STAT3 | 4.928571429 | 1 | Clustered | 21 |
|  |  | MAPK3 | 4.928571429 | 1 | Clustered | 21 |
|  |  | STAT1 | 4.928571429 | 1 | Clustered | 20 |
|  |  | HSPA1A | 0 | 0 |  | 20 |
|  |  | PTK2 | 4.928571429 | 1 | Clustered | 19 |
|  |  | MAPK14 | 0 | 0 |  | 18 |
|  |  | PTPN11 | 4.928571429 | 1 | Clustered | 17 |
|  |  | AKT1 | 4.928571429 | 1 | Seed | 17 |
|  |  | MAPK8 | 4.928571429 | 1 | Clustered | 15 |
|  |  | ERBB2 | 4.928571429 | 1 | Clustered | 14 |
|  |  | PIK3CA | 4.928571429 | 1 | Clustered | 12 |
|  |  | MET | 4.928571429 | 1 | Clustered | 12 |
|  |  | BCL2 | 0 | 0 |  | 12 |
|  |  | HIF1A | 0 | 0 |  | 12 |
|  |  | VEGFA | 4.928571429 | 1 | Clustered | 11 |
|  |  | RPS6KB1 | 0 | 0 |  | 10 |
|  |  | PPARG | 0 | 0 |  | 10 |
|  |  | TNFRSF1A | 4.928571429 | 1 | Clustered | 9 |
|  |  | MTOR | 0 | 0 |  | 9 |
|  |  | PARP1 | 0 | 0 |  | 9 |
|  |  | NOS2 | 0 | 0 |  | 7 |
|  |  | F2 | 0 | 0 |  | 7 |
|  |  | FLT1 | 0 | 0 |  | 7 |
|  |  | PGD | 0 | 0 |  | 7 |
|  |  | PSEN1 | 0 | 0 |  | 6 |
|  |  | CCND1 | 0 | 0 |  | 6 |
|  |  | TUBB1 | 0 | 0 |  | 6 |
|  |  | PPARA | 0 | 0 |  | 6 |
|  |  | PIK3CG | 0 | 0 |  | 5 |
|  |  | PLG | 0 | 0 |  | 5 |
|  |  | PGF | 0 | 0 |  | 5 |
|  |  | ICAM1 | 0 | 0 |  | 4 |
|  |  | VDR | 0 | 0 |  | 4 |
|  |  | SERPINE1 | 0 | 0 |  | 4 |
|  |  | DRD2 | 0 | 0 |  | 3 |
|  |  | CYP51A1 | 0 | 0 |  | 3 |
|  |  | TNNI3 | 0 | 0 |  | 3 |
|  |  | CHRM2 | 0 | 0 |  | 3 |
|  |  | G6PD | 0 | 0 |  | 3 |
|  |  | ARG1 | 0 | 0 |  | 3 |
|  |  | SELE | 0 | 0 |  | 3 |
|  |  | MMP2 | 0 | 0 |  | 3 |
|  |  | SLC6A4 | 0 | 0 |  | 3 |
|  |  | ADRA1D | 0 | 0 |  | 2 |
|  |  | TNNC1 | 0 | 0 |  | 2 |
|  |  | PSEN2 | 0 | 0 |  | 2 |
|  |  | GSR | 0 | 0 |  | 2 |
|  |  | P2RY1 | 0 | 0 |  | 2 |
|  |  | KCNH2 | 0 | 0 |  | 2 |
|  |  | TNNT2 | 0 | 0 |  | 2 |
|  |  | MMP3 | 0 | 0 |  | 2 |
|  |  | SELL | 0 | 0 |  | 1 |
|  |  | ALOX5 | 0 | 0 |  | 1 |
|  |  | CYP2C19 | 0 | 0 |  | 1 |
|  |  | IDO1 | 0 | 0 |  | 1 |
|  |  | ABCB1 | 0 | 0 |  | 1 |
|  |  | ADORA2A | 0 | 0 |  | 1 |
| KS | whole targets | APP | 16.4 | 3 | Clustered | 154 |
|  |  | HSP90AA1 | 7.125 | 4 | Clustered | 137 |
|  |  | HSP90AB1 | 7.125 | 4 | Clustered | 125 |
|  |  | SRC | 5.8 | 1 | Clustered | 119 |
|  |  | EGFR | 5.8 | 1 | Clustered | 115 |
|  |  | MAPK1 | 7.125 | 4 | Clustered | 105 |
|  |  | PRKCA | 7.125 | 4 | Clustered | 102 |
|  |  | GSK3B | 5.8 | 1 | Clustered | 96 |
|  |  | MAPK3 | 7.125 | 4 | Clustered | 94 |
|  |  | AKT1 | 7.125 | 4 | Clustered | 89 |
|  |  | CDK2 | 7.125 | 4 | Clustered | 89 |
|  |  | CDK1 | 5.8 | 1 | Clustered | 88 |
|  |  | PRKACA | 7.125 | 4 | Clustered | 83 |
|  |  | NTRK1 | 3.022222222 | 2 | Clustered | 83 |
|  |  | HNF4A | 3.022222222 | 2 | Clustered | 79 |
|  |  | ESR1 | 7.125 | 4 | Clustered | 74 |
|  |  | RELA | 5.8 | 1 | Clustered | 72 |
|  |  | VCP | 7.125 | 4 | Clustered | 68 |
|  |  | MDM2 | 7.125 | 4 | Clustered | 68 |
|  |  | EP300 | 5.8 | 1 | Clustered | 67 |
|  |  | PIK3R1 | 7.125 | 4 | Clustered | 64 |
|  |  | PTK2 | 7.125 | 4 | Clustered | 63 |
|  |  | RPS6KA3 | 5.8 | 1 | Clustered | 60 |
|  |  | ERBB2 | 7.125 | 4 | Clustered | 59 |
|  |  | CSNK2A1 | 7.125 | 4 | Clustered | 58 |
|  |  | AR | 5.8 | 1 | Clustered | 54 |
|  |  | ABL1 | 5.8 | 1 | Clustered | 54 |
|  |  | MAPT | 7.125 | 4 | Seed | 51 |
|  |  | PRKDC | 7.125 | 4 | Clustered | 51 |
|  |  | ESR2 | 5.8 | 1 | Clustered | 51 |
|  |  | CDK5 | 3.022222222 | 2 | Clustered | 51 |
|  |  | APEX1 | 3.022222222 | 2 | Clustered | 50 |
|  |  | STAT1 | 3.022222222 | 2 | Clustered | 49 |
|  |  | PLK1 | 3.022222222 | 2 | Clustered | 49 |
|  |  | SYK | 5.8 | 1 | Clustered | 48 |
|  |  | RAF1 | 5.8 | 1 | Clustered | 48 |
|  |  | PCNA | 3.022222222 | 2 | Clustered | 47 |
|  |  | ADRB2 | 7 | 6 | Clustered | 45 |
|  |  | INSR | 5.8 | 1 | Clustered | 45 |
|  |  | SIRT1 | 3.022222222 | 2 | Clustered | 45 |
|  |  | PARP1 | 5.8 | 1 | Clustered | 44 |
|  |  | OPRM1 | 16.4 | 3 | Clustered | 43 |
|  |  | RPS6KB1 | 7.125 | 4 | Clustered | 43 |
|  |  | IGF1R | 5.8 | 1 | Clustered | 43 |
|  |  | YWHAG | 5.8 | 1 | Clustered | 43 |
|  |  | HSP90B1 | 5.8 | 1 | Seed | 42 |
|  |  | HDAC1 | 3.022222222 | 2 | Clustered | 42 |
|  |  | HDAC2 | 3.022222222 | 2 | Clustered | 41 |
|  |  | PTGER3 | 16.4 | 3 | Clustered | 40 |
|  |  | CHRM2 | 16.4 | 3 | Clustered | 40 |
|  |  | DRD2 | 16.4 | 3 | Clustered | 40 |
|  |  | CHEK1 | 7.125 | 4 | Clustered | 40 |
|  |  | AURKA | 5.8 | 1 | Clustered | 40 |
|  |  | CDK4 | 5.8 | 1 | Clustered | 40 |
|  |  | TNF | 3.022222222 | 2 | Clustered | 40 |
|  |  | S1PR1 | 16.4 | 3 | Clustered | 39 |
|  |  | OPRD1 | 16.4 | 3 | Clustered | 39 |
|  |  | GCGR | 10.86956522 | 5 | Clustered | 39 |
|  |  | HDAC6 | 7.125 | 4 | Clustered | 39 |
|  |  | PIK3CA | 5.8 | 1 | Clustered | 39 |
|  |  | CNR1 | 16.4 | 3 | Clustered | 38 |
|  |  | ADRA2A | 16.4 | 3 | Clustered | 38 |
|  |  | DRD4 | 16.4 | 3 | Clustered | 38 |
|  |  | ADORA1 | 16.4 | 3 | Clustered | 38 |
|  |  | VEGFA | 5.8 | 1 | Clustered | 38 |
|  |  | PPARG | 7.125 | 4 | Clustered | 37 |
|  |  | LCK | 5.8 | 1 | Clustered | 37 |
|  |  | MTOR | 5.8 | 1 | Clustered | 37 |
|  |  | NR3C1 | 5.8 | 1 | Clustered | 37 |
|  |  | EZR | 3.022222222 | 2 | Clustered | 37 |
|  |  | HTR1B | 16.4 | 3 | Clustered | 36 |
|  |  | PTPN1 | 5.8 | 1 | Clustered | 36 |
|  |  | CCR4 | 16.4 | 3 | Clustered | 35 |
|  |  | DRD3 | 16.4 | 3 | Clustered | 35 |
|  |  | GRM6 | 16.4 | 3 | Clustered | 35 |
|  |  | CHRM4 | 16.4 | 3 | Clustered | 35 |
|  |  | HTR1A | 16.4 | 3 | Clustered | 35 |
|  |  | CNR2 | 16.4 | 3 | Clustered | 35 |
|  |  | GRM8 | 16.4 | 3 | Clustered | 35 |
|  |  | CXCR1 | 16.4 | 3 | Clustered | 35 |
|  |  | GRM1 | 10.86956522 | 5 | Clustered | 35 |
|  |  | TUBB3 | 5.8 | 1 | Clustered | 35 |
|  |  | ADRA2B | 16.4 | 3 | Clustered | 34 |
|  |  | OPRL1 | 16.4 | 3 | Clustered | 34 |
|  |  | HTR5A | 16.4 | 3 | Clustered | 34 |
|  |  | ADORA3 | 16.4 | 3 | Clustered | 34 |
|  |  | HTR1D | 16.4 | 3 | Clustered | 34 |
|  |  | ADRA2C | 16.4 | 3 | Clustered | 34 |
|  |  | HTR1F | 16.4 | 3 | Clustered | 34 |
|  |  | OPRK1 | 16.4 | 3 | Clustered | 34 |
|  |  | RXRA | 5.8 | 1 | Clustered | 34 |
|  |  | HDAC3 | 3.022222222 | 2 | Clustered | 34 |
|  |  | CCND1 | 3.022222222 | 2 | Clustered | 34 |
|  |  | SNCA | 5.8 | 1 | Clustered | 33 |
|  |  | PDPK1 | 3.022222222 | 2 | Clustered | 33 |
|  |  | CFTR | 0 | 0 |  | 33 |
|  |  | HDAC4 | 3.022222222 | 2 | Clustered | 32 |
|  |  | GRK2 | 3.022222222 | 2 | Seed | 32 |
|  |  | HDAC5 | 0 | 0 |  | 32 |
|  |  | F2 | 10.86956522 | 5 | Clustered | 31 |
|  |  | GRM5 | 10.86956522 | 5 | Clustered | 31 |
|  |  | RPS6KA1 | 7.125 | 4 | Clustered | 31 |
|  |  | MET | 7.125 | 4 | Clustered | 31 |
|  |  | CHEK2 | 5.8 | 1 | Clustered | 31 |
|  |  | NCOR2 | 5.8 | 1 | Clustered | 31 |
|  |  | ALDH2 | 3.022222222 | 2 | Clustered | 31 |
|  |  | CCNB1 | 1.4 | 9 | Clustered | 31 |
|  |  | GRM2 | 16.4 | 3 | Clustered | 30 |
|  |  | HRH3 | 16.4 | 3 | Clustered | 30 |
|  |  | GRM3 | 16.4 | 3 | Clustered | 30 |
|  |  | PGD | 5.8 | 1 | Clustered | 30 |
|  |  | PDGFRB | 5.8 | 1 | Clustered | 30 |
|  |  | BAD | 0 | 0 |  | 30 |
|  |  | IKBKB | 0 | 0 |  | 30 |
|  |  | GRM4 | 16.4 | 3 | Clustered | 29 |
|  |  | S1PR3 | 16.4 | 3 | Clustered | 29 |
|  |  | AGTR1 | 10.86956522 | 5 | Clustered | 29 |
|  |  | PIK3CB | 5.8 | 1 | Clustered | 29 |
|  |  | FGFR1 | 5.8 | 1 | Clustered | 29 |
|  |  | TAS2R31 | 16.4 | 3 | Seed | 28 |
|  |  | HRH4 | 16.4 | 3 | Clustered | 28 |
|  |  | HCK | 5.8 | 1 | Clustered | 28 |
|  |  | BCL2 | 3.022222222 | 2 | Clustered | 28 |
|  |  | ADRA1B | 10.86956522 | 5 | Clustered | 27 |
|  |  | HTR2A | 10.86956522 | 5 | Clustered | 27 |
|  |  | CHRM3 | 10.86956522 | 5 | Clustered | 27 |
|  |  | HRH1 | 10.86956522 | 5 | Clustered | 27 |
|  |  | JAK1 | 5.8 | 1 | Clustered | 27 |
|  |  | CDK6 | 3.022222222 | 2 | Clustered | 27 |
|  |  | SGK1 | 3.022222222 | 2 | Clustered | 27 |
|  |  | EDNRA | 10.86956522 | 5 | Clustered | 26 |
|  |  | PTGER1 | 10.86956522 | 5 | Clustered | 26 |
|  |  | HTR2B | 10.86956522 | 5 | Clustered | 26 |
|  |  | PDGFRA | 5.8 | 1 | Clustered | 26 |
|  |  | KIT | 1.25 | 10 | Clustered | 26 |
|  |  | CCKBR | 10.86956522 | 5 | Clustered | 25 |
|  |  | KDR | 3.022222222 | 2 | Clustered | 25 |
|  |  | DYRK1A | 1 | 11 | Seed | 25 |
|  |  | CCND2 | 0 | 0 |  | 25 |
|  |  | CCNA2 | 0 | 0 |  | 25 |
|  |  | TBK1 | 0 | 0 |  | 25 |
|  |  | MAP3K7 | 0 | 0 |  | 25 |
|  |  | PTAFR | 10.86956522 | 5 | Clustered | 24 |
|  |  | CHRM1 | 10.86956522 | 5 | Clustered | 24 |
|  |  | HTR2C | 10.86956522 | 5 | Clustered | 24 |
|  |  | RET | 7.125 | 4 | Clustered | 24 |
|  |  | AVPR2 | 7 | 6 | Clustered | 24 |
|  |  | GRIN2B | 3.022222222 | 2 | Clustered | 24 |
|  |  | TUBB1 | 3.022222222 | 2 | Clustered | 24 |
|  |  | CCNE1 | 3.022222222 | 2 | Clustered | 24 |
|  |  | EIF4A1 | 0 | 0 |  | 24 |
|  |  | DNMT1 | 0 | 0 |  | 24 |
|  |  | FFAR1 | 10.86956522 | 5 | Clustered | 23 |
|  |  | ADRA1A | 10.86956522 | 5 | Clustered | 23 |
|  |  | CHRM5 | 10.86956522 | 5 | Clustered | 23 |
|  |  | ADRA1D | 10.86956522 | 5 | Clustered | 23 |
|  |  | TACR1 | 10.86956522 | 5 | Clustered | 23 |
|  |  | TOP1 | 3.022222222 | 2 | Clustered | 23 |
|  |  | MAPKAPK2 | 0 | 0 |  | 23 |
|  |  | ITGB3 | 0 | 0 |  | 23 |
|  |  | DRD1 | 7 | 6 | Clustered | 22 |
|  |  | PYGL | 5.8 | 1 | Clustered | 22 |
|  |  | BRAF | 3.022222222 | 2 | Clustered | 22 |
|  |  | PDK1 | 0 | 0 |  | 22 |
|  |  | KDM1A | 0 | 0 |  | 22 |
|  |  | MAPK10 | 0 | 0 |  | 22 |
|  |  | KISS1R | 10.86956522 | 5 | Clustered | 21 |
|  |  | UTS2R | 10.86956522 | 5 | Seed | 21 |
|  |  | ADORA2A | 7 | 6 | Clustered | 21 |
|  |  | TOP2A | 3.022222222 | 2 | Clustered | 21 |
|  |  | PGF | 3.022222222 | 2 | Clustered | 21 |
|  |  | BCL2L1 | 0 | 0 |  | 21 |
|  |  | MCL1 | 0 | 0 |  | 21 |
|  |  | HTR7 | 7 | 6 | Clustered | 20 |
|  |  | ADRB1 | 7 | 6 | Clustered | 20 |
|  |  | IMPDH2 | 3.022222222 | 2 | Clustered | 20 |
|  |  | CDK3 | 3.022222222 | 2 | Clustered | 20 |
|  |  | RPS6KA5 | 3.022222222 | 2 | Clustered | 20 |
|  |  | FEN1 | 3.022222222 | 2 | Clustered | 19 |
|  |  | AKR1B1 | 0 | 0 |  | 19 |
|  |  | TERT | 0 | 0 |  | 19 |
|  |  | PTGER2 | 7 | 6 | Clustered | 18 |
|  |  | PTGER4 | 7 | 6 | Clustered | 18 |
|  |  | HTR6 | 7 | 6 | Clustered | 18 |
|  |  | AKR1B10 | 5.8 | 1 | Clustered | 18 |
|  |  | FKBP1A | 1.4 | 9 | Clustered | 18 |
|  |  | GRIN1 | 0 | 0 |  | 18 |
|  |  | ROCK1 | 0 | 0 |  | 18 |
|  |  | DRD5 | 7 | 6 | Clustered | 17 |
|  |  | PIK3CG | 3.022222222 | 2 | Clustered | 17 |
|  |  | PIM1 | 3.022222222 | 2 | Clustered | 17 |
|  |  | AURKB | 3.022222222 | 2 | Clustered | 17 |
|  |  | CCNB2 | 1.4 | 9 | Clustered | 17 |
|  |  | PGR | 0 | 0 |  | 17 |
|  |  | MAP3K8 | 0 | 0 |  | 17 |
|  |  | XIAP | 0 | 0 |  | 17 |
|  |  | CTSD | 0 | 0 |  | 17 |
|  |  | ADORA2B | 7 | 6 | Clustered | 16 |
|  |  | ADRB3 | 7 | 6 | Clustered | 16 |
|  |  | CRHR1 | 7 | 6 | Clustered | 16 |
|  |  | CCND3 | 5.8 | 1 | Clustered | 16 |
|  |  | AXL | 5.8 | 1 | Clustered | 16 |
|  |  | NEK2 | 3.022222222 | 2 | Clustered | 16 |
|  |  | CDC7 | 3.022222222 | 2 | Clustered | 16 |
|  |  | JAK3 | 1.25 | 10 | Clustered | 16 |
|  |  | PPARA | 0 | 0 |  | 16 |
|  |  | STAT6 | 0 | 0 |  | 16 |
|  |  | CDK5R1 | 0 | 0 |  | 16 |
|  |  | HRH2 | 7 | 6 | Clustered | 15 |
|  |  | GPBAR1 | 7 | 6 | Seed | 15 |
|  |  | DAPK1 | 3.022222222 | 2 | Clustered | 15 |
|  |  | HDAC7 | 3.022222222 | 2 | Clustered | 15 |
|  |  | PKN1 | 3.022222222 | 2 | Clustered | 15 |
|  |  | ALK | 0 | 0 |  | 15 |
|  |  | BIRC2 | 0 | 0 |  | 15 |
|  |  | PAK4 | 0 | 0 |  | 15 |
|  |  | ROCK2 | 0 | 0 |  | 15 |
|  |  | CTSB | 0 | 0 |  | 15 |
|  |  | AKR1C4 | 5.8 | 1 | Clustered | 14 |
|  |  | AKR1C2 | 5.8 | 1 | Clustered | 14 |
|  |  | AKR1A1 | 5.8 | 1 | Clustered | 14 |
|  |  | FLT3 | 3.022222222 | 2 | Clustered | 14 |
|  |  | FLT4 | 3.022222222 | 2 | Clustered | 14 |
|  |  | TAB1 | 0 | 0 |  | 14 |
|  |  | DNM1 | 0 | 0 |  | 14 |
|  |  | GSTP1 | 0 | 0 |  | 14 |
|  |  | PTK6 | 0 | 0 |  | 14 |
|  |  | NOS2 | 0 | 0 |  | 14 |
|  |  | MYLK | 0 | 0 |  | 14 |
|  |  | PDE4D | 0 | 0 |  | 14 |
|  |  | WEE1 | 0 | 0 |  | 14 |
|  |  | PIK3CD | 1.25 | 10 | Clustered | 13 |
|  |  | MAP3K14 | 0 | 0 |  | 13 |
|  |  | EEF2K | 0 | 0 |  | 13 |
|  |  | ADAM17 | 0 | 0 |  | 13 |
|  |  | AKR1C1 | 5.8 | 1 | Clustered | 12 |
|  |  | PLAA | 3.022222222 | 2 | Clustered | 12 |
|  |  | CCNA1 | 0 | 0 |  | 12 |
|  |  | AHR | 0 | 0 |  | 12 |
|  |  | ITK | 0 | 0 |  | 12 |
|  |  | SIRT2 | 0 | 0 |  | 12 |
|  |  | NR1I2 | 0 | 0 |  | 12 |
|  |  | HDAC9 | 0 | 0 |  | 12 |
|  |  | CCNE2 | 0 | 0 |  | 12 |
|  |  | IMPDH1 | 3.022222222 | 2 | Clustered | 11 |
|  |  | CHRNA7 | 2.571428571 | 7 | Clustered | 11 |
|  |  | IL2 | 1.25 | 10 | Clustered | 11 |
|  |  | MMP2 | 0 | 0 |  | 11 |
|  |  | MIF | 0 | 0 |  | 11 |
|  |  | ITGA2B | 0 | 0 |  | 11 |
|  |  | GABRG2 | 0 | 0 |  | 11 |
|  |  | ALOX5 | 0 | 0 |  | 11 |
|  |  | PTGS2 | 0 | 0 |  | 11 |
|  |  | ESRRB | 0 | 0 |  | 11 |
|  |  | MME | 0 | 0 |  | 11 |
|  |  | SPHK2 | 1.4 | 9 | Clustered | 10 |
|  |  | MKNK1 | 0 | 0 |  | 10 |
|  |  | BRD4 | 0 | 0 |  | 10 |
|  |  | DYRK1B | 0 | 0 |  | 10 |
|  |  | POLB | 0 | 0 |  | 10 |
|  |  | NR1H4 | 0 | 0 |  | 10 |
|  |  | FLT1 | 0 | 0 |  | 10 |
|  |  | CAMK2B | 0 | 0 |  | 10 |
|  |  | TLR9 | 0 | 0 |  | 10 |
|  |  | CHRNB4 | 2.571428571 | 7 | Clustered | 9 |
|  |  | CYP51A1 | 2.333333333 | 8 | Clustered | 9 |
|  |  | PFKFB3 | 0 | 0 |  | 9 |
|  |  | DUSP3 | 0 | 0 |  | 9 |
|  |  | PIM2 | 0 | 0 |  | 9 |
|  |  | LAP3 | 0 | 0 |  | 9 |
|  |  | ABCB1 | 0 | 0 |  | 9 |
|  |  | EPHB4 | 0 | 0 |  | 9 |
|  |  | CA9 | 0 | 0 |  | 9 |
|  |  | MPG | 0 | 0 |  | 9 |
|  |  | SREBF2 | 0 | 0 |  | 9 |
|  |  | CLK1 | 0 | 0 |  | 9 |
|  |  | ROS1 | 0 | 0 |  | 9 |
|  |  | PLG | 0 | 0 |  | 9 |
|  |  | ESRRA | 0 | 0 |  | 9 |
|  |  | CTSL | 0 | 0 |  | 9 |
|  |  | H1-0 | 0 | 0 |  | 9 |
|  |  | CHRNA3 | 2.571428571 | 7 | Clustered | 8 |
|  |  | CHRNA4 | 2.571428571 | 7 | Clustered | 8 |
|  |  | CYP1B1 | 2.333333333 | 8 | Seed | 8 |
|  |  | LTA4H | 0 | 0 |  | 8 |
|  |  | ARG1 | 0 | 0 |  | 8 |
|  |  | GABRA1 | 0 | 0 |  | 8 |
|  |  | SPHK1 | 0 | 0 |  | 8 |
|  |  | SERPINE1 | 0 | 0 |  | 8 |
|  |  | GRK6 | 0 | 0 |  | 8 |
|  |  | TRPV1 | 0 | 0 |  | 8 |
|  |  | SORD | 0 | 0 |  | 8 |
|  |  | MMP1 | 0 | 0 |  | 8 |
|  |  | ERCC5 | 0 | 0 |  | 8 |
|  |  | MERTK | 0 | 0 |  | 8 |
|  |  | CHRNB2 | 2.571428571 | 7 | Clustered | 7 |
|  |  | PDE4B | 0 | 0 |  | 7 |
|  |  | MMP7 | 0 | 0 |  | 7 |
|  |  | TNNI3 | 0 | 0 |  | 7 |
|  |  | GABRA3 | 0 | 0 |  | 7 |
|  |  | COMT | 0 | 0 |  | 7 |
|  |  | NR3C2 | 0 | 0 |  | 7 |
|  |  | GBA | 0 | 0 |  | 7 |
|  |  | GLO1 | 0 | 0 |  | 7 |
|  |  | PDE3B | 0 | 0 |  | 7 |
|  |  | CHRND | 0 | 0 |  | 7 |
|  |  | GABRB3 | 0 | 0 |  | 7 |
|  |  | AKR1C3 | 0 | 0 |  | 7 |
|  |  | KCNH2 | 0 | 0 |  | 7 |
|  |  | PI4KB | 0 | 0 |  | 7 |
|  |  | CHRNA5 | 2.571428571 | 7 | Clustered | 6 |
|  |  | CYP19A1 | 2.333333333 | 8 | Clustered | 6 |
|  |  | CYP2D6 | 2.333333333 | 8 | Clustered | 6 |
|  |  | CCNB3 | 1.4 | 9 | Clustered | 6 |
|  |  | TTR | 0 | 0 |  | 6 |
|  |  | SLC6A3 | 0 | 0 |  | 6 |
|  |  | TXK | 0 | 0 |  | 6 |
|  |  | NUAK1 | 0 | 0 |  | 6 |
|  |  | NEK6 | 0 | 0 |  | 6 |
|  |  | MMP3 | 0 | 0 |  | 6 |
|  |  | HTR3A | 0 | 0 |  | 6 |
|  |  | PIM3 | 0 | 0 |  | 6 |
|  |  | MMP14 | 0 | 0 |  | 6 |
|  |  | TNKS | 0 | 0 |  | 6 |
|  |  | TNNT2 | 0 | 0 |  | 6 |
|  |  | CSNK1G1 | 0 | 0 |  | 6 |
|  |  | ABCC1 | 0 | 0 |  | 6 |
|  |  | EGLN1 | 0 | 0 |  | 6 |
|  |  | EPHX1 | 0 | 0 |  | 6 |
|  |  | CYP2C19 | 2.333333333 | 8 | Clustered | 5 |
|  |  | CYP2C9 | 2.333333333 | 8 | Clustered | 5 |
|  |  | LNPEP | 0 | 0 |  | 5 |
|  |  | ERN1 | 0 | 0 |  | 5 |
|  |  | PLA2G2A | 0 | 0 |  | 5 |
|  |  | NAE1 | 0 | 0 |  | 5 |
|  |  | HDAC8 | 0 | 0 |  | 5 |
|  |  | PLA2G10 | 0 | 0 |  | 5 |
|  |  | HSD17B7 | 0 | 0 |  | 5 |
|  |  | KCNA5 | 0 | 0 |  | 5 |
|  |  | MMP9 | 0 | 0 |  | 5 |
|  |  | SLC6A4 | 0 | 0 |  | 5 |
|  |  | BACE1 | 0 | 0 |  | 5 |
|  |  | MMP13 | 0 | 0 |  | 5 |
|  |  | GABRA5 | 0 | 0 |  | 5 |
|  |  | PARP2 | 0 | 0 |  | 5 |
|  |  | ATP4A | 0 | 0 |  | 5 |
|  |  | PRMT3 | 0 | 0 |  | 5 |
|  |  | CBR1 | 0 | 0 |  | 5 |
|  |  | CHRNA2 | 2.571428571 | 7 | Seed | 4 |
|  |  | FNTB | 1 | 11 | Clustered | 4 |
|  |  | FNTA | 1 | 11 | Clustered | 4 |
|  |  | F3 | 0 | 0 |  | 4 |
|  |  | HSD17B3 | 0 | 0 |  | 4 |
|  |  | DPP4 | 0 | 0 |  | 4 |
|  |  | CTSK | 0 | 0 |  | 4 |
|  |  | ALPL | 0 | 0 |  | 4 |
|  |  | F10 | 0 | 0 |  | 4 |
|  |  | PNP | 0 | 0 |  | 4 |
|  |  | DCTPP1 | 0 | 0 |  | 4 |
|  |  | MAP3K12 | 0 | 0 |  | 4 |
|  |  | PTPRS | 0 | 0 |  | 4 |
|  |  | KLK2 | 0 | 0 |  | 4 |
|  |  | DHFR | 0 | 0 |  | 4 |
|  |  | GABRA6 | 0 | 0 |  | 4 |
|  |  | DPP9 | 0 | 0 |  | 4 |
|  |  | SIGMAR1 | 0 | 0 |  | 4 |
|  |  | CHRNG | 0 | 0 |  | 4 |
|  |  | SLC47A1 | 0 | 0 |  | 4 |
|  |  | ADAMTS4 | 0 | 0 |  | 4 |
|  |  | ABCG2 | 0 | 0 |  | 4 |
|  |  | KCNJ1 | 0 | 0 |  | 4 |
|  |  | TNNC1 | 0 | 0 |  | 3 |
|  |  | DBF4 | 0 | 0 |  | 3 |
|  |  | SHBG | 0 | 0 |  | 3 |
|  |  | HDAC11 | 0 | 0 |  | 3 |
|  |  | TDP1 | 0 | 0 |  | 3 |
|  |  | GUSB | 0 | 0 |  | 3 |
|  |  | CA2 | 0 | 0 |  | 3 |
|  |  | HPGDS | 0 | 0 |  | 3 |
|  |  | CHRNA1 | 0 | 0 |  | 3 |
|  |  | SCARB1 | 0 | 0 |  | 3 |
|  |  | ACVRL1 | 0 | 0 |  | 3 |
|  |  | CHRNA6 | 0 | 0 |  | 3 |
|  |  | HSD17B14 | 0 | 0 |  | 3 |
|  |  | RNPEP | 0 | 0 |  | 3 |
|  |  | TYMS | 0 | 0 |  | 3 |
|  |  | HSD11B1 | 0 | 0 |  | 3 |
|  |  | ANPEP | 0 | 0 |  | 3 |
|  |  | HDAC10 | 0 | 0 |  | 3 |
|  |  | PDE3A | 0 | 0 |  | 3 |
|  |  | MAOA | 0 | 0 |  | 3 |
|  |  | ADAMTS1 | 0 | 0 |  | 3 |
|  |  | TRPM8 | 0 | 0 |  | 3 |
|  |  | ELANE | 0 | 0 |  | 3 |
|  |  | XDH | 0 | 0 |  | 3 |
|  |  | TNKS2 | 0 | 0 |  | 3 |
|  |  | CHRNB3 | 0 | 0 |  | 3 |
|  |  | SLC18A2 | 0 | 0 |  | 3 |
|  |  | PAOX | 0 | 0 |  | 2 |
|  |  | NAAA | 0 | 0 |  | 2 |
|  |  | TYMP | 0 | 0 |  | 2 |
|  |  | TBXAS1 | 0 | 0 |  | 2 |
|  |  | CA3 | 0 | 0 |  | 2 |
|  |  | ALOX15B | 0 | 0 |  | 2 |
|  |  | METAP1 | 0 | 0 |  | 2 |
|  |  | ADCY5 | 0 | 0 |  | 2 |
|  |  | ST3GAL3 | 0 | 0 |  | 2 |
|  |  | TRPV3 | 0 | 0 |  | 2 |
|  |  | CISD1 | 0 | 0 |  | 2 |
|  |  | MPO | 0 | 0 |  | 2 |
|  |  | MAOB | 0 | 0 |  | 2 |
|  |  | TNNI3K | 0 | 0 |  | 2 |
|  |  | ITGAL | 0 | 0 |  | 2 |
|  |  | AMY1A | 0 | 0 |  | 2 |
|  |  | ALOX5AP | 0 | 0 |  | 2 |
|  |  | HSD11B2 | 0 | 0 |  | 2 |
|  |  | CES2 | 0 | 0 |  | 2 |
|  |  | SCN5A | 0 | 0 |  | 2 |
|  |  | QDPR | 0 | 0 |  | 2 |
|  |  | EPHX2 | 0 | 0 |  | 2 |
|  |  | ALOX15 | 0 | 0 |  | 2 |
|  |  | PDE10A | 0 | 0 |  | 2 |
|  |  | CHRNB1 | 0 | 0 |  | 2 |
|  |  | REN | 0 | 0 |  | 2 |
|  |  | MTAP | 0 | 0 |  | 2 |
|  |  | CA14 | 0 | 0 |  | 2 |
|  |  | ECE1 | 0 | 0 |  | 2 |
|  |  | PDE11A | 0 | 0 |  | 2 |
|  |  | GSTM2 | 0 | 0 |  | 1 |
|  |  | SLC6A2 | 0 | 0 |  | 1 |
|  |  | KCNK2 | 0 | 0 |  | 1 |
|  |  | STS | 0 | 0 |  | 1 |
|  |  | HPGD | 0 | 0 |  | 1 |
|  |  | MMP16 | 0 | 0 |  | 1 |
|  |  | PDE5A | 0 | 0 |  | 1 |
|  |  | ATP4B | 0 | 0 |  | 1 |
|  |  | FUCA1 | 0 | 0 |  | 1 |
|  |  | HSD17B2 | 0 | 0 |  | 1 |
|  |  | HSD17B1 | 0 | 0 |  | 1 |
|  |  | CD38 | 0 | 0 |  | 1 |
|  |  | CMA1 | 0 | 0 |  | 1 |
|  |  | MMP26 | 0 | 0 |  | 1 |
|  |  | SLC6A9 | 0 | 0 |  | 1 |
|  |  | ALOX12 | 0 | 0 |  | 1 |
|  |  | CTSS | 0 | 0 |  | 1 |
|  |  | ADCY1 | 0 | 0 |  | 1 |
|  |  | FAP | 0 | 0 |  | 1 |
|  |  | CTSC | 0 | 0 |  | 1 |
|  |  | FAAH | 0 | 0 |  | 1 |
|  |  | HTR3B | 0 | 0 |  | 1 |
|  |  | ODC1 | 0 | 0 |  | 1 |
|  |  | PTGES | 0 | 0 |  | 1 |
|  |  | NOX1 | 0 | 0 |  | 1 |
|  |  | ACHE | 0 | 0 |  | 1 |
|  |  | CA1 | 0 | 0 |  | 1 |
|  |  | PNMT | 0 | 0 |  | 1 |
|  |  | PON1 | 0 | 0 |  | 1 |
|  |  | DGAT1 | 0 | 0 |  | 1 |
|  |  | TSPO | 0 | 0 |  | 1 |
|  |  | DNPEP | 0 | 0 |  | 1 |
|  |  | WNT3A | 0 | 0 |  | 1 |
|  |  | CES1 | 0 | 0 |  | 1 |
|  |  | CA5A | 0 | 0 |  | 1 |
|  |  | ADH1A | 0 | 0 |  | 1 |
|  |  | ADH1C | 0 | 0 |  | 1 |
|  |  | GBA2 | 0 | 0 |  | 1 |
|  |  | FCER2 | 0 | 0 |  | 1 |
|  |  | DAO | 0 | 0 |  | 1 |
|  |  | PLA2G1B | 0 | 0 |  | 1 |
|  |  | PTGS1 | 0 | 0 |  | 1 |
|  |  | UGCG | 0 | 0 |  | 1 |
|  |  | MMP12 | 0 | 0 |  | 1 |
|  | VMC-related targets | SRC | 5.5 | 1 | Clustered | 39 |
|  |  | HSP90AA1 | 5.5 | 1 | Clustered | 38 |
|  |  | PRKCA | 5.5 | 1 | Clustered | 35 |
|  |  | EGFR | 5.5 | 1 | Seed | 33 |
|  |  | MAPK1 | 5.5 | 1 | Clustered | 32 |
|  |  | MAPK3 | 5.5 | 1 | Clustered | 29 |
|  |  | RELA | 5.5 | 1 | Clustered | 27 |
|  |  | ERBB2 | 5.5 | 1 | Clustered | 23 |
|  |  | AKT1 | 5.5 | 1 | Clustered | 23 |
|  |  | PTK2 | 5.5 | 1 | Clustered | 23 |
|  |  | STAT1 | 1.2 | 3 | Clustered | 23 |
|  |  | PRKACA | 0 | 0 |  | 23 |
|  |  | NTRK1 | 5.5 | 1 | Clustered | 22 |
|  |  | LCK | 1.2 | 3 | Seed | 18 |
|  |  | RAF1 | 5.5 | 1 | Clustered | 16 |
|  |  | RET | 5.5 | 1 | Clustered | 16 |
|  |  | PIK3CA | 1.2 | 3 | Clustered | 16 |
|  |  | ABL1 | 2.111111111 | 2 | Seed | 15 |
|  |  | MET | 0 | 0 |  | 15 |
|  |  | VEGFA | 5.5 | 1 | Clustered | 14 |
|  |  | TNF | 1.2 | 3 | Clustered | 14 |
|  |  | PPARG | 1.2 | 3 | Clustered | 13 |
|  |  | PARP1 | 2.111111111 | 2 | Clustered | 12 |
|  |  | ADRB2 | 0 | 0 |  | 11 |
|  |  | BCL2 | 0 | 0 |  | 11 |
|  |  | F2 | 2.111111111 | 2 | Clustered | 10 |
|  |  | RPS6KB1 | 0 | 0 |  | 10 |
|  |  | ITGB3 | 0 | 0 |  | 10 |
|  |  | IKBKB | 0 | 0 |  | 10 |
|  |  | MTOR | 0 | 0 |  | 10 |
|  |  | KIT | 0 | 0 |  | 10 |
|  |  | AGTR1 | 2.111111111 | 2 | Clustered | 9 |
|  |  | PCNA | 2.111111111 | 2 | Clustered | 9 |
|  |  | HRH1 | 2.111111111 | 2 | Clustered | 8 |
|  |  | PPARA | 0 | 0 |  | 8 |
|  |  | PGF | 0 | 0 |  | 8 |
|  |  | PIK3CG | 0 | 0 |  | 8 |
|  |  | ADAM17 | 0 | 0 |  | 7 |
|  |  | NOS2 | 0 | 0 |  | 7 |
|  |  | CCND1 | 0 | 0 |  | 7 |
|  |  | PGD | 0 | 0 |  | 7 |
|  |  | FKBP1A | 0 | 0 |  | 7 |
|  |  | MMP1 | 0 | 0 |  | 7 |
|  |  | EDNRA | 2.111111111 | 2 | Clustered | 6 |
|  |  | ADORA2A | 1.5 | 4 | Clustered | 6 |
|  |  | ITGA2B | 0 | 0 |  | 6 |
|  |  | RPS6KA5 | 0 | 0 |  | 6 |
|  |  | TUBB1 | 0 | 0 |  | 6 |
|  |  | ITK | 0 | 0 |  | 6 |
|  |  | PLG | 0 | 0 |  | 6 |
|  |  | TACR1 | 2.111111111 | 2 | Clustered | 5 |
|  |  | ADRA1D | 2.111111111 | 2 | Clustered | 5 |
|  |  | ADRB3 | 1.5 | 4 | Clustered | 5 |
|  |  | ADRB1 | 1.5 | 4 | Clustered | 5 |
|  |  | S1PR1 | 0 | 0 |  | 5 |
|  |  | FLT1 | 0 | 0 |  | 5 |
|  |  | IL2 | 0 | 0 |  | 5 |
|  |  | MMP2 | 0 | 0 |  | 5 |
|  |  | TNNI3 | 0 | 0 |  | 5 |
|  |  | HRH2 | 1.5 | 4 | Seed | 4 |
|  |  | SERPINE1 | 0 | 0 |  | 4 |
|  |  | ALOX5 | 0 | 0 |  | 4 |
|  |  | TNNT2 | 0 | 0 |  | 4 |
|  |  | KCNH2 | 0 | 0 |  | 4 |
|  |  | DRD2 | 0 | 0 |  | 4 |
|  |  | CHRM2 | 0 | 0 |  | 4 |
|  |  | MIF | 0 | 0 |  | 4 |
|  |  | ABCB1 | 0 | 0 |  | 3 |
|  |  | ARG1 | 0 | 0 |  | 3 |
|  |  | MMP9 | 0 | 0 |  | 3 |
|  |  | SLC6A4 | 0 | 0 |  | 3 |
|  |  | ELANE | 0 | 0 |  | 3 |
|  |  | TLR9 | 0 | 0 |  | 3 |
|  |  | TNNC1 | 0 | 0 |  | 2 |
|  |  | ACVRL1 | 0 | 0 |  | 2 |
|  |  | F3 | 0 | 0 |  | 2 |
|  |  | CTSL | 0 | 0 |  | 2 |
|  |  | NR3C2 | 0 | 0 |  | 2 |
|  |  | ADCY5 | 0 | 0 |  | 2 |
|  |  | MMP3 | 0 | 0 |  | 2 |
|  |  | ABCC1 | 0 | 0 |  | 1 |
|  |  | ADCY1 | 0 | 0 |  | 1 |
|  |  | CYP2C19 | 0 | 0 |  | 1 |
|  |  | CMA1 | 0 | 0 |  | 1 |
|  |  | MPO | 0 | 0 |  | 1 |
|  |  | CYP51A1 | 0 | 0 |  | 1 |
|  |  | ITGAL | 0 | 0 |  | 1 |
|  |  | SLC18A2 | 0 | 0 |  | 1 |
| JH | whole targets | PPARG | 1.5 | 2 | Clustered | 7 |
|  |  | PTPN1 | 1.5 | 2 | Clustered | 6 |
|  |  | ESR2 | 1.5 | 2 | Clustered | 6 |
|  |  | ESR1 | 1.5 | 2 | Seed | 5 |
|  |  | CYP51A1 | 1.5 | 1 | Clustered | 4 |
|  |  | NR1H2 | 0 | 0 |  | 4 |
|  |  | CYP17A1 | 1.5 | 1 | Clustered | 3 |
|  |  | CYP2C19 | 1.5 | 1 | Seed | 3 |
|  |  | CYP19A1 | 1.5 | 1 | Clustered | 3 |
|  |  | NR1H3 | 0 | 0 |  | 3 |
|  |  | PTPN6 | 0 | 0 |  | 3 |
|  |  | FDFT1 | 0 | 0 |  | 2 |
|  |  | PPARD | 0 | 0 |  | 2 |
|  |  | VDR | 0 | 0 |  | 2 |
|  |  | PTPN2 | 0 | 0 |  | 2 |
|  |  | DHCR7 | 0 | 0 |  | 2 |
|  |  | PPARA | 0 | 0 |  | 2 |
|  |  | NOS2 | 0 | 0 |  | 1 |
|  |  | G6PD | 0 | 0 |  | 1 |
|  |  | AR | 0 | 0 |  | 1 |
|  | VMC-related targets | CYP51A1 |  |  |  | 2 |
|  |  | PPARG |  |  |  | 2 |
|  |  | NOS2 |  |  |  | 1 |
|  |  | FDFT1 |  |  |  | 1 |
|  |  | VDR |  |  |  | 1 |
|  |  | CYP2C19 |  |  |  | 1 |
| CH | whole targets | APP | 9.470588235 | 2 | Clustered | 104 |
|  |  | SRC | 5.093023256 | 1 | Clustered | 87 |
|  |  | EGFR | 5.093023256 | 1 | Clustered | 77 |
|  |  | GSK3B | 5.093023256 | 1 | Clustered | 59 |
|  |  | CDK1 | 5.093023256 | 1 | Clustered | 57 |
|  |  | CDK2 | 2.181818182 | 4 | Clustered | 55 |
|  |  | NTRK1 | 5.093023256 | 1 | Clustered | 54 |
|  |  | PTK2 | 3.5 | 3 | Clustered | 50 |
|  |  | AKT1 | 5.093023256 | 1 | Clustered | 48 |
|  |  | PIK3R1 | 5.093023256 | 1 | Clustered | 46 |
|  |  | MAPT | 3.5 | 3 | Clustered | 40 |
|  |  | ABL1 | 5.093023256 | 1 | Clustered | 39 |
|  |  | CSNK2A1 | 5.093023256 | 1 | Clustered | 38 |
|  |  | AR | 0 | 0 |  | 38 |
|  |  | CDK5 | 2.181818182 | 4 | Clustered | 37 |
|  |  | ESR1 | 5.093023256 | 1 | Clustered | 36 |
|  |  | INSR | 3.5 | 3 | Clustered | 34 |
|  |  | BDKRB1 | 9.470588235 | 2 | Clustered | 33 |
|  |  | SIRT1 | 2.181818182 | 4 | Clustered | 33 |
|  |  | RPS6KA3 | 5.093023256 | 1 | Clustered | 32 |
|  |  | PRKDC | 5.093023256 | 1 | Clustered | 31 |
|  |  | SYK | 5.093023256 | 1 | Seed | 31 |
|  |  | PABPC1 | 3.5 | 3 | Clustered | 31 |
|  |  | IGF1R | 3.5 | 3 | Clustered | 31 |
|  |  | PLK1 | 1.333333333 | 5 | Clustered | 31 |
|  |  | APEX1 | 1.333333333 | 5 | Clustered | 31 |
|  |  | NMUR2 | 9.470588235 | 2 | Clustered | 30 |
|  |  | MAPK8 | 5.093023256 | 1 | Clustered | 30 |
|  |  | PTPN1 | 0 | 0 |  | 30 |
|  |  | LCK | 3.5 | 3 | Clustered | 28 |
|  |  | PIK3CA | 3.5 | 3 | Clustered | 28 |
|  |  | DRD2 | 9.470588235 | 2 | Clustered | 27 |
|  |  | ESR2 | 5.093023256 | 1 | Clustered | 27 |
|  |  | JAK2 | 3.5 | 3 | Clustered | 27 |
|  |  | CDK4 | 1.333333333 | 5 | Clustered | 26 |
|  |  | CHRM2 | 9.470588235 | 2 | Clustered | 25 |
|  |  | CASP3 | 5.093023256 | 1 | Clustered | 25 |
|  |  | MET | 3.5 | 3 | Clustered | 25 |
|  |  | CHEK1 | 3.5 | 3 | Clustered | 25 |
|  |  | CNR1 | 9.470588235 | 2 | Clustered | 24 |
|  |  | OPRM1 | 9.470588235 | 2 | Clustered | 24 |
|  |  | ADORA1 | 9.470588235 | 2 | Clustered | 24 |
|  |  | CHEK2 | 2.181818182 | 4 | Clustered | 24 |
|  |  | DRD4 | 9.470588235 | 2 | Clustered | 23 |
|  |  | PARP1 | 5.093023256 | 1 | Clustered | 23 |
|  |  | HDAC2 | 5.093023256 | 1 | Clustered | 23 |
|  |  | PPARG | 5.093023256 | 1 | Clustered | 23 |
|  |  | PIK3CB | 5.093023256 | 1 | Clustered | 23 |
|  |  | PTPN6 | 3.5 | 3 | Seed | 23 |
|  |  | MAPK9 | 2.181818182 | 4 | Clustered | 23 |
|  |  | F2 | 9.470588235 | 2 | Clustered | 22 |
|  |  | F2R | 9.470588235 | 2 | Clustered | 22 |
|  |  | OPRD1 | 9.470588235 | 2 | Clustered | 22 |
|  |  | JAK1 | 5.093023256 | 1 | Clustered | 22 |
|  |  | SNCA | 5.093023256 | 1 | Clustered | 22 |
|  |  | MTOR | 3.5 | 3 | Clustered | 22 |
|  |  | PSEN1 | 1 | 6 | Clustered | 22 |
|  |  | HTR1A | 9.470588235 | 2 | Clustered | 21 |
|  |  | ADRA2A | 9.470588235 | 2 | Clustered | 21 |
|  |  | GRM5 | 9.470588235 | 2 | Clustered | 21 |
|  |  | CXCR1 | 9.470588235 | 2 | Clustered | 21 |
|  |  | KIT | 5.093023256 | 1 | Clustered | 21 |
|  |  | CAPN1 | 1.333333333 | 5 | Clustered | 21 |
|  |  | TNF | 0 | 0 |  | 21 |
|  |  | ADRA2C | 9.470588235 | 2 | Clustered | 20 |
|  |  | SSTR4 | 9.470588235 | 2 | Clustered | 20 |
|  |  | CHRM4 | 9.470588235 | 2 | Clustered | 20 |
|  |  | ADORA3 | 9.470588235 | 2 | Clustered | 20 |
|  |  | CNR2 | 9.470588235 | 2 | Clustered | 20 |
|  |  | HCK | 5.093023256 | 1 | Clustered | 20 |
|  |  | CCND1 | 5.093023256 | 1 | Clustered | 20 |
|  |  | MAP2K1 | 1.333333333 | 5 | Clustered | 19 |
|  |  | GRM4 | 9.470588235 | 2 | Clustered | 18 |
|  |  | HTR2A | 9.470588235 | 2 | Clustered | 18 |
|  |  | ALDH2 | 3.5 | 3 | Clustered | 18 |
|  |  | FGFR1 | 3.5 | 3 | Clustered | 18 |
|  |  | CDK6 | 2.181818182 | 4 | Seed | 18 |
|  |  | GRIN2B | 0 | 0 |  | 18 |
|  |  | CFTR | 0 | 0 |  | 18 |
|  |  | PTAFR | 9.470588235 | 2 | Clustered | 17 |
|  |  | NPY5R | 9.470588235 | 2 | Clustered | 17 |
|  |  | TAS2R31 | 9.470588235 | 2 | Seed | 17 |
|  |  | KDR | 3.5 | 3 | Clustered | 17 |
|  |  | PTGER1 | 9.470588235 | 2 | Clustered | 16 |
|  |  | P2RY1 | 9.470588235 | 2 | Clustered | 16 |
|  |  | CHRM3 | 9.470588235 | 2 | Clustered | 16 |
|  |  | HCRTR1 | 9.470588235 | 2 | Clustered | 16 |
|  |  | CHRM1 | 9.470588235 | 2 | Clustered | 16 |
|  |  | CCNE1 | 5.093023256 | 1 | Clustered | 16 |
|  |  | TOP1 | 5.093023256 | 1 | Clustered | 16 |
|  |  | JAK3 | 3.5 | 3 | Clustered | 16 |
|  |  | CCND2 | 3.5 | 3 | Clustered | 16 |
|  |  | GCK | 3.5 | 3 | Clustered | 16 |
|  |  | TOP2A | 2.181818182 | 4 | Clustered | 16 |
|  |  | PTPN2 | 2.181818182 | 4 | Clustered | 16 |
|  |  | CCNB1 | 0 | 0 |  | 16 |
|  |  | CCNA2 | 0 | 0 |  | 16 |
|  |  | CDK9 | 0 | 0 |  | 16 |
|  |  | PPIA | 0 | 0 |  | 16 |
|  |  | ITGB3 | 0 | 0 |  | 16 |
|  |  | F2RL1 | 9.470588235 | 2 | Clustered | 15 |
|  |  | TACR3 | 9.470588235 | 2 | Clustered | 15 |
|  |  | HCRTR2 | 9.470588235 | 2 | Clustered | 15 |
|  |  | CCND3 | 5.093023256 | 1 | Clustered | 14 |
|  |  | WEE1 | 2.181818182 | 4 | Clustered | 14 |
|  |  | MCL1 | 0 | 0 |  | 14 |
|  |  | ROCK1 | 0 | 0 |  | 14 |
|  |  | TERT | 0 | 0 |  | 14 |
|  |  | ADORA2A | 5.093023256 | 1 | Clustered | 13 |
|  |  | AKR1B10 | 3.5 | 3 | Clustered | 13 |
|  |  | PYGL | 3.5 | 3 | Clustered | 13 |
|  |  | AXL | 3.5 | 3 | Clustered | 13 |
|  |  | GRIN1 | 0 | 0 |  | 13 |
|  |  | ACACA | 0 | 0 |  | 13 |
|  |  | CDK5R1 | 0 | 0 |  | 13 |
|  |  | BRAF | 0 | 0 |  | 13 |
|  |  | NCSTN | 2.181818182 | 4 | Clustered | 12 |
|  |  | CDC7 | 2.181818182 | 4 | Clustered | 12 |
|  |  | PSEN2 | 2.181818182 | 4 | Clustered | 12 |
|  |  | PIK3CG | 5.093023256 | 1 | Clustered | 11 |
|  |  | CSF1R | 5.093023256 | 1 | Clustered | 11 |
|  |  | PIK3CD | 5.093023256 | 1 | Clustered | 11 |
|  |  | CCNB2 | 1.333333333 | 5 | Clustered | 11 |
|  |  | CCNA1 | 1.333333333 | 5 | Clustered | 11 |
|  |  | PIM1 | 0 | 0 |  | 11 |
|  |  | ALK | 5.093023256 | 1 | Clustered | 10 |
|  |  | FLT3 | 5.093023256 | 1 | Clustered | 10 |
|  |  | AKR1B1 | 5.093023256 | 1 | Clustered | 10 |
|  |  | AKR1A1 | 3.5 | 3 | Clustered | 10 |
|  |  | AKR1C4 | 3.5 | 3 | Clustered | 10 |
|  |  | AKR1C2 | 3.5 | 3 | Clustered | 10 |
|  |  | AKR1C1 | 3.5 | 3 | Clustered | 10 |
|  |  | ITK | 3.5 | 3 | Clustered | 10 |
|  |  | ROCK2 | 1.333333333 | 5 | Clustered | 10 |
|  |  | CASP1 | 1 | 6 | Clustered | 10 |
|  |  | CASP7 | 1 | 6 | Clustered | 10 |
|  |  | ADAM17 | 0 | 0 |  | 10 |
|  |  | VDR | 0 | 0 |  | 10 |
|  |  | PTGER2 | 5.093023256 | 1 | Clustered | 9 |
|  |  | IL2 | 5.093023256 | 1 | Clustered | 9 |
|  |  | AVPR2 | 5.093023256 | 1 | Clustered | 9 |
|  |  | HTR6 | 5.093023256 | 1 | Clustered | 9 |
|  |  | CYP51A1 | 2.181818182 | 4 | Clustered | 9 |
|  |  | CA9 | 0 | 0 |  | 9 |
|  |  | MMP1 | 0 | 0 |  | 9 |
|  |  | MYLK | 0 | 0 |  | 9 |
|  |  | PDE4D | 0 | 0 |  | 9 |
|  |  | AURKB | 0 | 0 |  | 9 |
|  |  | MMP7 | 0 | 0 |  | 9 |
|  |  | ITGAV | 0 | 0 |  | 9 |
|  |  | MMP2 | 0 | 0 |  | 9 |
|  |  | MAPK10 | 0 | 0 |  | 9 |
|  |  | CYP17A1 | 2.181818182 | 4 | Clustered | 8 |
|  |  | G6PD | 1.333333333 | 5 | Clustered | 8 |
|  |  | NR1H3 | 0 | 0 |  | 8 |
|  |  | TGM2 | 0 | 0 |  | 8 |
|  |  | CTSV | 0 | 0 |  | 8 |
|  |  | PPARD | 0 | 0 |  | 8 |
|  |  | PKN1 | 0 | 0 |  | 8 |
|  |  | NOS1 | 0 | 0 |  | 8 |
|  |  | HTR7 | 5.093023256 | 1 | Clustered | 7 |
|  |  | ESRRA | 5.093023256 | 1 | Clustered | 7 |
|  |  | CYP1B1 | 2.181818182 | 4 | Clustered | 7 |
|  |  | CYP19A1 | 2.181818182 | 4 | Clustered | 7 |
|  |  | BRD4 | 0 | 0 |  | 7 |
|  |  | PLG | 0 | 0 |  | 7 |
|  |  | DAPK1 | 0 | 0 |  | 7 |
|  |  | EPHB4 | 0 | 0 |  | 7 |
|  |  | AKR1C3 | 0 | 0 |  | 7 |
|  |  | DAPK3 | 0 | 0 |  | 7 |
|  |  | MME | 0 | 0 |  | 7 |
|  |  | CCNE2 | 0 | 0 |  | 7 |
|  |  | CALCRL | 5.093023256 | 1 | Clustered | 6 |
|  |  | HRH2 | 5.093023256 | 1 | Clustered | 6 |
|  |  | CYP2C19 | 2.181818182 | 4 | Clustered | 6 |
|  |  | CYP11B1 | 2.181818182 | 4 | Clustered | 6 |
|  |  | CYP11B2 | 2.181818182 | 4 | Clustered | 6 |
|  |  | PSENEN | 2.181818182 | 4 | Clustered | 6 |
|  |  | TRPV1 | 0 | 0 |  | 6 |
|  |  | NOS2 | 0 | 0 |  | 6 |
|  |  | CCNB3 | 0 | 0 |  | 6 |
|  |  | PLA2G2A | 0 | 0 |  | 6 |
|  |  | NUAK1 | 0 | 0 |  | 6 |
|  |  | ADAM10 | 0 | 0 |  | 6 |
|  |  | CAMK2B | 0 | 0 |  | 6 |
|  |  | CASP6 | 0 | 0 |  | 6 |
|  |  | MMP3 | 0 | 0 |  | 6 |
|  |  | BACE1 | 0 | 0 |  | 6 |
|  |  | NAMPT | 0 | 0 |  | 6 |
|  |  | ALOX5 | 0 | 0 |  | 6 |
|  |  | MMP14 | 0 | 0 |  | 6 |
|  |  | NR1H2 | 0 | 0 |  | 6 |
|  |  | APH1B | 2.181818182 | 4 | Clustered | 5 |
|  |  | APH1A | 2.181818182 | 4 | Clustered | 5 |
|  |  | PREP | 0 | 0 |  | 5 |
|  |  | NAE1 | 0 | 0 |  | 5 |
|  |  | PFKFB3 | 0 | 0 |  | 5 |
|  |  | PI4KB | 0 | 0 |  | 5 |
|  |  | GLO1 | 0 | 0 |  | 5 |
|  |  | AHR | 0 | 0 |  | 5 |
|  |  | MPG | 0 | 0 |  | 5 |
|  |  | PPARA | 0 | 0 |  | 5 |
|  |  | GRK6 | 0 | 0 |  | 5 |
|  |  | TXK | 0 | 0 |  | 5 |
|  |  | PTGS2 | 0 | 0 |  | 5 |
|  |  | ABCB1 | 0 | 0 |  | 5 |
|  |  | MMP13 | 0 | 0 |  | 5 |
|  |  | PTPRS | 0 | 0 |  | 4 |
|  |  | SMO | 0 | 0 |  | 4 |
|  |  | ABCC1 | 0 | 0 |  | 4 |
|  |  | KCNJ3 | 0 | 0 |  | 4 |
|  |  | FDFT1 | 0 | 0 |  | 4 |
|  |  | ADA | 0 | 0 |  | 4 |
|  |  | FABP4 | 0 | 0 |  | 4 |
|  |  | FNTA | 0 | 0 |  | 4 |
|  |  | FBP1 | 0 | 0 |  | 4 |
|  |  | SOAT1 | 0 | 0 |  | 4 |
|  |  | TNKS | 0 | 0 |  | 4 |
|  |  | CTSK | 0 | 0 |  | 4 |
|  |  | SREBF2 | 0 | 0 |  | 4 |
|  |  | PDE4B | 0 | 0 |  | 4 |
|  |  | NEK2 | 0 | 0 |  | 3 |
|  |  | KCNK3 | 0 | 0 |  | 3 |
|  |  | ERN1 | 0 | 0 |  | 3 |
|  |  | DHCR7 | 0 | 0 |  | 3 |
|  |  | ARG1 | 0 | 0 |  | 3 |
|  |  | ELANE | 0 | 0 |  | 3 |
|  |  | TTR | 0 | 0 |  | 3 |
|  |  | CTSL | 0 | 0 |  | 3 |
|  |  | TGM1 | 0 | 0 |  | 3 |
|  |  | SLC6A4 | 0 | 0 |  | 3 |
|  |  | KCNJ5 | 0 | 0 |  | 3 |
|  |  | SLC6A3 | 0 | 0 |  | 3 |
|  |  | FABP3 | 0 | 0 |  | 3 |
|  |  | DBF4 | 0 | 0 |  | 3 |
|  |  | CA3 | 0 | 0 |  | 2 |
|  |  | HSD11B1 | 0 | 0 |  | 2 |
|  |  | ACACB | 0 | 0 |  | 2 |
|  |  | POLB | 0 | 0 |  | 2 |
|  |  | NEK6 | 0 | 0 |  | 2 |
|  |  | HMGCR | 0 | 0 |  | 2 |
|  |  | F13A1 | 0 | 0 |  | 2 |
|  |  | CPT1A | 0 | 0 |  | 2 |
|  |  | MMP9 | 0 | 0 |  | 2 |
|  |  | P2RX7 | 0 | 0 |  | 2 |
|  |  | EPHX1 | 0 | 0 |  | 2 |
|  |  | MPO | 0 | 0 |  | 2 |
|  |  | F10 | 0 | 0 |  | 2 |
|  |  | SQLE | 0 | 0 |  | 2 |
|  |  | FNTB | 0 | 0 |  | 2 |
|  |  | PNP | 0 | 0 |  | 2 |
|  |  | ODC1 | 0 | 0 |  | 2 |
|  |  | ALOX5AP | 0 | 0 |  | 2 |
|  |  | CA2 | 0 | 0 |  | 2 |
|  |  | TNKS2 | 0 | 0 |  | 1 |
|  |  | HSD11B2 | 0 | 0 |  | 1 |
|  |  | PLA2G1B | 0 | 0 |  | 1 |
|  |  | NQO2 | 0 | 0 |  | 1 |
|  |  | CMA1 | 0 | 0 |  | 1 |
|  |  | NR1I3 | 0 | 0 |  | 1 |
|  |  | CPT2 | 0 | 0 |  | 1 |
|  |  | MMP12 | 0 | 0 |  | 1 |
|  |  | KCNJ6 | 0 | 0 |  | 1 |
|  |  | TDP2 | 0 | 0 |  | 1 |
|  |  | CA14 | 0 | 0 |  | 1 |
|  |  | PTGES | 0 | 0 |  | 1 |
|  |  | ALOX15 | 0 | 0 |  | 1 |
|  |  | ALOX12 | 0 | 0 |  | 1 |
|  |  | HSD17B1 | 0 | 0 |  | 1 |
|  |  | ABCG2 | 0 | 0 |  | 1 |
|  |  | LYPLA2 | 0 | 0 |  | 1 |
|  |  | SHBG | 0 | 0 |  | 1 |
|  |  | RORC | 0 | 0 |  | 1 |
|  |  | SLC6A2 | 0 | 0 |  | 1 |
|  |  | PDE10A | 0 | 0 |  | 1 |
|  |  | UGT2B7 | 0 | 0 |  | 1 |
|  |  | SLC5A1 | 0 | 0 |  | 1 |
|  |  | DGAT1 | 0 | 0 |  | 1 |
|  |  | ACHE | 0 | 0 |  | 1 |
|  |  | CD38 | 0 | 0 |  | 1 |
|  |  | RBP4 | 0 | 0 |  | 1 |
|  |  | PRCP | 0 | 0 |  | 1 |
|  |  | FAP | 0 | 0 |  | 1 |
|  |  | KCNK9 | 0 | 0 |  | 1 |
|  |  | AMY1A | 0 | 0 |  | 1 |
|  |  | XDH | 0 | 0 |  | 1 |
|  |  | ECE1 | 0 | 0 |  | 1 |
|  |  | FAAH | 0 | 0 |  | 1 |
|  |  | MAOA | 0 | 0 |  | 1 |
|  | VMC-related targets | EGFR | 1.7 | 1 | Clustered | 22 |
|  |  | SRC | 2.25 | 2 | Clustered | 21 |
|  |  | PTK2 | 2.25 | 2 | Clustered | 16 |
|  |  | NTRK1 | 2.25 | 2 | Clustered | 14 |
|  |  | CASP3 | 0 | 0 |  | 12 |
|  |  | AKT1 | 2.25 | 2 | Clustered | 11 |
|  |  | MET | 2.25 | 2 | Clustered | 10 |
|  |  | ABL1 | 2.25 | 2 | Clustered | 10 |
|  |  | LCK | 1.7 | 1 | Clustered | 10 |
|  |  | MAPK8 | 2.25 | 2 | Clustered | 9 |
|  |  | PIK3CA | 1.7 | 1 | Seed | 9 |
|  |  | PARP1 | 2.25 | 2 | Seed | 8 |
|  |  | TNF | 0 | 0 |  | 8 |
|  |  | MMP1 | 0 | 0 |  | 7 |
|  |  | PPARG | 0 | 0 |  | 7 |
|  |  | CASP1 | 1.7 | 1 | Clustered | 6 |
|  |  | PSEN1 | 1.7 | 1 | Clustered | 6 |
|  |  | CASP7 | 1.7 | 1 | Clustered | 6 |
|  |  | PIK3CG | 1.7 | 1 | Clustered | 6 |
|  |  | KIT | 1.7 | 1 | Clustered | 6 |
|  |  | ITGB3 | 0 | 0 |  | 6 |
|  |  | ITGAV | 0 | 0 |  | 6 |
|  |  | MTOR | 0 | 0 |  | 5 |
|  |  | PSEN2 | 1.7 | 1 | Clustered | 4 |
|  |  | ITK | 1.7 | 1 | Clustered | 4 |
|  |  | MMP2 | 0 | 0 |  | 4 |
|  |  | F2 | 0 | 0 |  | 4 |
|  |  | ADAM17 | 0 | 0 |  | 4 |
|  |  | ADORA2A | 0 | 0 |  | 4 |
|  |  | DRD2 | 0 | 0 |  | 4 |
|  |  | CHRM2 | 0 | 0 |  | 3 |
|  |  | CYP51A1 | 0 | 0 |  | 3 |
|  |  | VDR | 0 | 0 |  | 3 |
|  |  | PLG | 0 | 0 |  | 3 |
|  |  | FDFT1 | 0 | 0 |  | 3 |
|  |  | ELANE | 0 | 0 |  | 3 |
|  |  | G6PD | 0 | 0 |  | 3 |
|  |  | MMP9 | 0 | 0 |  | 2 |
|  |  | NOS2 | 0 | 0 |  | 2 |
|  |  | MMP3 | 0 | 0 |  | 2 |
|  |  | IL2 | 0 | 0 |  | 2 |
|  |  | CCND1 | 0 | 0 |  | 1 |
|  |  | MPO | 0 | 0 |  | 1 |
|  |  | KCNJ5 | 0 | 0 |  | 1 |
|  |  | ABCC1 | 0 | 0 |  | 1 |
|  |  | CYP2C19 | 0 | 0 |  | 1 |
|  |  | FABP3 | 0 | 0 |  | 1 |
|  |  | PPARA | 0 | 0 |  | 1 |
|  |  | ARG1 | 0 | 0 |  | 1 |
|  |  | TDP2 | 0 | 0 |  | 1 |
|  |  | P2RY1 | 0 | 0 |  | 1 |
|  |  | CMA1 | 0 | 0 |  | 1 |
|  |  | ABCB1 | 0 | 0 |  | 1 |
|  |  | ADA | 0 | 0 |  | 1 |
|  |  | HRH2 | 0 | 0 |  | 1 |
| JXT | whole targets | APP | 13.28571429 | 4 | Clustered | 139 |
|  |  | HSP90AA1 | 7.76 | 5 | Clustered | 135 |
|  |  | SRC | 7.76 | 5 | Seed | 133 |
|  |  | HSP90AB1 | 7.76 | 5 | Clustered | 127 |
|  |  | EGFR | 7.76 | 5 | Clustered | 117 |
|  |  | PRKCA | 7.76 | 5 | Clustered | 104 |
|  |  | MAPK1 | 7.76 | 5 | Clustered | 93 |
|  |  | AKT1 | 7.76 | 5 | Clustered | 86 |
|  |  | CDK1 | 3.756097561 | 3 | Clustered | 84 |
|  |  | CDK2 | 3.444444444 | 1 | Clustered | 84 |
|  |  | GSK3B | 3.756097561 | 3 | Seed | 83 |
|  |  | MAPK3 | 7.76 | 5 | Clustered | 79 |
|  |  | PRKACA | 7.76 | 5 | Clustered | 76 |
|  |  | MAPK14 | 2.558139535 | 2 | Clustered | 75 |
|  |  | PTK2 | 7.76 | 5 | Clustered | 74 |
|  |  | FYN | 3.756097561 | 3 | Clustered | 69 |
|  |  | ESR1 | 3.444444444 | 1 | Seed | 69 |
|  |  | PIK3R1 | 3.756097561 | 3 | Clustered | 67 |
|  |  | AR | 7.76 | 5 | Clustered | 64 |
|  |  | LYN | 7.76 | 5 | Clustered | 61 |
|  |  | VCP | 3.756097561 | 3 | Clustered | 61 |
|  |  | MDM2 | 3.444444444 | 1 | Clustered | 60 |
|  |  | ABL1 | 3.756097561 | 3 | Clustered | 56 |
|  |  | CSNK2A1 | 3.756097561 | 3 | Clustered | 53 |
|  |  | MAPK8 | 3.444444444 | 1 | Clustered | 50 |
|  |  | SYK | 3.756097561 | 3 | Clustered | 49 |
|  |  | PTPN11 | 3.756097561 | 3 | Clustered | 49 |
|  |  | PRKCB | 3.444444444 | 1 | Clustered | 49 |
|  |  | LCK | 7.76 | 5 | Clustered | 48 |
|  |  | CDK5 | 3.444444444 | 1 | Clustered | 47 |
|  |  | INSR | 7.76 | 5 | Clustered | 46 |
|  |  | BDKRB1 | 13.28571429 | 4 | Clustered | 45 |
|  |  | PLK1 | 3.444444444 | 1 | Clustered | 45 |
|  |  | NCOR1 | 2.558139535 | 2 | Clustered | 45 |
|  |  | PPARG | 3.756097561 | 3 | Clustered | 43 |
|  |  | RAF1 | 3.756097561 | 3 | Clustered | 43 |
|  |  | ESR2 | 3.444444444 | 1 | Clustered | 43 |
|  |  | HDAC1 | 2.558139535 | 2 | Clustered | 43 |
|  |  | JAK2 | 7.76 | 5 | Clustered | 42 |
|  |  | RPS6KB1 | 7.76 | 5 | Clustered | 41 |
|  |  | PRKCZ | 3.756097561 | 3 | Clustered | 41 |
|  |  | AURKA | 3.756097561 | 3 | Clustered | 41 |
|  |  | IGF1R | 3.756097561 | 3 | Clustered | 41 |
|  |  | CDK4 | 2.558139535 | 2 | Clustered | 41 |
|  |  | MET | 7.76 | 5 | Clustered | 38 |
|  |  | CSK | 3.756097561 | 3 | Clustered | 38 |
|  |  | BCL2 | 3.444444444 | 1 | Clustered | 38 |
|  |  | PARP1 | 3.444444444 | 1 | Clustered | 38 |
|  |  | MTNR1B | 13.28571429 | 4 | Clustered | 37 |
|  |  | CCND1 | 3.756097561 | 3 | Clustered | 37 |
|  |  | EPHA2 | 3.444444444 | 1 | Clustered | 37 |
|  |  | MTNR1A | 13.28571429 | 4 | Clustered | 36 |
|  |  | YES1 | 7.76 | 5 | Clustered | 36 |
|  |  | NR3C1 | 7.76 | 5 | Clustered | 36 |
|  |  | HDAC3 | 3.444444444 | 1 | Clustered | 36 |
|  |  | HDAC2 | 2.558139535 | 2 | Clustered | 36 |
|  |  | PTPN1 | 3.444444444 | 1 | Clustered | 35 |
|  |  | MAPK9 | 0 | 0 |  | 35 |
|  |  | SNCA | 7.76 | 5 | Clustered | 34 |
|  |  | PIK3CA | 7.76 | 5 | Clustered | 34 |
|  |  | TNF | 2.558139535 | 2 | Clustered | 34 |
|  |  | S1PR1 | 13.28571429 | 4 | Clustered | 33 |
|  |  | DRD2 | 13.28571429 | 4 | Clustered | 33 |
|  |  | CHRM2 | 13.28571429 | 4 | Clustered | 33 |
|  |  | PTPN6 | 7.76 | 5 | Clustered | 33 |
|  |  | NCOR2 | 2.558139535 | 2 | Clustered | 33 |
|  |  | CHEK1 | 3.756097561 | 3 | Clustered | 32 |
|  |  | HDAC6 | 3.756097561 | 3 | Clustered | 32 |
|  |  | MTOR | 3.444444444 | 1 | Clustered | 32 |
|  |  | CASP3 | 1 | 8 | Clustered | 32 |
|  |  | CNR1 | 13.28571429 | 4 | Clustered | 30 |
|  |  | IKBKB | 3.756097561 | 3 | Clustered | 30 |
|  |  | PDPK1 | 3.444444444 | 1 | Clustered | 30 |
|  |  | CFTR | 3.444444444 | 1 | Clustered | 30 |
|  |  | JAK1 | 3.444444444 | 1 | Clustered | 30 |
|  |  | KAT2B | 2.558139535 | 2 | Clustered | 30 |
|  |  | EZR | 0 | 0 |  | 30 |
|  |  | NOS3 | 0 | 0 |  | 30 |
|  |  | ADRA2A | 13.28571429 | 4 | Clustered | 29 |
|  |  | DRD4 | 13.28571429 | 4 | Clustered | 29 |
|  |  | PTGER3 | 13.28571429 | 4 | Clustered | 29 |
|  |  | ADORA1 | 13.28571429 | 4 | Clustered | 29 |
|  |  | FPR1 | 13.28571429 | 4 | Clustered | 29 |
|  |  | MAP2K1 | 3.444444444 | 1 | Clustered | 29 |
|  |  | CHEK2 | 3.444444444 | 1 | Clustered | 29 |
|  |  | HDAC4 | 2.558139535 | 2 | Clustered | 29 |
|  |  | HTT | 0 | 0 |  | 29 |
|  |  | CXCR1 | 13.28571429 | 4 | Clustered | 28 |
|  |  | CHRM4 | 13.28571429 | 4 | Clustered | 28 |
|  |  | ADORA3 | 13.28571429 | 4 | Clustered | 28 |
|  |  | CNR2 | 13.28571429 | 4 | Clustered | 28 |
|  |  | DRD3 | 13.28571429 | 4 | Clustered | 28 |
|  |  | CDC25A | 3.756097561 | 3 | Clustered | 28 |
|  |  | HDAC5 | 0 | 0 |  | 28 |
|  |  | ADRA2C | 13.28571429 | 4 | Clustered | 27 |
|  |  | ADRA2B | 13.28571429 | 4 | Clustered | 27 |
|  |  | HTR1A | 13.28571429 | 4 | Clustered | 27 |
|  |  | CXCR3 | 13.28571429 | 4 | Clustered | 27 |
|  |  | NPY1R | 13.28571429 | 4 | Clustered | 27 |
|  |  | OPRK1 | 13.28571429 | 4 | Clustered | 27 |
|  |  | F2 | 8.5 | 6 | Clustered | 27 |
|  |  | GRM5 | 8.5 | 6 | Clustered | 27 |
|  |  | BTK | 3.756097561 | 3 | Clustered | 27 |
|  |  | FASN | 2.558139535 | 2 | Seed | 27 |
|  |  | BCL2L1 | 2.558139535 | 2 | Clustered | 27 |
|  |  | PDGFRB | 7.76 | 5 | Clustered | 26 |
|  |  | RET | 3.756097561 | 3 | Clustered | 26 |
|  |  | KDR | 3.756097561 | 3 | Clustered | 26 |
|  |  | CDK6 | 3.444444444 | 1 | Clustered | 26 |
|  |  | PSEN1 | 0 | 0 |  | 26 |
|  |  | HRH3 | 13.28571429 | 4 | Clustered | 25 |
|  |  | GRM4 | 13.28571429 | 4 | Clustered | 25 |
|  |  | S1PR3 | 13.28571429 | 4 | Clustered | 25 |
|  |  | KDM1A | 3.444444444 | 1 | Clustered | 25 |
|  |  | CDC25B | 3.444444444 | 1 | Clustered | 25 |
|  |  | CCND2 | 2.558139535 | 2 | Clustered | 25 |
|  |  | ALDH2 | 2.558139535 | 2 | Clustered | 25 |
|  |  | NPY5R | 13.28571429 | 4 | Seed | 24 |
|  |  | MAPKAPK2 | 3.444444444 | 1 | Clustered | 24 |
|  |  | CCNA2 | 3.444444444 | 1 | Clustered | 24 |
|  |  | CCNB1 | 2.558139535 | 2 | Clustered | 24 |
|  |  | TOP1 | 2.558139535 | 2 | Clustered | 24 |
|  |  | HTR2A | 8.5 | 6 | Clustered | 23 |
|  |  | CAPN1 | 3.444444444 | 1 | Clustered | 23 |
|  |  | EIF2AK2 | 3.444444444 | 1 | Clustered | 23 |
|  |  | CDK3 | 2.558139535 | 2 | Clustered | 23 |
|  |  | HRH1 | 8.5 | 6 | Clustered | 22 |
|  |  | GNRHR | 8.5 | 6 | Clustered | 22 |
|  |  | EPHB2 | 3.756097561 | 3 | Clustered | 22 |
|  |  | CCNE1 | 3.444444444 | 1 | Clustered | 22 |
|  |  | ADRA1B | 8.5 | 6 | Clustered | 21 |
|  |  | FGR | 7.76 | 5 | Clustered | 21 |
|  |  | BRAF | 3.756097561 | 3 | Clustered | 21 |
|  |  | TOP2A | 2.558139535 | 2 | Clustered | 21 |
|  |  | PDK1 | 2.558139535 | 2 | Clustered | 21 |
|  |  | HCRTR1 | 8.5 | 6 | Clustered | 20 |
|  |  | PTGFR | 8.5 | 6 | Clustered | 20 |
|  |  | HTR2B | 8.5 | 6 | Clustered | 20 |
|  |  | PTGER1 | 8.5 | 6 | Clustered | 20 |
|  |  | CHRM3 | 8.5 | 6 | Clustered | 20 |
|  |  | CHRM1 | 8.5 | 6 | Clustered | 20 |
|  |  | PPARD | 1 | 8 | Clustered | 20 |
|  |  | ROCK1 | 0 | 0 |  | 20 |
|  |  | TACR3 | 8.5 | 6 | Clustered | 19 |
|  |  | CHRM5 | 8.5 | 6 | Seed | 19 |
|  |  | HCRTR2 | 8.5 | 6 | Clustered | 19 |
|  |  | OXTR | 8.5 | 6 | Clustered | 19 |
|  |  | HTR2C | 8.5 | 6 | Clustered | 19 |
|  |  | NMBR | 8.5 | 6 | Clustered | 19 |
|  |  | AXL | 7.76 | 5 | Clustered | 19 |
|  |  | PKN1 | 3.756097561 | 3 | Clustered | 19 |
|  |  | ACP1 | 3.444444444 | 1 | Clustered | 19 |
|  |  | TERT | 2.558139535 | 2 | Clustered | 19 |
|  |  | MCL1 | 0 | 0 |  | 19 |
|  |  | MAPK10 | 0 | 0 |  | 19 |
|  |  | ITGA4 | 0 | 0 |  | 19 |
|  |  | EPHA1 | 3.756097561 | 3 | Clustered | 18 |
|  |  | EPHA4 | 3.756097561 | 3 | Clustered | 18 |
|  |  | CCND3 | 3.444444444 | 1 | Clustered | 18 |
|  |  | PDGFRA | 2.558139535 | 2 | Clustered | 18 |
|  |  | VDR | 1 | 8 | Clustered | 18 |
|  |  | DNM1 | 0 | 0 |  | 18 |
|  |  | PGR | 3.756097561 | 3 | Clustered | 17 |
|  |  | PYGL | 3.756097561 | 3 | Clustered | 17 |
|  |  | EPHA3 | 3.756097561 | 3 | Clustered | 17 |
|  |  | PIM1 | 3.444444444 | 1 | Clustered | 17 |
|  |  | PPARA | 3.444444444 | 1 | Clustered | 17 |
|  |  | PIK3CG | 2.558139535 | 2 | Clustered | 17 |
|  |  | WEE1 | 2.558139535 | 2 | Clustered | 17 |
|  |  | PPIA | 0 | 0 |  | 17 |
|  |  | PTPN2 | 0 | 0 |  | 17 |
|  |  | AVPR2 | 3.444444444 | 1 | Clustered | 16 |
|  |  | AURKB | 3.444444444 | 1 | Clustered | 16 |
|  |  | CCNB2 | 2.558139535 | 2 | Clustered | 16 |
|  |  | MDM4 | 1 | 8 | Seed | 16 |
|  |  | ROCK2 | 0 | 0 |  | 16 |
|  |  | GSTP1 | 0 | 0 |  | 16 |
|  |  | JAK3 | 0 | 0 |  | 16 |
|  |  | ITGB2 | 3.756097561 | 3 | Clustered | 15 |
|  |  | TYK2 | 3.444444444 | 1 | Clustered | 15 |
|  |  | PSEN2 | 1.8 | 7 | Clustered | 15 |
|  |  | PRKCH | 0 | 0 |  | 15 |
|  |  | KIF11 | 0 | 0 |  | 15 |
|  |  | BMX | 0 | 0 |  | 15 |
|  |  | FEN1 | 0 | 0 |  | 15 |
|  |  | LIMK1 | 0 | 0 |  | 15 |
|  |  | HDAC9 | 0 | 0 |  | 15 |
|  |  | PDE4D | 0 | 0 |  | 15 |
|  |  | NR1H3 | 0 | 0 |  | 15 |
|  |  | ITK | 3.756097561 | 3 | Clustered | 14 |
|  |  | NOS2 | 3.444444444 | 1 | Clustered | 14 |
|  |  | CDC7 | 3.444444444 | 1 | Clustered | 14 |
|  |  | ADORA2A | 3.444444444 | 1 | Clustered | 14 |
|  |  | CYP51A1 | 2.558139535 | 2 | Clustered | 14 |
|  |  | ADAM17 | 2.558139535 | 2 | Clustered | 14 |
|  |  | CDK5R1 | 2.558139535 | 2 | Clustered | 14 |
|  |  | DUSP3 | 0 | 0 |  | 14 |
|  |  | PTPRF | 0 | 0 |  | 14 |
|  |  | THRB | 0 | 0 |  | 14 |
|  |  | CYP17A1 | 2.558139535 | 2 | Clustered | 13 |
|  |  | NCSTN | 1.8 | 7 | Clustered | 13 |
|  |  | CTSD | 0 | 0 |  | 13 |
|  |  | ALK | 0 | 0 |  | 13 |
|  |  | BLK | 0 | 0 |  | 13 |
|  |  | ALOX5 | 0 | 0 |  | 13 |
|  |  | XIAP | 0 | 0 |  | 13 |
|  |  | DAPK1 | 0 | 0 |  | 13 |
|  |  | PTK6 | 0 | 0 |  | 13 |
|  |  | FLT3 | 0 | 0 |  | 13 |
|  |  | AKR1B10 | 3.756097561 | 3 | Clustered | 12 |
|  |  | HTR6 | 3.444444444 | 1 | Clustered | 12 |
|  |  | HDAC7 | 3.444444444 | 1 | Clustered | 12 |
|  |  | CCNA1 | 2.558139535 | 2 | Clustered | 12 |
|  |  | THRA | 2.558139535 | 2 | Clustered | 12 |
|  |  | CASP7 | 0 | 0 |  | 12 |
|  |  | AKR1B1 | 0 | 0 |  | 12 |
|  |  | NOS1 | 0 | 0 |  | 12 |
|  |  | AKR1A1 | 3.756097561 | 3 | Clustered | 11 |
|  |  | AKR1C2 | 3.756097561 | 3 | Clustered | 11 |
|  |  | AKR1C1 | 3.756097561 | 3 | Clustered | 11 |
|  |  | EPHA5 | 3.756097561 | 3 | Clustered | 11 |
|  |  | AKR1C4 | 3.756097561 | 3 | Clustered | 11 |
|  |  | HTR7 | 3.444444444 | 1 | Clustered | 11 |
|  |  | PTGER2 | 3.444444444 | 1 | Clustered | 11 |
|  |  | PTGIR | 3.444444444 | 1 | Clustered | 11 |
|  |  | PLK2 | 2.558139535 | 2 | Clustered | 11 |
|  |  | NR1I2 | 2.558139535 | 2 | Clustered | 11 |
|  |  | NR1H2 | 1 | 8 | Clustered | 11 |
|  |  | IL2 | 0 | 0 |  | 11 |
|  |  | CA9 | 0 | 0 |  | 11 |
|  |  | NEK2 | 0 | 0 |  | 11 |
|  |  | CCNE2 | 0 | 0 |  | 11 |
|  |  | PTGS2 | 0 | 0 |  | 11 |
|  |  | SF3B3 | 0 | 0 |  | 11 |
|  |  | PIK3CD | 0 | 0 |  | 11 |
|  |  | EPHB3 | 3.756097561 | 3 | Clustered | 10 |
|  |  | EPHA6 | 3.756097561 | 3 | Clustered | 10 |
|  |  | PLAA | 2.558139535 | 2 | Clustered | 10 |
|  |  | ESRRB | 0 | 0 |  | 10 |
|  |  | MMP2 | 0 | 0 |  | 10 |
|  |  | ADAM10 | 0 | 0 |  | 10 |
|  |  | GABRG2 | 0 | 0 |  | 10 |
|  |  | MIF | 0 | 0 |  | 10 |
|  |  | ADORA2B | 3.444444444 | 1 | Clustered | 9 |
|  |  | CYP19A1 | 2.558139535 | 2 | Clustered | 9 |
|  |  | CYP1B1 | 2.558139535 | 2 | Clustered | 9 |
|  |  | IMPDH1 | 2.558139535 | 2 | Clustered | 9 |
|  |  | PLAU | 2.558139535 | 2 | Clustered | 9 |
|  |  | MMP7 | 2.558139535 | 2 | Clustered | 9 |
|  |  | G6PD | 0 | 0 |  | 9 |
|  |  | GRK6 | 0 | 0 |  | 9 |
|  |  | IL6 | 0 | 0 |  | 9 |
|  |  | PLG | 0 | 0 |  | 9 |
|  |  | DYRK1B | 0 | 0 |  | 9 |
|  |  | CHRNA7 | 0 | 0 |  | 9 |
|  |  | ESRRA | 0 | 0 |  | 9 |
|  |  | AHR | 0 | 0 |  | 9 |
|  |  | ERN1 | 0 | 0 |  | 9 |
|  |  | GLUL | 0 | 0 |  | 9 |
|  |  | TLR4 | 0 | 0 |  | 9 |
|  |  | TLR9 | 0 | 0 |  | 9 |
|  |  | ICAM1 | 0 | 0 |  | 9 |
|  |  | CYP11B1 | 2.558139535 | 2 | Clustered | 8 |
|  |  | CYP11B2 | 2.558139535 | 2 | Clustered | 8 |
|  |  | CYP2D6 | 2.558139535 | 2 | Clustered | 8 |
|  |  | CYP2C19 | 2.558139535 | 2 | Clustered | 8 |
|  |  | CAMK2B | 0 | 0 |  | 8 |
|  |  | FABP5 | 0 | 0 |  | 8 |
|  |  | SLC6A4 | 0 | 0 |  | 8 |
|  |  | PFKFB3 | 0 | 0 |  | 8 |
|  |  | GRIA1 | 0 | 0 |  | 8 |
|  |  | CPT1A | 0 | 0 |  | 8 |
|  |  | MAST3 | 0 | 0 |  | 8 |
|  |  | MMP14 | 0 | 0 |  | 8 |
|  |  | TYRO3 | 0 | 0 |  | 8 |
|  |  | PLAT | 0 | 0 |  | 8 |
|  |  | CALCRL | 3.444444444 | 1 | Clustered | 7 |
|  |  | TAAR1 | 3.444444444 | 1 | Clustered | 7 |
|  |  | CYP24A1 | 2.558139535 | 2 | Clustered | 7 |
|  |  | GABRB3 | 1 | 10 | Clustered | 7 |
|  |  | BRD4 | 0 | 0 |  | 7 |
|  |  | SAE1 | 0 | 0 |  | 7 |
|  |  | HDAC10 | 0 | 0 |  | 7 |
|  |  | BPTF | 0 | 0 |  | 7 |
|  |  | HMGCR | 0 | 0 |  | 7 |
|  |  | BACE1 | 0 | 0 |  | 7 |
|  |  | SLC29A1 | 0 | 0 |  | 7 |
|  |  | FDFT1 | 0 | 0 |  | 7 |
|  |  | FLT1 | 0 | 0 |  | 7 |
|  |  | FABP1 | 0 | 0 |  | 7 |
|  |  | MMP1 | 0 | 0 |  | 7 |
|  |  | GLO1 | 0 | 0 |  | 7 |
|  |  | FABP4 | 0 | 0 |  | 7 |
|  |  | COMT | 0 | 0 |  | 7 |
|  |  | GABRA1 | 0 | 0 |  | 7 |
|  |  | SLC6A3 | 0 | 0 |  | 7 |
|  |  | SREBF2 | 0 | 0 |  | 7 |
|  |  | NR1H4 | 0 | 0 |  | 7 |
|  |  | CCNB3 | 2.558139535 | 2 | Clustered | 6 |
|  |  | PSENEN | 1.8 | 7 | Clustered | 6 |
|  |  | GABRA3 | 1 | 10 | Clustered | 6 |
|  |  | CLK1 | 0 | 0 |  | 6 |
|  |  | EGLN1 | 0 | 0 |  | 6 |
|  |  | MMP3 | 0 | 0 |  | 6 |
|  |  | AKR1C3 | 0 | 0 |  | 6 |
|  |  | ITGB7 | 0 | 0 |  | 6 |
|  |  | TXK | 0 | 0 |  | 6 |
|  |  | CBR1 | 0 | 0 |  | 6 |
|  |  | ABCB1 | 0 | 0 |  | 6 |
|  |  | PIM2 | 0 | 0 |  | 6 |
|  |  | KCNH2 | 0 | 0 |  | 6 |
|  |  | NR3C2 | 0 | 0 |  | 6 |
|  |  | NAMPT | 0 | 0 |  | 6 |
|  |  | APH1B | 1.8 | 7 | Clustered | 5 |
|  |  | APH1A | 1.8 | 7 | Seed | 5 |
|  |  | CHRNA3 | 1.25 | 9 | Clustered | 5 |
|  |  | F3 | 0 | 0 |  | 5 |
|  |  | HTR3A | 0 | 0 |  | 5 |
|  |  | HSD17B3 | 0 | 0 |  | 5 |
|  |  | FNTA | 0 | 0 |  | 5 |
|  |  | TNKS | 0 | 0 |  | 5 |
|  |  | ABCC1 | 0 | 0 |  | 5 |
|  |  | NEK6 | 0 | 0 |  | 5 |
|  |  | TYMS | 0 | 0 |  | 5 |
|  |  | NR1I3 | 0 | 0 |  | 5 |
|  |  | ITGAL | 0 | 0 |  | 5 |
|  |  | IDH1 | 0 | 0 |  | 5 |
|  |  | MMP9 | 0 | 0 |  | 5 |
|  |  | ARG1 | 0 | 0 |  | 5 |
|  |  | ERCC5 | 0 | 0 |  | 5 |
|  |  | PDE4B | 0 | 0 |  | 5 |
|  |  | CHRNB2 | 1.25 | 9 | Clustered | 4 |
|  |  | CHRNB4 | 1.25 | 9 | Clustered | 4 |
|  |  | GABRA5 | 1 | 10 | Seed | 4 |
|  |  | GABRA2 | 0 | 0 |  | 4 |
|  |  | DHCR7 | 0 | 0 |  | 4 |
|  |  | CTSK | 0 | 0 |  | 4 |
|  |  | IDO1 | 0 | 0 |  | 4 |
|  |  | PDE4A | 0 | 0 |  | 4 |
|  |  | PTPRS | 0 | 0 |  | 4 |
|  |  | NUAK1 | 0 | 0 |  | 4 |
|  |  | HDAC11 | 0 | 0 |  | 4 |
|  |  | ABCG2 | 0 | 0 |  | 4 |
|  |  | F10 | 0 | 0 |  | 4 |
|  |  | PIM3 | 0 | 0 |  | 4 |
|  |  | FNTB | 0 | 0 |  | 4 |
|  |  | TTR | 0 | 0 |  | 4 |
|  |  | CPT2 | 0 | 0 |  | 4 |
|  |  | P2RX7 | 0 | 0 |  | 4 |
|  |  | MMP13 | 0 | 0 |  | 4 |
|  |  | CHRNA4 | 1.25 | 9 | Seed | 3 |
|  |  | RORC | 0 | 0 |  | 3 |
|  |  | PLA2G2A | 0 | 0 |  | 3 |
|  |  | POLB | 0 | 0 |  | 3 |
|  |  | PDE9A | 0 | 0 |  | 3 |
|  |  | HDAC8 | 0 | 0 |  | 3 |
|  |  | SCN5A | 0 | 0 |  | 3 |
|  |  | ELANE | 0 | 0 |  | 3 |
|  |  | ATP12A | 0 | 0 |  | 3 |
|  |  | UBA2 | 0 | 0 |  | 3 |
|  |  | MALT1 | 0 | 0 |  | 3 |
|  |  | HSD11B2 | 0 | 0 |  | 3 |
|  |  | PREP | 0 | 0 |  | 3 |
|  |  | NOX4 | 0 | 0 |  | 3 |
|  |  | KCNK3 | 0 | 0 |  | 3 |
|  |  | LNPEP | 0 | 0 |  | 3 |
|  |  | GLRA1 | 0 | 0 |  | 3 |
|  |  | SCN2A | 0 | 0 |  | 3 |
|  |  | ALPL | 0 | 0 |  | 3 |
|  |  | TNKS2 | 0 | 0 |  | 3 |
|  |  | LSS | 0 | 0 |  | 3 |
|  |  | SQLE | 0 | 0 |  | 3 |
|  |  | KCNQ2 | 0 | 0 |  | 3 |
|  |  | CTSL | 0 | 0 |  | 3 |
|  |  | SIGMAR1 | 0 | 0 |  | 3 |
|  |  | DHODH | 0 | 0 |  | 2 |
|  |  | CA14 | 0 | 0 |  | 2 |
|  |  | MPO | 0 | 0 |  | 2 |
|  |  | SLC18A2 | 0 | 0 |  | 2 |
|  |  | PRF1 | 0 | 0 |  | 2 |
|  |  | RORA | 0 | 0 |  | 2 |
|  |  | FABP3 | 0 | 0 |  | 2 |
|  |  | PGGT1B | 0 | 0 |  | 2 |
|  |  | KCNK9 | 0 | 0 |  | 2 |
|  |  | NAAA | 0 | 0 |  | 2 |
|  |  | SHBG | 0 | 0 |  | 2 |
|  |  | GSTM2 | 0 | 0 |  | 2 |
|  |  | HSD17B14 | 0 | 0 |  | 2 |
|  |  | GUSB | 0 | 0 |  | 2 |
|  |  | UGT2B7 | 0 | 0 |  | 2 |
|  |  | CA2 | 0 | 0 |  | 2 |
|  |  | AMY1A | 0 | 0 |  | 2 |
|  |  | MMP26 | 0 | 0 |  | 2 |
|  |  | CA3 | 0 | 0 |  | 2 |
|  |  | ALOX15 | 0 | 0 |  | 2 |
|  |  | PDE10A | 0 | 0 |  | 2 |
|  |  | ODC1 | 0 | 0 |  | 2 |
|  |  | XDH | 0 | 0 |  | 2 |
|  |  | ANPEP | 0 | 0 |  | 2 |
|  |  | SCN10A | 0 | 0 |  | 2 |
|  |  | MAOA | 0 | 0 |  | 2 |
|  |  | PLA2G1B | 0 | 0 |  | 1 |
|  |  | MPEG1 | 0 | 0 |  | 1 |
|  |  | PTGES | 0 | 0 |  | 1 |
|  |  | SLC22A6 | 0 | 0 |  | 1 |
|  |  | NOX1 | 0 | 0 |  | 1 |
|  |  | PDE4C | 0 | 0 |  | 1 |
|  |  | HSD17B1 | 0 | 0 |  | 1 |
|  |  | KCNA3 | 0 | 0 |  | 1 |
|  |  | TYR | 0 | 0 |  | 1 |
|  |  | TRPA1 | 0 | 0 |  | 1 |
|  |  | CTSS | 0 | 0 |  | 1 |
|  |  | SCN9A | 0 | 0 |  | 1 |
|  |  | MMP15 | 0 | 0 |  | 1 |
|  |  | ALOX15B | 0 | 0 |  | 1 |
|  |  | PTGS1 | 0 | 0 |  | 1 |
|  |  | ADCY5 | 0 | 0 |  | 1 |
|  |  | CES1 | 0 | 0 |  | 1 |
|  |  | MAOB | 0 | 0 |  | 1 |
|  |  | CES2 | 0 | 0 |  | 1 |
|  |  | STS | 0 | 0 |  | 1 |
|  |  | PLA2G7 | 0 | 0 |  | 1 |
|  |  | SLC6A2 | 0 | 0 |  | 1 |
|  |  | CACNA1G | 0 | 0 |  | 1 |
|  |  | MMP12 | 0 | 0 |  | 1 |
|  |  | TRPM8 | 0 | 0 |  | 1 |
|  |  | ALOX12 | 0 | 0 |  | 1 |
|  |  | PNMT | 0 | 0 |  | 1 |
|  |  | PAOX | 0 | 0 |  | 1 |
|  |  | GPR35 | 0 | 0 |  | 1 |
|  |  | SLC6A9 | 0 | 0 |  | 1 |
|  |  | KCNQ3 | 0 | 0 |  | 1 |
|  |  | HSD11B1 | 0 | 0 |  | 1 |
|  |  | SLC5A7 | 0 | 0 |  | 1 |
|  |  | UPP1 | 0 | 0 |  | 1 |
|  |  | TBXAS1 | 0 | 0 |  | 1 |
|  |  | HPGDS | 0 | 0 |  | 1 |
|  |  | CA4 | 0 | 0 |  | 1 |
|  |  | SLC5A1 | 0 | 0 |  | 1 |
|  |  | ACHE | 0 | 0 |  | 1 |
|  |  | CD38 | 0 | 0 |  | 1 |
|  | VMC-related targets | SRC | 3.5 | 2 | Clustered | 39 |
|  |  | EGFR | 3.5 | 2 | Seed | 35 |
|  |  | HSP90AA1 | 3.5 | 2 | Clustered | 34 |
|  |  | PRKCA | 2.545454545 | 1 | Clustered | 34 |
|  |  | MAPK1 | 3.5 | 2 | Clustered | 29 |
|  |  | MAPK3 | 3.5 | 2 | Clustered | 27 |
|  |  | MAPK14 | 1.5 | 3 | Clustered | 27 |
|  |  | PTK2 | 3.5 | 2 | Clustered | 25 |
|  |  | AKT1 | 2.545454545 | 1 | Clustered | 23 |
|  |  | PRKACA | 2.545454545 | 1 | Seed | 23 |
|  |  | LCK | 3.5 | 2 | Clustered | 20 |
|  |  | PTPN11 | 2.545454545 | 1 | Clustered | 20 |
|  |  | FYN | 2.545454545 | 1 | Clustered | 17 |
|  |  | BCL2 | 1.5 | 3 | Clustered | 17 |
|  |  | RET | 3.5 | 2 | Clustered | 16 |
|  |  | MAPK8 | 2.545454545 | 1 | Clustered | 16 |
|  |  | TNF | 1.5 | 3 | Seed | 16 |
|  |  | CASP3 | 0 | 0 |  | 16 |
|  |  | MET | 2.545454545 | 1 | Clustered | 15 |
|  |  | ABL1 | 2.545454545 | 1 | Clustered | 15 |
|  |  | RAF1 | 2.545454545 | 1 | Clustered | 14 |
|  |  | PPARG | 2.545454545 | 1 | Clustered | 14 |
|  |  | PARP1 | 1.5 | 3 | Clustered | 13 |
|  |  | IKBKB | 0 | 0 |  | 13 |
|  |  | NOS3 | 0 | 0 |  | 13 |
|  |  | PIK3CA | 2.545454545 | 1 | Clustered | 12 |
|  |  | RPS6KB1 | 1.5 | 3 | Clustered | 12 |
|  |  | MTOR | 0 | 0 |  | 11 |
|  |  | PSEN1 | 0 | 0 |  | 9 |
|  |  | FASN | 0 | 0 |  | 9 |
|  |  | PPARA | 1.5 | 3 | Clustered | 8 |
|  |  | EIF2AK2 | 1.5 | 3 | Clustered | 8 |
|  |  | ITGB2 | 0 | 0 |  | 8 |
|  |  | PIK3CG | 0 | 0 |  | 8 |
|  |  | ADAM17 | 1.5 | 3 | Clustered | 7 |
|  |  | F2 | 0 | 0 |  | 7 |
|  |  | NOS2 | 0 | 0 |  | 7 |
|  |  | VDR | 0 | 0 |  | 7 |
|  |  | BLK | 0 | 0 |  | 7 |
|  |  | ICAM1 | 0 | 0 |  | 6 |
|  |  | S1PR1 | 0 | 0 |  | 6 |
|  |  | ITK | 0 | 0 |  | 6 |
|  |  | CCND1 | 0 | 0 |  | 6 |
|  |  | CASP7 | 0 | 0 |  | 6 |
|  |  | PLG | 0 | 0 |  | 6 |
|  |  | MMP1 | 0 | 0 |  | 6 |
|  |  | DRD2 | 1 | 4 | Clustered | 5 |
|  |  | CHRM2 | 1 | 4 | Clustered | 5 |
|  |  | IL2 | 0 | 0 |  | 5 |
|  |  | PSEN2 | 0 | 0 |  | 5 |
|  |  | MIF | 0 | 0 |  | 5 |
|  |  | EPHA5 | 0 | 0 |  | 4 |
|  |  | PLAT | 0 | 0 |  | 4 |
|  |  | TLR4 | 0 | 0 |  | 4 |
|  |  | FLT1 | 0 | 0 |  | 4 |
|  |  | SLC6A4 | 0 | 0 |  | 4 |
|  |  | ITGA4 | 0 | 0 |  | 4 |
|  |  | MMP2 | 0 | 0 |  | 4 |
|  |  | FDFT1 | 0 | 0 |  | 4 |
|  |  | IL6 | 0 | 0 |  | 4 |
|  |  | HRH1 | 0 | 0 |  | 4 |
|  |  | CXCR3 | 1 | 4 | Seed | 3 |
|  |  | F3 | 0 | 0 |  | 3 |
|  |  | ELANE | 0 | 0 |  | 3 |
|  |  | ALOX5 | 0 | 0 |  | 3 |
|  |  | KCNH2 | 0 | 0 |  | 3 |
|  |  | G6PD | 0 | 0 |  | 3 |
|  |  | TLR9 | 0 | 0 |  | 3 |
|  |  | CYP51A1 | 0 | 0 |  | 3 |
|  |  | ITGAL | 0 | 0 |  | 3 |
|  |  | ABCB1 | 0 | 0 |  | 2 |
|  |  | MMP9 | 0 | 0 |  | 2 |
|  |  | PRF1 | 0 | 0 |  | 2 |
|  |  | NR3C2 | 0 | 0 |  | 2 |
|  |  | MMP3 | 0 | 0 |  | 2 |
|  |  | ABCC1 | 0 | 0 |  | 1 |
|  |  | SCN5A | 0 | 0 |  | 1 |
|  |  | ARG1 | 0 | 0 |  | 1 |
|  |  | IDO1 | 0 | 0 |  | 1 |
|  |  | CYP2C19 | 0 | 0 |  | 1 |
|  |  | CPT2 | 0 | 0 |  | 1 |
|  |  | CTSL | 0 | 0 |  | 1 |
|  |  | MPO | 0 | 0 |  | 1 |
|  |  | ADCY5 | 0 | 0 |  | 1 |
|  |  | ADORA2A | 0 | 0 |  | 1 |
|  |  | SLC18A2 | 0 | 0 |  | 1 |

| **Table S10. Tissue- and cell-specific location of targets** | | | | | | | | | | |
| --- | --- | --- | --- | --- | --- | --- | --- | --- | --- | --- |
| **Herb** | **Target type** | **GO** | **Category** | **Description** | **LogP** | **Enrichment** | **Z-score** | **GeneID** | **Hits** | **Log(q-value)** |
| HQ | whole targets | PGB:00045 | PaGenBase | Tissue-specific: placenta | -7.6 | 4.5 | 7.5 | 251|412|649|898|1017|1588|1612|1956|2321|3292|3294|3480|5328|6513|6530|6532|6573|7046|7430|9536 | ALPG|STS|BMP1|CCNE1|CDK2|CYP19A1|DAPK1|EGFR|FLT1|HSD17B1|HSD17B2|IGF1R|PLAU|SLC2A1|SLC6A2|SLC6A4|SLC19A1|TGFBR1|EZR|PTGES | -6.3 |
|  |  | PGB:00002 | PaGenBase | Cell-specific: HEPG2 | -6.5 | 3.6 | 6.5 | 763\|866\|993\|1543\|1845\|2147\|2152\|2168\|3294\|4233\|5140\|5143\|5770\|5781\|6198\|6532\|6573\|7276\|7364\|9734\|9971\|51564 | CA5A\|SERPINA6\|CDC25A\|CYP1A1\|DUSP3\|F2\|F3\|FABP1\|HSD17B2\|MET\|PDE3B\|PDE4C\|PTPN1\|PTPN11\|RPS6KB1\|SLC6A4\|SLC19A1\|TTR\|UGT2B7\|HDAC9\|NR1H4\|HDAC7 | -5.2 |
|  |  | PGB:00001 | PaGenBase | Tissue-specific: liver | -6.4 | 2.9 | 6.1 | 383\|590\|763\|866\|1109\|1312\|1557\|1584\|1610\|1645\|1723\|1956\|2147\|2152\|2159\|2168\|3290\|3294\|4255\|5340\|5444\|6462\|6530\|7498\|8824\|9970\|9971\|29881\|79054 | ARG1\|BCHE\|CA5A\|SERPINA6\|AKR1C4\|COMT\|CYP2C19\|CYP11B1\|DAO\|AKR1C1\|DHODH\|EGFR\|F2\|F3\|F10\|FABP1\|HSD11B1\|HSD17B2\|MGMT\|PLG\|PON1\|SHBG\|SLC6A2\|XDH\|CES2\|NR1I3\|NR1H4\|NPC1L1\|TRPM8 | -5.1 |
|  |  | PGB:00049 | PaGenBase | Cell-specific: Adipocyte | -5.8 | 5.7 | 6.9 | 2152\|2167\|4128\|4311\|4313\|4321\|5156\|5159\|5468\|6319\|10062\|11096 | F3\|FABP4\|MAOA\|MME\|MMP2\|MMP12\|PDGFRA\|PDGFRB\|PPARG\|SCD\|NR1H3\|ADAMTS5 | -4.6 |
|  |  | PGB:00031 | PaGenBase | Cell-specific: HUVEC | -5.6 | 3.5 | 5.9 | 558\|595\|840\|902\|1024\|2048\|2152\|2321\|2561\|3265\|4312\|4321\|5139\|5340\|5999\|6095\|7046\|7299\|23450 | AXL\|CCND1\|CASP7\|CCNH\|CDK8\|EPHB2\|F3\|FLT1\|GABRB2\|HRAS\|MMP1\|MMP12\|PDE3A\|PLG\|RGS4\|RORA\|TGFBR1\|TYR\|SF3B3 | -4.4 |
|  |  | PGB:00008 | PaGenBase | Tissue-specific: small intestine | -5.5 | 5.9 | 6.8 | 251\|1268\|2101\|2169\|4311\|4312\|4321\|6523\|9153\|29881\|57016 | ALPG\|CNR1\|ESRRA\|FABP2\|MME\|MMP1\|MMP12\|SLC5A1\|SLC28A2\|NPC1L1\|AKR1B10 | -4.4 |
|  |  | PGB:00048 | PaGenBase | Tissue-specific: bone marrow | -5.2 | 4.2 | 5.9 | 890\|1511\|1991\|2099\|2870\|4317\|4353\|5144\|6850\|6916\|9212\|10013\|64386\|81027 | CCNA2\|CTSG\|ELANE\|ESR1\|GRK6\|MMP8\|MPO\|PDE4D\|SYK\|TBXAS1\|AURKB\|HDAC6\|MMP25\|TUBB1 | -4 |
|  |  | PGB:00018 | PaGenBase | Tissue-specific: lung | -4.8 | 2.9 | 5.2 | 218\|246\|247\|762\|765\|1215\|1543\|1991\|3725\|3815\|4233\|4317\|4322\|4353\|5319\|5743\|5771\|6532\|27159\|81027\|134864 | ALDH3A1\|ALOX15\|ALOX15B\|CA4\|CA6\|CMA1\|CYP1A1\|ELANE\|JUN\|KIT\|MET\|MMP8\|MMP13\|MPO\|PLA2G1B\|PTGS2\|PTPN2\|SLC6A4\|CHIA\|TUBB1\|TAAR1 | -3.7 |
|  |  | PGB:00014 | PaGenBase | Cell-specific: DRG | -4.3 | 2.9 | 4.9 | 43\|102\|624\|760\|1268\|1545\|1588\|2166\|2167\|2555\|3062\|3293\|4137\|4914\|5468\|5979\|6530\|8900\|10062 | ACHE\|ADAM10\|BDKRB2\|CA2\|CNR1\|CYP1B1\|CYP19A1\|FAAH\|FABP4\|GABRA2\|HCRTR2\|HSD17B3\|MAPT\|NTRK1\|PPARG\|RET\|SLC6A2\|CCNA1\|NR1H3 | -3.3 |
|  |  | PGB:00078 | PaGenBase | Cell-specific: liver cell | -4.3 | 6.2 | 5.9 | 1646\|2147\|2152\|2167\|2168\|7276\|7364\|8644 | AKR1C2\|F2\|F3\|FABP4\|FABP1\|TTR\|UGT2B7\|AKR1C3 | -3.2 |
|  |  | PGB:00011 | PaGenBase | Tissue-specific: spleen | -4.3 | 2.8 | 4.8 | 251\|1269\|2208\|3359\|3577\|4312\|4318\|4321\|4325\|5293\|5294\|5551\|5731\|5777\|6916\|10062\|11040\|54106\|64386\|81027 | ALPG\|CNR2\|FCER2\|HTR3A\|CXCR1\|MMP1\|MMP9\|MMP12\|MMP16\|PIK3CD\|PIK3CG\|PRF1\|PTGER1\|PTPN6\|TBXAS1\|NR1H3\|PIM2\|TLR9\|MMP25\|TUBB1 | -3.2 |
|  |  | PGB:00072 | PaGenBase | Tissue-specific: uterus | -3.7 | 5.8 | 5.3 | 1511\|2099\|2260\|3558\|4316\|4321\|10846 | CTSG\|ESR1\|FGFR1\|IL2\|MMP7\|MMP12\|PDE10A | -2.7 |
|  |  | PGB:00087 | PaGenBase | Cell-specific: Lymphoma burkitts Raji | -3.7 | 4.4 | 4.9 | 590\|1269\|2033\|2208\|4282\|7421\|9970\|10783\|64386 | BCHE\|CNR2\|EP300\|FCER2\|MIF\|VDR\|NR1I3\|NEK6\|MMP25 | -2.6 |
|  |  | PGB:00057 | PaGenBase | Tissue-specific: ovary | -3.5 | 3.3 | 4.5 | 1588\|2048\|2099\|2100\|2561\|2864\|3290\|8972\|10054\|10846\|29881\|134864 | CYP19A1\|EPHB2\|ESR1\|ESR2\|GABRB2\|FFAR1\|HSD11B1\|MGAM\|UBA2\|PDE10A\|NPC1L1\|TAAR1 | -2.5 |
|  |  | PGB:00065 | PaGenBase | Tissue-specific: Breast | -3.4 | 4.1 | 4.7 | 231\|367\|1646\|3356\|3725\|4233\|5021\|6319\|116085 | AKR1B1\|AR\|AKR1C2\|HTR2A\|JUN\|MET\|OXTR\|SCD\|SLC22A12 | -2.4 |
|  |  | PGB:00192 | PaGenBase | Cell-specific: GM133 | -3.1 | 16 | 6.5 | 4311\|4321\|5467 | MME\|MMP12\|PPARD | -2.2 |
|  |  | PGB:00047 | PaGenBase | Cell-specific: THY+ | -2.9 | 4.3 | 4.3 | 239\|816\|873\|2099\|6714\|7046\|9149 | ALOX12\|CAMK2B\|CBR1\|ESR1\|SRC\|TGFBR1\|DYRK1B | -2 |
|  |  | PGB:00121 | PaGenBase | Cell-specific: CD34+ | -2.7 | 7.6 | 4.8 | 1511\|1991\|3815\|4353 | CTSG\|ELANE\|KIT\|MPO | -1.8 |
|  |  | PGB:00120 | PaGenBase | Cell-specific: Cardiac Myocytes | -2.7 | 5.7 | 4.4 | 558\|4312\|5327\|5999\|10769 | AXL\|MMP1\|PLAT\|RGS4\|PLK2 | -1.8 |
|  |  | PGB:00079 | PaGenBase | Tissue-specific: Caudate nucleus | -2.5 | 6.7 | 4.4 | 135\|2100\|3358\|10846 | ADORA2A\|ESR2\|HTR2C\|PDE10A | -1.6 |
|  |  | PGB:00115 | PaGenBase | Cell-specific: CD33+ Myeloid | -2.5 | 6.7 | 4.4 | 1263\|3725\|4170\|120892 | PLK3\|JUN\|MCL1\|LRRK2 | -1.6 |
|  |  | PGB:00015 | PaGenBase | Tissue-specific: Smooth Muscle | -2.4 | 4 | 3.7 | 136\|558\|4312\|5742\|5743\|10769 | ADORA2B\|AXL\|MMP1\|PTGS1\|PTGS2\|PLK2 | -1.5 |
|  |  | PGB:00081 | PaGenBase | Cell-specific: Bronchial Epithelial Cells | -2.4 | 3.5 | 3.6 | 771\|1956\|2152\|4233\|5328\|5743\|5792 | CA12\|EGFR\|F3\|MET\|PLAU\|PTGS2\|PTPRF | -1.5 |
|  |  | PGB:00080 | PaGenBase | Cell-specific: huh-7 | -2.4 | 8.9 | 4.6 | 2168\|7276\|9971 | FABP1\|TTR\|NR1H4 | -1.5 |
|  |  | PGB:00068 | PaGenBase | Tissue-specific: Stomach | -2.3 | 5.7 | 4 | 768\|4282\|9153\|27159 | CA9\|MIF\|SLC28A2\|CHIA | -1.4 |
|  |  | PGB:00028 | PaGenBase | Tissue-specific: prostate cancer | -2.1 | 5 | 3.6 | 4363\|5139\|6198\|8654 | ABCC1\|PDE3A\|RPS6KB1\|PDE5A | -1.2 |
|  |  | PGB:00007 | PaGenBase | Tissue-specific: colon | -1.9 | 2.2 | 2.8 | 759\|760\|766\|840\|1080\|2169\|2859\|5143\|5979\|7015\|9153 | CA1\|CA2\|CA7\|CASP7\|CFTR\|FABP2\|GPR35\|PDE4C\|RET\|TERT\|SLC28A2 | -1.1 |
|  |  | PGB:00060 | PaGenBase | Tissue-specific: retinoblastoma | -1.9 | 3.5 | 3 | 4312\|4314\|4843\|7298\|7421 | MMP1\|MMP3\|NOS2\|TYMS\|VDR | -1 |
|  |  | PGB:00063 | PaGenBase | Cell-specific: OVR278S | -1.8 | 4.2 | 3.1 | 2260\|4638\|5465\|8658 | FGFR1\|MYLK\|PPARA\|TNKS | -0.97 |
|  |  | PGB:00095 | PaGenBase | Cell-specific: LNCAP | -1.8 | 5.6 | 3.4 | 231\|367\|4128 | AKR1B1\|AR\|MAOA | -0.95 |
|  |  | PGB:00067 | PaGenBase | Cell-specific: Brain cell | -1.8 | 4.1 | 3.1 | 4313\|5156\|5328\|5743 | MMP2\|PDGFRA\|PLAU\|PTGS2 | -0.95 |
|  |  | PGB:00073 | PaGenBase | Tissue-specific: salivary gland | -1.7 | 2.8 | 2.7 | 765\|5578\|5802\|6573\|9149\|10381 | CA6\|PRKCA\|PTPRS\|SLC19A1\|DYRK1B\|TUBB3 | -0.85 |
|  |  | PGB:00101 | PaGenBase | Tissue-specific: Colorectal adenocarcinoma | -1.7 | 5 | 3.1 | 595\|6790\|7153 | CCND1\|AURKA\|TOP2A | -0.84 |
|  |  | PGB:00041 | PaGenBase | Tissue-specific: Blood | -1.6 | 2.3 | 2.5 | 1520\|2358\|3577\|5293\|5586\|5777\|5836\|65220 | CTSS\|FPR2\|CXCR1\|PIK3CD\|PKN2\|PTPN6\|PYGL\|NADK | -0.8 |
|  |  | PGB:00022 | PaGenBase | Tissue-specific: adrenal gland | -1.5 | 2.4 | 2.4 | 1584\|1586\|2208\|2350\|5156\|5243\|6095 | CYP11B1\|CYP17A1\|FCER2\|FOLR2\|PDGFRA\|ABCB1\|RORA | -0.73 |
|  |  | PGB:00016 | PaGenBase | Tissue-specific: thymus | -1.4 | 2.3 | 2.3 | 135\|2322\|3932\|4012\|4321\|9612\|11238 | ADORA2A\|FLT3\|LCK\|LNPEP\|MMP12\|NCOR2\|CA5B | -0.64 |
|  |  | PGB:00050 | PaGenBase | Tissue-specific: trachea | -1.3 | 3 | 2.3 | 218\|246\|328\|1543 | ALDH3A1\|ALOX15\|APEX1\|CYP1A1 | -0.54 |
|  |  | PGB:00032 | PaGenBase | Tissue-specific: cortex | -1.3 | 3.7 | 2.4 | 2561\|3064\|6532 | GABRB2\|HTT\|SLC6A4 | -0.51 |
|  | VMC-related targets | PGB:00031 | PaGenBase | Cell-specific: HUVEC | -6.2 | 9.2 | 8.2 | 595\|840\|2152\|2321\|3265\|4312\|5340\|7046\|7299 | CCND1\|CASP7\|F3\|FLT1\|HRAS\|MMP1\|PLG\|TGFBR1\|TYR | -4.8 |
|  |  | PGB:00018 | PaGenBase | Tissue-specific: lung | -6.1 | 7.6 | 7.6 | 1215\|1543\|1991\|3725\|3815\|4233\|4317\|4353\|6532\|81027 | CMA1\|CYP1A1\|ELANE\|JUN\|KIT\|MET\|MMP8\|MPO\|SLC6A4\|TUBB1 | -4.8 |
|  |  | PGB:00121 | PaGenBase | Cell-specific: CD34+ | -5.6 | 42 | 13 | 1511\|1991\|3815\|4353 | CTSG\|ELANE\|KIT\|MPO | -4.3 |
|  |  | PGB:00060 | PaGenBase | Tissue-specific: retinoblastoma | -3.9 | 16 | 7.4 | 4312\|4314\|4843\|7421 | MMP1\|MMP3\|NOS2\|VDR | -2.7 |
|  |  | PGB:00002 | PaGenBase | Cell-specific: HEPG2 | -3.9 | 6.2 | 5.6 | 1543\|2147\|2152\|4233\|5781\|6198\|6532 | CYP1A1\|F2\|F3\|MET\|PTPN11\|RPS6KB1\|SLC6A4 | -2.7 |
|  |  | PGB:00048 | PaGenBase | Tissue-specific: bone marrow | -3.5 | 8.3 | 5.7 | 1511\|1991\|4317\|4353\|81027 | CTSG\|ELANE\|MMP8\|MPO\|TUBB1 | -2.3 |
|  |  | PGB:00045 | PaGenBase | Tissue-specific: placenta | -2.9 | 6.2 | 4.7 | 1956\|2321\|6530\|6532\|7046 | EGFR\|FLT1\|SLC6A2\|SLC6A4\|TGFBR1 | -1.8 |
|  |  | PGB:00014 | PaGenBase | Cell-specific: DRG | -2.9 | 5 | 4.4 | 43\|624\|4914\|5468\|5979\|6530 | ACHE\|BDKRB2\|NTRK1\|PPARG\|RET\|SLC6A2 | -1.8 |
|  |  | PGB:00011 | PaGenBase | Tissue-specific: spleen | -2.7 | 4.5 | 4.1 | 4312\|4318\|5294\|5551\|54106\|81027 | MMP1\|MMP9\|PIK3CG\|PRF1\|TLR9\|TUBB1 | -1.6 |
|  |  | PGB:00001 | PaGenBase | Tissue-specific: liver | -2.6 | 3.8 | 3.9 | 383\|1557\|1956\|2147\|2152\|5340\|6530 | ARG1\|CYP2C19\|EGFR\|F2\|F3\|PLG\|SLC6A2 | -1.5 |
|  |  | PGB:00036 | PaGenBase | Cell-specific: MOLT4 | -2.2 | 8.3 | 4.4 | 3932\|4312\|5578 | LCK\|MMP1\|PRKCA | -1.2 |
|  |  | PGB:00081 | PaGenBase | Cell-specific: Bronchial Epithelial Cells | -2.2 | 8.3 | 4.4 | 1956\|2152\|4233 | EGFR\|F3\|MET | -1.2 |
|  |  | PGB:00049 | PaGenBase | Cell-specific: Adipocyte | -2.2 | 7.8 | 4.2 | 2152\|4313\|5468 | F3\|MMP2\|PPARG | -1.1 |
|  |  | PGB:00010 | PaGenBase | Tissue-specific: adipose tissue | -2 | 6.5 | 3.8 | 383\|1215\|4353 | ARG1\|CMA1\|MPO | -0.89 |
|  |  | PGB:00052 | PaGenBase | Cell-specific: HELA | -1.9 | 6 | 3.6 | 43\|624\|4314 | ACHE\|BDKRB2\|MMP3 | -0.8 |
| YGZ | whole targets | PGB:00014 | PaGenBase | Cell-specific: DRG | -8.3 | 4.4 | 7.8 | 43|760|1268|1545|1588|1675|2166|2167|2246|2346|2555|3293|3357|3362|4137|5170|5468|5733|6530|7134|7137|8900|10062 | ACHE|CA2|CNR1|CYP1B1|CYP19A1|CFD|FAAH|FABP4|FGF1|FOLH1|GABRA2|HSD17B3|HTR2B|HTR6|MAPT|PDPK1|PPARG|PTGER3|SLC6A2|TNNC1|TNNI3|CCNA1|NR1H3 | -7 |
|  |  | PGB:00095 | PaGenBase | Cell-specific: LNCAP | -8.1 | 19 | 12 | 150\|231\|367\|2346\|3291\|3817\|4128\|5737 | ADRA2A\|AKR1B1\|AR\|FOLH1\|HSD11B2\|KLK2\|MAOA\|PTGFR | -6.7 |
|  |  | PGB:00045 | PaGenBase | Tissue-specific: placenta | -6.2 | 4.5 | 6.7 | 412\|898\|1017\|1588\|1612\|1956\|2321\|2324\|3292\|3294\|3480\|5054\|5228\|6530\|6532\|9536 | STS\|CCNE1\|CDK2\|CYP19A1\|DAPK1\|EGFR\|FLT1\|FLT4\|HSD17B1\|HSD17B2\|IGF1R\|SERPINE1\|PGF\|SLC6A2\|SLC6A4\|PTGES | -5 |
|  |  | PGB:00001 | PaGenBase | Tissue-specific: liver | -6.2 | 3.1 | 6.1 | 146\|148\|383\|590\|763\|866\|1109\|1557\|1565\|1610\|1645\|1956\|2147\|2159\|2168\|2645\|3290\|3294\|5340\|6462\|6530\|7498\|8824\|9970\|29881 | ADRA1D\|ADRA1A\|ARG1\|BCHE\|CA5A\|SERPINA6\|AKR1C4\|CYP2C19\|CYP2D6\|DAO\|AKR1C1\|EGFR\|F2\|F10\|FABP1\|GCK\|HSD11B1\|HSD17B2\|PLG\|SHBG\|SLC6A2\|XDH\|CES2\|NR1I3\|NPC1L1 | -5 |
|  |  | PGB:00079 | PaGenBase | Tissue-specific: Caudate nucleus | -5.1 | 13 | 8.1 | 135\|1812\|2100\|3358\|10733\|10846 | ADORA2A\|DRD1\|ESR2\|HTR2C\|PLK4\|PDE10A | -4 |
|  |  | PGB:00078 | PaGenBase | Cell-specific: liver cell | -5 | 7.7 | 6.9 | 1646\|2147\|2167\|2168\|7276\|7364\|8644\|10188 | AKR1C2\|F2\|FABP4\|FABP1\|TTR\|UGT2B7\|AKR1C3\|TNK2 | -3.9 |
|  |  | PGB:00065 | PaGenBase | Tissue-specific: Breast | -5 | 5.7 | 6.3 | 231\|367\|1591\|1646\|2064\|3356\|4233\|5021\|6319\|116085 | AKR1B1\|AR\|CYP24A1\|AKR1C2\|ERBB2\|HTR2A\|MET\|OXTR\|SCD\|SLC22A12 | -3.9 |
|  |  | PGB:00002 | PaGenBase | Cell-specific: HEPG2 | -5 | 3.5 | 5.5 | 152\|763\|866\|993\|1591\|2147\|2168\|3291\|3294\|4233\|5140\|5770\|5781\|6198\|6532\|7276\|7364 | ADRA2C\|CA5A\|SERPINA6\|CDC25A\|CYP24A1\|F2\|FABP1\|HSD11B2\|HSD17B2\|MET\|PDE3B\|PTPN1\|PTPN11\|RPS6KB1\|SLC6A4\|TTR\|UGT2B7 | -3.8 |
|  |  | PGB:00018 | PaGenBase | Tissue-specific: lung | -4.6 | 3.1 | 5.1 | 146\|151\|246\|762\|765\|1859\|3383\|4233\|4322\|4353\|5319\|5743\|5771\|6098\|6532\|7010\|10855\|81027 | ADRA1D\|ADRA2B\|ALOX15\|CA4\|CA6\|DYRK1A\|ICAM1\|MET\|MMP13\|MPO\|PLA2G1B\|PTGS2\|PTPN2\|ROS1\|SLC6A4\|TEK\|HPSE\|TUBB1 | -3.5 |
|  |  | PGB:00031 | PaGenBase | Cell-specific: HUVEC | -4.5 | 3.5 | 5.2 | 146\|148\|558\|595\|1024\|2321\|2561\|4321\|5054\|5139\|5228\|5340\|5599\|6095\|7299 | ADRA1D\|ADRA1A\|AXL\|CCND1\|CDK8\|FLT1\|GABRB2\|MMP12\|SERPINE1\|PDE3A\|PGF\|PLG\|MAPK8\|RORA\|TYR | -3.4 |
|  |  | PGB:00048 | PaGenBase | Tissue-specific: bone marrow | -4.1 | 4.2 | 5.2 | 890\|2099\|2268\|2529\|2870\|4353\|5144\|6850\|6916\|9212\|81027 | CCNA2\|ESR1\|FGR\|FUT7\|GRK6\|MPO\|PDE4D\|SYK\|TBXAS1\|AURKB\|TUBB1 | -3.1 |
|  |  | PGB:00049 | PaGenBase | Cell-specific: Adipocyte | -3.5 | 4.8 | 4.9 | 2167\|4128\|4313\|4321\|5159\|5468\|6319\|10062 | FABP4\|MAOA\|MMP2\|MMP12\|PDGFRB\|PPARG\|SCD\|NR1H3 | -2.5 |
|  |  | PGB:00004 | PaGenBase | Tissue-specific: kidney | -3.2 | 2.7 | 4 | 152\|553\|596\|760\|771\|983\|1591\|1815\|3291\|5465\|5599\|5733\|7364\|50507\|116085 | ADRA2C\|AVPR1B\|BCL2\|CA2\|CA12\|CDK1\|CYP24A1\|DRD4\|HSD11B2\|PPARA\|MAPK8\|PTGER3\|UGT2B7\|NOX4\|SLC22A12 | -2.3 |
|  |  | PGB:00067 | PaGenBase | Cell-specific: Brain cell | -3 | 6.4 | 4.8 | 4313\|5054\|5733\|5743\|7134 | MMP2\|SERPINE1\|PTGER3\|PTGS2\|TNNC1 | -2 |
|  |  | PGB:00047 | PaGenBase | Cell-specific: THY+ | -2.7 | 4.7 | 4.2 | 239\|816\|873\|2099\|6714\|9149 | ALOX12\|CAMK2B\|CBR1\|ESR1\|SRC\|DYRK1B | -1.8 |
|  |  | PGB:00011 | PaGenBase | Tissue-specific: spleen | -2.6 | 2.4 | 3.5 | 1269\|2529\|3577\|4067\|4318\|4321\|5293\|5294\|5731\|5777\|6402\|6916\|10062\|81027 | CNR2\|FUT7\|CXCR1\|LYN\|MMP9\|MMP12\|PIK3CD\|PIK3CG\|PTGER1\|PTPN6\|SELL\|TBXAS1\|NR1H3\|TUBB1 | -1.7 |
|  |  | PGB:00100 | PaGenBase | Tissue-specific: mammary gland | -2.6 | 11 | 5.3 | 4314\|5021\|6401 | MMP3\|OXTR\|SELE | -1.7 |
|  |  | PGB:00072 | PaGenBase | Tissue-specific: uterus | -2.5 | 5.2 | 4.1 | 2099\|3357\|4321\|5733\|10846 | ESR1\|HTR2B\|MMP12\|PTGER3\|PDE10A | -1.7 |
|  |  | PGB:00062 | PaGenBase | Cell-specific: Skeletal Muscle Stromal Cells | -2.5 | 9.9 | 4.9 | 3643\|7134\|7137 | INSR\|TNNC1\|TNNI3 | -1.6 |
|  |  | PGB:00028 | PaGenBase | Tissue-specific: prostate cancer | -2.4 | 6.3 | 4.2 | 1326\|4363\|5139\|6198 | MAP3K8\|ABCC1\|PDE3A\|RPS6KB1 | -1.5 |
|  |  | PGB:00037 | PaGenBase | Tissue-specific: heart | -2.3 | 2.7 | 3.3 | 558\|1129\|2170\|2837\|3741\|5139\|7134\|7137\|7139\|8658 | AXL\|CHRM2\|FABP3\|UTS2R\|KCNA5\|PDE3A\|TNNC1\|TNNI3\|TNNT2\|TNKS | -1.5 |
|  |  | PGB:00087 | PaGenBase | Cell-specific: Lymphoma burkitts Raji | -2.2 | 3.7 | 3.5 | 590\|1269\|3816\|7421\|9970\|10783 | BCHE\|CNR2\|KLK1\|VDR\|NR1I3\|NEK6 | -1.4 |
|  |  | PGB:00041 | PaGenBase | Tissue-specific: Blood | -2.2 | 2.9 | 3.2 | 1432\|2268\|3577\|4067\|5293\|5777\|5836\|6402 | MAPK14\|FGR\|CXCR1\|LYN\|PIK3CD\|PTPN6\|PYGL\|SELL | -1.3 |
|  |  | PGB:00101 | PaGenBase | Tissue-specific: Colorectal adenocarcinoma | -1.9 | 6.3 | 3.7 | 595\|6790\|7153 | CCND1\|AURKA\|TOP2A | -1.1 |
|  |  | PGB:00016 | PaGenBase | Tissue-specific: thymus | -1.9 | 2.9 | 2.9 | 135\|1814\|2289\|2322\|4321\|6772\|11238 | ADORA2A\|DRD3\|FKBP5\|FLT3\|MMP12\|STAT1\|CA5B | -1.1 |
|  |  | PGB:00008 | PaGenBase | Tissue-specific: small intestine | -1.8 | 3.4 | 2.9 | 1268\|2101\|4321\|29881\|57016 | CNR1\|ESRRA\|MMP12\|NPC1L1\|AKR1B10 | -0.93 |
|  |  | PGB:00081 | PaGenBase | Cell-specific: Bronchial Epithelial Cells | -1.7 | 3.1 | 2.7 | 771\|1956\|4233\|5743\|5792 | CA12\|EGFR\|MET\|PTGS2\|PTPRF | -0.82 |
|  |  | PGB:00057 | PaGenBase | Tissue-specific: ovary | -1.6 | 2.5 | 2.5 | 1588\|2099\|2100\|2561\|3290\|10846\|29881 | CYP19A1\|ESR1\|ESR2\|GABRB2\|HSD11B1\|PDE10A\|NPC1L1 | -0.76 |
|  |  | PGB:00029 | PaGenBase | Cell-specific: HL60 | -1.6 | 3.6 | 2.7 | 1326\|4363\|5771\|7376 | MAP3K8\|ABCC1\|PTPN2\|NR1H2 | -0.74 |
|  |  | PGB:00060 | PaGenBase | Tissue-specific: retinoblastoma | -1.6 | 3.6 | 2.7 | 4314\|4323\|4843\|7421 | MMP3\|MMP14\|NOS2\|VDR | -0.74 |
|  |  | PGB:00032 | PaGenBase | Tissue-specific: cortex | -1.6 | 4.6 | 2.9 | 2561\|5141\|6532 | GABRB2\|PDE4A\|SLC6A4 | -0.73 |
|  |  | PGB:00026 | PaGenBase | Cell-specific: CD71+ EarlyErythroid | -1.5 | 2.9 | 2.5 | 383\|759\|760\|6622\|9429 | ARG1\|CA1\|CA2\|SNCA\|ABCG2 | -0.71 |
|  |  | PGB:00015 | PaGenBase | Tissue-specific: Smooth Muscle | -1.5 | 3.3 | 2.6 | 558\|5054\|5742\|5743 | AXL\|SERPINE1\|PTGS1\|PTGS2 | -0.66 |
|  |  | PGB:00030 | PaGenBase | Tissue-specific: prostate | -1.4 | 2.4 | 2.3 | 2911\|3817\|4314\|5144\|5422\|5742 | GRM1\|KLK2\|MMP3\|PDE4D\|POLA1\|PTGS1 | -0.59 |
|  |  | PGB:00063 | PaGenBase | Cell-specific: OVR278S | -1.4 | 3.9 | 2.6 | 4638\|5465\|8658 | MYLK\|PPARA\|TNKS | -0.56 |
|  | VMC-related targets | PGB:00031 | PaGenBase | Cell-specific: HUVEC | -5.5 | 9 | 7.6 | 146\|595\|2321\|5054\|5228\|5340\|5599\|7299 | ADRA1D\|CCND1\|FLT1\|SERPINE1\|PGF\|PLG\|MAPK8\|TYR | -4.3 |
|  |  | PGB:00045 | PaGenBase | Tissue-specific: placenta | -4.1 | 8.3 | 6.2 | 1956\|2321\|5054\|5228\|6530\|6532 | EGFR\|FLT1\|SERPINE1\|PGF\|SLC6A2\|SLC6A4 | -2.9 |
|  |  | PGB:00067 | PaGenBase | Cell-specific: Brain cell | -3.2 | 19 | 7.1 | 4313\|5054\|7134 | MMP2\|SERPINE1\|TNNC1 | -2.2 |
|  |  | PGB:00037 | PaGenBase | Tissue-specific: heart | -3 | 6.5 | 4.9 | 1129\|2170\|7134\|7137\|7139 | CHRM2\|FABP3\|TNNC1\|TNNI3\|TNNT2 | -1.9 |
|  |  | PGB:00018 | PaGenBase | Tissue-specific: lung | -2.9 | 5 | 4.4 | 146\|3383\|4233\|4353\|6532\|81027 | ADRA1D\|ICAM1\|MET\|MPO\|SLC6A4\|TUBB1 | -1.8 |
|  |  | PGB:00001 | PaGenBase | Tissue-specific: liver | -2.9 | 4.2 | 4.2 | 146\|383\|1557\|1956\|2147\|5340\|6530 | ADRA1D\|ARG1\|CYP2C19\|EGFR\|F2\|PLG\|SLC6A2 | -1.8 |
|  |  | PGB:00060 | PaGenBase | Tissue-specific: retinoblastoma | -2.8 | 13 | 5.8 | 4314\|4843\|7421 | MMP3\|NOS2\|VDR | -1.7 |
|  |  | PGB:00002 | PaGenBase | Cell-specific: HEPG2 | -2.5 | 4.9 | 4 | 2147\|4233\|5781\|6198\|6532 | F2\|MET\|PTPN11\|RPS6KB1\|SLC6A4 | -1.4 |
|  |  | PGB:00014 | PaGenBase | Cell-specific: DRG | -2.3 | 4.6 | 3.8 | 43\|5468\|6530\|7134\|7137 | ACHE\|PPARG\|SLC6A2\|TNNC1\|TNNI3 | -1.3 |
|  |  | PGB:00011 | PaGenBase | Tissue-specific: spleen | -1.5 | 3.3 | 2.6 | 4318\|5294\|6402\|81027 | MMP9\|PIK3CG\|SELL\|TUBB1 | -0.46 |
| KS | whole targets | PGB:00001 | PaGenBase | Tissue-specific: liver | -10 | 3.3 | 8.2 | 124|126|146|148|383|590|763|949|1066|1109|1312|1557|1559|1565|1610|1645|1723|1956|2147|2152|2159|2642|2918|2946|3248|3290|3294|3690|5340|5444|6256|6462|6530|6652|6716|7498|8824|8856|9971|10454|79054 | ADH1A|ADH1C|ADRA1D|ADRA1A|ARG1|BCHE|CA5A|SCARB1|CES1|AKR1C4|COMT|CYP2C19|CYP2C9|CYP2D6|DAO|AKR1C1|DHODH|EGFR|F2|F3|F10|GCGR|GRM8|GSTM2|HPGD|HSD11B1|HSD17B2|ITGB3|PLG|PON1|RXRA|SHBG|SLC6A2|SORD|SRD5A2|XDH|CES2|NR1I2|NR1H4|TAB1|TRPM8 | -9.1 |
|  |  | PGB:00072 | PaGenBase | Tissue-specific: uterus | -7.5 | 8 | 8.7 | 1909\|2099\|2191\|2260\|3357\|3558\|4316\|4321\|5241\|5733\|10846\|56547 | EDNRA\|ESR1\|FAP\|FGFR1\|HTR2B\|IL2\|MMP7\|MMP12\|PGR\|PTGER3\|PDE10A\|MMP26 | -6.3 |
|  |  | PGB:00049 | PaGenBase | Cell-specific: Adipocyte | -6.4 | 5.4 | 7.1 | 185\|1513\|2052\|2152\|2191\|4128\|4311\|4313\|4321\|5156\|5159\|5320\|5468\|11096 | AGTR1\|CTSK\|EPHX1\|F3\|FAP\|MAOA\|MME\|MMP2\|MMP12\|PDGFRA\|PDGFRB\|PLA2G2A\|PPARG\|ADAMTS5 | -5.2 |
|  |  | PGB:00045 | PaGenBase | Tissue-specific: placenta | -6.1 | 3.7 | 6.3 | 185\|251\|412\|898\|1017\|1588\|1612\|1956\|2321\|2324\|3248\|3292\|3294\|3480\|5054\|5228\|6530\|6532\|7430\|9536 | AGTR1\|ALPG\|STS\|CCNE1\|CDK2\|CYP19A1\|DAPK1\|EGFR\|FLT1\|FLT4\|HPGD\|HSD17B1\|HSD17B2\|IGF1R\|SERPINE1\|PGF\|SLC6A2\|SLC6A4\|EZR\|PTGES | -5 |
|  |  | PGB:00014 | PaGenBase | Cell-specific: DRG | -6 | 3 | 6 | 43\|124\|126\|185\|760\|1268\|1545\|1588\|2166\|3293\|3357\|3362\|4137\|4914\|5170\|5298\|5468\|5733\|5979\|6256\|6530\|7134\|7137\|8900\|23173 | ACHE\|ADH1A\|ADH1C\|AGTR1\|CA2\|CNR1\|CYP1B1\|CYP19A1\|FAAH\|HSD17B3\|HTR2B\|HTR6\|MAPT\|NTRK1\|PDPK1\|PI4KB\|PPARG\|PTGER3\|RET\|RXRA\|SLC6A2\|TNNC1\|TNNI3\|CCNA1\|METAP1 | -4.9 |
|  |  | PGB:00002 | PaGenBase | Cell-specific: HEPG2 | -5.5 | 3 | 5.6 | 152\|763\|1803\|1845\|2147\|2152\|3172\|3291\|3294\|4233\|5140\|5320\|5770\|6197\|6198\|6532\|6869\|7276\|8856\|9734\|9971\|27163\|51564 | ADRA2C\|CA5A\|DPP4\|DUSP3\|F2\|F3\|HNF4A\|HSD11B2\|HSD17B2\|MET\|PDE3B\|PLA2G2A\|PTPN1\|RPS6KA3\|RPS6KB1\|SLC6A4\|TACR1\|TTR\|NR1I2\|HDAC9\|NR1H4\|NAAA\|HDAC7 | -4.4 |
|  |  | PGB:00008 | PaGenBase | Tissue-specific: small intestine | -5.4 | 5.2 | 6.5 | 147\|251\|1268\|2101\|2904\|2918\|4311\|4312\|4321\|8694\|11200\|57016 | ADRA1B\|ALPG\|CNR1\|ESRRA\|GRIN2B\|GRM8\|MME\|MMP1\|MMP12\|DGAT1\|CHEK2\|AKR1B10 | -4.4 |
|  |  | PGB:00048 | PaGenBase | Tissue-specific: bone marrow | -5.4 | 3.9 | 6 | 598\|890\|1991\|2099\|2529\|2870\|3674\|4317\|4353\|5144\|6850\|6916\|7124\|9212\|10013\|81027 | BCL2L1\|CCNA2\|ELANE\|ESR1\|FUT7\|GRK6\|ITGA2B\|MMP8\|MPO\|PDE4D\|SYK\|TBXAS1\|TNF\|AURKB\|HDAC6\|TUBB1 | -4.3 |
|  |  | PGB:00031 | PaGenBase | Cell-specific: HUVEC | -5.3 | 3.1 | 5.6 | 94\|146\|148\|558\|595\|1901\|2152\|2321\|3269\|3690\|3776\|4312\|4321\|5054\|5139\|5228\|5340\|6051\|6582\|6869\|7299 | ACVRL1\|ADRA1D\|ADRA1A\|AXL\|CCND1\|S1PR1\|F3\|FLT1\|HRH1\|ITGB3\|KCNK2\|MMP1\|MMP12\|SERPINE1\|PDE3A\|PGF\|PLG\|RNPEP\|SLC22A2\|TACR1\|TYR | -4.3 |
|  |  | PGB:00011 | PaGenBase | Tissue-specific: spleen | -5.3 | 2.8 | 5.4 | 251\|1269\|1890\|2208\|2529\|3055\|3359\|3577\|3674\|3683\|3702\|4312\|4318\|4321\|4325\|5293\|5294\|5731\|6916\|7294\|8973\|9020\|11040\|54106\|81027 | ALPG\|CNR2\|TYMP\|FCER2\|FUT7\|HCK\|HTR3A\|CXCR1\|ITGA2B\|ITGAL\|ITK\|MMP1\|MMP9\|MMP12\|MMP16\|PIK3CD\|PIK3CG\|PTGER1\|TBXAS1\|TXK\|CHRNA6\|MAP3K14\|PIM2\|TLR9\|TUBB1 | -4.2 |
|  |  | PGB:00016 | PaGenBase | Tissue-specific: thymus | -4.5 | 3.7 | 5.3 | 135\|156\|1136\|1814\|2322\|3363\|3702\|3932\|4012\|4321\|6772\|7294\|9612\|11238 | ADORA2A\|GRK2\|CHRNA3\|DRD3\|FLT3\|HTR7\|ITK\|LCK\|LNPEP\|MMP12\|STAT1\|TXK\|NCOR2\|CA5B | -3.5 |
|  |  | PGB:00018 | PaGenBase | Tissue-specific: lung | -4.3 | 2.6 | 4.8 | 146\|151\|246\|247\|762\|765\|1215\|1859\|1991\|3269\|3355\|3815\|4233\|4317\|4322\|4353\|5319\|5743\|6098\|6532\|8856\|81027\|89780 | ADRA1D\|ADRA2B\|ALOX15\|ALOX15B\|CA4\|CA6\|CMA1\|DYRK1A\|ELANE\|HRH1\|HTR1F\|KIT\|MET\|MMP8\|MMP13\|MPO\|PLA2G1B\|PTGS2\|ROS1\|SLC6A4\|NR1I2\|TUBB1\|WNT3A | -3.3 |
|  |  | PGB:00095 | PaGenBase | Cell-specific: LNCAP | -4.3 | 9 | 6.6 | 150\|231\|367\|3291\|3817\|4128 | ADRA2A\|AKR1B1\|AR\|HSD11B2\|KLK2\|MAOA | -3.3 |
|  |  | PGB:00004 | PaGenBase | Tissue-specific: kidney | -4.1 | 2.5 | 4.6 | 152\|496\|596\|760\|771\|983\|1142\|1803\|1815\|2103\|3291\|3758\|4282\|4316\|5465\|5733\|5972\|6524\|6582\|50507\|55244\|116085 | ADRA2C\|ATP4B\|BCL2\|CA2\|CA12\|CDK1\|CHRNB3\|DPP4\|DRD4\|ESRRB\|HSD11B2\|KCNJ1\|MIF\|MMP7\|PPARA\|PTGER3\|REN\|SLC5A2\|SLC22A2\|NOX4\|SLC47A1\|SLC22A12 | -3.1 |
|  |  | PGB:00079 | PaGenBase | Tissue-specific: Caudate nucleus | -4.1 | 8.1 | 6.2 | 135\|1812\|2100\|2915\|3358\|10846 | ADORA2A\|DRD1\|ESR2\|GRM5\|HTR2C\|PDE10A | -3.1 |
|  |  | PGB:00063 | PaGenBase | Cell-specific: OVR278S | -3.7 | 5.9 | 5.4 | 2260\|3055\|4638\|5322\|5465\|5753\|8658 | FGFR1\|HCK\|MYLK\|PLA2G5\|PPARA\|PTK6\|TNKS | -2.8 |
|  |  | PGB:00067 | PaGenBase | Cell-specific: Brain cell | -3.7 | 5.8 | 5.3 | 4313\|5054\|5156\|5733\|5743\|7134\|9510 | MMP2\|SERPINE1\|PDGFRA\|PTGER3\|PTGS2\|TNNC1\|ADAMTS1 | -2.8 |
|  |  | PGB:00037 | PaGenBase | Tissue-specific: heart | -3.6 | 2.8 | 4.3 | 558\|1129\|1138\|1903\|2837\|3741\|4012\|5139\|5322\|6331\|7134\|7137\|7139\|8658\|9261\|51086 | AXL\|CHRM2\|CHRNA5\|S1PR3\|UTS2R\|KCNA5\|LNPEP\|PDE3A\|PLA2G5\|SCN5A\|TNNC1\|TNNI3\|TNNT2\|TNKS\|MAPKAPK2\|TNNI3K | -2.6 |
|  |  | PGB:00060 | PaGenBase | Tissue-specific: retinoblastoma | -3.4 | 4.6 | 4.8 | 1513\|2191\|4312\|4314\|4323\|4843\|5111\|7298 | CTSK\|FAP\|MMP1\|MMP3\|MMP14\|NOS2\|PCNA\|TYMS | -2.5 |
|  |  | PGB:00015 | PaGenBase | Tissue-specific: Smooth Muscle | -3.2 | 4.3 | 4.6 | 136\|558\|1803\|2191\|4312\|5054\|5742\|5743 | ADORA2B\|AXL\|DPP4\|FAP\|MMP1\|SERPINE1\|PTGS1\|PTGS2 | -2.4 |
|  |  | PGB:00057 | PaGenBase | Tissue-specific: ovary | -3.2 | 2.9 | 4.1 | 155\|496\|1137\|1588\|2099\|2100\|2864\|2946\|3290\|5241\|5972\|8972\|10846 | ADRB3\|ATP4B\|CHRNA4\|CYP19A1\|ESR1\|ESR2\|FFAR1\|GSTM2\|HSD11B1\|PGR\|REN\|MGAM\|PDE10A | -2.3 |
|  |  | PGB:00137 | PaGenBase | Cell-specific: LN18 | -3.1 | 15 | 6.3 | 1513\|3294\|6916 | CTSK\|HSD17B2\|TBXAS1 | -2.2 |
|  |  | PGB:00039 | PaGenBase | Cell-specific: THY- | -2.6 | 5.2 | 4.2 | 1991\|3248\|3363\|3674\|27306 | ELANE\|HPGD\|HTR7\|ITGA2B\|HPGDS | -1.8 |
|  |  | PGB:00028 | PaGenBase | Tissue-specific: prostate cancer | -2.5 | 5 | 4.1 | 1326\|4363\|5139\|6198\|8654 | MAP3K8\|ABCC1\|PDE3A\|RPS6KB1\|PDE5A | -1.7 |
|  |  | PGB:00022 | PaGenBase | Tissue-specific: adrenal gland | -2.4 | 2.8 | 3.4 | 495\|706\|949\|2208\|3776\|5156\|5243\|5409\|7357\|7442 | ATP4A\|TSPO\|SCARB1\|FCER2\|KCNK2\|PDGFRA\|ABCB1\|PNMT\|UGCG\|TRPV1 | -1.6 |
|  |  | PGB:00121 | PaGenBase | Cell-specific: CD34+ | -2.4 | 6.2 | 4.2 | 1991\|3674\|3815\|4353 | ELANE\|ITGA2B\|KIT\|MPO | -1.6 |
|  |  | PGB:00068 | PaGenBase | Tissue-specific: Stomach | -2 | 4.6 | 3.4 | 495\|496\|768\|4282 | ATP4A\|ATP4B\|CA9\|MIF | -1.2 |
|  |  | PGB:00062 | PaGenBase | Cell-specific: Skeletal Muscle Stromal Cells | -2 | 6.4 | 3.7 | 3643\|7134\|7137 | INSR\|TNNC1\|TNNI3 | -1.2 |
|  |  | PGB:00087 | PaGenBase | Cell-specific: Lymphoma burkitts Raji | -1.9 | 2.8 | 2.8 | 590\|1269\|2033\|2208\|3816\|4282\|10783 | BCHE\|CNR2\|EP300\|FCER2\|KLK1\|MIF\|NEK6 | -1.1 |
|  |  | PGB:00047 | PaGenBase | Cell-specific: THY+ | -1.8 | 3 | 2.9 | 239\|816\|873\|2099\|6714\|9149 | ALOX12\|CAMK2B\|CBR1\|ESR1\|SRC\|DYRK1B | -1.1 |
|  |  | PGB:00026 | PaGenBase | Cell-specific: CD71+ EarlyErythroid | -1.8 | 2.6 | 2.7 | 383\|598\|759\|760\|4860\|6622\|9429 | ARG1\|BCL2L1\|CA1\|CA2\|PNP\|SNCA\|ABCG2 | -1 |
|  |  | PGB:00073 | PaGenBase | Tissue-specific: salivary gland | -1.8 | 2.6 | 2.7 | 765\|3351\|5578\|5802\|9149\|10381\|27035 | CA6\|HTR1B\|PRKCA\|PTPRS\|DYRK1B\|TUBB3\|NOX1 | -1 |
|  |  | PGB:00065 | PaGenBase | Tissue-specific: Breast | -1.7 | 2.6 | 2.6 | 231\|367\|1646\|2064\|3356\|4233\|116085 | AKR1B1\|AR\|AKR1C2\|ERBB2\|HTR2A\|MET\|SLC22A12 | -0.96 |
|  |  | PGB:00069 | PaGenBase | Cell-specific: A204 | -1.7 | 3.9 | 3 | 887\|2260\|4325\|7134 | CCKBR\|FGFR1\|MMP16\|TNNC1 | -0.96 |
|  |  | PGB:00078 | PaGenBase | Cell-specific: liver cell | -1.6 | 3.1 | 2.7 | 1646\|2147\|2152\|7276\|8644 | AKR1C2\|F2\|F3\|TTR\|AKR1C3 | -0.9 |
|  |  | PGB:00056 | PaGenBase | Tissue-specific: spinal cord | -1.5 | 3.5 | 2.7 | 1128\|2152\|2342\|3558 | CHRM1\|F3\|FNTB\|IL2 | -0.8 |
|  |  | PGB:00084 | PaGenBase | Tissue-specific: Lymph node | -1.5 | 2.9 | 2.5 | 1018\|5594\|6916\|9252\|11200 | CDK3\|MAPK1\|TBXAS1\|RPS6KA5\|CHEK2 | -0.76 |
|  |  | PGB:00025 | PaGenBase | Tissue-specific: pancreas | -1.5 | 2 | 2.2 | 328\|598\|706\|887\|1080\|1144\|3292\|4193\|5319 | APEX1\|BCL2L1\|TSPO\|CCKBR\|CFTR\|CHRND\|HSD17B1\|MDM2\|PLA2G1B | -0.72 |
|  |  | PGB:00101 | PaGenBase | Tissue-specific: Colorectal adenocarcinoma | -1.4 | 4.1 | 2.7 | 595\|6790\|7153 | CCND1\|AURKA\|TOP2A | -0.69 |
|  |  | PGB:00036 | PaGenBase | Cell-specific: MOLT4 | -1.4 | 2.4 | 2.3 | 146\|1080\|3702\|3932\|4312\|5578 | ADRA1D\|CFTR\|ITK\|LCK\|MMP1\|PRKCA | -0.68 |
|  |  | PGB:00081 | PaGenBase | Cell-specific: Bronchial Epithelial Cells | -1.4 | 2.4 | 2.3 | 771\|1956\|2152\|4233\|5743\|9510 | CA12\|EGFR\|F3\|MET\|PTGS2\|ADAMTS1 | -0.68 |
|  |  | PGB:00030 | PaGenBase | Tissue-specific: prostate | -1.4 | 2.1 | 2.1 | 247\|2911\|3817\|4314\|5144\|5742\|59340\|79054 | ALOX15B\|GRM1\|KLK2\|MMP3\|PDE4D\|PTGS1\|HRH4\|TRPM8 | -0.66 |
|  |  | PGB:00007 | PaGenBase | Tissue-specific: colon | -1.4 | 1.8 | 2 | 759\|760\|766\|1080\|2859\|5595\|5753\|5979\|7015\|8399\|27035 | CA1\|CA2\|CA7\|CFTR\|GPR35\|MAPK3\|PTK6\|RET\|TERT\|PLA2G10\|NOX1 | -0.62 |
|  | VMC-related targets | PGB:00031 | PaGenBase | Cell-specific: HUVEC | -10 | 11 | 11 | 94\|146\|595\|1901\|2152\|2321\|3269\|3690\|4312\|5054\|5228\|5340\|6869\|7299 | ACVRL1\|ADRA1D\|CCND1\|S1PR1\|F3\|FLT1\|HRH1\|ITGB3\|MMP1\|SERPINE1\|PGF\|PLG\|TACR1\|TYR | -8.7 |
|  |  | PGB:00121 | PaGenBase | Cell-specific: CD34+ | -5.1 | 31 | 11 | 1991\|3674\|3815\|4353 | ELANE\|ITGA2B\|KIT\|MPO | -3.9 |
|  |  | PGB:00018 | PaGenBase | Tissue-specific: lung | -4.9 | 5.6 | 6.2 | 146\|1215\|1991\|3269\|3815\|4233\|4317\|4353\|6532\|81027 | ADRA1D\|CMA1\|ELANE\|HRH1\|KIT\|MET\|MMP8\|MPO\|SLC6A4\|TUBB1 | -3.8 |
|  |  | PGB:00045 | PaGenBase | Tissue-specific: placenta | -4 | 6.5 | 5.7 | 185\|1956\|2321\|5054\|5228\|6530\|6532 | AGTR1\|EGFR\|FLT1\|SERPINE1\|PGF\|SLC6A2\|SLC6A4 | -2.9 |
|  |  | PGB:00036 | PaGenBase | Cell-specific: MOLT4 | -3.9 | 10 | 6.5 | 146\|3702\|3932\|4312\|5578 | ADRA1D\|ITK\|LCK\|MMP1\|PRKCA | -2.8 |
|  |  | PGB:00048 | PaGenBase | Tissue-specific: bone marrow | -3.8 | 7.4 | 5.8 | 1991\|3674\|4317\|4353\|7124\|81027 | ELANE\|ITGA2B\|MMP8\|MPO\|TNF\|TUBB1 | -2.7 |
|  |  | PGB:00014 | PaGenBase | Cell-specific: DRG | -3.6 | 4.9 | 5.1 | 43\|185\|4914\|5468\|5979\|6530\|7134\|7137 | ACHE\|AGTR1\|NTRK1\|PPARG\|RET\|SLC6A2\|TNNC1\|TNNI3 | -2.6 |
|  |  | PGB:00060 | PaGenBase | Tissue-specific: retinoblastoma | -3.4 | 12 | 6.2 | 4312\|4314\|4843\|5111 | MMP1\|MMP3\|NOS2\|PCNA | -2.4 |
|  |  | PGB:00011 | PaGenBase | Tissue-specific: spleen | -3.4 | 4.5 | 4.7 | 3674\|3683\|3702\|4312\|4318\|5294\|54106\|81027 | ITGA2B\|ITGAL\|ITK\|MMP1\|MMP9\|PIK3CG\|TLR9\|TUBB1 | -2.4 |
|  |  | PGB:00001 | PaGenBase | Tissue-specific: liver | -3.1 | 3.6 | 4.2 | 146\|383\|1557\|1956\|2147\|2152\|3690\|5340\|6530 | ADRA1D\|ARG1\|CYP2C19\|EGFR\|F2\|F3\|ITGB3\|PLG\|SLC6A2 | -2.1 |
|  |  | PGB:00037 | PaGenBase | Tissue-specific: heart | -3 | 5.2 | 4.6 | 1129\|1138\|6331\|7134\|7137\|7139 | CHRM2\|CHRNA5\|SCN5A\|TNNC1\|TNNI3\|TNNT2 | -2 |
|  |  | PGB:00067 | PaGenBase | Cell-specific: Brain cell | -2.7 | 13 | 5.7 | 4313\|5054\|7134 | MMP2\|SERPINE1\|TNNC1 | -1.8 |
|  |  | PGB:00049 | PaGenBase | Cell-specific: Adipocyte | -2.7 | 7.7 | 4.9 | 185\|2152\|4313\|5468 | AGTR1\|F3\|MMP2\|PPARG | -1.7 |
|  |  | PGB:00002 | PaGenBase | Cell-specific: HEPG2 | -2.4 | 4 | 3.7 | 2147\|2152\|4233\|6198\|6532\|6869 | F2\|F3\|MET\|RPS6KB1\|SLC6A4\|TACR1 | -1.4 |
|  |  | PGB:00016 | PaGenBase | Tissue-specific: thymus | -2.2 | 5.3 | 3.8 | 135\|3702\|3932\|6772 | ADORA2A\|ITK\|LCK\|STAT1 | -1.2 |
|  |  | PGB:00027 | PaGenBase | Cell-specific: K562 | -2 | 3.3 | 3.1 | 25\|1138\|3757\|7124\|7137\|7422 | ABL1\|CHRNA5\|KCNH2\|TNF\|TNNI3\|VEGFA | -1 |
|  |  | PGB:00081 | PaGenBase | Cell-specific: Bronchial Epithelial Cells | -1.9 | 6.1 | 3.6 | 1956\|2152\|4233 | EGFR\|F3\|MET | -0.91 |
|  |  | PGB:00010 | PaGenBase | Tissue-specific: adipose tissue | -1.6 | 4.8 | 3 | 383\|1215\|4353 | ARG1\|CMA1\|MPO | -0.65 |
|  |  | PGB:00022 | PaGenBase | Tissue-specific: adrenal gland | -1.4 | 4.2 | 2.7 | 706\|5243\|5409 | TSPO\|ABCB1\|PNMT | -0.49 |
| JH | whole targets | PGB:00001 | PaGenBase | Tissue-specific: liver | -5.8 | 7.9 | 7.5 | 590|866|1557|3290|6462|6530|8824|9970|29881 | BCHE|SERPINA6|CYP2C19|HSD11B1|SHBG|SLC6A2|CES2|NR1I3|NPC1L1 | -3.7 |
|  |  | PGB:00057 | PaGenBase | Tissue-specific: ovary | -4.3 | 12 | 7.3 | 1588\|2099\|2100\|3290\|29881 | CYP19A1\|ESR1\|ESR2\|HSD11B1\|NPC1L1 | -2.5 |
|  |  | PGB:00014 | PaGenBase | Cell-specific: DRG | -3.1 | 6.7 | 5 | 43\|1588\|5468\|6530\|10062 | ACHE\|CYP19A1\|PPARG\|SLC6A2\|NR1H3 | -1.4 |
|  |  | PGB:00045 | PaGenBase | Tissue-specific: placenta | -2.8 | 8 | 5 | 1588\|6530\|6532\|9536 | CYP19A1\|SLC6A2\|SLC6A4\|PTGES | -1.2 |
|  |  | PGB:00087 | PaGenBase | Cell-specific: Lymphoma burkitts Raji | -2.8 | 13 | 5.8 | 590\|7421\|9970 | BCHE\|VDR\|NR1I3 | -1.2 |
|  |  | PGB:00002 | PaGenBase | Cell-specific: HEPG2 | -2.3 | 5.7 | 4 | 866\|5770\|6532\|7364 | SERPINA6\|PTPN1\|SLC6A4\|UGT2B7 | -0.75 |
|  |  | PGB:00011 | PaGenBase | Tissue-specific: spleen | -2 | 4.9 | 3.5 | 5731\|5777\|6916\|10062 | PTGER1\|PTPN6\|TBXAS1\|NR1H3 | -0.53 |
|  | VMC-related targets | PGB:00014 | PaGenBase | Cell-specific: DRG | -3.1 | 15 | 6.4 | 43\|5468\|6530 | ACHE\|PPARG\|SLC6A2 | -0.97 |
| CH | whole targets | PGB:00049 | PaGenBase | Cell-specific: Adipocyte | -7.8 | 7.7 | 8.8 | 32|1513|2052|2167|2191|4128|4311|4313|4321|5320|5468|5950|10062 | ACACB|CTSK|EPHX1|FABP4|FAP|MAOA|MME|MMP2|MMP12|PLA2G2A|PPARG|RBP4|NR1H3 | -6.4 |
|  |  | PGB:00001 | PaGenBase | Tissue-specific: liver | -6.7 | 3.2 | 6.4 | 383\|590\|763\|866\|1109\|1557\|1584\|1585\|1645\|1956\|2147\|2159\|2203\|2645\|3290\|3294\|3690\|5340\|5950\|6462\|6480\|6530\|7498\|8564\|9970\|29881 | ARG1\|BCHE\|CA5A\|SERPINA6\|AKR1C4\|CYP2C19\|CYP11B1\|CYP11B2\|AKR1C1\|EGFR\|F2\|F10\|FBP1\|GCK\|HSD11B1\|HSD17B2\|ITGB3\|PLG\|RBP4\|SHBG\|ST6GAL1\|SLC6A2\|XDH\|KMO\|NR1I3\|NPC1L1 | -5.5 |
|  |  | PGB:00008 | PaGenBase | Tissue-specific: small intestine | -6.5 | 7.4 | 7.9 | 1268\|2101\|2904\|4311\|4312\|4321\|6523\|8694\|11200\|29881\|57016 | CNR1\|ESRRA\|GRIN2B\|MME\|MMP1\|MMP12\|SLC5A1\|DGAT1\|CHEK2\|NPC1L1\|AKR1B10 | -5.2 |
|  |  | PGB:00014 | PaGenBase | Cell-specific: DRG | -5.7 | 3.6 | 6 | 43\|102\|760\|1268\|1545\|1588\|1675\|2166\|2167\|2203\|3062\|3362\|4137\|4914\|5298\|5468\|6530\|8900\|10062 | ACHE\|ADAM10\|CA2\|CNR1\|CYP1B1\|CYP19A1\|CFD\|FAAH\|FABP4\|FBP1\|HCRTR2\|HTR6\|MAPT\|NTRK1\|PI4KB\|PPARG\|SLC6A2\|CCNA1\|NR1H3 | -4.5 |
|  |  | PGB:00095 | PaGenBase | Cell-specific: LNCAP | -5.4 | 14 | 8.5 | 150\|231\|367\|3291\|3760\|4128 | ADRA2A\|AKR1B1\|AR\|HSD11B2\|KCNJ3\|MAOA | -4.2 |
|  |  | PGB:00137 | PaGenBase | Cell-specific: LN18 | -5.3 | 31 | 11 | 834\|1513\|3294\|6916 | CASP1\|CTSK\|HSD17B2\|TBXAS1 | -4.1 |
|  |  | PGB:00060 | PaGenBase | Tissue-specific: retinoblastoma | -4.7 | 7.1 | 6.5 | 623\|1513\|2191\|4312\|4314\|4323\|4843\|7421 | BDKRB1\|CTSK\|FAP\|MMP1\|MMP3\|MMP14\|NOS2\|VDR | -3.6 |
|  |  | PGB:00022 | PaGenBase | Tissue-specific: adrenal gland | -4.6 | 4.7 | 5.7 | 706\|1584\|1585\|1586\|2208\|3762\|3777\|5243\|6095\|7442\|64840 | TSPO\|CYP11B1\|CYP11B2\|CYP17A1\|FCER2\|KCNJ5\|KCNK3\|ABCB1\|RORA\|TRPV1\|PORCN | -3.5 |
|  |  | PGB:00072 | PaGenBase | Tissue-specific: uterus | -4.3 | 7.2 | 6.2 | 2099\|2191\|2260\|3558\|4316\|4321\|10846 | ESR1\|FAP\|FGFR1\|IL2\|MMP7\|MMP12\|PDE10A | -3.2 |
|  |  | PGB:00045 | PaGenBase | Tissue-specific: placenta | -4.2 | 3.7 | 5.1 | 898\|1017\|1588\|1612\|1956\|2162\|3292\|3294\|3480\|6530\|6532\|7052\|9536 | CCNE1\|CDK2\|CYP19A1\|DAPK1\|EGFR\|F13A1\|HSD17B1\|HSD17B2\|IGF1R\|SLC6A2\|SLC6A4\|TGM2\|PTGES | -3.2 |
|  |  | PGB:00078 | PaGenBase | Cell-specific: liver cell | -4.1 | 6.8 | 5.9 | 1646\|2147\|2167\|5950\|7276\|7364\|8644 | AKR1C2\|F2\|FABP4\|RBP4\|TTR\|UGT2B7\|AKR1C3 | -3 |
|  |  | PGB:00002 | PaGenBase | Cell-specific: HEPG2 | -3.8 | 3 | 4.6 | 152\|763\|866\|1585\|2147\|3291\|3294\|4233\|5320\|5770\|5950\|6197\|6532\|7276\|7364 | ADRA2C\|CA5A\|SERPINA6\|CYP11B2\|F2\|HSD11B2\|HSD17B2\|MET\|PLA2G2A\|PTPN1\|RBP4\|RPS6KA3\|SLC6A4\|TTR\|UGT2B7 | -2.8 |
|  |  | PGB:00016 | PaGenBase | Tissue-specific: thymus | -3.7 | 4.1 | 4.9 | 100\|135\|839\|1515\|2322\|3363\|3702\|3932\|4321\|7294 | ADA\|ADORA2A\|CASP6\|CTSV\|FLT3\|HTR7\|ITK\|LCK\|MMP12\|TXK | -2.7 |
|  |  | PGB:00011 | PaGenBase | Tissue-specific: spleen | -3.6 | 2.8 | 4.3 | 1269\|2149\|2208\|3055\|3577\|3702\|4312\|4318\|4321\|5293\|5294\|5731\|5777\|6916\|7294\|10062 | CNR2\|F2R\|FCER2\|HCK\|CXCR1\|ITK\|MMP1\|MMP9\|MMP12\|PIK3CD\|PIK3CG\|PTGER1\|PTPN6\|TBXAS1\|TXK\|NR1H3 | -2.6 |
|  |  | PGB:00048 | PaGenBase | Tissue-specific: bone marrow | -3.5 | 3.8 | 4.6 | 890\|1991\|2099\|2870\|4353\|5144\|6850\|6916\|7124\|9212 | CCNA2\|ELANE\|ESR1\|GRK6\|MPO\|PDE4D\|SYK\|TBXAS1\|TNF\|AURKB | -2.5 |
|  |  | PGB:00192 | PaGenBase | Cell-specific: GM133 | -3.4 | 20 | 7.4 | 4311\|4321\|5467 | MME\|MMP12\|PPARD | -2.4 |
|  |  | PGB:00063 | PaGenBase | Cell-specific: OVR278S | -3 | 6.5 | 4.9 | 2260\|3055\|4638\|5465\|8658 | FGFR1\|HCK\|MYLK\|PPARA\|TNKS | -2.1 |
|  |  | PGB:00079 | PaGenBase | Tissue-specific: Caudate nucleus | -2.9 | 8.4 | 5.1 | 135\|2100\|2915\|10846 | ADORA2A\|ESR2\|GRM5\|PDE10A | -2 |
|  |  | PGB:00031 | PaGenBase | Cell-specific: HUVEC | -2.8 | 2.8 | 3.7 | 558\|595\|840\|2149\|3690\|4312\|4321\|5340\|5599\|6095\|7052\|7299 | AXL\|CCND1\|CASP7\|F2R\|ITGB3\|MMP1\|MMP12\|PLG\|MAPK8\|RORA\|TGM2\|TYR | -1.9 |
|  |  | PGB:00026 | PaGenBase | Cell-specific: CD71+ EarlyErythroid | -2.8 | 4.1 | 4.1 | 383\|759\|760\|4860\|6622\|7052\|9429 | ARG1\|CA1\|CA2\|PNP\|SNCA\|TGM2\|ABCG2 | -1.9 |
|  |  | PGB:00004 | PaGenBase | Tissue-specific: kidney | -2.3 | 2.3 | 3.2 | 152\|760\|771\|983\|1815\|2150\|3291\|4316\|5465\|5599\|7364\|50507\|116085 | ADRA2C\|CA2\|CA12\|CDK1\|DRD4\|F2RL1\|HSD11B2\|MMP7\|PPARA\|MAPK8\|UGT2B7\|NOX4\|SLC22A12 | -1.5 |
|  |  | PGB:00018 | PaGenBase | Tissue-specific: lung | -2.2 | 2.2 | 3 | 246\|762\|765\|1215\|1991\|3815\|4233\|4322\|4353\|5319\|5743\|5771\|6532 | ALOX15\|CA4\|CA6\|CMA1\|ELANE\|KIT\|MET\|MMP13\|MPO\|PLA2G1B\|PTGS2\|PTPN2\|SLC6A4 | -1.3 |
|  |  | PGB:00087 | PaGenBase | Cell-specific: Lymphoma burkitts Raji | -2.2 | 3.7 | 3.5 | 590\|1269\|2208\|7421\|9970\|10783 | BCHE\|CNR2\|FCER2\|VDR\|NR1I3\|NEK6 | -1.3 |
|  |  | PGB:00041 | PaGenBase | Tissue-specific: Blood | -2.2 | 2.9 | 3.2 | 834\|1520\|3055\|3577\|5293\|5777\|5836\|10135 | CASP1\|CTSS\|HCK\|CXCR1\|PIK3CD\|PTPN6\|PYGL\|NAMPT | -1.3 |
|  |  | PGB:00121 | PaGenBase | Cell-specific: CD34+ | -2.1 | 7.2 | 4 | 1991\|3815\|4353 | ELANE\|KIT\|MPO | -1.2 |
|  |  | PGB:00065 | PaGenBase | Tissue-specific: Breast | -2.1 | 3.4 | 3.2 | 231\|367\|1646\|3356\|4233\|116085 | AKR1B1\|AR\|AKR1C2\|HTR2A\|MET\|SLC22A12 | -1.2 |
|  |  | PGB:00036 | PaGenBase | Cell-specific: MOLT4 | -1.7 | 3.1 | 2.7 | 100\|1080\|3702\|3932\|4312 | ADA\|CFTR\|ITK\|LCK\|MMP1 | -0.82 |
|  |  | PGB:00015 | PaGenBase | Tissue-specific: Smooth Muscle | -1.5 | 3.3 | 2.6 | 558\|2191\|4312\|5743 | AXL\|FAP\|MMP1\|PTGS2 | -0.66 |
|  |  | PGB:00120 | PaGenBase | Cell-specific: Cardiac Myocytes | -1.5 | 4.3 | 2.8 | 558\|2149\|4312 | AXL\|F2R\|MMP1 | -0.66 |
|  |  | PGB:00066 | PaGenBase | Cell-specific: T-lymphoblast | -1.5 | 4.2 | 2.7 | 100\|1021\|29881 | ADA\|CDK6\|NPC1L1 | -0.64 |
|  |  | PGB:00056 | PaGenBase | Tissue-specific: spinal cord | -1.4 | 4 | 2.6 | 1128\|2342\|3558 | CHRM1\|FNTB\|IL2 | -0.59 |
|  |  | PGB:00047 | PaGenBase | Cell-specific: THY+ | -1.4 | 3.1 | 2.4 | 239\|816\|2099\|6714 | ALOX12\|CAMK2B\|ESR1\|SRC | -0.57 |
|  | VMC-related targets | PGB:00031 | PaGenBase | Cell-specific: HUVEC | -4.7 | 8.3 | 6.7 | 595\|840\|3690\|4312\|5340\|5599\|7299 | CCND1\|CASP7\|ITGB3\|MMP1\|PLG\|MAPK8\|TYR | -3.4 |
|  |  | PGB:00060 | PaGenBase | Tissue-specific: retinoblastoma | -4.1 | 18 | 8 | 4312\|4314\|4843\|7421 | MMP1\|MMP3\|NOS2\|VDR | -3 |
|  |  | PGB:00121 | PaGenBase | Cell-specific: CD34+ | -4.1 | 36 | 10 | 1991\|3815\|4353 | ELANE\|KIT\|MPO | -2.9 |
|  |  | PGB:00036 | PaGenBase | Cell-specific: MOLT4 | -3.6 | 13 | 6.6 | 100\|3702\|3932\|4312 | ADA\|ITK\|LCK\|MMP1 | -2.4 |
|  |  | PGB:00018 | PaGenBase | Tissue-specific: lung | -3 | 5.3 | 4.6 | 1215\|1991\|3815\|4233\|4353\|6532 | CMA1\|ELANE\|KIT\|MET\|MPO\|SLC6A4 | -1.9 |
|  |  | PGB:00001 | PaGenBase | Tissue-specific: liver | -3 | 4.4 | 4.4 | 383\|1557\|1956\|2147\|3690\|5340\|6530 | ARG1\|CYP2C19\|EGFR\|F2\|ITGB3\|PLG\|SLC6A2 | -1.9 |
|  |  | PGB:00016 | PaGenBase | Tissue-specific: thymus | -2.9 | 8.3 | 5.1 | 100\|135\|3702\|3932 | ADA\|ADORA2A\|ITK\|LCK | -1.8 |
|  |  | PGB:00010 | PaGenBase | Tissue-specific: adipose tissue | -2.1 | 7.5 | 4.1 | 383\|1215\|4353 | ARG1\|CMA1\|MPO | -1.1 |
|  |  | PGB:00022 | PaGenBase | Tissue-specific: adrenal gland | -2 | 6.5 | 3.8 | 706\|3762\|5243 | TSPO\|KCNJ5\|ABCB1 | -0.89 |
|  |  | PGB:00048 | PaGenBase | Tissue-specific: bone marrow | -1.8 | 5.8 | 3.5 | 1991\|4353\|7124 | ELANE\|MPO\|TNF | -0.75 |
|  |  | PGB:00014 | PaGenBase | Cell-specific: DRG | -1.7 | 3.8 | 2.9 | 43\|4914\|5468\|6530 | ACHE\|NTRK1\|PPARG\|SLC6A2 | -0.63 |
|  |  | PGB:00011 | PaGenBase | Tissue-specific: spleen | -1.6 | 3.5 | 2.7 | 3702\|4312\|4318\|5294 | ITK\|MMP1\|MMP9\|PIK3CG | -0.5 |
|  |  | PGB:00045 | PaGenBase | Tissue-specific: placenta | -1.5 | 4.3 | 2.8 | 1956\|6530\|6532 | EGFR\|SLC6A2\|SLC6A4 | -0.43 |
| JXT | whole targets | PGB:00002 | PaGenBase | Cell-specific: HEPG2 | -8.4 | 3.8 | 7.6 | 152|763|866|993|1585|1591|1845|2041|2147|2152|2168|3291|3294|4233|5143|5320|5770|5781|6198|6532|7276|7364|8856|9734|9971|27163|51564 | ADRA2C|CA5A|SERPINA6|CDC25A|CYP11B2|CYP24A1|DUSP3|EPHA1|F2|F3|FABP1|HSD11B2|HSD17B2|MET|PDE4C|PLA2G2A|PTPN1|PTPN11|RPS6KB1|SLC6A4|TTR|UGT2B7|NR1I2|HDAC9|NR1H4|NAAA|HDAC7 | -7.1 |
|  |  | PGB:00001 | PaGenBase | Tissue-specific: liver | -7.6 | 2.9 | 6.7 | 383\|590\|763\|866\|1066\|1109\|1312\|1557\|1565\|1584\|1585\|1645\|1723\|1956\|2041\|2147\|2152\|2159\|2168\|2946\|3290\|3294\|5340\|5444\|6462\|6530\|6716\|7498\|8824\|8856\|9970\|9971\|29881\|79054 | ARG1\|BCHE\|CA5A\|SERPINA6\|CES1\|AKR1C4\|COMT\|CYP2C19\|CYP2D6\|CYP11B1\|CYP11B2\|AKR1C1\|DHODH\|EGFR\|EPHA1\|F2\|F3\|F10\|FABP1\|GSTM2\|HSD11B1\|HSD17B2\|PLG\|PON1\|SHBG\|SLC6A2\|SRD5A2\|XDH\|CES2\|NR1I2\|NR1I3\|NR1H4\|NPC1L1\|TRPM8 | -6.3 |
|  |  | PGB:00011 | PaGenBase | Tissue-specific: spleen | -6.4 | 3.1 | 6.2 | 640\|695\|1269\|3359\|3577\|3676\|3683\|3702\|4067\|4312\|4318\|4321\|4325\|4846\|5293\|5294\|5551\|5731\|5777\|6916\|7099\|7294\|10062\|11040\|54106\|219972 | BLK\|BTK\|CNR2\|HTR3A\|CXCR1\|ITGA4\|ITGAL\|ITK\|LYN\|MMP1\|MMP9\|MMP12\|MMP16\|NOS3\|PIK3CD\|PIK3CG\|PRF1\|PTGER1\|PTPN6\|TBXAS1\|TLR4\|TXK\|NR1H3\|PIM2\|TLR9\|MPEG1 | -5.3 |
|  |  | PGB:00049 | PaGenBase | Cell-specific: Adipocyte | -6 | 5.4 | 6.9 | 1513\|2152\|2167\|2194\|2752\|4128\|4313\|4321\|5156\|5159\|5320\|5468\|10062 | CTSK\|F3\|FABP4\|FASN\|GLUL\|MAOA\|MMP2\|MMP12\|PDGFRA\|PDGFRB\|PLA2G2A\|PPARG\|NR1H3 | -4.9 |
|  |  | PGB:00014 | PaGenBase | Cell-specific: DRG | -5.5 | 3 | 5.7 | 43\|102\|760\|1268\|1545\|1588\|1675\|2166\|2167\|2555\|3062\|3293\|3357\|3362\|5152\|5170\|5468\|5733\|5979\|6530\|7941\|8900\|10062 | ACHE\|ADAM10\|CA2\|CNR1\|CYP1B1\|CYP19A1\|CFD\|FAAH\|FABP4\|GABRA2\|HCRTR2\|HSD17B3\|HTR2B\|HTR6\|PDE9A\|PDPK1\|PPARG\|PTGER3\|RET\|SLC6A2\|PLA2G7\|CCNA1\|NR1H3 | -4.4 |
|  |  | PGB:00048 | PaGenBase | Tissue-specific: bone marrow | -5.1 | 4 | 5.8 | 598\|890\|1991\|2099\|2268\|2870\|3676\|4317\|4353\|5144\|6850\|6916\|7124\|9212\|10013 | BCL2L1\|CCNA2\|ELANE\|ESR1\|FGR\|GRK6\|ITGA4\|MMP8\|MPO\|PDE4D\|SYK\|TBXAS1\|TNF\|AURKB\|HDAC6 | -4.1 |
|  |  | PGB:00072 | PaGenBase | Tissue-specific: uterus | -5 | 6.5 | 6.5 | 2099\|3357\|3558\|4316\|4321\|5241\|5733\|10846\|56547 | ESR1\|HTR2B\|IL2\|MMP7\|MMP12\|PGR\|PTGER3\|PDE10A\|MMP26 | -3.9 |
|  |  | PGB:00016 | PaGenBase | Tissue-specific: thymus | -4.9 | 4 | 5.6 | 135\|1136\|1814\|2322\|3363\|3676\|3702\|3932\|4012\|4194\|4321\|7294\|9612\|11238 | ADORA2A\|CHRNA3\|DRD3\|FLT3\|HTR7\|ITGA4\|ITK\|LCK\|LNPEP\|MDM4\|MMP12\|TXK\|NCOR2\|CA5B | -3.8 |
|  |  | PGB:00045 | PaGenBase | Tissue-specific: placenta | -4.8 | 3.4 | 5.4 | 412\|649\|898\|1017\|1588\|1612\|1956\|2321\|3292\|3294\|3480\|3569\|5328\|6530\|6532\|7430\|9536 | STS\|BMP1\|CCNE1\|CDK2\|CYP19A1\|DAPK1\|EGFR\|FLT1\|HSD17B1\|HSD17B2\|IGF1R\|IL6\|PLAU\|SLC6A2\|SLC6A4\|EZR\|PTGES | -3.8 |
|  |  | PGB:00031 | PaGenBase | Cell-specific: HUVEC | -4.7 | 3.1 | 5.2 | 558\|595\|660\|840\|1901\|2048\|2152\|2321\|2833\|3269\|4312\|4321\|4846\|5340\|5599\|6095\|6326\|7299\|23450 | AXL\|CCND1\|BMX\|CASP7\|S1PR1\|EPHB2\|F3\|FLT1\|CXCR3\|HRH1\|MMP1\|MMP12\|NOS3\|PLG\|MAPK8\|RORA\|SCN2A\|TYR\|SF3B3 | -3.7 |
|  |  | PGB:00095 | PaGenBase | Cell-specific: LNCAP | -4.5 | 9.7 | 6.9 | 150\|231\|367\|3291\|4128\|5737 | ADRA2A\|AKR1B1\|AR\|HSD11B2\|MAOA\|PTGFR | -3.5 |
|  |  | PGB:00060 | PaGenBase | Tissue-specific: retinoblastoma | -4.4 | 5.5 | 5.9 | 623\|1513\|4312\|4314\|4323\|4843\|5739\|7298\|7421 | BDKRB1\|CTSK\|MMP1\|MMP3\|MMP14\|NOS2\|PTGIR\|TYMS\|VDR | -3.4 |
|  |  | PGB:00079 | PaGenBase | Tissue-specific: Caudate nucleus | -4.2 | 8.8 | 6.5 | 135\|2100\|2890\|2915\|3358\|10846 | ADORA2A\|ESR2\|GRIA1\|GRM5\|HTR2C\|PDE10A | -3.3 |
|  |  | PGB:00004 | PaGenBase | Tissue-specific: kidney | -4.1 | 2.6 | 4.6 | 152\|596\|760\|771\|983\|1591\|1815\|2103\|2798\|3291\|4282\|4316\|4543\|4886\|5465\|5599\|5733\|6524\|7364\|9356\|50507 | ADRA2C\|BCL2\|CA2\|CA12\|CDK1\|CYP24A1\|DRD4\|ESRRB\|GNRHR\|HSD11B2\|MIF\|MMP7\|MTNR1A\|NPY1R\|PPARA\|MAPK8\|PTGER3\|SLC5A2\|UGT2B7\|SLC22A6\|NOX4 | -3.2 |
|  |  | PGB:00078 | PaGenBase | Cell-specific: liver cell | -3.9 | 5.4 | 5.4 | 1646\|2147\|2152\|2167\|2168\|7276\|7364\|8644 | AKR1C2\|F2\|F3\|FABP4\|FABP1\|TTR\|UGT2B7\|AKR1C3 | -3 |
|  |  | PGB:00057 | PaGenBase | Tissue-specific: ovary | -3.6 | 3.2 | 4.4 | 1137\|1588\|2048\|2099\|2100\|2946\|3290\|5241\|8972\|10054\|10846\|29881\|134864 | CHRNA4\|CYP19A1\|EPHB2\|ESR1\|ESR2\|GSTM2\|HSD11B1\|PGR\|MGAM\|UBA2\|PDE10A\|NPC1L1\|TAAR1 | -2.6 |
|  |  | PGB:00008 | PaGenBase | Tissue-specific: small intestine | -3.5 | 4.2 | 4.7 | 147\|1268\|2101\|4312\|4321\|6523\|11200\|29881\|57016 | ADRA1B\|CNR1\|ESRRA\|MMP1\|MMP12\|SLC5A1\|CHEK2\|NPC1L1\|AKR1B10 | -2.6 |
|  |  | PGB:00047 | PaGenBase | Cell-specific: THY+ | -3.2 | 4.3 | 4.6 | 239\|816\|873\|2099\|3738\|3786\|6714\|9149 | ALOX12\|CAMK2B\|CBR1\|ESR1\|KCNA3\|KCNQ3\|SRC\|DYRK1B | -2.4 |
|  |  | PGB:00137 | PaGenBase | Cell-specific: LN18 | -3.2 | 16 | 6.6 | 1513\|3294\|6916 | CTSK\|HSD17B2\|TBXAS1 | -2.3 |
|  |  | PGB:00041 | PaGenBase | Tissue-specific: Blood | -3.1 | 3 | 4.1 | 1432\|1520\|2194\|2268\|2357\|3577\|3689\|4067\|5293\|5777\|5836\|10135 | MAPK14\|CTSS\|FASN\|FGR\|FPR1\|CXCR1\|ITGB2\|LYN\|PIK3CD\|PTPN6\|PYGL\|NAMPT | -2.3 |
|  |  | PGB:00018 | PaGenBase | Tissue-specific: lung | -3.1 | 2.3 | 3.8 | 151\|246\|247\|762\|765\|1991\|3269\|3383\|3569\|4233\|4317\|4322\|4353\|5319\|5743\|5771\|6532\|8856\|134864 | ADRA2B\|ALOX15\|ALOX15B\|CA4\|CA6\|ELANE\|HRH1\|ICAM1\|IL6\|MET\|MMP8\|MMP13\|MPO\|PLA2G1B\|PTGS2\|PTPN2\|SLC6A4\|NR1I2\|TAAR1 | -2.2 |
|  |  | PGB:00015 | PaGenBase | Tissue-specific: Smooth Muscle | -2.8 | 4.1 | 4.1 | 136\|558\|3569\|4312\|5742\|5743\|10769 | ADORA2B\|AXL\|IL6\|MMP1\|PTGS1\|PTGS2\|PLK2 | -1.9 |
|  |  | PGB:00007 | PaGenBase | Tissue-specific: colon | -2.7 | 2.5 | 3.6 | 759\|760\|766\|840\|1080\|2859\|3569\|5143\|5152\|5595\|5753\|5979\|7015\|27035 | CA1\|CA2\|CA7\|CASP7\|CFTR\|GPR35\|IL6\|PDE4C\|PDE9A\|MAPK3\|PTK6\|RET\|TERT\|NOX1 | -1.9 |
|  |  | PGB:00064 | PaGenBase | Cell-specific: RPMI 8226 | -2.7 | 7.3 | 4.7 | 412\|3695\|10203\|11040 | STS\|ITGB7\|CALCRL\|PIM2 | -1.8 |
|  |  | PGB:00081 | PaGenBase | Cell-specific: Bronchial Epithelial Cells | -2.7 | 3.5 | 3.8 | 771\|1956\|2152\|4233\|5328\|5743\|5792\|7378 | CA12\|EGFR\|F3\|MET\|PLAU\|PTGS2\|PTPRF\|UPP1 | -1.8 |
|  |  | PGB:00120 | PaGenBase | Cell-specific: Cardiac Myocytes | -2.5 | 5 | 4 | 558\|3569\|4312\|5327\|10769 | AXL\|IL6\|MMP1\|PLAT\|PLK2 | -1.7 |
|  |  | PGB:00146 | PaGenBase | Tissue-specific: hippocampus | -2.4 | 9.4 | 4.8 | 2043\|7150\|7525 | EPHA4\|TOP1\|YES1 | -1.6 |
|  |  | PGB:00065 | PaGenBase | Tissue-specific: Breast | -2.4 | 3.2 | 3.5 | 231\|367\|1591\|1646\|2049\|3356\|4233\|5021 | AKR1B1\|AR\|CYP24A1\|AKR1C2\|EPHB3\|HTR2A\|MET\|OXTR | -1.6 |
|  |  | PGB:00056 | PaGenBase | Tissue-specific: spinal cord | -2.4 | 4.7 | 3.8 | 1128\|2152\|2342\|3558\|60482 | CHRM1\|F3\|FNTB\|IL2\|SLC5A7 | -1.6 |
|  |  | PGB:00115 | PaGenBase | Cell-specific: CD33+ Myeloid | -2.3 | 5.8 | 4 | 1066\|4170\|7099\|10135 | CES1\|MCL1\|TLR4\|NAMPT | -1.5 |
|  |  | PGB:00067 | PaGenBase | Cell-specific: Brain cell | -2.3 | 4.5 | 3.7 | 4313\|5156\|5328\|5733\|5743 | MMP2\|PDGFRA\|PLAU\|PTGER3\|PTGS2 | -1.5 |
|  |  | PGB:00080 | PaGenBase | Cell-specific: huh-7 | -2.2 | 7.8 | 4.3 | 2168\|7276\|9971 | FABP1\|TTR\|NR1H4 | -1.4 |
|  |  | PGB:00131 | PaGenBase | Cell-specific: B-lymphocyte | -2.2 | 5.3 | 3.8 | 3695\|9429\|10892\|11040 | ITGB7\|ABCG2\|MALT1\|PIM2 | -1.4 |
|  |  | PGB:00022 | PaGenBase | Tissue-specific: adrenal gland | -2.2 | 2.7 | 3.1 | 706\|1584\|1585\|1586\|3777\|5156\|5243\|5409\|6095 | TSPO\|CYP11B1\|CYP11B2\|CYP17A1\|KCNK3\|PDGFRA\|ABCB1\|PNMT\|RORA | -1.4 |
|  |  | PGB:00087 | PaGenBase | Cell-specific: Lymphoma burkitts Raji | -2 | 3 | 3.1 | 590\|1269\|4047\|4282\|7421\|9970\|10783 | BCHE\|CNR2\|LSS\|MIF\|VDR\|NR1I3\|NEK6 | -1.3 |
|  |  | PGB:00026 | PaGenBase | Cell-specific: CD71+ EarlyErythroid | -1.9 | 2.8 | 2.9 | 383\|598\|759\|760\|5610\|6622\|9429 | ARG1\|BCL2L1\|CA1\|CA2\|EIF2AK2\|SNCA\|ABCG2 | -1.2 |
|  |  | PGB:00032 | PaGenBase | Tissue-specific: cortex | -1.8 | 4.3 | 3.2 | 3064\|3984\|5141\|6532 | HTT\|LIMK1\|PDE4A\|SLC6A4 | -1.1 |
|  |  | PGB:00084 | PaGenBase | Tissue-specific: Lymph node | -1.6 | 3.1 | 2.7 | 1018\|3785\|5594\|6916\|11200 | CDK3\|KCNQ2\|MAPK1\|TBXAS1\|CHEK2 | -0.87 |
|  |  | PGB:00063 | PaGenBase | Cell-specific: OVR278S | -1.6 | 3.6 | 2.8 | 2043\|5465\|5753\|8658 | EPHA4\|PPARA\|PTK6\|TNKS | -0.86 |
|  |  | PGB:00101 | PaGenBase | Tissue-specific: Colorectal adenocarcinoma | -1.5 | 4.4 | 2.8 | 595\|6790\|7153 | CCND1\|AURKA\|TOP2A | -0.76 |
|  |  | PGB:00073 | PaGenBase | Tissue-specific: salivary gland | -1.4 | 2.4 | 2.3 | 765\|2049\|5578\|5802\|9149\|27035 | CA6\|EPHB3\|PRKCA\|PTPRS\|DYRK1B\|NOX1 | -0.68 |
|  |  | PGB:00107 | PaGenBase | Cell-specific: SHSYSY-RA | -1.4 | 4 | 2.6 | 1136\|3785\|5979 | CHRNA3\|KCNQ2\|RET | -0.66 |
|  |  | PGB:00068 | PaGenBase | Tissue-specific: Stomach | -1.3 | 3.7 | 2.5 | 768\|4282\|8989 | CA9\|MIF\|TRPA1 | -0.6 |
|  | VMC-related targets | PGB:00031 | PaGenBase | Cell-specific: HUVEC | -8.2 | 9.4 | 9.6 | 595\|840\|1901\|2152\|2321\|2833\|3269\|4312\|4846\|5340\|5599\|7299 | CCND1\|CASP7\|S1PR1\|F3\|FLT1\|CXCR3\|HRH1\|MMP1\|NOS3\|PLG\|MAPK8\|TYR | -6.9 |
|  |  | PGB:00011 | PaGenBase | Tissue-specific: spleen | -5.9 | 6.4 | 7.2 | 640\|3676\|3683\|3702\|4312\|4318\|4846\|5294\|5551\|7099\|54106 | BLK\|ITGA4\|ITGAL\|ITK\|MMP1\|MMP9\|NOS3\|PIK3CG\|PRF1\|TLR4\|TLR9 | -4.7 |
|  |  | PGB:00018 | PaGenBase | Tissue-specific: lung | -3.5 | 4.7 | 4.9 | 1991\|3269\|3383\|3569\|4233\|4317\|4353\|6532 | ELANE\|HRH1\|ICAM1\|IL6\|MET\|MMP8\|MPO\|SLC6A4 | -2.5 |
|  |  | PGB:00060 | PaGenBase | Tissue-specific: retinoblastoma | -3.5 | 12 | 6.4 | 4312\|4314\|4843\|7421 | MMP1\|MMP3\|NOS2\|VDR | -2.4 |
|  |  | PGB:00048 | PaGenBase | Tissue-specific: bone marrow | -2.9 | 6.4 | 4.8 | 1991\|3676\|4317\|4353\|7124 | ELANE\|ITGA4\|MMP8\|MPO\|TNF | -2 |
|  |  | PGB:00120 | PaGenBase | Cell-specific: Cardiac Myocytes | -2.9 | 15 | 6.2 | 3569\|4312\|5327 | IL6\|MMP1\|PLAT | -2 |
|  |  | PGB:00036 | PaGenBase | Cell-specific: MOLT4 | -2.9 | 8.5 | 5.2 | 3702\|3932\|4312\|5578 | ITK\|LCK\|MMP1\|PRKCA | -1.9 |
|  |  | PGB:00049 | PaGenBase | Cell-specific: Adipocyte | -2.8 | 8 | 5 | 2152\|2194\|4313\|5468 | F3\|FASN\|MMP2\|PPARG | -1.8 |
|  |  | PGB:00002 | PaGenBase | Cell-specific: HEPG2 | -2.5 | 4.1 | 3.8 | 2147\|2152\|4233\|5781\|6198\|6532 | F2\|F3\|MET\|PTPN11\|RPS6KB1\|SLC6A4 | -1.5 |
|  |  | PGB:00045 | PaGenBase | Tissue-specific: placenta | -2.4 | 4.8 | 3.9 | 1956\|2321\|3569\|6530\|6532 | EGFR\|FLT1\|IL6\|SLC6A2\|SLC6A4 | -1.4 |
|  |  | PGB:00016 | PaGenBase | Tissue-specific: thymus | -2.2 | 5.5 | 3.9 | 135\|3676\|3702\|3932 | ADORA2A\|ITGA4\|ITK\|LCK | -1.3 |
|  |  | PGB:00001 | PaGenBase | Tissue-specific: liver | -2 | 2.9 | 3 | 383\|1557\|1956\|2147\|2152\|5340\|6530 | ARG1\|CYP2C19\|EGFR\|F2\|F3\|PLG\|SLC6A2 | -1.1 |
|  |  | PGB:00081 | PaGenBase | Cell-specific: Bronchial Epithelial Cells | -1.9 | 6.4 | 3.7 | 1956\|2152\|4233 | EGFR\|F3\|MET | -0.98 |
|  |  | PGB:00014 | PaGenBase | Cell-specific: DRG | -1.7 | 3.2 | 2.8 | 43\|5468\|5979\|6530\|7941 | ACHE\|PPARG\|RET\|SLC6A2\|PLA2G7 | -0.74 |
|  |  | PGB:00007 | PaGenBase | Tissue-specific: colon | -1.5 | 3.5 | 2.7 | 840\|3569\|5595\|5979 | CASP7\|IL6\|MAPK3\|RET | -0.59 |
|  |  | PGB:00022 | PaGenBase | Tissue-specific: adrenal gland | -1.5 | 4.4 | 2.8 | 706\|5243\|5409 | TSPO\|ABCB1\|PNMT | -0.55 |
|  |  | PGB:00041 | PaGenBase | Tissue-specific: Blood | -1.3 | 3.7 | 2.4 | 1432\|2194\|3689 | MAPK14\|FASN\|ITGB2 | -0.37 |

| **Table S11. Potential match relation between component and target.** | | |
| --- | --- | --- |
| **Target** | **Component ID** | **Index** |
| CHRM2 | MOL000033 | 1 |
|  | MOL000358 | 1 |
|  | MOL006824 | 1 |
|  | MOL003680 | 1 |
|  |  | 2 |
|  | MOL005944 | 1 |
|  |  | 2 |
|  | MOL006562 | 1 |
|  |  | 2 |
|  | MOL006564 | 1 |
|  |  | 2 |
|  | MOL006565 | 1 |
|  |  | 2 |
|  | MOL000449 | 1 |
|  | MOL000953 | 1 |
|  | MOL004653 | 1 |
|  | MOL000506 | 1 |
|  |  | 2 |
| FABP3 | MOL000211 | 1 |
|  | MOL006824 | 1 |
|  | MOL000469 | 1 |
| TNNC1-TNNI3-TNNT2 | MOL001002 | 1 |
|  |  | 2 |
|  |  | 3 |
|  |  | 4 |
|  |  | 5 |
|  |  | 6 |
|  |  | 7 |
|  |  | 8 |
|  |  | 9 |
|  | MOL006570 | 1 |
|  |  | 2 |
|  | MOL006582 | 1 |
|  |  | 2 |
|  | MOL000456 | 1 |
| MMP1 | MOL000378 | 1 |
|  | MOL000438 | 1 |
|  | MOL006604 | 1 |
|  |  | 2 |
|  |  | 3 |
|  | MOL006623 | 1 |
|  |  | 2 |
|  | MOL006626 | 1 |
|  |  | 2 |
|  |  | 3 |
|  |  | 4 |
|  |  | 5 |
|  | MOL004653 | 1 |
|  | MOL000483 | 1 |
|  |  | 2 |
|  | MOL000491 | 1 |
|  | MOL000497 | 1 |
|  |  | 2 |
|  |  | 3 |
|  |  | 4 |
|  |  | 5 |
